# Parentage‐based tagging combined with genetic stock identification is a cost‐effective and viable replacement for coded‐wire tagging in large‐scale assessments of marine Chinook salmon fisheries in British Columbia, Canada

**DOI:** 10.1111/eva.13203

**Published:** 2021-03-19

**Authors:** Terry D. Beacham, Colin G. Wallace, Kim Jonsen, Brenda McIntosh, John R. Candy, Katherine Horst, Cheryl Lynch, David Willis, Wilf Luedke, Lee Kearey, Eric B. Rondeau

**Affiliations:** ^1^ Fisheries and Oceans Canada Pacific Biological Station Nanaimo British Columbia Canada; ^2^ Fisheries and Oceans Canada Regional Headquarters Vancouver British Columbia Canada; ^3^ Fisheries and Oceans Canada South Coast Stock Assessment Division Nanaimo British Columbia Canada

**Keywords:** Chinook salmon, coded‐wire tags, fishery management, genetic stock identification, genotyping by sequencing, parentage‐based tagging

## Abstract

Wild Pacific salmon, including Chinook salmon *Oncorhynchus tshawytscha*, have been supplemented with hatchery propagation for over 50 years in support of increased ocean harvest, mitigation for hydroelectric development, and conservation of threatened populations. In Canada, the Wild Salmon Policy for Pacific salmon was established with the goal of maintaining and restoring healthy and diverse Pacific salmon populations, making conservation of wild salmon and their habitats the highest priority for resource management decision‐making. For policy implementation, a new approach to the assessment and management of Chinook salmon and the associated hatchery production and fisheries management are needed. Implementation of genetic stock identification (GSI) and parentage‐based tagging (PBT) for marine fisheries assessment may overcome problems associated with coded‐wire tag‐based (CWT) assessment and management of Chinook salmon fisheries, providing at a minimum information equivalent to that derived from the CWT program. GSI and PBT were used to identify Chinook salmon sampled in 2018 and 2019 marine fisheries (18,819 individuals genotyped) in British Columbia to specific conservation units (CU), populations, and broodyears. Individuals were genotyped at 391 single nucleotide polymorphisms via direct sequencing of amplicons. Very high accuracy of assignment to population and age (>99.5%) via PBT was observed for 1994 Chinook salmon of ages 2–4 years, with a 105,722–individual, 380–population baseline available for assignment. Application of a GSI‐PBT system of identification to individuals in 2019 fisheries provided high‐resolution estimates of stock composition, catch, and exploitation rate by CU or population, with fishery exploitation rates directly comparable to those provided by CWTs for 13 populations. GSI and PBT provide an alternate, cheaper, and more effective method in the assessment and management of Canadian‐origin Chinook salmon relative to CWTs, and an opportunity for a genetics‐based system to replace the current CWT system for salmon assessment.

## 1 INTRODUCTION

Supplementation of Pacific salmon abundance via hatchery production has been conducted for many years in Canada with two principal objectives, primarily increasing ocean harvest for selected species or enhancing production from specific populations of conservation concern. Canada initiated a Salmonid Enhancement Program in the 1970s with the objective of doubling catch of Pacific salmon in British Columbia (BC; Hilborn & Winton, 1993). This objective was never achieved, but led to the simultaneous exploitation of hatchery‐enhanced and wild populations in mixed‐stock fisheries, potentially leading to over‐exploitation of wild populations. Management of mixed‐stock fisheries is a matter of continuing concern (Flagg, 2015; HSRG, 2014). The effects of straying of hatchery‐produced individuals into wild‐spawning populations are also of concern (Araki et al. 2008; Jones et al. 2018; McClure et al. 2008). Canada responded to concerns over declining wild population abundance by developing the Policy for Conservation of Wild Pacific Salmon (WSP; Fisheries & Oceans Canada, 2005) with the goal of maintaining and restoring healthy and diverse Pacific salmon populations, making conservation of wild salmon and their habitats the highest priority for resource management decision‐making. Under the WSP, wild salmon populations are identified and maintained in Conservation Units (CUs) that are identified based on genetic traits, biogeographic distribution, life‐history characteristics, and local knowledge where available. For Chinook salmon (*Oncorhynchus tshawytscha*), 84 CUs have been defined for Canadian populations.

As some Chinook salmon populations are enhanced through hatchery production, easy access to the juveniles produced is obtained. To aid in assessment of mixed‐stock fisheries, some portion of the juveniles is marked with coded‐wire tags (CWTs; Jefferts et al. 1963) prior to release from the hatchery. The presence of these tags is implied in returning adults either by absence of the adipose fin which was clipped at the time of tagging or by means of an electronic tag detection (ETD) system applied to individual salmon sampled in fisheries, hatcheries, or on the spawning grounds. These “indicator” populations, coupled with CWT marking of selected wild populations in the United States but generally not in Canada, provide the basis for current fishery assessment and management regimes for Chinook salmon. The key assumption underlying an assessment method employing indicator populations is that the indicator population displays characteristics that are representative of the other untagged (naturally spawning) populations within the management unit or geographic region that it is intended to represent. Once recovered, the tags are decoded to determine the hatchery origin and age of the individual fish. Originally, only individuals marked with a CWT also received an adipose fin clip prior to hatchery release, with the externally visible clip mark allowing CWT‐marked fish to be identified visually and sampled from fisheries or river collections. However, since the 1990s, in order to facilitate fisheries that exploited Chinook salmon produced only in hatcheries, most Chinook salmon released from many hatcheries in Washington, Oregon, and the Columbia River drainage were mass marked by receiving an adipose fin clip, but not necessarily a corresponding CWT.

The Chinook Technical Committee of the Pacific Salmon Commission (PSC) uses a Chinook Model to generate key outputs of relevance to the PSC’s annual fishery management cycle, where preseason prediction of abundance is a key driver of subsequent fisheries in southeast Alaska and BC (PSC‐CTC, 2018). Fishery exploitation rates are derived from CWT recoveries and are used in model calibration. There is also a Fisheries Regulation Assessment Model (FRAM) that is used as the primary analytical and assessment tool for fisheries off the U.S. west coast, where CWT recoveries are used to estimate stock‐specific abundance and exploitation rates (PFMC, 2008). The utility of the CWT system for fisheries assessment has been eroded by the extensive release of the previously mentioned adipose fin‐clipped individuals without CWTs (mass marking). Without coastwide implementation of electronic sampling, inadequate application and recovery of double‐index CWTs, as well as misalignment of fisheries regulations with assessment programs may occur (PSCSFEC, 2016). Additionally, significant deficiencies in FRAM model predictions for Chinook salmon fishery assessment due to the use of incomplete and outdated baseline data have recently been demonstrated (Moran et al. 2018). The limited number of populations currently marked with CWTs in BC represents only 25% of the 84 CUs defined for Chinook salmon under Canada's WSP, restricting wide‐scale assessment of fishery impacts on existing CUs via indicator populations.

The necessity of maintaining a viable CWT system for Chinook salmon assessment was recognized under the Pacific Salmon Treaty between Canada and the United States through a Memorandum of Understanding. In 2004, given the impairment of CWT recovery through mass marking of some American hatchery production, the PSC convened an expert panel to examine limitations of the CWT program and to evaluate the capacity of alternative technologies to provide data to improve assessment of Chinook salmon. One finding of the panel was that a parentage‐based tagging (PBT) approach as proposed by Anderson and Garza (2005) could provide the equivalent of CWT recovery data (hatchery of release and age of the sampled individual), and could be easily integrated with a genetic stock identification (GSI) system to provide stock of origin for all fish from PBT hatcheries (PSC, 2005). There was a recognition that a genetics‐based assessment method could provide equivalent information to that of a CWT‐based method, but an empirical demonstration of the equivalency was mandatory.

A rockslide in the middle portion of the Fraser River drainage in southern BC was discovered in late 2018 which was recognized as having the potential of severely impeding upstream migration of salmon utilizing spawning habitat in the middle and upper portions of the drainage. Accordingly, substantial restrictions on Chinook salmon fisheries in BC were implemented in 2019 in order to minimize fisheries exploitation on populations that would utilize this habitat. There was prior minimal hatchery enhancement of populations in the region, and the restrictive fisheries management regime implemented heightened interest among some stakeholders in mark‐selective (adipose fin clip) fisheries that would target hatchery production from other areas. In BC, mass marking of Chinook salmon hatchery production via an adipose fin clip has not been implemented, although a pilot project was initiated for mass marking of Sarita River and Conuma River hatchery production on the west coast of Vancouver Island (WCVI) in 2020. There is the potential for more substantial mass marking of BC hatchery Chinook salmon production in the future. Mass marking via an adipose fin clip can impair the recovery of CWTs, as now many adipose fin‐clipped individuals do not carry a CWT, thus requiring operation of an ETD system to allow practical recovery of CWTs. In spite of operation of an ETD system to screen a portion of the commercial catch to identify salmon with a CWT, and increased tagging rates on the indicator populations, reduced marine survival rates due to a prolonged low productivity regime in the Pacific Northwest and associated low harvest rates has resulted in fewer tags obtained from the current CWT assessment program. If a program of routine mass marking of Chinook salmon hatchery production in BC is implemented, further expense and difficulty in CWT recovery for Chinook salmon in BC may occur.

A genetics‐based assessment method can incorporate both GSI and PBT methods to produce high‐resolution stock composition and age structure of catch in mixed‐stock fisheries. As proposed by Anderson and Garza (2005), illustrated as potentially possible by Anderson and Garza (2006), and outlined by Steele et al. (2019), PBT uses molecular‐based approaches to conduct large‐scale parentage assignments and has resulted in the unprecedented ability to identify genetically millions of hatchery‐origin salmonids. Assignments are made to parents of known origin, and with that information, it is possible to determine the origin and age of individuals sampled in fisheries. Application of a GSI‐PBT system of identification of coho salmon (*O. kisutch*) in fisheries and escapements (number of salmon that “escape” fisheries and return to fresh water to spawn) in BC provided high‐resolution estimates of stock composition, catch, and exploitation rate by CU or population, providing an alternate and more effective method in the assessment and management of Canadian‐origin coho salmon relative to CWTs (Beacham, Wallace, Jonsen, McIntosh, Candy, Willis, Lynch, Moore et al. 2019).

Recent molecular and analytical improvements have made it possible for a genetics‐based assessment system to provide an alternative to the current CWT‐based system. Direct DNA sequencing, coupled with automated scoring of the genotypes, results in cost‐effective genotyping and unprecedented ability to provide accurate estimates of stock composition or individual identification to very discrete geographic regions or CUs. As noted by Beacham, Wallace, et al. (2020), it is a new era in the application of genetic variation to resource management and forensic analysis. Integration of PBT and GSI into a single application can produce fishery stock composition estimates of very high resolution, as well as origin and age of individuals sampled when identified via PBT, the same information as provided by CWTs as first described by Anderson and Garza (2005) and subsequently demonstrated in studies by Hess et al. (2016), Beacham et al. (2017, 2018), Beacham, Wallace, Jonsen, McIntosh, Candy, Willis, Lynch, Moore et al. (2019) and Steele et al. (2019).

Chinook salmon is the most important Pacific salmon species in terms of CWT application, the most diverse in terms of age structure of returning adults, and was the species of most concern to a panel examining deficiencies in the CWT program (PSC, 2005). The challenge in evaluating a PBT application in Chinook salmon is equivalent to that posed with coho salmon; specifically, this requires that a GSI‐PBT approach provide the equivalent of CWT recovery data in an empirical demonstration on a coastal scale. Age at maturity in Chinook salmon in BC (mainly ages 2–6 years) is more variable than that of coho salmon (2–4 years), and thus would potentially present the largest challenge for Pacific salmon in correct assignment of individuals via PBT, as correct assignment to wider age span is required. Beacham et al. (2018) provided an initial indication that implementation of a GSI‐PBT evaluation method may be possible for Chinook salmon. However, empirical demonstration of correct age assignments across the full suite of age of return was not available, nor was empirical demonstration of the technology to mixed‐stock fishery samples available, where both GSI and PBT are applied in estimation of stock composition, with a coastwide baseline of populations available for utilization in the analyses.

The current study is an evaluation of the application of the GSI‐PBT methodology outlined by Beacham et al. (2018) to selected Chinook salmon fisheries in BC to determine whether GSI and PBT can be used to provide more information on fishery contributions by hatchery and CU than is available from CWTs. Several improvements have been made to the methods and results outlined by Beacham et al. (2018). First, the single nucleotide polymorphism (SNP) panel has been enhanced from 321 to 391 SNPs available for genotyping. Second, the baseline has been substantially enhanced to include populations from Russia, Alaska, the Yukon Territory, BC, the Pacific Northwest, and California, allowing greater resolution in estimation of stock composition in samples from mixed‐stock fisheries. Third, we evaluated the population‐level resolution obtained from CWTs and the GSI‐PBT methodology by CU for some 2018 and 2019 fisheries in which Chinook salmon were caught, along with catch estimation by CU for the fisheries sampled. Complete broodstock genotyping for PBT analysis was conducted since 2013 for selected hatchery‐enhanced populations, and a stock identification baseline comprising some 380 populations ranging from Russia to California was employed for GSI. After evaluation of the results of the fishery sampling program in 2019, we conclude that a genetic approach can emulate and improve upon the results available from the current CWT program for assessment and management of Canadian Chinook salmon enhancement and fisheries in BC, and provide critical information to improve wild Chinook salmon assessment and conservation.

## 2 METHODS

### 2.1 Fishery sample collection

**FIGURE 1:**
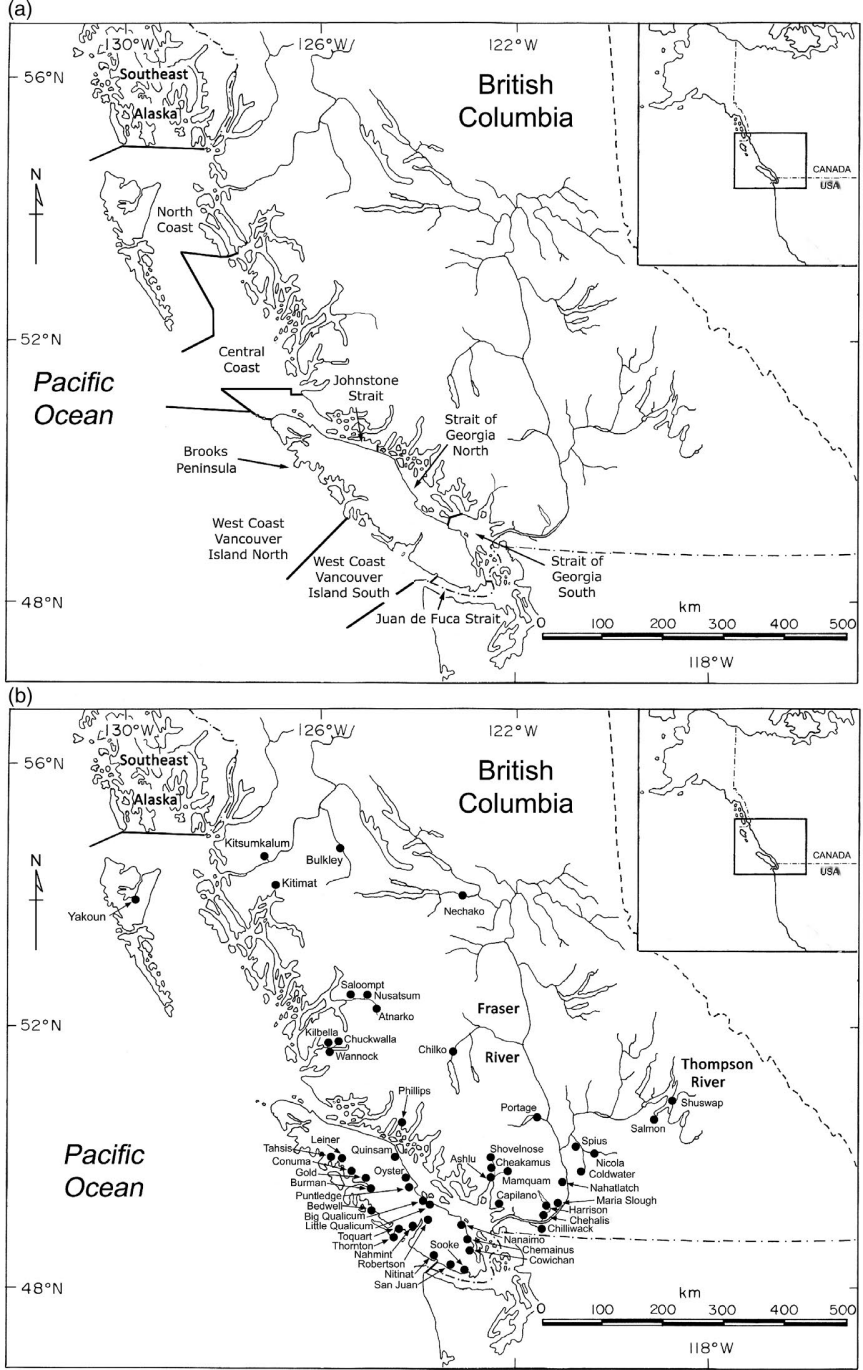
Map indicating geographic locations for fishery sampling (a) and 48 populations for which parentage‐based tagging was applied in estimation of stock composition or origins of 2019 hatchery broodstocks (b)

The initial sampling for both GSI and PBT application occurred in 2018 fisheries, as 2018 marked the first year in which PBT identifications could potentially be made across the suite of most likely age of return, and the GSI baseline was ready for initial application. The intent of the fishery analysis in 2018 was to evaluate the extent to which PBT identifications could be made in commercial and recreational fisheries, as well as to evaluate the performance of the GSI baseline for mixed‐stock analysis. A total of 6286 individuals were genotyped from fishery samples collected in 2018. Samples were pooled for analysis by fishery and gear as outlined in Figure 1a. Fisheries that occurred within the defined geographic regions were separated by sector and gear (commercial troll, commercial net, recreational, First Nations, and test fisheries).

In the northern BC troll fishery, offloads were selected at random and every 5th or 10th fish was selected from the load to a maximum of 50 fish from a single load. Individuals with a CWT were not excluded from the sample. Tissue samples from sampled individuals were subsequently provided for genotyping. Samples from the northern BC recreational fishery were provided by operators of fishing lodges or through direct Fisheries and Oceans Canada (DFO) creel sampling. Samples were also obtained from a gillnet test fishery at Tyee operating at the mouth of the Skeena River.

In southern BC, samples were available from a First Nations troll fishery (T’aaq‐wiihak fishery) conducted off the WCVI. In the WCVI creel survey, if individual fishers agreed, heads of Chinook salmon were sampled and otoliths collected for subsequent possible hatchery identification, as well as a tissue sample for genetic analysis. Samples from the 2018 WCVI recreational fishery were genotyped subject to their having been screened previously for individuals identified via CWT or by a specific hatchery otolith mark. Individuals with an adipose fin clip but no CWT were deemed to be of US origin. If individuals were identified by these methods, then they were not subsequently provided for genotyping. In the Strait of Georgia (SoG) and the Juan de Fuca Strait (JDF) recreational fishery off Victoria, BC, samples from the recreational fishery were obtained from a DFO creel survey program supplemented by samples provided by the Avid Anglers. The Avid Anglers are a “citizen science” group of volunteers who, if given the opportunity, fish year round, and collect biological information and tissue samples. The Capilano River hatchery derby samples were obtained from a recreational fishery that occurred in July within approximately 15 km of the mouth of the Capilano River. Johnstone Strait recreational samples were obtained from the creel survey program. In each sample analyzed over all BC fisheries, the number of individuals identified via PBT relative to the number of genotypes in the sample was tabulated, and summarized over sample, fishery, and season.

Minimum size limits were in effect for recreational fisheries in the SoG, with individuals <62 cm fork length defined as sublegal and required to be released. The origins of the sublegal individuals were unknown via CWTs, as the CWT could not be recovered from an individual released alive, and sublegal individuals would not be observed in the creel sampling program. Fin clip samples were obtained from released sublegal individuals via the Avid Anglers sampling program, and GSI and PBT analyses were subsequently conducted on these individuals.

Given the encouraging results from the analyses of 2018 fishery samples for both PBT and GSI, the objective of fishery sampling in 2019 was to provide an empirical demonstration of numbers of CWT and PBT identifications of Canadian‐origin individuals observed in fisheries, and subsequently estimation of age‐specific exploitation rate in specific fisheries in BC. As noted previously, fishery restrictions were imposed in 2019 in order to minimize exploitation of middle and upper Fraser River drainage populations, as well as to support conservation priorities. The opening of the northern troll fishery was delayed until August 20th, and the WCVI troll fishery to August 1st. Non‐retention of Chinook salmon was implemented in the Johnstone Strait and northern SoG recreational fishery until July 14th, in the southern SoG and the JDF until July 31st, and in the offshore WCVI waters until July 14th. Despite later fishery openings, a total of 12,533 individuals was genotyped from fishery samples collected in 2019.

In northern BC, the 2019 commercial troll fishery, recreational fisheries, and Skeena River test fishery were sampled as in 2018, with greater numbers of individuals genotyped. Additional fisheries sampled included the central coast recreational fishery through addition of samples from a fishing lodge, a First Nation food, social, and ceremonial fishery, and a central coast terminal gillnet fishery. In southern BC, samples were obtained from the WCVI troll fishery, a test fishery near Brooks Peninsula, an Alberni Inlet gillnet fishery, and the WCVI recreational fishery. Unlike 2018, CWT‐marked individuals, as well as individuals containing a hatchery otolith mark were also included in the WCVI recreational samples that were genotyped. Samples from Avid Anglers again supplemented samples from the DFO creel survey program in the SoG and Victoria area, with the Capilano River derby samples genotyped as well. As in 2018, Johnstone Strait recreational samples were obtained from the DFO creel survey program.

### 2.2 Evaluating accuracy of PBT age determination

Samples of 1333 adipose fins of juveniles that were clipped as part of CWT marking were obtained in 2017 from 16 hatchery populations, as these individuals were the offspring of the hatchery broodstock sampled in 2016. These juveniles constituted samples of known origin and age and were subsequently used in evaluation of population and broodyear assignment, with the baseline available for potential parentage assignment including hatchery broodstocks genotyped prior to 2017. Genetic tagging rates in these 16 populations were calculated as outlined by Satterthwaite et al. (2015). Tagging rate in a year for two‐parent assignments was estimated as = (the proportion of the broodstock successfully genotyped)^2^. Genetic tagging rates for single‐parent assignments was estimated as = 1 − (proportion of broodstock not genotyped)^2^. The expected number of PBT identifications was estimated as sample size × genetic tagging rate for both two‐parent and one‐parent identifications.

In 2017, hatchery broodstocks were genotyped, and at four hatcheries it was possible to match individuals that were marked with CWTs with age and origin of individuals estimated via PBT. The objective of the analysis was to evaluate accuracy of assignments of the individuals marked with CWTs with respect to population and age. The baseline for potential assignment included hatchery broodstocks genotyped prior to 2017. Once the parents of the juveniles and those individuals with CWTs were identified, they were subsequently removed from the baseline and parentage assignment conducted again in order to evaluate the rate of false positive assignments.

### 2.3 Baseline

The initial baseline was outlined by Beacham et al. (2018) and consisted of 36,241 individuals genotyped at 319 SNPs from 45 populations, with the distribution of populations in southern BC. The baseline has been subsequently expanded to include 105,722 individuals genotyped at 391 SNPs from 380 populations, ranging from Russia, the Yukon River drainage, southeast Alaska, BC, the Pacific Northwest, and California. Populations included in the baseline are outlined in Table S1, with the populations from BC arranged by CU, and with Russian and United States of America (US) populations arranged by geographic (reporting) region. The CU boundaries for southern BC are indicated in Figure S1, while those for northern BC are indicated in Figure S2. The SNPs genotyped in the expanded panel are outlined in Table S2, along with primer sequences for the amplicons and *F*_ST_ and heterozygosity estimates for the SNPs. Some SNPs were found to have duplicate positions when aligned to the Chinook reference genome; these are most likely a result of genome assembly artifacts and not true duplications (K. Christensen, Univ. of Victoria, pers. comm.). For markers with “Y” in the “Multiple” column in Table S2, the position represented reflects one of the possible locations.

### 2.4 Library preparation and genotyping

The detailed procedure for library preparation and genotyping was outlined by Beacham et al. (2018), and a summarized version provided by Beacham, Wallace, Jonsen, McIntosh, Candy, Willis, Lynch, Moore et al. (2019). The process involved loading amplified DNA from 768 individuals (up to 391 amplicons per individual) on a P1 chip v3 (chip used with the Ion Torrent Proton sequencer) with an Ion Chef (laboratory instrument used to robotically load DNA libraries on to a sequencing chip). Two chips were loaded consecutively with one run of the Ion Chef, and both chips were then subsequently loaded on to an Ion Torrent Proton sequencer. After the sequencing run was completed, amplicon sequences were aligned to the coho salmon (*O. kisutch*) genome (RefSeq assembly accession GCF_002021735.1) supplemented with short sequences containing the observed Chinook salmon SNPs not definitively located in the coho genome. Alignment and determination of SNP genotypes at the sites specified by the hotspot file within target regions were conducted with Proton software Variant Caller^®^. Genotype determination was conducted with Proton software Variant Caller^®^, and SNP genotypes at the sites specified by the hotspot file within target regions were called by Variant Caller. Genotypes at all available SNPs for each individual were assembled to provide multi‐locus genotypes that were the basic input for PBT analysis. Genotypes had to be available for at least 150 SNPs for an individual to be retained in the baseline. In a test where the DNA of the same 768 individuals was genotyped on two occasions, an average genotyping error rate of 1.14% (1839 discrepancies in 161,280 single‐locus genotype comparisons) was observed over the 319 SNPs scored (Beacham et al. 2018). The species identification SNP *OkiOts_120255*‐*113* (Starks et al. 2016) and sex identification SNP *Ots_SEXY3*‐*1* were omitted from subsequent parentage and GSI analyses, leaving 389 SNPs for subsequent analysis.

### 2.5 Heterozygosity and *F*_ST_ analysis

Expected and observed heterozygosities by locus over all baseline populations were determined with adegenet (Jombart & Ahmed, 2011). Estimation of *F*_ST_ by locus was conducted with ape (Paradis & Schliep, 2018). BWA mem 0.7.17‐r1188 (Li, 2013) was used to align amplified sequences to the reference alignments subsequently filtered using filter_sam_file.py in snp‐placer (commit 8bd5e72 ‐ https://github.com/CNuge/snp-placer), and a modified version of SNP‐placer written in R (https://github.com/erondeau/snp-placercommit: eabfc78) was used to place the markers onto the Chinook reference genome assembly GCF_002872995.1 (Christensen et al., 2018). Bedtools getfasta v2.26.0 (Quinlan & Hall, 2010) was used to extract flanking sequences before and after alignment, and manually reviewed to ensure correct position was identified. Multiple best‐alignments (MapQ = 0 in sam file) were flagged in Table S2; the majority were determined most likely to be a result of genome assembly artifacts and not true duplications, and alternate mapping locations were removed from the genome using bedtools maskfasta v2.26.0 (Quinlan & Hall, 2010) for subsequent alignments and plotting. Positional information was used to plot *F*_ST_ by marker along the genome using R (R core team, 2020).

### 2.6 Identification of individuals

Since the inception of genotyping of hatchery broodstocks in 2013, the number of hatcheries participating in the program has increased, so that in the 2019 fisheries, it was potentially possible to assign parents from 48 hatchery (Figure 1b). The same techniques as outlined by Beacham et al. (2018) for PBT and GSI analysis were used in the current study. Summarized briefly, initially PBT was used for individual assignment, and the analysis was conducted where the genotypes of individuals to be identified were matched to the genotypes of prospective parents (COLONY, Jones & Wang, 2010; Wang, 2016). COLONY was run with all broodstock sampled during a single year input as a single unit for analysis of fishery or broodstock samples, with no differentiation among populations. Five age classes (2–6 years) may occur in fishery and broodstock sampling, and thus five runs of COLONY may have been conducted for each year of fishery or broodstock sampling. The choice of utilizing COLONY over SNPPIT (Anderson, 2012) was primarily to generate both two‐parent and single‐parent assignments. Where incomplete brood sampling was obtained, accurate single‐parent PBT‐based assignments could still be made, especially for hatcheries with incomplete sampling or underperforming genotyping. COLONY was run by broodyear for two reasons. First, it allowed partitioning the brood age classes across multiple computer systems, speeding up the analysis overall. Second, binning parents into year classes allowed two‐parent assignments only to individuals that could have been conceivably been crossed, limiting occasional cross‐year assignments that we presume resulted from close relatives and/or small effective population sizes. Two‐parent assignments were accepted only when both assigned parents originated from the same population in the same year and the probability of correct assignment was ≥0.95 for the parent pair. Restrictions were placed on acceptance of single‐parent assignments. First, if a two‐parent assignment had already been made for an individual in a particular year, any subsequent single‐parent assignments in alternate years were rejected. Second, if single‐parent assignments in alternate years were observed for the same individual, the assignment with the higher probability was accepted, subject to the probability being at least 0.05 higher than the competing assignment, and having the assignment probability ≥0.95. Third, an additional constraint on the single‐parent assignment before it was accepted was that both the PBT assignment and GSI assignment corresponded to populations in the same CU. Individuals for which no prospective parents were identified in the fishery samples or hatchery broodstocks available for analysis were passed to GSI for potential assignment. Polygamous mating was assumed for the COLONY analysis. Simple pairwise comparisons between offspring and potential parents were conducted. The PBT baseline for individuals sampled in the 2018 and 2019 fishery and hatchery broodstocks included all broodstocks sampled in 2013–2016 for the 2018 fishery and broodstock samples and 2013–2017 for the 2019 fishery and broodstock samples (Table S3). The parent pair output file was the basic file used in subsequent analyses.

The second method of individual identification is GSI, in which the genetic profiles of whole populations potentially contributing to a mixed‐stock sample are used to estimate the origin of each individual in the sample (RUBIAS; Moran & Anderson, 2019). This analysis was restricted to those individuals not assigned to candidate parents via COLONY. For each sample, individuals not assigned by COLONY were then assigned with RUBIAS, with the population posterior means file the basic file used for subsequent analyses. This file contained the probability of assignment of the individual to each of the 380 populations in the baseline. Stock composition was estimated through the combination of files generated with both COLONY and RUBIAS. Individuals assigned via COLONY were assigned a probability of 1.00 of originating from the identified population, with a 0.00 probability assigned to all other populations in the baseline. This level of assignment accuracy via PBT was observed previously for Chinook salmon with a smaller panel of SNPs than employed in the current study (Beacham et al. 2018). These data were then combined with the probability of assignment for those individuals unassigned via COLONY to each of the populations in the baseline via RUBIAS. A total of 25,000 iterations was run, with the first 5000 iterations set as burn‐in. The last 5000 iterations from the Monte Carlo Markov Chain from RUBIAS were used to estimate the origin of individuals and stock composition, with the mean allocation to each population in the baseline. Standard deviations of estimated stock compositions were also determined from the last 5000 iterations from the Monte Carlo Markov Chain. This approach allowed estimation of uncertainty from sources of variance from both the sample size and the genetic assignments. Stock composition by CU or reporting group was determined by summation of allocations to all populations in the baseline that belonged to the CU or reporting group under consideration.

### 2.7 Exploitation rate

Although it was potentially possible to assign parents from 48 hatchery populations for individuals sampled in the 2019 fishery, only a portion of these populations was marked with CWTs, and escapement estimation programs were conducted for only a subset of the populations marked with CWTs. Escapement estimation programs were restricted essentially to the populations designated as PST indicator populations. Exploitation rate of Chinook salmon in BC fisheries was estimated via both CWTs and genetics. Exploitation rate for a population was defined as catch/(catch + escapement). For CWTs, the observed number of CWTs was corrected by estimated tag loss rates prior to expansions. CWT expansions can be first made from the observed recoveries of a population in a fishery sample expanded to the unsampled portion of the catch (deriving estimated number from observed number), and subsequently expanded to the unmarked portion of the release (deriving expanded from estimated). For example, if 20 Robertson Creek tags were recovered in a sample of 1000 individuals and the fishery catch was 5000 Chinook salmon, then the estimated number of Robertson Creek tags that should have been observed had the entire fishery been sampled was 20 × (5000/1000) = 100 CWTs. In order to estimate the total contribution of Robertson Creek hatchery‐origin Chinook salmon to the fishery, the estimated number of tags is further expanded by the proportion of juveniles that had been marked with CWTs in a year. For example, if 10% of the Robertson Creek juveniles had been tagged prior to release from the hatchery, then the estimated number of tags (100) is expanded by the marking rate (10%) to indicate that Robertson Creek accounted for 100 × 10 = 1000 individuals in the 5000 Chinook salmon caught in the fishery. There can be an adjustment to the observed (adjusted observed) to ensure that the total expanded number is not greater than the total released number. For determining CWT exploitation rates in our study, the estimated numbers of CWTs were used in both catch and escapement to avoid adding uncertainty of the additional expansion to the unmarked release number and the uncertainty around that estimate.

For genetics, the monthly catch in a fishery was multiplied by a monthly stock composition estimate in order to estimate population‐specific catch. Catch for a population was summed over all fisheries, and age‐specific catch was estimated for those populations where at least 20 PBT identifications were made by apportioning the total population catch by the age ratios in the PBT identifications for the population. As the Capilano hatchery sourced its broodstock from the Chilliwack hatchery and thus the two hatcheries had the same genetic population, catch estimates for the Capilano population and the Chilliwack population were summed and apportioned to the respective populations by the ratio of PBT identifications for each population in the fishery. As there were 303 fishery PBT identifications for Chilliwack River and 131 identifications for Capilano River, the overall combined catch for the Capilano River and Chilliwack River populations was apportioned 69.4% to Chilliwack and 30.6% to Capilano. Broodstock sampling for PBT analysis was not conducted at the Little Qualicum River hatchery. Catch estimates for the Little Qualicum River and Big Qualicum River populations, both similar genetically, were summed and apportioned to the respective populations by the ratio of smolt releases for the 2014, 2015, 2016, and 2017 broodstocks (mean release 2,236,140 smolts, 39.7% to Little Qualicum and mean release 3,403,467 smolts, 60.3% to Big Qualicum). The escapement for populations was generally estimated either by means of a counting fence or mark–recapture studies. Age composition of the escapement additional to the hatchery broodstock was determined via a combination of CWT recoveries and scales, as genotyping of the escapement was restricted to the hatchery broodstock.

## 3 RESULTS

### 3.1 Accuracy of estimation of age and population of origin via PBT

**FIGURE 2:**
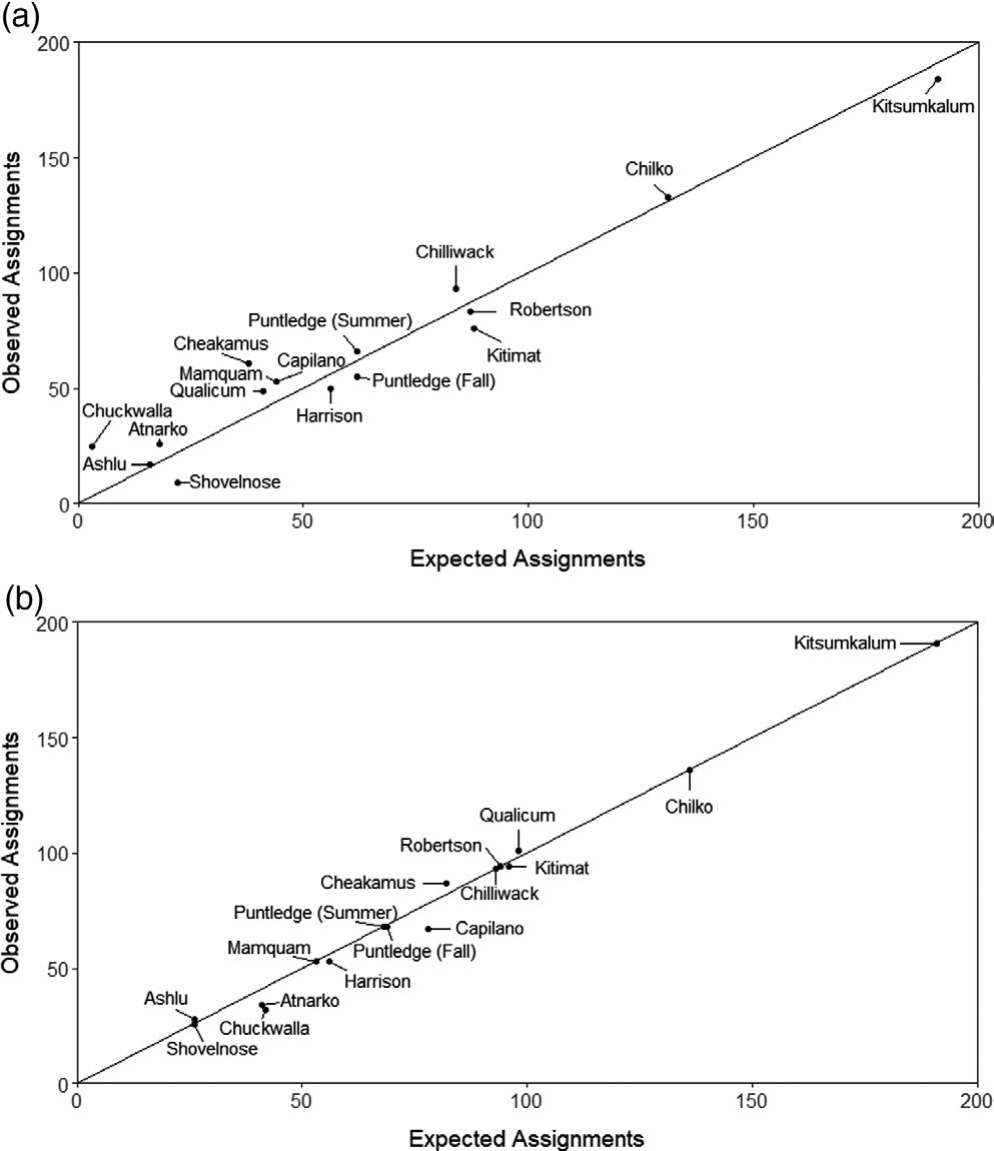
Observed versus expected number of PBT identifications for 2017 juveniles from 16 populations of Chinook salmon, with expected number of identifications based on sample size and population genetic tagging rates. (a) Two‐parent assignments. (b) One‐parent assignments

The initial test of accuracy of age and population of origin via PBT was derived from genotyping 1333 juveniles from the 2016 hatchery broodstock production from 16 populations. No assignments were obtained for 8.1% of the juveniles genotyped, with failure of assignments dominated by the Chuckwalla River population, where only 38.6% (32/83) of the juveniles were assigned (Table 1). However, only 29.5% (5/17) of potential parents were successfully genotyped from the 2016 broodstock (Table S3), accounting for the high failure rate of assignment for the juveniles from this population. The observed number of PBT identifications corresponded closely with the expected number for both two‐parent (Figure 2a) and one‐parent assignments (Figure 2b) based upon genetic tagging rate in the 16 populations, illustrating that PBT identifications were made at expected rates in mixed‐origin samples. Two‐parent assignments were obtained from 77.5% (1033 juveniles) of the juveniles genotyped, and all assignments were 100% accurate with respect to population of origin and age. Single‐parent assignments were obtained from 14.4% (192 juveniles), and 88.9% of assignments were accurate to population of origin and 100% to age. Single‐parent misassignments were observed between Big Qualicum River and Puntledge River fall populations, and between Chilliwack River and Capilano River populations. Big Qualicum River production has been previously transferred to the Puntledge River, and broodstock for the Capilano River hatchery was obtained from the Chilliwack River hatchery, potentially accounting for the single‐parent population misassignments. Once the parents had been identified, they were removed from the baseline and the parentage analysis conducted again in order to evaluate the level of false positive assignment. No parental assignments were subsequently made, providing a 0% false positive rate in this test.

**TABLE 1:** Accuracy of population assignment and age determination (%) for age 1‐year samples of juvenile Chinook salmon sampled from 16 populations in British Columbia prior to hatchery releases during 2017, with individuals assigned via parentage‐based tagging

Population	*N*	Not assigned	2‐parent	2‐parent assignment	1‐parent	1‐parent assignment	2‐parent % accuracy population	1‐parent % accuracy population
Kitsumkalum	191	0	184	Kitsumkalum	7	Kitsumkalum	100.0	100.0
Kitimat	96	2	76	Kitimat	18	Kitimat	100.0	100.0
Chuckwalla	83	51	25	Chuckwalla	7	Chuckwalla	100.0	100.0
Atnarko	48	14	26	Atnarko	8	Atnarko	100.0	100.0
Robertson	94	0	83	Robertson	11	Robertson	100.0	100.0
Puntledge (Summer)	68	0	66	Puntledge (Summer)	2	Puntledge (Summer)	100.0	100.0
Puntledge (Fall)	69	1	55	Puntledge (Fall)	13	Puntledge (Fall)	100.0	100.0
Qualicum	119	18	49	Qualicum	44	Qualicum	100.0	84.6
					8	Puntledge		
Ashlu	28	0	17	Ashlu	11	Ashlu	100.0	100.0
Mamquam	53	0	53	Mamquam	0	–	100.0	–
Cheakamus	95	8	61	Cheakamus	26	Cheakamus	100.0	100.0
Shovelnose	26	0	9	Shovelnose	17	Shovelnose	100.0	100.0
Capilano	78	11	53	Capilano	10	Capilano	100.0	71.4
					4	Chilliwack		
Chilko	136	0	133	Chilko	3	Chilko	100.0	100.0
Chilliwack	93	0	93	Chilliwack	0	–	100.0	–
Harrison	56	3	50	Harrison	3	Harrison	100.0	100.0
Total	1333	108	1033		192		100.0	88.9

Note

The baseline available for potential parentage assignment included hatchery broodstocks genotyped prior to 2017. *N* is number of juveniles genotyped for each population, as well as the number not subsequently assigned via PBT, and those assigned via 2 and 1 parents

The second test of accuracy of population of origin and age was conducted with coded‐wire tagged individuals from 2017 hatchery broodstocks or escapements from five populations, with CWTs allowing for a broader range of age variation than with the juveniles tested previously. Genotypes and assignments were obtained from 769 individuals that also were marked with a CWT. Two‐parent assignments were 100% accurate with respect to population of origin, but approximately 99.4% accurate with respect to age (Table 2). Two Quinsam River individuals identified as age 4 years via CWTs were assigned to parents in the 2014 broodyear, identifying them as age 3 years. Conversely, in the Puntledge River fall population, two individuals identified as age 3 years via CWTs were assigned to parents in the 2013 broodyear, identifying them as age 4 years. All single‐parent assignments (87) were 100% accurate with respect to population of origin and age. Under the assumption that the CWTs were decoded accurately, of the 769 assignments, 100% were accurate with respect to population of origin, and 99.5% were accurate with respect to age (Table 2). Once the parents had been identified, they were removed from the baseline and the parentage analysis conducted again in order to evaluate the level of false positive assignment. Like the juveniles analyzed previously, no parental assignments were made, providing a 0% false positive rate.

**TABLE 2:** Accuracy of population assignment and age (year) determination (%) for coded‐wire tagged Chinook salmon sampled from 2017 hatchery broodstocks or escapements for five populations in British Columbia

Population	CWT age	*N* CWT	Two‐parent PBT age			One‐parent PBT age			% Accuracy age determination
			2	3	4	2	3	4	
Quinsam	3	26		26					100.0
	4	365		2	319			44	99.5
Puntledge summer	2	1	1						100.0
	3	30		29			1		100.0
	4	10			10				100.0
Puntledge fall	2	7	7						100.0
	3	91		70	2		19		97.8
	4	20			16			4	100.0
Qualicum	2	18	14			4			100.0
	3	82		72			10		100.0
	4	23			18			5	100.0
Robertson	2	2	2						100.0
	3	81		73			8		100.0
	4	13			13				100.0
Total	2	28	24			4			100.0
	3	310		270	2		38		99.4
	4	431		2	376			53	99.5

Note

*N* CWT is the number of individuals containing a CWT for a specific age. Assignment to population of origin for the individuals with a CWT was 100% accurate via PBT.

In the current study, no errors were made in assigning individuals to population of origin via two‐parent PBT assignments (1707 assignments), but 4.3% (287 assignments) of single‐parent assignments were incorrect to population. The assignments errors were confined to some (4/14) single‐parent assignments of Capilano River hatchery fry identified as originating from the Chilliwack River hatchery broodstock, and some (8/52) Big Qualicum River fry as originating from the fall Puntledge River hatchery broodstock. The Capilano River hatchery sources its broodstock from the Chilliwack River hatchery, so essentially errors in identification were made to individual parents from the same population. Chinook salmon from the Big Qualicum River have been transferred to the Puntledge River fall population previously, and genotyping success rate of the 2016 Big Qualicum River broodstock (63%, Table S3) likely accounted for the assignment errors. Age of individuals identified through PBT in the current study was 99.8% accurate (1990/1994).

### 3.2 GSI applied to 2018 fishery sampling

Fishery samples were obtained from 12 fisheries in 2018 and stock compositions were estimated for the samples (Table S4). Based upon the geographic locations and fishery timing, inferences can be drawn as to the reliability of the estimated stock compositions in actual fisheries application, with a fuller description of the results outlined in Supplementary Results 2.3. In total, 6.286 individuals were genotyped from 12 fisheries, and estimated stock compositions corresponded to expectations based upon the locations of the fisheries.

### 3.3 GSI applied to 2019 fishery sampling

In 2019, sampling was conducted for 17 fisheries in BC, with 12.533 Chinook salmon genotyped from the fishery sampling. A more detailed description of the GSI results available from the fishery sampling was outlined in Supplementary Results 2.4. In general, estimated stock compositions were consistent with expectations based upon geographic locations of the fisheries. Fisheries in more terminal locations were estimated to have been comprised almost entirely of Chinook salmon originating from a single CU (Table S5).

### 3.4 Strait of Georgia legal versus sublegal catch

**FIGURE 3:**
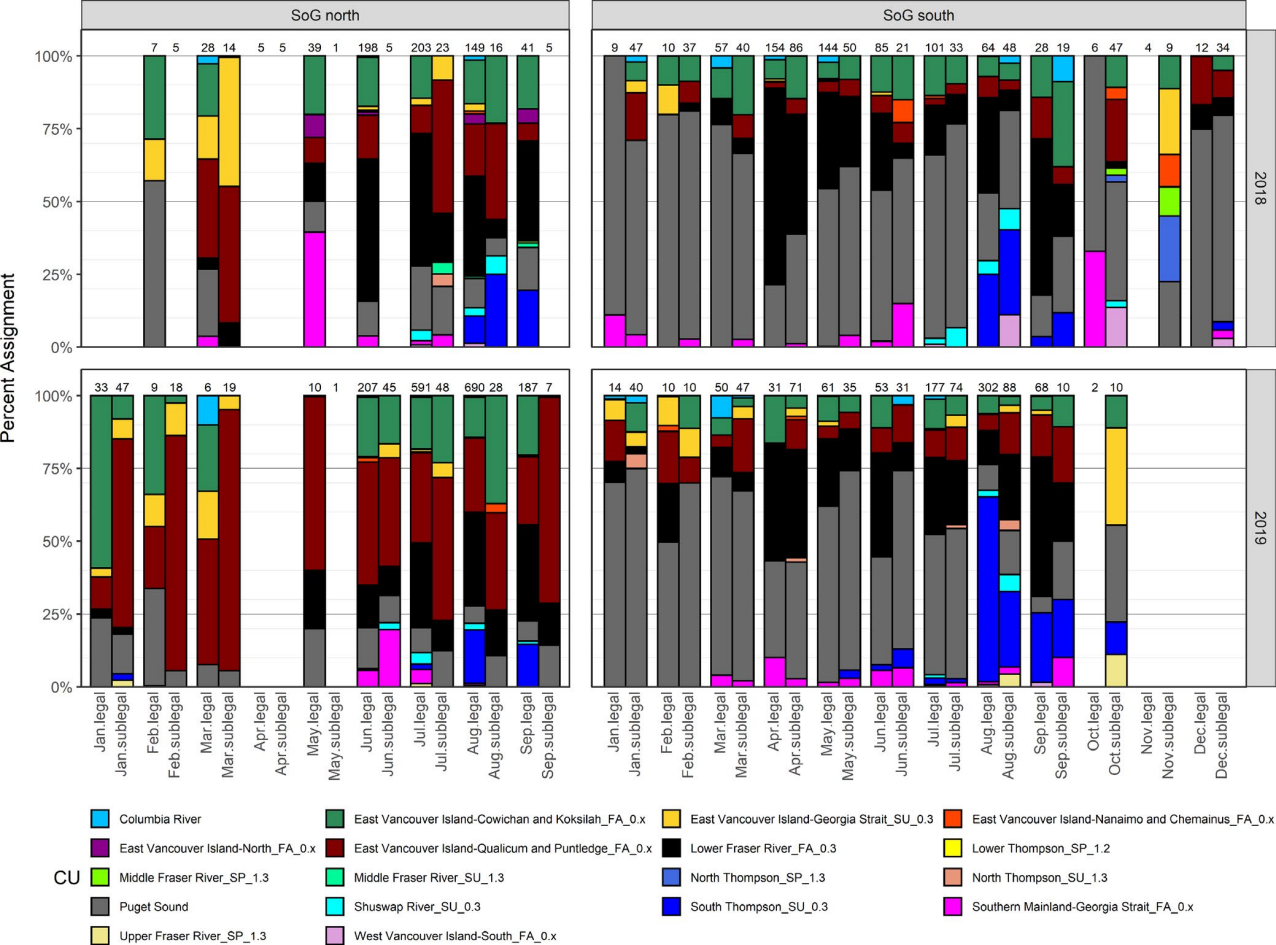
Estimated stock compositions (%) for legal (fork length >62 cm) and sublegal (fork length < 62 cm) Chinook salmon caught in the Strait of Georgia (SoG) north and south recreational fisheries, 2018 and 2019. Values above bars are number of individuals genotyped for each size class by month

In the recreational fishery in the SoG in 2018 and 2019, individual Chinook salmon had to exceed 62 cm in length in order to be retained (legal), while those individuals shorter than this limit were released (sublegal). In 2018, substantial differences in stock composition were observed between legal‐sized and sublegal‐sized catches in the same month in the northern SoG fishery (Figure 3). For example, the ECVI‐Qualicum and Puntledge fall age 0.x CU comprised 6.2%–17.8% of the legal‐sized catch between July and September, while the CU comprised between 33.0% and 59.7% of the sublegal‐sized catch for the same months. Conversely, Chinook salmon from the lower Fraser River fall age 0.3 CU comprised between 34.1% and 47.5% of the legal‐sized catch between June and September, while the CU comprised 4.8%–20.0% of the sublegal‐sized catch for the same months. Chinook salmon originating from different CUs were present in markedly different proportions above and below the 62‐cm legal size limit in the northern SoG fishery. In 2019, substantial differences in stock composition between legal and sublegal catches in the same month in the SoG north recreational fishery were observed, similar to those in 2018 (Figure 3).

In 2018, there were substantial differences in stock composition between legal and sublegal catches in the same month in the southern SoG recreational fishery. For example, stock compositions of the Cowichan and Koksilah fall age 0.x CU tended to be higher in the sublegal catch than in the legal catch in most months (Figure 3). Chinook salmon from Puget Sound contributed 30.8%–63.6% of the sublegal catch from April through August, and continued to be a significant contributor to the sublegal catch for the duration of the year, with similar observations in 2019.

### 3.5 PBT applied to fishery sampling

In 2018, 889 PBT identifications were made in the 6286 individuals genotyped (14.1% identification rate), and the identifications ranged over 27 populations (Table S6). The Robertson Creek population dominated in the PBT identifications, comprising 51.6% of all identifications, and there was a wide geographic range in their fishery identifications, other than in the SoG. Fishery PBT identifications for the Quinsam River population were similarly geographically wide ranging, but were also observed in the SoG. Fishery PBT identifications for the Chilliwack River population (17.1% of all identifications) were more restricted geographically, largely in fisheries in Johnstone Strait, the SoG, and JDF. Fishery PBT identifications of the Capilano River broodstock (10.3%) were largely restricted to the SoG. PBT provided the first known occurrence of identification of individuals originating from the non‐CWT populations in Canadian fisheries.

As broodstock genotyping for some selected populations commenced in 2013, 2019 marked the first year in which PBT could be applied to fishery sampling with the expectation that PBT identifications for those populations previously genotyped were potentially available for most ages comprising the samples (2–6 years). In 2019, 2702 PBT identifications were made in the 12,533 individuals genotyped from fisheries (21.6% identification rate), and the identifications ranged over 31 populations (Table S7). The Robertson Creek population again dominated the fishery PBT identifications, accounting for 46.8% (1264/2702) of the identifications, with again with Robertson Creek individuals identified in virtually all fisheries outside of the SoG. As in 2018, fishery PBT identifications for the Chilliwack River population (12.4%) were largely restricted to fisheries in Johnstone Strait, the SoG, and JDF, similar to the fishery PBT identifications of the Capilano River broodstock (5.0%). Notably, two fishery PBT identifications were made for the Nicola River population, a population of conservation concern and one in which virtually all of the hatchery production is currently marked with CWTs. One PBT identification was observed in July samples from the recreational fishery in the northern SoG, and one identification in July samples from the southern SoG (Table S7).

### 3.6 Fishery age composition derived from CWTs and PBT

**FIGURE 4:**
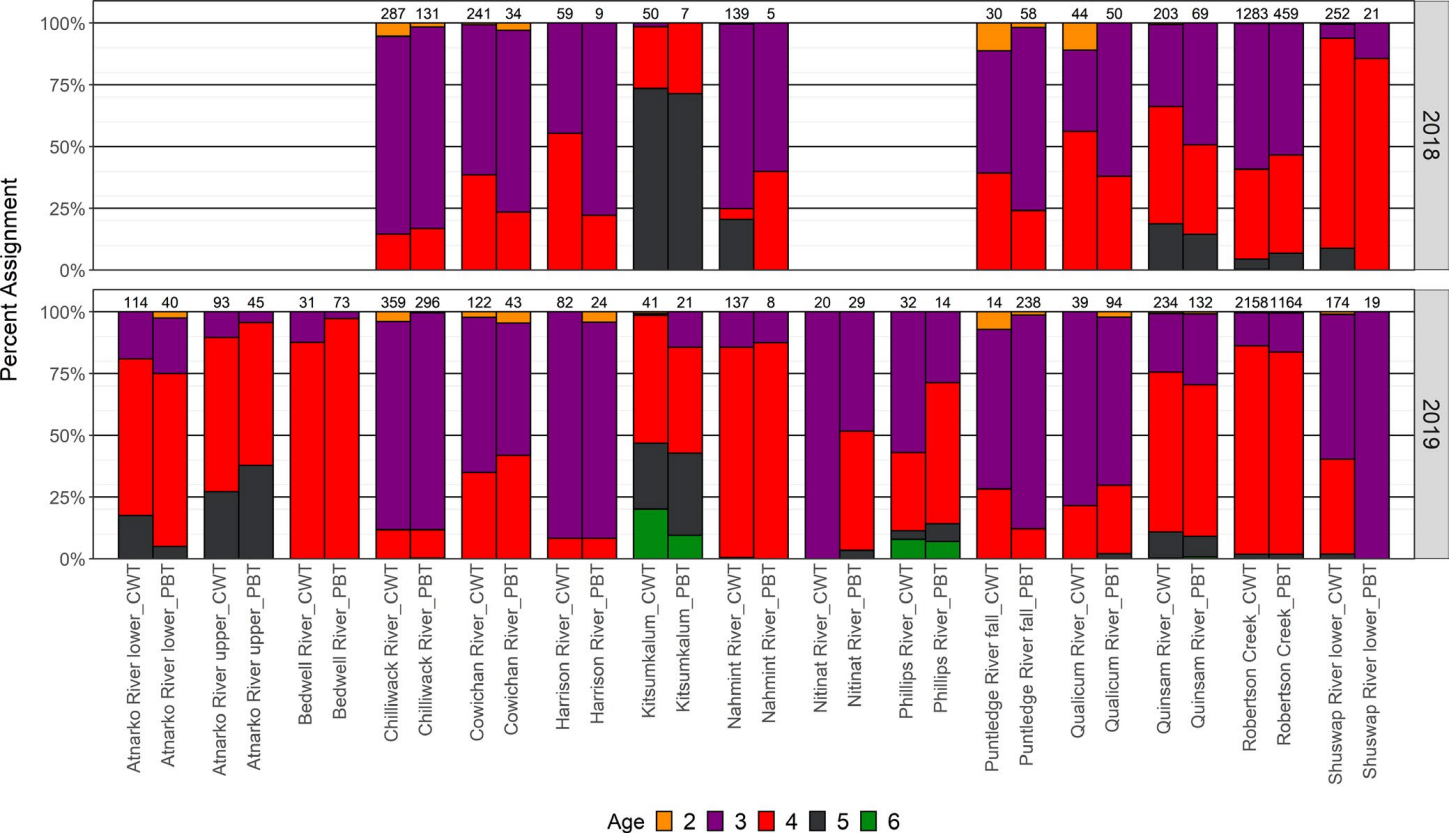
Comparisons in age composition between CWTs and parentage‐based tagging (PBT) age compositions for Chinook salmon populations in 2018 and 2019 fishery sampling. Age composition derived from CWTs was obtained after expansions for tagging rate and fishery sampling rate. Observed number of CWTs recovered from fisheries and the number of PBT identifications are indicated at the top of each bar. At least five CWTs and five PBT identifications had to be observed for the population in a year before inclusion in the figure

With 3591 PBT identifications available from fishery sampling in 2018 and 2019, there was an opportunity to compare fishery‐derived age compositions obtained from CWTs and PBT. With comparisons restricted to those populations with a minimum of five CWT and five PBT identifications in a year, there was in general good agreement in age compositions across a range of populations (Figure 4, Table S8). For example, there was no significant difference in age composition derived from the two methods for the Robertson Creek population in either 2018 ($\chi_{\left(4\right)}^{2}$ = 6.01, *p* > 0.19) or 2019 ($\chi_{\left(3\right)}^{2}$ = 4.13, *p* > 0.24), for the Quinsam River population in either 2018 ($\chi_{\left(3\right)}^{2}$ = 5.93, *p* > 0.11) or 2019 ($\chi_{\left(4\right)}^{2}$=1.72, *p* > 0.78), or for the Cowichan River population in 2018 ($\chi_{\left(2\right)}^{2}$=3.86, *p* > 0.14) or 2019 ($\chi_{\left(2\right)}^{2}$ = 1.36, *p* > 0.50). CWT marking for the Nitinat River population only started in 2016, so there was no opportunity for recovery of CWTs from older‐aged individuals that were observed with PBT. Fishery‐derived age compositions for a population in a year were similar between CWTs and PBT.

Legal‐sized individuals were primarily age 3 (83%) and 4 (16%) years, whereas sublegal individuals were primarily age 2 (68%) and 3 (32%) years, with little difference in the age composition in the fishery in the northern and southern regions of the SoG (Table 3). Age composition of the sublegal catch could only be obtained via PBT, as the sublegal individuals had to be released, and thus it was not possible to determine age via CWTs.

**TABLE 3:** Age distribution (%) of PBT identified Chinook salmon above 62 cm fork length (Legal) or below (Sublegal) in recreational fisheries in the Strait of Georgia in 2018 and 2019. Sample size is in parenthesis

Age	Strait of Georgia north				Strait of Georgia south				Strait of Georgia combined			
	*N*	Legal	*N*	Sublegal	*N*	Legal	*N*	Sublegal	*N*	Legal	*N*	Sublegal
2	3	0.5	67	67.0	3	1.3	80	68.4	6	0.7	147	67.7
3	511	84.0	33	33.0	180	80.7	36	30.8	691	83.1	69	31.8
4	101	15.2			40	17.9	1	0.8	141	16.0	1	0.5
5	2	0.3							2	0.2		
Mean	617	3.17	100	2.33	223	3.17	117	2.32	840	3.17	217	2.33

### 3.7 GSI applied in combination with PBT to fishery sampling

When PBT and GSI are applied in combination, it is possible to evaluate fishery samples for both age and stock composition. Sampling in the recreational fishery in the SoG indicated that Chinook salmon are present year round in the SoG, and that substantial differences in stock composition were observed in the northern and southern portions of the SoG. For example, in the northern SoG, winter residents were primarily sublegal‐sized individuals from the Puntledge River, Qualicum River, and Cowichan River populations, whereas those in the southern SoG were primarily from Puget Sound populations (Table 4, Figure 4). Individuals from the lower Fraser fall age 0.3 CU were relatively more abundant in the southern SoG and arrived in April, with legal‐sized individuals relatively more abundant than sublegal individuals. Chinook salmon from the summer South Thompson age 0.3 CU were more prevalent in the southern SoG and arrived in August and September. Seasonal, regional, size‐related variability stock compositions were observed in the recreational fishery in the SoG.

**TABLE 4:** Observed number of PBT identifications by month and age in legal (Leg; >62 cm fork length) and sublegal (Sub; <62 cm) fishery sampling for Puntledge River fall, Big Qualicum River, Capilano River, and Chilliwack River fall populations in the northern and southern recreational fishery in the Strait of Georgia (SoG) 2018–2019

Age	January		February		March		April		May		June		July		August		September	
	Leg	Sub	Leg	Sub	Leg	Sub	Leg	Sub	Leg	Sub	Leg	Sub	Leg	Sub	Leg	Sub	Leg	Sub
	SoG north Puntledge fall																	
2		11		5		3						7	1	5		2		1
3		2			1	5	1		30	4	50		48		20			
4									1		6		7		2			
	SoG north Big Qualicum																	
2		5		2		1				1		5	1	1		3		
3	1				2	1		1	4		19	1	22	2	3	2		
4	1								3		12		3		2			
	SoG south Puntledge fall																	
2		2				1								3		4	1	
3	1						1		1		2		3		5		1	
4																	1	
	SoG south Big Qualicum																	
2		2				4		1				1		2		4		1
3		1		1	1		2			1	2	1			2		1	
4									1				1				1	
	SoG south Capilano																	
2				1				3	1	1								1
3		1	1		8	1	2	4	2	1	7		13		5			
4			1								3				6			
	SoG south Chilliwack																	
2								20		3		2		11		4		1
3					18	1	8	9	14	2	12		23		14	1	10	
4			1		6		1		1		3		3		0		1	

### 3.8 Estimation of catch by CU

The application of GSI and PBT enabled estimation of regional and CU contributions of Chinook salmon to the 2019 marine catch in BC. Approximately 396,000 Chinook salmon were estimated to have been caught in marine fisheries in BC, with approximately 6% originating from CUs in northern and central BC, 1% from the southern BC mainland CUs, 17% from Fraser River CUs, 8% from ECVI CUs, 39% from WCVI CUs, and 29% from the western US (Table 5). In the Fraser River drainage, the South Thompson River summer age 0.3 CU was by far the dominant contributor to the BC catch (67% of drainage contribution, 11.6% of BC total catch), followed by the Lower Fraser fall age 0.3 CU (21% of drainage contribution, 3.6% of BC total catch). For the ECVI region, the Qualicum River‐Puntledge River fall age 0.3 CU was the dominant contributor (63% of regional contribution, 4.6% of BC total catch) followed by the Cowichan River‐Koksilah River fall age 0.x CU (33% of regional contribution, 2.4% of BC total catch). For the WCVI region, the WCVI south fall age x.3 CU was by far the dominant contributor (79% of regional contribution, 30.8% of BC total catch). The Robertson Creek population in this CU was estimated by itself to have contributed 24.0% of the total BC catch. The Nootka Sound‐Kyuquot Sound fall age x.3 CU contributed 21% to the WCVI regional total and 8.0% to the BC catch, with the Conuma River population itself contributing 6.5% of the total BC catch. In 2019, Chinook salmon from Puget Sound contributed a minimum of 6.7% to the total BC catch, as did Columbia River‐origin Chinook salmon (10.4%), Oregon‐origin (4.6%), and California‐origin (0.8%).

**TABLE 5:** Catch of Chinook salmon by conservation unit (CU) or region for fisheries in BC during 2019 with catch derived from GSI‐PBT for 13 fisheries in BC

CU/Region	1	2	3	4	5	6	7	8	9	10	11	12	13
SEAK	13	3											16
Alsek	47												47
Unuk		286											286
Taku early					3								3
Taku mid		2											2
Taku late					1								1
Stikine early		21			1								22
Stikine late		32			5								37
Haida north	103	97											200
Nass upper		1397		51	40				1				1489
Nass lower		1319	17	1	3				6				1346
Ecstall		495											495
Sk‐est		516											516
Skeena lower		317	13		25								355
Kalum early		23											23
Kalum late		3019							8				3027
Zymoetz		446											446
Sicintine													0
Skeena tribs		1293			17								1310
Skeena lakes		697	53		1								751
Skeena upper		618			9								627
Bulkley		20											20
Central lake	205	503	3		194								905
Central stream		549	27	66	1								643
Rivers		315	141		75								531
Wannock		1282	216		13								1511
Bella		1652	395	4936	80				95				7158
Dean		104	69	285	40								498
Docee	103	225											328
Klina		520	79	42	98	751	11		18				1519
SM‐fjords		134	86	18	67	21							326
SM‐Georgia		113	172	1	45	789	96						1216
UFR Sp 1.3		116				125			146				387
MFR Su 1.3	74	105				223	42	242	102			117	905
MFR Sp 1.3	7	81				39	57	13	53				250
Portage 1.3	94												94
NTho Sp 1.3			1			113	30						144
NTho Su 1.3		111	10		13	39		72	132			117	494
Shus Su 0.3		1414	27	18	180	1006	219	830	832			75	4601
Bess Su 1.2		17											17
STho Su 0.3	4370	9554	774		1230	565	6566	7948	8618	3025		3319	45,969
STho Su 1.3		1		70		21			48				140
LTho Sp 1.2						112	6						118
LFra Sp 1.3	103												103
LFra Su 1.3		97			10								107
Pitt Su 1.3			17	18	81	459	31		10				616
Maria 0.3									16				16
LFra Fa 0.3		282	310	17	151	5218	2884	1932	1156	2018		234	14,202
ECVI N 0.x		1624	958	213	562	235	141		303				4036
Qual‐Punt	414	564	1540	121	779	12,291	1089	541	679			5	18,023
Nan‐Chem F		9				24	10		4				47
Nan Sp 1.x													0
ECVI Su 0.3					14	969	55		11				1049
Cow‐Kok	46	103	32	18	101	5941	842	836	607	672		345	9543
Nootka 0.x	20	2057	268	184	2768		6		11,902		14,099	245	31,549
WCVI N 0.x				53					656				709
WCVI S 0.x	3243	12,439	1586	319	3389		110	3894	36,203	337	57,800	2684	122,004
Okanagan 1.x	35	84	1		2			2	134				258
JDF		161			58			167	304				690
Wash coastal	8788	3300	14		42			132	533	840		573	14,222^a^
NPuget S	47	326	29	84	263	769	329	2792	3583	1312		127	9.661^a^
SPuget S		280	303	70	405	2054	1192	5947	4319	3731		2451	20,752^a^
Col lower	1337	744	43		55	21		280	1778	2026		1524	7808^a^
Col mid Sp		42	51	4	39		19	168	960	864			2147^a^
Col up Sp													0
Col up Su Fa	10,896	6170	265		217	30		30	1714	3741		626	23,689^a^
Snake Fa	2646	1254	58	17	57	12		20	880	694		795	6433^a^
Snake Sp Su	149												149^a^
N C Oregon	6599	1689							348	1221		732	10,589^a^
Willa upper	195	299							82	70			646^a^
S Oregon	3267	1416							630	796		903	7012^a^
Klam‐Trin									42				42^a^
CCV Fa		20		16	3			70	428	1237		457	2231^a^
CCV Sp				1	7			12	207	611		123	961^a^
Cal coast													0
Total	42,801	60,357	7558	6623	11,144	31,827	13,735	25,933	85,218^a^	23,195	71,899	15,452	395,742

Note

CU catch was estimated as (monthly catch) * (population‐specific monthly stock composition) summed over all populations in the CU in the baseline. Fisheries were as follows: 1) northern troll, 2) northern sport, 3) central sport, 4) central net and First Nations food, social, and ceremonial (FSC) fishery, 5) Johnstone Strait sport, 6) Strait of Georgia‐north sport, 7) Strait of Georgia‐south sport, 8) Juan de Fuca Strait sport, 9) west coast Vancouver Island sport, 10) WCVI troll, 11) WCVI gillnet and seine, 12) First Nation WCVI troll, and 13) all fisheries.

^a^ Includes an estimated 9% (7680 Chinook salmon) of WCVI sport catch which were adipose fin clipped but not otolith marked and which were assumed to be of US hatchery origin. Stock composition was not available for these estimated 7680 Chinook salmon and has not been included in individual US geographic regions.

### 3.9 Assessment of hatchery broodstocks

Genotyping of selected hatchery broodstocks began in 2013, with partial genotyping of nine populations and full genotyping of 17 populations (Table S3). The number of hatchery broodstocks that were genotyped increased over time. By 2018, 42 hatchery populations were sampled, and 14,530 of 14,949 individuals were successfully genotyped (97.2% success rate; Table S3). Of these 42 populations, there were 19 hatchery broodstocks for which it was possible to investigate origins of individuals in the 2018 broodstocks via PBT identification, as full or nearly full genotyping of hatchery broodstocks had been conducted annually since 2013. Of these 19 populations, 12,392 of 12,855 individuals were successfully genotyped (96.4% success rate; Table S9). Five large hatchery broodstocks (Robertson Creek, Quinsam River, Big Qualicum River, Chilliwack River (Chilliwack and Capilano broodstocks), and Atnarko River) accounted for 75.5% of the total number of broodstock individuals genotyped for these 19 populations. Overall, approximately 41% of individuals genotyped (5024 fish) from these 19 populations were assigned to hatchery parents from 2013 to 2016. The PBT assignment rate varied considerably among populations, ranging from 0% at Harrison River where the broodstock was obtained by seining in the river to 89% for the Puntledge River summer broodstock where the hatchery consistently produces a substantial portion of the returning adults.

In 2019, the hatchery broodstock sampling program was expanded and 53 hatchery populations were sampled, with 19,722 of 19,938 individuals successfully genotyped (98.9% success rate; Table S3). Of these 53 populations, 23 hatchery broodstocks were analyzed via PBT, with 16,092 of 16,254 Chinook salmon successfully genotyped (99.0% success rate) for these broodstocks (Table S10). As in 2018, no PBT assignments were observed in the Harrison River broodstock as the population is largely naturally spawned, but substantial assignments (73%) observed in the summer Puntledge River broodstock, a population which is largely hatchery origin.

Hatchery‐origin jacks or jills (age 2 spawners) comprised an average 0.4% of hatchery broodstocks in both 2018 and 2019, with the highest values observed for the Sarita River population (6.2% 2018, 2.7% 2019), and with many populations having no jacks observed in the broodstock (Tables S9,S10). Strays identified in sampled populations identified via PBT were incorporated into broodstocks at an average rate of 0.4% in both 2018 and 2019, with virtually all straying occurring between geographically proximate populations. In the Puntledge River, although not defined as strays, offspring from previously defined fall‐returning parents were incorporated into summer‐return broodstocks at a rate of 4.2% in 2018 (12 of 286 individuals) and 4.7% in 2019 (7 of 150 individuals). Conversely, offspring from previously defined summer‐returning parents were incorporated into the fall‐return broodstock at a rate of 0.9% in 2018 (7 of 815 individuals) and 1.3% in 2019 (13 of 968 individuals).

### 3.10 Estimation of exploitation rate (ER)

**FIGURE 5:**
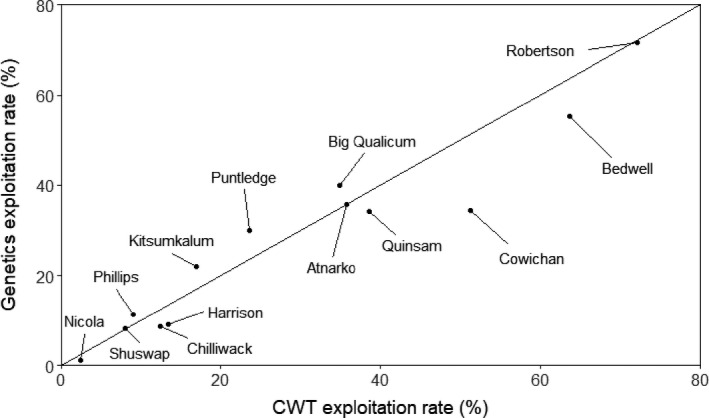
Exploitation rate (%) in 2019 Canadian marine fisheries for 13 populations estimated via CWTs and via GSI‐PBT. Exploitation rate for a population is defined as catch/(catch + escapement). The line y=x is illustrated

One of the main assessment requirements for fishery management is estimation of ER. Importantly, either CWTs or genetics can be applied to estimate population‐specific ER. There was a wide range in observed population ERs in Canadian marine fisheries, ranging from the low exploitation rate population Nicola River (2.4% CWT, 1.2% genetics) to the higher rate population Robertson Creek (72.2% CWT, 71.7% genetics; Table 6). For the 13 populations evaluated, there was generally a close agreement between 2019 Canadian marine fishery ERs estimated via CWTs and genetics (*r*_(11)_ = 0.960, *p* < 0.01; Figure 5). The greatest discrepancy in ER between the two methods (16.7%) was observed in the Cowichan River population, which was due to the under estimation of CWTs in the escapement sampling (see Section 4.2). With the Cowichan River population removed from the analysis, there was very close alignment between ERs estimated through CWTs and genetics for the remaining 12 populations (*r*_(10)_ = 0.979, *p* < 0.01).

**TABLE 6:** Exploitation rates (%) via CWTs and genetic stock identification (GSI) with parentage‐based tagging (PBT) for specific populations in 2019 Canadian marine fisheries

Population	Age	CWT			GSI and PBT		
		Catch	Escapement	ER	Catch	Escapement	ER
Kitsumkalum	3	1.0	0.0	100.0	99	0.0	100.0
	4	77.1	426.7	15.3	896	989	47.5
	5	46.1	234.8	16.4	697	4945	12.4
	6	28.4	88.2	24.4	199	690	22.4
	Total	152.6	749.7	16.9	1891	6673^a^	22.1
Atnarko	2				94	70	57.3
	3	114.3	265.8	30.1	969	2347	29.2
	4	483.0	852.2	36.2	4208	6958	37.7
	5	148.8	224.6	39.9	1211	2267	34.8
	6				0	23	0.0
	Total	746.1	1342.6	35.7	6482	11,675	35.7
Phillips	2				0	31	0.0
	3	68.9	631.5	9.8	94	1201	7.3
	4	36.5	535.0	6.4	186	1127	14.2
	5	4.3	24.6	14.8	23	172	11.8
	6	9.8	0.0	100.0	23	0	100.0
	Total	119.5	1191.1	9.1	326	2531	11.4
Shuswap	Total	454.5	5160.2	8.1	4165	45,187	8.4
Nicola	Total	29.4	1199.6	2.4	45	3858	1.2
Harrison	2		75.6	0.0	199	1093	15.4
	3	269.4	1028.7	20.8	4143	19,340	17.6
	4	23.8	779.9	3.0	393	26,668	1.5
	5		10.8	0.0	0	431	0.0
	Total	293.2	1895.0	13.4	4735	47,532	9.1
Chilliwack	2	39.1	843.1	4.4	27	3739	0.7
	3	1113.6	7046.7	13.6	5882	58,923	9.1
	4	124.7	1137.3	9.9	770	11,290	6.4
	5	0.0	0.0		20	85	19.0
	Total	1277.4	9027.2	12.4	6699	74,037	8.3
Quinsam	2	7.8	44.9	14.9	28	119	19.0
	3	236.1	495.0	32.3	995	1809	35.5
	4	716.3	1078.7	39.9	2122	4520	31.9
	5	116.8	92.6	55.8	287	322	47.1
	6	2.9	1.0	74.5	28	14	66.7
	Total	1079.9	1712.2	38.6	3460	6784	34.2
Puntledge fall	2	4.9	109.6	4.3	88	3366	2.5
	3	42.7	89.8	32.2	6356	9220	40.1
	4	22.4	26.9	45.4	895	4459	16.7
	Total	70.0	226.3	23.6	7340	17.045	30.1
Big Qualicum	2	0.0	37.0	0.0	135	2199	8.9
	3	139.0	190.2	42.2	4380	4177	51.2
	4	67.9	158.4	30.0	1782	3257	35.4
	5	0.0	1.4	0.0	135	48	73.8
	Total	206.9	387.0	34.8	6432	9681	39.9
Cowichan	2	10.9	58.4	15.7	439	3166	12.2
	3	427.9	304.0	58.5	5.104	5382	48.7
	4	182.7	229.5	44.3	3998	9466	29.7
	5	0.0	0.0		0	95	0.0
	Total	621.5	591.9	51.2	9541	18,109	34.5
Robertson	2	82.5	490.6	14.4	475	4630	9.3
	3	2616.1	1614.3	61.8	15,021	9322	61.7
	4	6955.0	1625.0	81.1	77,765	23126	77.1
	5	122.0	28.4	81.1	1806	490	78.7
	Total	9775.6	3764.3	72.2	95,067	37,568	71.7
Bedwell		158.4	90^b^	63.7	404	399	55.3

Note

Age‐specific exploitation rates were determined only for those populations in which at least 20 PBT identifications were observed in fishery sampling.

^a^ Includes 49 age 7 year Kitsumkalum, and age composition derived from scale analysis.

^b^ Hatchery proportion of escapement determined via adipose fin clip rate.

Age‐specific exploitation rates are also of interest in fisheries assessment. For the three populations in which age 6 year comparisons were possible, there was good agreement between the two assessment methods (*r*_(1)_ = 0.995, *p* < 0.01; Table 6). There was poor agreement in exploitation rates of age 5 year Chinook salmon between the two methods in seven populations (*r*_(5)_ = 0.572, *p* > 0.10), but this was largely due to the Big Qualicum River population (CWT 0.0%, genetics 73.8%). With the Big Qualicum River population removed, there was good agreement between the two techniques (*r*_(5)_ = 0.958, *p* < 0.01). Comparisons of exploitation rates of age 4 year Chinook salmon were possible in 10 populations, and reasonable agreement was observed (*r*_(8)_ = 0.765, *p* < 0.05), with the largest discrepancy observed in the Kitsumkalum River population (15.3% CWT, 47.5% genetics). With this population removed, there was reasonable agreement between the two techniques (*r*_(7)_ = 0.891, *p* < 0.01). Comparisons of exploitation rates of age 3 year Chinook salmon were possible in 10 populations, and good agreement was observed (*r*_(8)_ = 0.979, *p* < 0.01). For age 2 year (jack) salmon, CWTs were observed in the catch for five populations and in the escapement for seven populations whereas jack PBT identifications in the catch were observed in eight populations, and jacks were observed in the escapement for nine populations. This led to poor agreement in jack exploitation rates between the two methods (*r*_(7)_ = −0.095, *p* > 0.10), largely as a result of 0.0% CWT‐derived exploitation rates in three populations, whereas jack exploitation rates for those same populations ranged from 8.9% (Big Qualicum) to 57.3% (Atnarko).

## 4 DISCUSSION

The first accomplishment of the study was to demonstrate that PBT assignments were accurate with respect to identification of population of origin and individual age. The second accomplishment was to illustrate that the number of PBT identifications conformed to expectations of tag rates by delivering appropriate levels of observed assignments. The third accomplishment of the current study was to provide GSI‐derived high‐resolution stock composition estimates for 2018 and 2019 Canadian fisheries. This study marked the first time that Chinook salmon fisheries impacts in Canada could be evaluated by CU, and thus has enabled an assessment that was sufficiently informative for conservation‐based management as envisaged in the WSP. There is no other method of fishery assessment that can provide this level of resolution for mixed‐stock analysis. The fourth accomplishment was to merge a wide‐ranging PBT‐based assessment of fishery impacts with the GSI‐based assessment to evaluate stock composition and age structure of both legal‐sized and sublegal catches of Chinook salmon, illustrating that GSI and PBT can be applied in combination to provide information unavailable from CWTs. The fifth accomplishment was to sample 2019 fisheries with enough intensity for the genetic analysis to provide a realistic comparison with an existing CWT sampling program that has been in existence for many years. Merging these five accomplishments together has provided an opportunity to evaluate the finding an expert panel who predicted that PBT could provide the equivalent of CWT recovery data and could be easily integrated with a GSI program system to provide stock of origin for all fish sampled in fisheries (PSC, 2005).

### 4.1 Accuracy of estimation of stock composition

One major application of a GSI‐PBT approach to fisheries assessment is to estimate the stock composition of the catch, which can include contributions from populations that are not marked with CWTs. One major difference between CWT and GSI‐PBT fisheries applications is the inability of the CWT approach to provide estimates of stock composition of the catch from a fishery. For specific CWT‐tagged populations, CWT recoveries are used to estimate the total contribution from those populations through “expansions” of the number of recovered CWTs to account for the CWT marking rate and proportion of the catch sampled. However, no estimation of the catch contributions from untagged populations is possible, precluding the estimation of stock composition for the entire fishery sample that includes fish from tagged and untagged populations. Both CWTs and GSI‐PBT can be used to estimate the catch of the hatchery component of a population (possibly even a wild index population) for later application in estimation of fishery exploitation rate, but only the GSI‐PBT approach can provide reliable estimates of stock composition of CUs and populations within the CU in the fishery sample.

Both PBT and GSI can be used to identify individuals to specific populations, but only PBT can determine the age of the individual, as once the parents are identified in the hatchery broodstock, age of the individual is easily determined by calculating the difference between the year of broodstock sampling and fishery sampling. When the accuracy of identification of population of origin and age were combined with the levels of accuracy previously reported by Beacham et al. (2018) for population and age identification, we concluded that the level of accuracy for both population and age determined via PBT was sufficiently high (>99.8%) to justify application to mixed‐stock fishery sample evaluation.

### 4.2 Exploitation rate

Exploitation rates of Chinook salmon in 2019 Canadian marine fisheries derived from CWTs and genetics were highly comparable for 12 of 13 populations evaluated, with the Cowichan River population the exception. For that population, the exploitation rate derived from CWTs (51.2%) was higher than from genetics (34.5%). However, the number of CWTs observed in the escapement (estimated 592 CWTs) was considerably less than the number of adipose fin‐clipped individuals (2488) observed in the escapement estimate of 18,109 Chinook salmon (K. Pellett, Fisheries and Oceans, pers. comm.), as both values should be comparable since all adipose fin‐clipped individuals carry a CWT. Failure to recover some CWTs during escapement sampling led to overestimation of exploitation rate derived from CWTs. The estimate of exploitation rate for the Cowichan River population derived from genetics was similar to those of other populations on the east coast of Vancouver Island (30.1%–39.9%).

Age‐specific exploitation rates across populations were comparable between CWTs and genetics for ages 3–6 years, with the age 5‐year discrepancy limited to the Big Qualicum River population. In that instance, 2.4 times as many PBT identifications were made (94) as CWTs recovered (39) in the fishery sampling, with sampling error with respect to CWTs likely attributable for the discrepancy. Jack exploitation rates were estimated as 0.0% for three populations via CWTs (Big Qualicum, Harrison, Atnarko), whereas exploitation rates estimated via genetics ranged from 8.9% to 57.3%. This discrepancy in estimated exploitation was a result of no jacks from these populations observed in fishery samples via CWTs but they were present in the escapement through CWT or scale observations. In contrast, jacks were observed in the fishery samples for these populations via PBT. Given the observed accuracy in identifying jacks via PBT, it was unlikely that this discrepancy was a result of errors in age determination of individuals in fishery samples via PBT. PBT identification did not require an individual to be adipose fin clipped before detection, so perhaps there was less likelihood of an adipose fin‐clipped jack to be sampled in fisheries than an intact jack. It is possible that individual fishers may have declined to have jacks sampled in the creel survey, as they may not have wanted an already small fish to be beheaded for CWT detection.

### 4.3 Conservation Unit fisheries and escapement management and assessment

For the first time, Chinook salmon fisheries impacts in Canada were available by CU, and was a strategic development in implementation of Canada's WSP. The GSI‐PBT approach to fishery assessment enables catch by CU to be determined for any Canadian fishery, and provides for managing a combination of mixed‐stock ocean fisheries and potential in‐river fisheries that exploit only healthy CUs as envisioned by Price et al. (2017). The use of PBT to identify members of hatchery or wild indicator populations and GSI to identify remaining individuals in the catch identifies the previously unknown components of the harvest when assessed with CWTs.

With the genetics approach to fisheries assessment, it may also be possible to estimate wild escapement by CU. The proposed approach could use late season fishery information, combined with representative GSI‐PBT information from the fishery and escapement results from key hatchery indicator populations, to form the basis for estimating CU escapement of wild Chinook salmon. The key assumption is that ratio of catch of the CU divided by the catch of the indicator population in the CU in the same fishery equals the equivalent ratio in escapement. That is, within a fishery near the end of the season:

$$C_{\text{ws}}{\text{/C}}_{\text{hs}}=E_{\text{ws}}/E_{\text{hs}}$$

where C_ws_ = Catch (encounters) of Chinook salmon delineated by CU from samples analyzed by GSI and C_hs_ =Catch (encounters) of a hatchery‐marked indicator population of Chinook salmon from samples analyzed by PBT and GSI where required. A hatchery‐marked (or adipose clipped) Chinook salmon would be first analyzed by PBT; with those not identified by PBT then run through regular GSI DNA methods. E_hs_ = Escapement of hatchery‐marked Chinook salmon in the indicator population, for example, Quinsam River for the ECVI north CU. E_ws_ = Escapement of wild Chinook salmon by CU which is the unknown that can be calculated. Escapement in the Quinsam River is sampled and estimated, and otoliths from the Quinsam River population are thermally marked prior to hatchery release. Very high levels of GSI accuracy (99.9%) for this population have been observed in known‐origin samples (TD Beacham, C Wallace, K Jonsen, BJG Sutherland, C Gummer, and EB Rondeau unpublished data), and thus catch of this population can be estimated accurately. By determining catch by CU, as well as catch and escapement of the Quinsam River population, an estimate can be made for the escapement of other populations in the CU, which will all be wild in origin.

### 4.4 Hatchery management

As the CWT system is impaired by inadequacies in sampling and assessment associated with mass marking of hatchery fish, some of which are released without a CWT, Canada has not implemented mass marking of Chinook salmon hatchery production partially over concerns for the integrity of the current CWT system of assessment. An unintended consequence of this decision is the inability to harvest only hatchery‐origin individuals in mark‐selective fisheries, as there is no way to identify visually hatchery‐origin individuals. This lack of harvest can subsequently lead to straying of hatchery‐origin individuals into wild populations, and the inclusion of strays into hatchery broodstocks for populations of conservation concern. The question naturally arises as to whether a more appropriate fisheries assessment system can be applied that is complementary to mass marking of hatchery production via a visible mark such as an adipose fin clip.

The obvious solution to the previous question is a genetics‐based system of fishery assessment. If all hatchery production were adipose‐fin clipped prior to hatchery release, mark‐selective fisheries could be implemented which may allow improved fishing opportunities for Chinook salmon in BC. With a visible mark denoting hatchery origin of an individual, hatchery broodstock management and assessment of hatchery production with either harvest augmentation or conservation goals could be implemented. Mass marking enables hatchery managers to ensure the inclusion of naturally produced fish in the broodstock if desired, and removal of hatchery‐produced fish at fences or weirs in the natural environment to control the relative influences of the natural and hatchery environment on hatchery‐supplemented populations in which gene flow between the two spawning environments takes place (Mobrand et al. 2005). Moreover, mass marking combined with parentage analysis enables assessment of the reproductive success of hatchery‐produced fish that return to spawn in the natural environment (Abadia‐Cardosa et al. 2013; Ford et al. 2015).

Substantial improvements in assessment could be possible if a genetics‐based assessment of fishery and hatchery broodstocks were implemented. Genetic identification does not require lethal sampling, provides the sex of the sampled individual, and allows sampling and release of fish at all life stages if required. In contrast, recovery of CWTs requires lethal sampling, precluding the subsequent release of sampled individuals and determination of the sex in juvenile samples. Sampling for genetic analysis requires only a tissue sample (as little as a mucous swab or scale) for analysis, while recovery of CWTs requires heads or snouts of individuals to be sampled. Whereas CWTs are of limited use in the study of hatchery‐wild interactions, the non‐lethal and simple tissue sampling has made genetic analysis of interactions commonplace in ecological studies (Sekino et al. 2005; Denson et al. 2012; Ashton et al. 2016). Moreover, the hatchery pedigree that can be obtained using PBT enables direct estimation of inbreeding and outbreeding effects in hatchery production and estimation of genetic parameters such as heritability (Kozfkay et al. 2008, Berejikian et al. 2017).

### 4.5 Utility of PBT‐GSI for fisheries assessment

In 2004, the PSC convened an expert panel to examine limitations of the CWT program for both Chinook salmon and coho salmon, and to evaluate the capacity of alternative technologies to provide data to improve assessment of salmon. The panel noted that PBT could provide the equivalent of CWT recovery data, but that an empirical demonstration was needed to validate theoretical PBT results that suggested broad feasibility (PSC, 2005). With no large‐scale PBT applications developed in the intervening years, the PSC again commissioned in 2014 an evaluation of the feasibility and cost‐effectiveness of developing a coordinated coastwide tag recovery system using PBT, stipulating that a transition from the coastwide CWT system to a PBT system would require that:

1. The PBT system generate at least the same information currently generated from the CWT system via run reconstruction (cohort) analyses of estimated recoveries from individual CWT release groups.
2. The PBT system would have long‐term annual operating costs no greater than or, ideally, substantially less than those of the existing CWT system.
3. The cost of a coastwide PBT system was *substantially* less than that of the existing CWT system or that PBT delivers additional or novel information, not provided by the existing CWT system, to inform management of fisheries for coho and Chinook salmon (PSC, 2015).

The result of the 2014 PSC request for the previously noted evaluation led to the non peer‐reviewed report of Satterthwaite et al. (2015). Various scenarios were explored in the report, and the report has been currently interpreted as concluding that the transition from a CWT‐based assessment system to a genetics‐based system is not cost effective or feasible (PSC Southern Endowment Fund Committee, pers. comm.).

The results of the current study, as well as those of Beacham et al. (2018), allowed an evaluation of the whether a GSI‐PBT system of assessment for Chinook salmon in Canadian fisheries can meet the three criteria outlined by PSC (2015). For 2019 Canadian fishery sampling, similar overall population exploitation rates were observed between CWTs and genetics. For the Cowichan River population specifically, more representative exploitation rates were obtained from the genetics application than from CWTs. Non‐jack age‐specific exploitation rates spanning four age classes were again similar between CWTs and genetics. With respect to jacks, more representative exploitation rates were potentially derived from genetics than CWTs. We conclude that the first requirement for transition from a CWT‐based assessment method to a genetics method as outlined by the PSC has been met for Canadian populations.

With respect to costs (requirement 2), current CWT marking plans for Chinook salmon released from hatcheries in BC include marking 6,275,000 individuals with a CWT. Current cost of a CWT is $0.13 Cdn, with the cost of tag insertion and fin clipping estimated at $0.14 per individual (M. Thom, DFO, pers. comm.), as well as an annual cost of $30,000 for maintenance of CWT machines and ETD equipment. Total annual cost of CWT tagging is estimated at $1,724,250 (6,275,000 × 0.27 + 30,000). Comparable PBT costs for “marking” would be genotyping of all broodstock at hatcheries where CWTs are currently applied, as well as some additional populations (Table S3), which currently is approximately 20,000 individuals at a $20/fish genotyping charge (equipment maintenance included), for a total of $400,000.

Approximately 8000 CWTs for Chinook salmon have been recovered annually from fisheries in BC prior to 2019, with a cost of $5/fish (Satterthwaite et al. 2015) estimated to recover and read the CWT. Approximately 5400 CWTs are recovered annually from escapement sampling for Chinook Salmon in BC. A total cost of $67,000 (13,400×$5) is therefore estimated for tag recovery and reading of fishery and escapement samples of Chinook salmon. Direct cost of activities associated with the CWT program for Chinook salmon that would be no longer conducted under a GSI‐PBT program total $1,791,750 ($1,724,250 marking + $67,000 tag recovery and reading). If CWTs were no longer applied, an ETD system would no longer need to be conducted, but the annual cost of running this system has not been included in the estimation of cost differentials between the two programs.

Under a GSI‐PBT program, suppose that 17,000 Chinook salmon are sampled in fisheries in BC (approximately 4500 more than in 2019). Sampling of freshwater fisheries in the Fraser River as well as marine fisheries along the west coast of Haida Gwaii would prove beneficial for increased coverage for genetics assessments. Chinook salmon could be sampled from escapements as is done now under the CWT program. Note however that escapement age determination in BC is currently done via both CWTs and scale analysis, and in the absence of CWTs could be done entirely via scale analysis. Age determination of Chinook salmon via scales has been reported as relatively accurate (McNicol & MacLellan, 2010), but additional confirmation of the accuracy would be desirable. If escapements were sampled only for age determination, the genotyping cost associated with escapement sampling could be added to additional fishery sampling. Fishery sampling could thus be increased to 23,000 individuals at a cost of $460,000 (23,000 × $20), and when combined with the broodstock genotyping costs ($400,000), total genotyping cost for broodstock, fishery, and escapement sampling analysis would be $860,000. The relative cost differential between the CWT program ($1,791,750) and the PBT program ($860,000) for Chinook salmon in BC is $931,750 in favor of the PBT program, or about 48% of the cost of the CWT program, and with the cost of running the ETD system not included in the cost of the CWT program. However, the infrastructure currently associated with the CWT program is an additional cost and gap in the genetics program. That is, the cost of data management, standards, collection, storage, reporting, and access to hatchery data systems and international treaty models has not been included in costs for either assessment method. The principal reason that a genetics‐based assessment system is more cost effective than a CWT system in Canada is that with survival rates of hatchery‐origin salmon currently <2%, 98% of the cost of applying tags to juveniles is simply wasted, as there are limited returns from which to retrieve tags. The assertion that a genetics‐based Canadian fishery assessment program is not cost effective or feasible at this time is without foundation, and ignores demonstrated applications reported in the peer‐reviewed literature (Beacham, Jonsen, et al., 2020; Beacham et al.,^2018^; Beacham, Wallace, Jonsen, McIntosh, Candy, Willis, Lynch, Moore et al. 2019).

With respect to requirement 3 of the PSC concerning additional or novel information, a PBT assessment system can be seamlessly combined with a GSI management system to provide fishery assessment to include wild population conservation and management of enhancement programs, as well as the ability to monitor sublegal size Chinook salmon. The successful application of current genetic technologies in this study, allowing accurate identification of Chinook salmon sampled from mixed‐stock fisheries, has enabled the first Canadian assessment of fishery impacts that is sufficiently informative for conservation‐based management as envisaged in the WSP. Additional or novel information that can be obtained via PBT has been outlined in a parallel GSI‐PBT program for coho salmon by Beacham, Wallace, Jonsen, McIntosh, Candy, Willis, Lynch & Withler et al. (2019), who concluded that application of PBT provided valuable information for assessment and management of hatchery‐origin coho salmon in BC. Additionally, the performance of tagged CWT indicator populations is based on an expectation of biological homogeneity over the geographic region represented by the indicator population. The indicator population is expected to be representative, in terms of life history, marine distribution, productivity, and exploitation pattern, of other populations within the indicator region, but this basic assumption of the CWT assessment method may not be valid in some regions (Beacham, Wallace, Jonsen, McIntosh, Candy, Willis, Lynch & Withler et al. 2019).

When fisheries sampling was conducted, no distinction was made between Chinook salmon displaying an adipose fin clip and unclipped individuals. Therefore, a GSI‐PBT program could proceed on the basis of no marking or limited visual marking of hatchery‐origin salmon as is done now with the CWT program. However, if all hatchery‐origin Chinook salmon were visually marked with an adipose fin clip before hatchery release, fishery sampling could be targeted toward only the hatchery component, only the wild component, or both, depending upon the objective of the sampling. In essence, a GSI‐PBT approach to fishery assessment provides an opportunity for mass marking of Canadian Chinook salmon hatchery production prior to hatchery release, and at no increased cost to an assessment program. In the CWT‐based assessment program, release of adipose fin‐clipped individuals without a CWT leads to degradation of the assessment system and was the reason for convening the expert panel in 2004 to assess alternatives for the system at the time. One issue associated with the reluctance of Canada to mass mark Chinook salmon hatchery production has been the potential impact on the assessment capability of the CWT program and the associated increased costs in conducting a suitable CWT‐based assessment program. With no way to visually distinguish between hatchery‐origin and wild‐origin Chinook salmon, concerns for the integrity of the CWT assessment system have stifled appropriate management of hatchery broodstocks and development of mark‐selective fisheries. A GSI‐PBT method of assessment would facilitate improvements in both of these areas.

One concern often raised in implementation of mark‐selective fisheries is the estimation of incidental mortality of unmarked released individuals. As noted by Satterthwaite et al. (2015), current attempts to evaluate the effect of mark‐selective fisheries rely on double‐index tag groups of individuals, in which hatchery releases of adipose fin‐clipped and CWT‐marked individuals are paired with individuals that receive a CWT but no corresponding adipose fin clip. However, CWTs cannot be obtained from individuals released in a mark‐selective fishery, so the impact of specific fisheries on mortality of unmarked individuals cannot be evaluated directly. Relative measures of survival may be estimated by recovery of CWTs from clipped and unclipped individuals in the escapements. In theory, a similar approach could be followed with a genetics‐based evaluation, with PBT being used to identify hatchery‐origin individuals in fishery sampling. If the clip status of the individual is recorded, differential impacts of the fishery on retained and released individuals could be directly estimated. Genetics provides the opportunity to evaluate fishery impacts on the released unmarked portion of the catch in a mark‐selective fishery, provided that a DNA sample is obtained prior to release of the unmarked individual.

In implementation of GSI‐PBT system of assessment for Canadian fisheries, the question of the appropriate sampling level for fisheries arises if a population of low abundance or conservation concern is present in fisheries. For Chinook salmon in BC, the population of conservation concern centers on the Nicola River population, a spring‐run population in the lower Thompson River, a major tributary of the Fraser River drainage. As Chinook salmon fishery sampling incorporating PBT did not commence until 2018, and initially only in a limited manner, direct comparisons between CWT and PBT identifications were available only for 2019. In 2019, the number of Nicola River‐origin CWTs recovered from marine fisheries in Canada was one CWT, and virtually 100% of hatchery production of this population is already marked with CWTs. Thus, there is no way to amplify the marking rate for this population. For genetics, two PBT identifications were observed in 2019 marine fishery sampling. Thus, there was little evidence to indicate that genetic analysis was less likely to identify Nicola River Chinook salmon, even though <3.5% of the catch was genotyped. Thus, there is no evidence to indicate that PBT analysis based upon current genetics sampling programs are less able to detect populations of conservation concern compared with the current CWT assessment program. Should the level of fisheries genotyping be increased to 23,000 individuals as noted previously, 84% higher than actually conducted in 2019, the increased sampling effort can be directed toward those fisheries and times where Nicola River‐origin individuals are known to have been caught, such as in lower Fraser River fisheries.

### 4.6 Future developments

The current study has demonstrated the GSI‐PBT capability to identify BC‐origin Chinook salmon to specific Canadian hatcheries and CUs, allowing the option of replacement of the current CWT system for Chinook salmon assessment in BC with a GSI‐PBT‐based approach. The 391‐SNP panel used in the current study to genotype the 2016–2018 Chinook salmon broodstocks and 2018–2019 mixed‐stock fishery samples has been upgraded. A 547‐SNP panel now exists, and has been used to genotype the 2019 Chinook salmon broodstocks at an expanding number of hatcheries (Table S3). It is anticipated that this enhanced SNP panel and the increasing number of facilities at which broodstock genotyping is occurring will provide improved stock composition results relative to those of the current study when applied to Chinook salmon fishery samples in 2022. If Canada were to implement a GSI‐PBT method of assessment, then complete assessment of exploitation rates for Canadian populations would require genetic analysis of samples from American fisheries if CWTs are retained as the assessment tool for American fisheries. Should a GSI‐PBT method of analysis be deemed practical for American assessment purposes, it is conceivable that a coastwide GSI‐PBT assessment method could be implemented for Chinook salmon fisheries. In any event, adoption of a GSI‐PBT method of domestic fishery assessment allows Canada the option to consider the implementation of mass marking via the adipose fin clip of all Chinook salmon hatchery production at hatcheries where it is feasible to do so.

Coded‐wire tag‐based marking of subyearling Chinook salmon of the 2019 broodyear was severely impacted in BC by the emergence of the COVID‐19 pandemic in early 2020. Due to human health and safety concerns, only about 5% of the intended number of individuals were marked with CWTs, and partial marking of the hatchery production was restricted to four hatcheries. This lack of tagging will severely impact assessment of Canadian Chinook salmon fisheries via CWTs in 2022 and 2023, as age 3‐ and 4‐year‐olds comprise the majority of the returns in BC. However, about 20,000 individuals were genotyped from the 2019 hatchery broodstocks, and a genetics‐based assessment method should provide comparable information to that which would have been derived from the CWT mark recovery program and subsequent assessment.

### 4.7 Summary

Parentage‐based tagging provided an effective method to identify the age and hatchery of origin of individual Chinook salmon. GSI provided identification of mixed‐stock fishery samples to CU, a requirement for implementation of management of wild populations as mandated by Canada's WSP for Pacific salmon. With 3.24% of the 2019 Chinook salmon catch in BC genotyped, fishery impact assessments via genetics were generally comparable to those via CWTs. When GSI and PBT were combined in a concurrent application, the GSI‐PBT method of fisheries assessment for Canadian populations outperformed a CWT‐based assessment method on many levels, including stock composition resolution, cost, and ancillary information. Moreover, unlike CWTs, GSI‐PBT‐based assessment benefits from the external (allowing visual recoveries) mass marking of hatchery‐produced salmon, thereby facilitating improved hatchery broodstock management, monitoring of wild‐enhanced fish interactions, and the evaluation of hatchery contributions to harvest. The ability to identify easily hatchery‐produced salmon has been recognized as important for managing the risks and assessing the benefits of hatchery production of salmonids at the domestic, bilateral, and international levels.

## CONFLICT OF INTEREST

There are no conflicts of interest to declare.

## Supporting information

Supplementary Material

## Data Availability

Multi‐locus genotypes for all individuals sampled in 2018 and 2019 fisheries, as well as all hatchery broodstocks, will be available at DRYAD upon manuscript acceptance.
