# Supplementary material for: Parentage‐based tagging combined with genetic stock identification is a cost‐effective and viable replacement for coded‐wire tagging in large‐scale assessments of marine Chinook salmon fisheries in British Columbia, Canada
Source: Evol Appl. 2021 Mar 19;14(5):1365–89. doi: 10.1111/eva.13203 (PMC8127719; doi:10.1111/eva.13203)

1 Supplementary Methods

- 1. Comparing creel survey and Avid Anglers catch stock compositions

Differences in stock composition between the Avid Anglers and the creel survey program were evaluated by Fisher’s exact test (Fisher 1954), with pooling of catches from CUs into these evaluation groups; 1) Klinaklini_summer_1.3, Southern Mainland-Southern Fjords_fall_0.x, Southern Mainland-Georgia Strait_fall_0.x; 2) upper and middle Fraser River (5 CUs); 3) Thompson River (7 CUs); 4) lower Fraser River (5 CUs); 5) East Vancouver Island-North_fall_0.x, East Vancouver Island-Qualicum and Puntledge_fall_0.x; 6) other ECVI CUs (4 CUs); 7) WCVI (3 CUs); and 8) American regions (17 regions). Any comparison between the Avid Anglers and creel survey program stock compositions which had a total of either 0 or 1 estimated individuals in any of the eight previously defined groupswere excluded from the analysis.

2 Supplementary Results

2.1 Heterozygosity and *F*_ST_

Expected heterozygosity ranged from 0.01 to 0.50 across the 389 SNPs surveyed (Table S2, excluding species ID and sex ID markers), with a mean expected heterozygosity of 0.29. Expected heterozygosities were > 0.40 for 35% of the SNPs surveyed (Figure S3). Global *F*_ST_ across SNPs ranged from 0.01 to 0.40, and 39% of the SNPs displayed a *F*_ST_ value between 0.05 and 0.10 (Figure S4). The mean *F*_ST_ value across loci was 0.115. SNPs included in the panel included many from a previous version of the panel (Beacham et al. 2018) and with the addition of new EPIC4 SNPs (Table S2). The EPIC4 SNPs were chosen for their initial stock identification capability separating BC populations, so heterozygosities and *F*_ST_ values may not be representative of SNPs present in the genome.

2.2 Genomic distribution of SNPs

The 389 SNPs surveyed were broadly distributed over the 34 chromosomes present in the Chinook salmon genome, ranging from a minimum of one SNP present on chromosome Otsh26 to 21 SNPs present on chromosome Otsh01 (Figure S5). Average marker spacing across the chromosomes was 5.01Mbp. There were 39 SNPs present in scaffolds unassigned to specific chromosomes. SNPs with higher *F*_ST_ values were also widely distributed across chromosomes, so there was no clustering of these SNP sites on specific chromosomes.

2.3 GSI applied to 2018 fishery sampling

Broodstock genotyping at selected hatcheries in BC did not commence until 2013, and thus 2018 marked the first year in which PBT could be applied to identify individuals subject to capture in fisheries (primarily age 3 years to age 5 years, but some age 2 and age 6 years can also be present). It also marked the first year in which the newly-developed SNP baseline was extensive enough to allow GSI estimates to be made with confidence to CU for fisheries in BC, as well as identifying American-origin contributions to catch. Sampling in the northern troll fishery (Areas 1 and 101) in 2018 indicated that exploitation was centered on mainly migrating stocks, as north or central coast CUs contributed little to the fishery (Supplementary Table S4). The recreational fishery at the north end of Haida Gwaii (Area 1) exploited Chinook salmon in more inshore areas than the commercial troll fleet, and this was reflected in observed stock compositions. As might be expected, given the difference in physical fishing locations of the commercial troll and recreational vessels, stock compositions of the recreational samples reflected less dependence on migrating stocks located further offshore, and greater dependence of contributions from CUs or more local distribution, such as the limited June sample (n=18) which indicated significant contributions from populations in local CUs (Kitsumkalum late CU 16.7%, Wannock CU, 16.7%, Rivers Inlet 5.6%).

In the recreational fishery north of Johnstone Strait (Area 11), Chinook salmon from the WCVI Nootka and Kyuquot fall age 0.x CU were an important component of the catch from June through August, peaking at 78.0% of the catch in August (Table S4). In Area 12, the north end of Johnstone Strait immediately south of Area 11, the ECVI north fall age 0.x CU was present in the catch, ranging from 8.8% to 11.0% of the June through August catch.

In the northern Strait of Georgia (SoG), Chinook salmon were present in the area throughout the year, including the late fall and winter period. Chinook salmon originating from the ECVI-Qualicum and Puntledge fall age 0.x CU and Cowichan and Koksilah fall age 0.x CU were the dominant contributors to the winter fishery, contributing up to approximately 50% and 30% respectively of the fishery for legal-sized Chinook salmon (> 62 cm fork length). Chinook salmon originating from Puget Sound in Washington were also important contributors to the winter fishery, contributing in some months over 50% of the catch of legal-sized Chinook salmon (Table S4).

In the southern SoG recreational fishery, Chinook salmon also were present in the area throughout the year, including the late fall and winter period. In contrast to the northern SoG, Chinook from Puget Sound in Washington dominated the January through March catch, ranging from 76.5% to 88.9% of the legal catch. Chinook salmon from the lower Fraser River fall age 0.3 CU, after comprising 8.8% of the legal catch in March, were major contributors to the April and May catch, comprising 67.5% and 40.7%, respectively, and thereafter contributed significantly to the legal catch through September, abruptly declining thereafter and remaining low for the duration of the year.

In the Juan de Fuca Strait (JDF) recreational fishery, American-origin Chinook salmon, largely from Puget Sound, dominated the catch in the fishery from January through May, generally above 90% of the catch in any month. By June, Chinook salmon from CUs of conservation concern, such as the Upper Fraser River spring age 1.3 (3.1%), Middle Fraser spring age 1.3 (7.8%), North Thompson summer age 1.3 (2.0%), South Thompson summer age 1.3 (2.0%), and Lower Thompson spring age 1.2 (2.0%) were observed in the fishery (Table S4). By October, catch in the fishery was largely American in origin.

Marked changes in monthly stock compositions were observed in the southern west coast of Vancouver Island (WCVI) recreational fishery, as well as the fishery exploiting migrating stocks in the early summer. For example, Puget Sound stocks contributed 51.0%, 33.2%, 13.6%, and 0.0% to the June, July, August, and September samples, respectively. Conversely, the local WCVI South fall age 0.x CU contributed 1.5%, 12.7%, 46.2%, and 80.4%, respectively. Chinook salmon originating from CUs of conservation concern were observed in the fishery in June (Upper Fraser spring age 1.3 7.4%, middle Fraser spring age 1.3 1.5%, middle Fraser summer age 1.3 1.0%) and July (North Thompson spring age 1.3 1.4%, North Thompson summer age 1.3 1.5%) (Table S4).

2.4 GSI applied to 2019 fishery sampling

As noted previously, substantial restrictions were placed on the 2019 fishery compared with 2018 due to conservation concerns related to the rockslide in the middle portion of the Fraser River drainage. Although the fisheries were more restricted in 2019, more extensive PBT and GSI fishery sampling was conducted in 2019 compared with 2018, as the objective of the 2019 fishery sampling was to sample the same fisheries as would be done for CWT sampling which would lead to a direct comparison of CWT- and GSI and PBT-based assessments. With the opening of the northern troll fishery delayed until mid August and extending into late September, migrating stocks formed the basis of the fishery (Table S5). In the recreational fishery at the north end of Haida Gwaii, Chinook salmon from Pacific Northwest sources comprised increasingly important components of the catch as the season progressed from May to September, with the coastal Washington component increasing from 0.0% in May to 19.4% in September, as did the upper Columbia River summer/fall stock (9.1% to 20.4%) and the northern/central Oregon stock (1.8% to 26.8%).

Newly-sampled central coast fisheries in 2019 included a local First Nations fishery where the major contributor to the fishery was Chinook salmon from the Bella Coola River-Bentinck Arm CU (29.0%), the WCVI south fall age 0.x CU (10.0%), and the ECVI north fall age 0.x CU (10.3%). Next was a terminal gillnet fishery near the mouth of the Bella Coola River, and unsurprisingly 90.5% of the catch was estimated to originate from the Bella Coola- Bentinck CU. Lastly, an Area 10 recreational fishery was sampled, with Area 10 just north of Area 11, and the most southern statistical area in the central coast. In June and July, the WCVI-Nootka and Kyuquot fall age 0.x CU (36.0% - 42.9%) and WCVI south fall age 0.x CU (21.4%-40.0%) were major contributors to the fishery.

In the recreational fishery north of Johnstone Strait (Area 11), as in Area 10, Chinook salmon from the WCVI Nootka and Kyuquot_fall_0.x CU were an important component of the catch in June and July, accounting for 40.2%-42.5% of the catch, but declining in August to 5.0%). In the recreational fishery in northern Johnstone Strait (Area 12), major contributors to the July and August catch were Chinook salmon from the South Thompson summer age 0.3 CU (23.0% – 29.8%), the WCVI south age 0.x CU (15.0% - 23.0%), and the ECVI-Qualicum and Puntledge_fall_age 0.x CU (7.7% - 14.2%).

In the northern SoG, Chinook salmon were again present in the winter, as in 2018. Chinook salmon originating from the ECVI-Qualicum and Puntledge_fall_age 0.x CU and Cowichan and Koksilah_fall age 0.x CU were the dominant CUs in the winter fishery, contributing 10.9% - 43.6% and 23.1% - 58.8%% respectively of the fishery for legal-sized Chinook salmon. As in 2018, the relative abundance of the lower Fraser River fall age 0.3 CU increased in May, and comprised 20.0% of the legal-sized fishery.

In the southern SoG, Chinook salmon were again present in the winter, as in 2018. Chinook salmon originating from the ECVI-Qualicum and Puntledge_fall_age 0.x CU and Cowichan and Koksilah_fall age 0.x CU were the contributors to the winter fishery, but the lower Fraser River fall age 0.3 CU was also present (Table S5). As in 2018, Chinook from Puget Sound in Washington dominated the January through March catch (Figure 3).

In the JDF recreational fishery, with the fishery restricted owing to conservation concerns, monthly samples > 10 salmon were available only in August and September. Chinook salmon from the South Thompson summer age 0.x CU were the most prevalent (64.9%) in August, with the September fishery largely comprised of the previous CU (31.0%), the WCVI south fall age 0.x CU (27.6%), and the lower Fraser fall age 0.x CU (17.2%).

In the southern WCVI recreational fishery, Chinook salmon from the WCVI south age 0.x CU comprised an increasing proportion of the catch from June (23.6%) to September (92.3%), coincident with migration timing of populations in the CU. Stock composition from a terminal gillnet fishery in Nootka Sound was estimated at 99.8% WCVI-Nootka and Kyuquot fall age 0.x CU, with the Conuma River population estimated to have contributed approximately 90% and the Burman River population 10% to the catch (Table S5).

2.5 Creel surveys versus Avid Anglers

The objective of this portion of the study was to evaluate whether stock identification information from the Avid Anglers was similar to the of the traditional creel survey. In the northern SoG, the Avid Anglers 2018 sampling was conducted both within the time frame of the creel survey (March, June, July, August, September) and outside of the traditional creel survey, as well as including sublegal-sized fish that were released after sampling. Stock compositions could be compared between the Avid Anglers sampling and the creel survey only for the legal-sized component of the catch. No significant difference in stock composition was observed between the Avid Anglers sampling and the creel survey for March, July, August and September (all P>0.05), but a difference was observed in the June sample (χ^2^_(4)_=21.2, P<0.01). The lower Fraser River fall age 0.3 CU comprised 56.3% of the creel survey sample but only 28.6% of the Avid Anglers sample, largely accounting for the significant difference in observed stock composition.

In the southern SoG, comparisons of 2018 monthly stock composition between the creel survey and the Avid Anglers catch were available from March through September. No significant difference in stock composition was observed between the Avid Anglers sampling and the creel survey for March, June, July, August and September (all P>0.05), but a difference was observed in the April (χ^2^_(3)_=9.1, P<0.05) and May samples (χ^2^_(3)_=23.2, P<0.01). In April, the lower Fraser River fall age 0.3 CU comprised 75.6% of the creel survey sample but only 52.7% of the Avid Anglers sample, largely accounting for the significant difference in observed stock composition, similar to the SoG north June comparison. For May, American-origin Chinook salmon constituted 90.9% of the creel sample but only 45.7% of the Avid Anglers sample.

Comparisons of monthly stock composition between the creel survey and the Avid Anglers catch were available in the JDF 2018 recreational fishery on a monthly basis from April through September. No significant difference in stock composition was observed between the Avid Anglers sampling and the creel survey for all months inclusive (all P>0.05).

In 2019 in the northern SoG, significant differences in stock composition were observed between the Avid Anglers sampling and the creel survey for July (χ^2^_(6)_=42.7, P<0.01) and August (χ^2^_(5)_=22.5, P<0.01), but not September (P>0.05). The Klinaklini summer age 1.3, Southern Mainland-Georgia Strait fall age 0.x, Shuswap River summer age 0.3, and South Thompson River summer age 0.3 CU were relatively more abundant in the Avid Anglers sampling (19.0%) than in the creel survey (1.9%). As in 2018 when there was a significant difference between Avid Anglers and creel survey samples, the lower Fraser River fall age 0.3 CU was relatively more abundant in the creel survey (42.6%) than in the Avid Anglers sample (23.2%).

In the southern SoG, no significant differences were observed in stock compositions between the Avid Anglers and creel survey samples for March, July, and September (all P>0.05), but a difference was observed in August (χ^2^_(6)_=26.3, P<0.01). In August, Chinook salmon from ECVI CUs were higher in the Avid Anglers samples (16.1%) than in the creel survey (5.5%), while salmon from Puget Sound were less common in the Avid Anglers samples (3.8%) compared with the creel survey (19.8%). In the JDF recreational fishery, no significant difference in stock composition was observed between the Avid Anglers sampling and the creel survey for either August or September (P>0.05). Overall, no significant differences in stock composition were observed between the creel survey and the Avid Anglers in 21 months of comparisons (78% of total comparisons) in 2018 and 2019, with differences noted in six months (22% of comparisons).

Supplementary Table S1. Chinook salmon spawning locations, sample collection years, and total number of fish genotyped for 380 populations in one Russian and 24 American geographic areas, and 68 Canadian Conservation Units (CUs) ranging from Russia to California. N is the number of fish genotyped in the population.

| Region/Conservation Unit | CU Number | Population | Years | N |
| --- | --- | --- | --- | --- |
| Russia |  | Apuka_River | 2005 | 29 |
|  |  | Avacha River | 2003 | 34 |
|  |  | Bistraya River | 2001 | 80 |
|  |  | Bolshaya River | 2002, 2004 | 64 |
|  |  | Icha River | 2004 | 50 |
|  |  | Kamchatka River | 2003 | 45 |
|  |  | Karymay River | 2002 | 35 |
|  |  | Kikchik River | 2003 | 33 |
|  |  | Kol River | 2003 | 47 |
|  |  | Pahacha River | 2002 | 81 |
|  |  | Palana River | 2002 | 49 |
|  |  | Pymta River | 2002 | 94 |
|  |  | Tigil River | 2002 | 39 |
|  |  | Vorovskaja_River | 2003 | 49 |
| Alaska_Koyukuk River |  | Henshaw Creek | 2015 | 100 |
|  |  | Koyukuk River mid fork | 2010, 2011, 2012 | 50 |
|  |  | Koyukuk River south fork | 2003 | 50 |
| Alaska_Tanana River |  | Chatanika River | 2001, 2007 | 47 |
|  |  | Chena River | 2001 | 47 |
|  |  | GoodpasterRiver | 2006, 2007, 2011 | 77 |
|  |  | Kantishna River | 2005 | 104 |
|  |  | Salcha River | 2005 | 94 |
| Alaska_Lower Yukon River |  | Andreafsky River | 2003 | 96 |
|  |  | Andreafsky River east fork | 2002 | 28 |
|  |  | Anvik River | 2002, 2007 | 87 |
|  |  | Archuelingik River | 2002, 2003 | 60 |
|  |  | Kateel River | 2002, 2012 | 60 |
|  |  | Gisasa River | 2001 | 65 |
|  |  | Nulato River north fork | 2012 | 36 |
|  |  | Nulato River south fork | 2012 | 55 |
| Alaska_Mid Yukon River |  | Melozitna River | 2003 | 14 |
|  |  | Tozita River | 2002 | 98 |
| Alaska_Upper Yukon River |  | Beaver Creek | 1997 | 90 |
|  |  | Chandalar River | 2003, 2004 | 98 |
|  |  | Kandik River | 2007, 2008, 2009, 2010 | 57 |
|  |  | Sheenjek River | 2002, 2004, 2006, 2011 | 66 |
|  |  | Coleen River | 2011, 2013 | 60 |
|  |  | Teedraanjik River | 2016, 2017 | 48 |
| Northern Yukon River and tributaries (NYR) | 76 | Chandindu River | 2004 | 105 |
|  |  | Klondike River | 1999, 2002, 2004, 2006, 2007, 2009, 2010, 2011, 2012, 2013 | 165 |
|  |  | Yukon Rive lower | 2006, 2007 | 175 |
| Salmon Fork (SFork) | 77 | Salmon Fork River | 2015 | 43 |
| White and tributaries (White) | 75 | Nisling River | 2004, 2005, 2015 | 40 |
|  |  | Tincup Creek | 2009, 2011, 2012 | 134 |
| Porcupine (Por) | 78 | Porcupine River | 2007, 2010, 2016 | 237 |
| Nordenskiold (Norden) | 71 | Nordenskiold River | 2006, 2012, 2013, 2016 | 96 |
| Upper Yukon River (upper YR) | 69 | Takhini River | 2002, 2003, 2005 | 118 |
|  |  | Teslin Lake | 2006 | 165 |
|  |  | Teslin River | 2006, 2009, 2010, 2011, 2013 | 195 |
|  |  | Whitehorse hatchery | 1997 | 99 |
| Middle Yukon River and tributaries (MidYR) | 73 | Big Salmon River | 2004, 2006, 2007, 2008, 2014, 2016, 2017 | 196 |
|  |  | Little Salmon River | 2005, 2006, 2007, 2010 | 197 |
|  |  | Tatchun River | 2003, 2005 | 95 |
| Yukon River-Teslin headwaters (Teslin) | 68 | Hundred Mile Creek | 2015, 2016 | 55 |
|  |  | McNeil_River | 2016 | 25 |
|  |  | Morley River | 2002, 2003, 2005, 2006, 2008, 2009, 2010, 2011, 2016, 2017 | 114 |
|  |  | Nisutlin River | 2014, 2015, 2016 | 164 |
|  |  | Swift River | 2013, 2016 | 35 |
|  |  | Wolf River | 2003, 2014, 2015, 2016 | 91 |
| Pelly (Pelly) | 72 | Blind Creek | 2005, 2007, 2008 | 134 |
|  |  | Earn River | 2003, 2004 | 43 |
|  |  | Glenlyon River | 2003, 2004, 2005 | 61 |
|  |  | Hoole River | 2003, 2004, 2005, 2015, 2016, 2017 | 107 |
|  |  | Kalzas River | 2003, 2004, 2011 | 73 |
|  |  | Pelly River | 2009, 2014 | 59 |
|  |  | Ross River | 2006, 2014, 2015, 2016 | 100 |
| Stewart (Stew) | 74 | Mayo River | 2003, 2009, 2011 | 57 |
|  |  | McQuesten River | 2011, 2012, 2014 | 137 |
|  |  | Stewart River | 1996, 2005, 2006 | 140 |
| South East Alaska |  | Big Boulder Creek | 1992, 1995, 2004 | 105 |
|  |  | Chickamin River | 1999 | 97 |
|  |  | King Salmon River | 1993, 1999, 2007, 2008, 2010 | 106 |
|  |  | Situk River | 1988, 1990, 1991 | 111 |
|  |  | Tahini River | 1992, 2004 | 140 |
|  |  | Unuk River | 1999 | 103 |
| Alsek (Alsek) | 67 | Blanchard River | 2002, 2003 | 96 |
|  |  | Goat Creek | 2007, 2009, 2011, 2012, 2013 | 100 |
|  |  | Klukshu River | 2001 | 88 |
|  |  | Kudwat Creek | 2008, 2010, 2011 | 69 |
|  |  | Takhanne River | 2002, 2003, 2008, 2010, 2011 | 99 |
|  |  | Tatshenshini River | 2001 | 24 |
| Unuk (Unuk) | 59 | CrippleCreek | 1988, 2003 | 135 |
| Taku_early timing (Taku early) | 63 | Dudidontu River | 2005, 2008 | 81 |
|  |  | Nahlin River | 2006, 2007 | 97 |
|  |  | Tseta Creek | 2008, 2010 | 210 |
| Taku mid timing (Taku mid) | 64 | Little Tatsamenie River | 2005, 2006, 2007 | 135 |
|  |  | Nakina River | 2005, 2006 | 114 |
|  |  | Tatsmenie Lake outlet | 2005 | 36 |
|  |  | Yeth River | 2008, 2009, 2010 | 53 |
| Taku_late timing (Taku late) | 65 | Hackett River | 2008 | 95 |
|  |  | Kowatua Creek | 1989, 1990, 2005 | 141 |
| Stikine early timing (LSTK early) | 60 | Christina Creek | 2002 | 86 |
|  |  | Johnny Tashoots Creek | 2001, 2004, 2005, 2008, 2009 | 89 |
|  |  | Little Tahltan River | 2010 | 130 |
|  |  | Shakes Creek | 2001 | 90 |
|  |  | Tahltan River | 2008, 2009, 2011 | 151 |
| Stikine late timing (LSTK late) | 61 | Craig River | 2002 | 91 |
|  |  | Tuya River | 2008, 2009, 2011, 2012, 2013 | 40 |
|  |  | Verrett River | 2007, 2009, 2010 | 118 |
| Haida Gwaii-North (HGN) | 43 | Yakoun River | 1996, 2001, 2016 | 77 |
| Upper Nass (UNR) | 58 | Cranberry River | 1996, 1997 | 62 |
|  |  | Damdochax River | 1995, 1996, 1997 | 90 |
|  |  | Kiteen River | 2006 | 55 |
|  |  | Kwinageese River | 1996, 1997 | 93 |
|  |  | Meziadin River | 1995, 1996 | 122 |
|  |  | Oweegee Creek | 1995, 1996, 1997 | 100 |
|  |  | Snowbank Creek | 1996 | 47 |
|  |  | Tseax River | 1996, 2002, 2006, 2008 | 86 |
| Portland Sound-Observatory Inlet-Lower Nass (LNR-P) | 57 | Ishkheenickh River | 2004, 2006 | 104 |
|  |  | Kateen River | 2005 | 94 |
|  |  | Kincolith River | 1996 | 133 |
|  |  | Kwinamass River | 2002, 2003 | 41 |
|  |  | Seaskinnish Creek | 1995, 1996 | 59 |
| Ecstall (ECST) | 46 | Ecstall River | 2001, 2002, 2013 | 102 |
| Skeena Estuary (SKEst) | 45 | Kloiya River | 2003, 2011, 2015 | 27 |
| Lower Skeena (LSK) | 48 | Exchamsiks River | 2009 | 95 |
|  |  | Exstew River | 2009 | 95 |
|  |  | Fiddler Creek | 2010 | 93 |
|  |  | Gitnadoix River | 2009 | 63 |
|  |  | Kasiks River | 2009 | 61 |
|  |  | Khyex River | 2010 | 37 |
|  |  | Zymagotitz River | 2006, 2009 | 110 |
| Kalum_early timing (Kalum-E) | 49 | Cedar River | 1996 | 20 |
| Kalum_late timing (Kalum-L) | 50 | Kitsumkalum River lower | 2013, 2014, 2015, 2016 | 559 |
| Zymoetz (Zym) | 80 | Thomas Creek | 2004, 2009, 2010 | 96 |
| Sicintine (Sic) | 81 | Sicintine River | 2010 | 115 |
| Middle Skeena-mainstem tributaries | 54 | Bulkley River Lower | 1999 | 96 |
| (MSK-MS) |  | Kispoix River | 2004, 2006, 2008, 2010 | 98 |
|  |  | Kitseguecla River | 2009 | 95 |
|  |  | Kitwanga River | 2003 | 93 |
|  |  | Kuldo Creek | 2008, 2009 | 95 |
|  |  | Nangeese River | 2010 | 29 |
|  |  | Shegunia River | 2009, 2011, 2012 | 159 |
|  |  | Slamgeesh River | 2005, 2006, 2007, 2008, 2009 | 83 |
|  |  | Suskwa River | 2009, 2011, 2012 | 101 |
|  |  | Sweetin River | 2008, 2010 | 96 |
| Middle Skeena-large lakes (MSK-LGLKS) | 53 | Babiine River | 2010, 2011 | 103 |
|  |  | Bear River | 2012 | 95 |
|  |  | Morice River | 2010, 2011 | 176 |
| Upper Skeena (USK) | 56 | Kluatantan River | 2006, 2008, 2009, 2010 | 33 |
|  |  | Kluayaz Creek | 2007, 2009 | 127 |
|  |  | Otsi Creek | 2007, 2009, 2011 | 89 |
|  |  | Squingula River | 2008, 2009 | 114 |
|  |  | Sustut River | 2012 | 90 |
| Upper Bulkley River (MSK-UprBulk) | 55 | Bulkely River upper | 2016, 2017, 2018 | 73 |
| North and Central Coast- lake (NCC lake) | 41 | Kitlope River | 2004, 2006 | 95 |
| North and Central Coast-streams (NCC-stream) | 42 | Hirsch Creek | 1998, 1999 | 79 |
|  |  | Kildala River | 2000 | 94 |
|  |  | Kitimat River | 2016, 2018 | 1256 |
| Rivers Inlet (RI) | 37 | Ashlulm Creek | 2000, 2002, 2003, 2005 | 51 |
|  |  | Chuckwalla River | 2000, 2001, 2005, 2016, 2017, 2018 | 102 |
|  |  | Kilbella River | 2000, 2005, 2016, 2017, 2018 | 101 |
|  |  | Neechanz River | 2000, 2002, 2003, 2005 | 45 |
| Wannock (WANN) | 38 | Wannock River | 1996, 2016, 2017, 2018 | 300 |
| Bella Coola-Bentinck (BCR-BENT) | 39 | Atnarko River lower | 2013, 2014, 2015, 2016, 2017, 2018 | 2,353 |
|  |  | Atnarko River upper | 2013, 2014, 2015, 2016, 2017, 2018 | 2,499 |
|  |  | Nusatsum River | 1996, 2006, 2016, 2018 | 146 |
|  |  | Salloomt River | 1996, 2006, 2016, 2018 | 110 |
| Dean River (DEAN) | 40 | Dean River lower | 2003, 2004, 2006 | 157 |
|  |  | Dean River upper | 2006 | 38 |
|  |  | Takia River | 2003, 2006 | 40 |
| Docee (DOCEE) | 36 | Docee River | 2002, 2004, 2007, 2010 | 87 |
| Klinaklini_SU_1.3 (KLINA) | 35 | Devereux Creek | 1998 | 61 |
|  |  | Klinaklini River | 2002 | 95 |
| Southern Mainland-Southern Fjords_FA_0.x (Smn-Sfj) | 28 | Phillips River | 2005, 2009, 2013, 2014, 2015, 2016, 2017, 2018 | 486 |
| Southern Mainland-Georgia Strait_FA_0.x (Smn-GStr) | 20 | Ashlu Creek | 2004, 2008, 2016, 2017, 2018 | 35 |
|  |  | Cheakamus River | 2005, 2006, 2007, 2008, 2012, 2017, 2018 | 212 |
|  |  | Cheakamus River-Tenderfoot | 2013, 2016 | 23 |
|  |  | Cheakamus River fall | 2006, 2007, 2008, 2011 | 112 |
|  |  | Cheakamus River summer | 2008 | 38 |
|  |  | Mamquam River | 1996, 2007, 2008, 2012, 2016, 2017, 2018 | 78 |
|  |  | Porteau Cove | 2013 | 92 |
|  |  | Shovelnose Creek | 1996, 2004, 2016, 2017, 2018 | 51 |
|  |  | Squamish River | 1997, 2004 | 48 |
|  |  | Tenderfoot Creek | 2016 | 39 |
| Upper Fraser River_SP_1.3 (UFR spring) | 12 | Bad River (James Creek) | 1984 | 15 |
|  |  | Bowron River | 1997, 2003, 2009 | 65 |
|  |  | Dome Creek | 1996 | 100 |
|  |  | Fontoniko Creek | 1996 | 44 |
|  |  | Goat River | 1997, 2001 | 40 |
|  |  | Holmes River | 1996, 1999, 2002 | 31 |
|  |  | Horsey Creek | 1997, 2000, 2003, 2004, 2010 | 20 |
|  |  | Indianpoint Creek | 1995 | 43 |
|  |  | Kenneth Creek | 2001, 2002, 2004 | 62 |
|  |  | McGregor River | 1997 | 62 |
|  |  | Morkill River | 2001 | 95 |
|  |  | Nevin Creek | 2001, 2003, 2004, 2010, 2011, 2014, 2016 | 19 |
|  |  | Ptarmigan Creek | 2000, 2001, 2004 | 24 |
|  |  | Salmon River | 1997 | 89 |
|  |  | Slim Creek | 1996, 1998, 2001 | 92 |
|  |  | Swift Creek | 1996, 2010, 2012 | 38 |
|  |  | Tete Jaune | 2001 | 72 |
|  |  | Torpy River | 2001 | 71 |
|  |  | Willow River | 1995, 2004 | 63 |
| Middle Fraser River_SU_1.3 (MFR summer) | 11 | Cariboo River | 1996, 2007, 2008 | 77 |
|  |  | Chilko River | 2014, 2015, 2016, 2017, 2018 | 323 |
|  |  | Elkin Creek | 1996, 2010 | 90 |
|  |  | Kuzkwa River | 2007, 2008, 2009, 2012, 2013 | 65 |
|  |  | Nechako River | 1996 | 82 |
|  |  | Pinchi Creek | 2000, 2005, 2008, 2009, 2012 | 26 |
|  |  | Quesnel River | 1996 | 91 |
|  |  | Stuart River | 1996 | 45 |
|  |  | Taseko River | 1998, 2002, 2010 | 78 |
| Middle Fraser River_SP_1.3 (MFR-spring) | 10 | Baezaeko River | 1985 | 14 |
|  |  | Baker Creek | 2008 | 30 |
|  |  | Bridge River | 1996, 2018 | 88 |
|  |  | Cariboo River upper | 2001, 2017 | 97 |
|  |  | Chilako River | 1998 | 14 |
|  |  | Chilcotin River lower | 1996, 2000, 2001 | 92 |
|  |  | Chilcotin River upper | 2001 | 90 |
|  |  | Cottonwood River lower | 2004, 2008 | 64 |
|  |  | Endako_River | 2006, 2007, 2008, 2009 | 103 |
|  |  | Horsefly River | 1997, 2011, 2012, 2013 | 39 |
|  |  | Nazko_River | 1983, 1984, 1985 | 47 |
|  |  | Shovel Creek | 2009 | 25 |
|  |  | West Road (Blackwater) River | 1997, 2007, 2008, 2014 | 79 |
| Middle Fraser River-Portage_FA_1.3 (Portage) | 9 | Portage Creek | 2002, 2005, 2006, 2008, 2017, 2018 | 68 |
| Middle Fraser-Fraser Canyon_SP_1.3 (FR canyon) | 8 | Nahatlatch River | 1991, 2002, 2003, 2007, 2013 | 23 |
| North Thompson_SP_1.3 (NTh-spring) | 18 | Blue River | 2000, 2002, 2003, 2004, 2006, 2007, 2009, 2012, 2013, 2014, 2016, 2017 | 66 |
|  |  | Finn Creek | 1996, 2010, 2013 | 100 |
| North Thompson_SU_1.3 (NTh-summer) | 19 | Barriere River | 2000, 2001, 2002 | 40 |
|  |  | Clearwater River | 1997 | 82 |
|  |  | Lemieux Creek | 2002, 2004, 2008, 2010, 2013 | 52 |
|  |  | North Thompson River | 2001 | 93 |
|  |  | Raft River | 2008, 2009, 2010, 2013 | 84 |
| Shuswap River_SU_0.3 (STh-SHUR) | 15 | Shuswap River lower | 2013, 2014, 2015, 2016, 2017, 2018 | 1,205 |
|  |  | Shuswap River middle | 2013, 2014, 2015, 2016, 2017, 2018 | 596 |
| South Thompson-Bessette Creek_SU_1.2 (STh-BESS) | 16 | Bessette Creek | 2012, 2013, 2014, 2015, 2016 | 74 |
|  |  | Duteau Creek | 2001, 2003, 2006, 2010, 2013 | 43 |
| South Thompson_SU_0.3 (STh-0.3) | 13 | Adams River | 2010 | 98 |
|  |  | Little River | 1996, 2010 | 83 |
|  |  | South Thompson River | 1996 | 76 |
|  |  | Thompson River (below Kamloops Lake) | 2001, 2008 | 97 |
| South Thompson_SU_1.3 (STh-1.3) | 14 | Eagle River | 2010 | 96 |
|  |  | Salmon River | 1996, 1999, 2013, 2018 | 122 |
|  |  | Seymour River | 2002, 2003, 2010 | 34 |
| Lower Thompson_SP_1.2 (LTh) | 17 | Bonaparte River | 1996, 2006 | 46 |
|  |  | Coldwater River | 2013, 2014, 2015, 2018 | 187 |
|  |  | Coldwater River upper | 2001, 2002, 2004, 2005, 2006 | 97 |
|  |  | Deadman River | 1997, 1998, 1999, 2006 | 100 |
|  |  | Louis Creek | 2001, 2006, 2008, 2010, 2013 | 108 |
|  |  | Nicola River | 2013, 2014, 2015, 2017, 2018 | 532 |
|  |  | Spius Creek | 2013, 2014, 2015, 2018 | 177 |
|  |  | Spius Creek upper | 2001, 2002, 2006 | 101 |
| Lower Fraser River_SP_1.3 (LFR-spring) | 4 | Birkenhead River | 2003, 2005, 2006 | 86 |
| Lower Fraser River_SU_1.3 (LFR-summer) | 6 | Big Silver Creek | 1996, 2005, 2006, 2009, 2012 | 83 |
|  |  | Sloquet Creek | 2003, 2004, 2006 | 21 |
| Lower Fraser River-Upper Pitt_SU_1.3 (LFR-spring) | 5 | Blue Creek | 2006, 2007, 2008, 2011, 2012 | 50 |
|  |  | Pitt River upper | 2005, 2010, 2011, 2012 | 54 |
| Maria Slough_SU_0.3 (Maria) | 7 | Maria Slough | 1999, 2001, 2005 | 96 |
| Lower Fraser River_FA_0.3 (LFR-fall) | 3 | Capilano River | 2013, 2015, 2016, 2017, 2018 | 1,945 |
|  |  | Chilliwack River fall | 2018 | 971 |
|  |  | Chilliwack – Vedder River | 2013, 2014, 2015, 2016, 2017, 2018 | 4,613 |
|  |  | Harrison River | 2013, 2014, 2015, 2016, 2017, 2018 | 1,468 |
|  |  | Stave River | 2001, 2002 | 148 |
| East Vancouver Island-North_FA_0.x (NEVI) | 29 | Campbell River | 2014 | 56 |
|  |  | Nimpkish River | 2011, 2016 | 164 |
|  |  | Quatse River | 1996 | 21 |
|  |  | Quinsam River | 2013, 2014, 2015, 2016, 2017, 2018 | 8,432 |
|  |  | Woss Lake | 2001 | 30 |
|  |  | Woss River | 2016 | 59 |
| East Vancouver Island-Qualicum and Puntledge_FA_0.x (QP-fall) | 27 | Little Qualicum River | 2013, 2018 | 399 |
|  |  | Puntledge River fall | 2013, 2014, 2015, 2016, 2017, 2018 | 4,827 |
|  |  | Big Qualicum River | 2013, 2014, 2015, 2016, 2017, 2018 | 8,801 |
| East Vancouver Island-Nanaimo and Chemainus_FA_0.x EVI-fall) | 25 | Chemainus River | 1996, 2015, 2016, 2017 | 71 |
|  |  | Nanaimo River fall | 1998, 2002 | 84 |
| East Vancouver Island-Nanaimo_SP_1.x (NanR-spr) | 23 | Nanaimo River upper | 2004 | 56 |
| East Vancouver Island-Georgia Strait_SU_0.3 (EVI-GStr-sum) | 83 | Nanaimo River spring | 2005 | 95 |
|  |  | Nanaimo River summer | 2018 | 75 |
|  |  | Puntledge_River | 2013, 2014, 2015, 2016, 2017, 2018 | 1,819 |
| East Vancouver Island-Cowichan and Koksilah_FA_0.x (CWCH-KOK) | 22 | Cowichan River | 2013, 2014, 2015, 2016, 2017, 2018 | 2,044 |
| West Vancouver Island-Nootka and Kyuquot_FA_0.x (NoKy) | 32 | Artlish River | 2015 | 36 |
|  |  | Burman River | 1985, 1986, 1989, 1990, 2013, 2014, 2015, 2016 | 828 |
|  |  | Conuma River | 2013, 2014, 2015, 2016 | 897 |
|  |  | Gold River | 2013, 2014, 2015 | 373 |
|  |  | Kaouk River | 2010, 2015 | 152 |
|  |  | Leiner River | 2014, 2015 | 144 |
|  |  | Tahsis River | 2014, 2015 | 85 |
|  |  | Tahsish River | 2015, 2017 | 61 |
|  |  | Tlupana River | 2002, 2003, 2013, 2014 | 102 |
|  |  | Zeballos River | 2006, 2007, 2008, 2009 | 112 |
| West Vancouver Island-North_FA_0.x (NWVI) | 33 | Colonial Creek | 1999, 2004, 2015, 2016 | 69 |
|  |  | Marble River | 2000, 2015 | 91 |
| West Vancouver Island-South_FA_0.x (SWVI) | 31 | Bedwell River | 2007, 2014, 2015, 2016, 2017, 2018 | 121 |
|  |  | Cypre River | 2014, 2015, 2016 | 62 |
|  |  | GORDON_River | 2014 | 34 |
|  |  | Kennedy River lower | 2007, 2015, 2017 | 131 |
|  |  | Megin River | 2003, 2006, 2007, 2015 | 57 |
|  |  | Moyeha River | 2004, 2010, 2011, 2018 | 52 |
|  |  | Nahmint River | 2013, 2014, 2015, 2016, 2017, 2018 | 229 |
|  |  | Nitinat River | 2013, 2014, 2015, 2016, 2018 | 1,830 |
|  |  | Robertson Creek | 2013, 2014, 2015, 2016, 2017, 2018 | 26,092 |
|  |  | San Juan River | 2014, 2015, 2017, 2018 | 348 |
|  |  | Sarita River | 2013, 2014, 2015, 2016, 2017, 2018 | 1,516 |
|  |  | Sooke River | 2014, 2015 | 193 |
|  |  | Stamp River (above falls) | 1973, 2015, 2017 | 79 |
|  |  | Thornton Creek | 2015, 2016, 2018 | 260 |
|  |  | Toquart River | 1999, 2000, 2015, 2017, 2018 | 156 |
|  |  | Tranquil Creek | 1996, 2004, 2014, 2015, 2016, 2017, 2018 | 98 |
| Okanagan_1.x (OK) | 1 | Okanagan River | 2000, 2001, 2002, 2003, 2004, 2005, 2006, 2007, 2008 | 100 |
| Juan de Fuca (JDF) |  | Elwha River fall | 1996 | 96 |
| Coastal Washington (COWA) |  | Forks Creek hatchery | 2005 | 95 |
|  |  | Hoh River | 1995, 1996, 1997 | 57 |
|  |  | Queets River | 1997 | 26 |
|  |  | Quinault River | 1995, 1997 | 52 |
|  |  | Sol Duc River | 1995 | 78 |
| North Puget Sound (NPS) |  | Nooksack River | 1998 | 99 |
|  |  | Skagit River | 1996 | 99 |
|  |  | Snohomish River | 2009, 2010 | 87 |
|  |  | Stillaguamish River | 1996 | 82 |
|  |  | Skykomish River | 1996 | 57 |
| South Puget Sound (SPS) |  | White River | 1994 | 94 |
|  |  | Green River | 1997 | 100 |
|  |  | Green River Kendal hatchery | 1998 | 50 |
|  |  | Soos Creek | 2004 | 94 |
| Lower Columbia River (LCR) |  | Abernathy River | 1995 | 95 |
|  |  | Coweeman River | 1993, 1996 | 57 |
|  |  | Cowlitz Hatchery spring | 2004 | 97 |
| Mid Columbia River_SP (MCR-Sp) |  | John Day River east fork | 2000 | 40 |
|  |  | John Day River main fork | 2000 | 33 |
|  |  | John Day River north fork | 2000 | 39 |
|  |  | Naches River | 1993 | 30 |
|  |  | Spring Creek hatchery | 2001, 2002 | 37 |
| Upper Columbia River_SP (UCR-Sp) |  | Chewuck River | 1993 | 88 |
|  |  | Chiwawa River spring | 1993 | 95 |
|  |  | Entiat River | 2002 | 64 |
|  |  | Twisp River | 1995 | 89 |
| Upper Columbia River_SU_FA (UCR-SuF) |  | Deschutes River | 1998 | 80 |
|  |  | Hanford Reach | 2004, 2006 | 93 |
|  |  | Osoyoos LAKE | 2003, 2009 | 21 |
|  |  | Similkameen River | 2006 | 72 |
|  |  | Wenatchee River | 1993 | 91 |
| Snake River_FA (SR-F) |  | Lyon’s Ferry | 1998, 2003 | 114 |
| Snake River_SP_SU (SR-SpSu) |  | Frenchman Creek | 1991, 1992 | 46 |
|  |  | Imnaha Creek | 2002, 2003 | 89 |
|  |  | Johnson Creek | 2001 | 86 |
|  |  | Marsh Creek | 1989, 1991, 1998 | 104 |
|  |  | McCall Creek | 1997 | 32 |
|  |  | McCall hatchery | 1989 | 24 |
|  |  | Minam reek | 1994, 2002 | 91 |
|  |  | Rapid River | 1997, 1999 | 93 |
|  |  | Salmon River east fork | 1999 | 40 |
|  |  | Salmon River upper | 1989, 1992, 1993 | 125 |
|  |  | Secech River | 2002, 2003 | 90 |
|  |  | Tucannon River | 1995 | 86 |
|  |  | Upper Valley Creek | 1998 | 64 |
|  |  | Valley Creek | 1989 | 14 |
|  |  | Wenaha River | 1998 | 40 |
| North & Central Oregon (NCOR) |  | Cle Elum hatchery | 2004 | 68 |
|  |  | Euchre Creek | 1996 | 55 |
|  |  | Nehalem River | 1996 | 53 |
|  |  | Siuslaw River | 2011 | 54 |
|  |  | Smith River –Umpqua | 1997, 1998 | 71 |
|  |  | Trask hatchery | 1997 | 85 |
| Upper Willamette River (UWR) |  | Clackamas River | 1997, 1999 | 145 |
|  |  | Sandy River | 1997 | 64 |
|  |  | Santiam River north | 1997 | 83 |
| South Oregon coastal (SOR) |  | Cole River | 1995 | 42 |
|  |  | Hunter Creek | 1995 | 94 |
|  |  | Lobster Creek | 1998 | 47 |
|  |  | Nestucca River fall | 2004, 2005 | 91 |
|  |  | Pistol River | 1995 | 64 |
|  |  | Umpqua spring | 2004 | 84 |
|  |  | Winchuk River | 1995 | 80 |
| California Klamath Trinity (CAKT) |  | Blue Creek | 1999 | 92 |
|  |  | Salmon River spring | 1998 | 26 |
|  |  | Trinity River hatchery fall | 1998 | 86 |
| California Central Valley_Fall (CACV-F) |  | American River | 1999 | 44 |
|  |  | Battle Creek | 1999 | 34 |
|  |  | Butte Creek fall | 2000 | 48 |
|  |  | Feather River fall | 1999, 2000 | 54 |
|  |  | Merced River | 1998, 1999 | 81 |
|  |  | Sacramento River fall | 1995 | 35 |
|  |  | Stanislaus River | 1998 | 25 |
|  |  | Toulumne River | 1998 | 27 |
|  |  | Yuba River fall | 2000 | 35 |
| California Central Valley_Spring (CACV-Sp) |  | Butte Creek | 2002, 2003 | 74 |
|  |  | Feather River spring | 1999, 2000 | 77 |
|  |  | Yuba River spring | 2000 | 26 |
| Coastal California (CACO) |  | Eel River fall | 2000 | 88 |
| Total |  |  |  | 105,722 |

|  |  |  |  |  |
| --- | --- | --- | --- | --- |
|  |  |  |  |  |
|  |  |  |  |  |

Supplementary Table S2. Loci, amplicon forward and reverse primer sequences, orientation of forward primer (Or), chromosome (Chrom), position in genome, nucleotides observed at SNP position (positive strand of genome)(Allele), expected heterozygosity (He), Fst value, and potential for multiple mapping positions (Multi, Y=yes).

| Locus | Forward primer | Reverse primer | Or | Chrom | Position | Allele | He | Fst | Multi |
| --- | --- | --- | --- | --- | --- | --- | --- | --- | --- |
| Oki_RAD100331-77 | GTGACTACGCCACCCAAATAAC | TGTCAAAGAGAAACACATCAGAACAGG | - | NC_037101.1 | 2849628 | T/A | 0.173 | 0.111 |  |
| Oki_RAD101032-83 | TGGAGACTGGCGAGTCCAATA | ACATCCATACGACAGAAATATTGGCAAA | + | NC_037116.1 | 45012302 | A/T | 0.140 | 0.150 |  |
| Oki_RAD102175-139 | CCTGGGCTGGTGTGGTTAAATGTGA | CTCAAGTTTTGAGAGAGTTTTCAATTGGCAT | - | NC_037115.1 | 20937561 | G/A | 0.282 | 0.090 |  |
| Oki_RAD104180-36 | GCAGGAAGCATATAGGCTGGGA | GTTCAGATCCAAACCACATGTACAGC | - | NC_037105.1 | 75398426 | T/A | 0.491 | 0.157 |  |
| Oki_RAD107732-76 | CAACTCCCGATGGACGTCCTCA | GAGGCTACGCGGGACCACTTC | + | NC_037103.1 | 55350388 | C/A | 0.500 | 0.146 |  |
| Oki_RAD109706-184 | TAGCTGTATGTGTACATGGAGGAAA | ACTCATCGCCAGGATGAACTTG | + | NC_037112.1 | 55756317 | G/A | 0.006 | 0.031 |  |
| Oki_RAD112339-36 | GCCCTCGTTGCCCGAGA | TGTGGACTTTCCATGAAAAATCAAGCATATC | + | NW_020142537.1 | 171765 | G/A | 0.036 | 0.072 | Y |
| Oki_RAD115799-173 | ACATCCTCGCACAGGCTGCAT | AGGAAGGGAGAGGGTGTCCT | - | NC_037108.1 | 24028789 | A/C | 0.112 | 0.143 |  |
| Oki_RAD14549-101 | GCTTTTCACAAGAACCTGCTGTT | CTTTGAGAGACATATCCAACTCCTCCT | - | NC_037129.1 | 28526694 | C/A | 0.199 | 0.068 |  |
| Oki_RAD24617-78 | GTTTTCATTTCCCACAGCGAGGT | GGGATTACCTCCAGATGTCTTTAGTGAT | N/A | N/A | 31* | T/G | 0.323 | 0.040 |  |
| Oki_RAD29294-129 | TGCAGGAAATTAGCTTTCAAACTGGAA | GTTTCGATTTACTAAAATGACGTGGAACTCTAC | + | NC_037099.1 | 79343247 | C/T | 0.181 | 0.335 |  |
| Oki_RAD39712-122 | TGTGGTGGAGCTGGTCCAAGC | ACCCTGCCTGTCCTGACCTCTA | - | NC_037105.1 | 6353785 | G/A | 0.442 | 0.121 |  |
| Oki_RAD42227-59 | GCCAACAGAGGGTCAAGTCAAT | TGCCTTTCCCCAGCTATTCTAGAT | - | NC_037100.1 | 30837246 | G/A | 0.470 | 0.088 |  |
| Oki_RAD42296-114 | TGAAGGGTGTTTCTTTCGACAC | AAAAAGTTGTTAATTTGTTGTTGTCTTTTACCC | + | NC_037107.1 | 5215488 | C/T | 0.202 | 0.067 |  |
| Oki_RAD42662-170 | GACTTCTGCCCTCAGTTGAACG | TTTCGTGATATCAAATTGCGATCAAATTATG | - | NC_037121.1 | 1973441 | T/C | 0.399 | 0.085 | Y |
| Oki_RAD43051-180 | TGCAGGACGTTGGTAAAGC | CATAAAAGAGGCGCTAACATTGTAAATGCA | + | NC_037113.1 | 19803773 | G/C | 0.499 | 0.249 |  |
| Oki_RAD45746-124 | CATGAGGAAGGAGTATGTCTTCACCT | TCGCGATTATGTCTCACCAAGT | - | NC_037101.1 | 53478115 | C/T | 0.040 | 0.091 |  |
| Oki_RAD46141-53 | TGCAGGTAGAACATACCCTGAAAAAT | CCCTGATCCAGTGGAAACATAAAATAAG | - | NC_037104.1 | 19877069 | G/A | 0.326 | 0.076 |  |
| Oki_RAD49488-134 | TGCAGGTAACTGATGGAAACATTTG | GTCATCCAATAGCCATGGTAGCCAAAATAATA | - | NC_037105.1 | 35701400 | T/A | 0.500 | 0.175 |  |
| Oki_RAD49991-192 | AGGAGGTGTCAAGTCCTGAGTATT | CTTGAAAAAGTCACCAGTTCGTCAAATA | - | NC_037104.1 | 33452255 | C/T | 0.478 | 0.096 |  |
| Oki_RAD53655-150 | CAGCGTTGGTGAAGGTTTCAGAG | CATGGTCAGCAAACGTTTGATACCT | - | NC_037097.1 | 21916034 | A/T | 0.141 | 0.089 |  |
| Oki_RAD53705-53 | CCACAAGGAGTCGCTAACCC | TTTAAATCTGACCAAACAGACCTCCCT | - | NC_037107.1 | 40312224 | T/G | 0.068 | 0.065 |  |
| Oki_RAD55090-126 | CTCTATGGTGCGGAGCATTGG | TGTGTTTACATAAGAGGATAAGAGTTGAGCA | - | NC_037124.1 | 3927427 | C/T | 0.340 | 0.043 |  |
| Oki_RAD57956-155 | GCATGCAATGACATCAATCCAGACCTACT | CGTGATCAAAGGTGTGGTCATCTTTCAAAAT | - | NC_037110.1 | 7042377 | C/G | 0.107 | 0.039 |  |
| Oki_RAD60246-129 | CCAGGGTACGCTGTAGGACTT | AGCAAATCCAATAGCAATCTTGGC | + | NC_037104.1 | 69074951 | T/C | 0.401 | 0.049 |  |
| Oki_RAD63267-39 | CTCATCCTGACATGACCCTCCATGAC | GCACCTGCAAGTTCCCGGAATG | - | NC_037128.1 | 10317153 | T/C | 0.006 | 0.006 |  |
| Oki_RAD66324-122 | CAGGATCGTCTGAGATGAGCCACCT | CTCCAGCATGCCAGTGGTGAG | - | NC_037127.1 | 24678193 | A/G | 0.314 | 0.092 |  |
| Oki_RAD68033-114 | TGCAGGGATGTTGCCACTC | AGAAGAGACACCACACTCGACTGTT | - | NC_037109.1 | 6542518 | A/C | 0.143 | 0.111 |  |
| Oki_RAD70600-167 | CCTACCACCAACAGTGCAAGAT | GCTGTCACTGTGTATTACATGAGATAGA | - | NC_037126.1 | 27037661 | C/A | 0.111 | 0.055 | Y |
| Oki_RAD72326-154 | TATTGTCTCCCAGCGTAGGCA | CAGTGAGTTAACTGTTATATGGAGGCA | - | NC_037119.1 | 19388146 | C/G | 0.362 | 0.040 |  |
| Oki_RAD72503-47 | AGGAGCGGGTCCCTGCTTTGATAT | CACCCAAATGGCGACCGCATC | + | NC_037109.1 | 44009806 | G/T | 0.332 | 0.095 |  |
| Oki_RAD73094-109 | CTACACATGAAGCGCCACCAG | GATGTGTTGGTGGTTGGGTGTTTATG | + | NC_037115.1 | 12942279 | G/C | 0.260 | 0.080 |  |
| Oki_RAD78773-118 | GCCAATCAGCAGTCTACTTACATGAT | CCCACAGTCAACGCTATCTCTTTAC | - | NC_037100.1 | 9339817 | A/T | 0.059 | 0.136 |  |
| Oki_RAD78988-46 | CAACACACCCCCTCCGCTCAAT | ATTAGAGTTCGTAATCCGGCCCTTCAC | - | NC_037102.1 | 58335781 | T/C | 0.345 | 0.047 |  |
| Oki_RAD90772-47 | TGCAGGTATGACTTTCCAAATGC | ATCTGCATATATCACTACGTCATCCAT | + | NC_037099.1 | 12490877 | T/C | 0.128 | 0.066 |  |
| Oki_RAD92875-166 | GCAGGCCAAGAGGGTGTC | GCAGCCCAGATGCTCTTTGCAG | - | NC_037119.1 | 6535542 | C/G | 0.339 | 0.114 |  |
| Oki_RAD93028-94 | GAGGCTGGTGTGTAACCTGTC | TGGAGCAGTGAGATTCAAACATGTC | + | NC_037098.1 | 30211856 | C/A | 0.081 | 0.064 |  |
| Oki_RAD94260-197 | ACCTTGATGCAAGGGTAAGACATT | AAGAGACACAACACCCTTCATTATATCA | + | NC_037130.1 | 1331387 | G/C | 0.491 | 0.199 |  |
| Oki_RAD95780-166 | AGGCTGACCTCGGTCATCAGGTAA | CCCCCTTTTTCTCCCTAATTTTGATATTTTCTC | - | NC_037100.1 | 8152104 | G/A | 0.398 | 0.070 |  |
| Oki_RAD99813-123 | CAGCAGGGCCAAACTGTCTCG | TTCTCTGCTCTCTTTTATTCATTTATCCGTTGA | - | NC_037098.1 | 898160 | C/T | 0.106 | 0.220 |  |
| Oki10077183-41 | GGTCCAAACTGCAGGACTACAAAA | AGTTTATTTTGGACAGGTCCCTCCT | + | NC_037100.1 | 41119849 | G/A | 0.011 | 0.064 |  |
| Oki10451599-73 | CCCAGCCTTAATTCCACACTACG | AAGACATGGAGGTACAATGTGGCTA | - | NC_037110.1 | 21219456 | T/G | 0.013 | 0.039 |  |
| Oki104569261-312 | GATGTTGCCAATCTTGGCCTTCTTA | CCCTCCCCCTTAAGGTCATTCA | + | NC_037111.1 | 23086003 | G/A | 0.472 | 0.086 |  |
| Oki10797446-61 | GCAGACCTCGAACTGAATCTTCTTT | GCTAACTTTGGCACTGAGACACTATC | + | NC_037118.1 | 10045389 | G/T | 0.451 | 0.053 |  |
| Oki109651152-177 | CTACCTTTTAAAACGGTATAAGATACACGTATG | CTAACATGGATCCTTTGTCCTGATTTTGC | + | NC_037103.1 | 20705199 | G/A | 0.004 | 0.014 |  |
| Oki109874122-233 | TGGGTAGCTAAACATGAATAGGCA | CCGTTATAATAAGCCTCACGGTTATGAA | - | NC_037118.1 | 15609078 | G/A | 0.065 | 0.089 | Y |
| Oki110078191-224 | GAGGGAGAATACGAAAGGTGGTTG | GATTGGCTGCAGAGTTACTGCTAA | - | NC_037110.1 | 3568634 | A/C | 0.165 | 0.136 |  |
| Oki116362411-478 | TCAAGAAGGTGAGCCAGTGTAC | TTGTCACACATTTGGCAAGACAGT | - | NC_037118.1 | 32429381 | G/A | 0.163 | 0.061 |  |
| Oki117815369-247 | CCCATGATGCAATGGTAGGACT | GCTTCACTAGAGGTACAGCAAGC | + | NC_037099.1 | 64762862 | T/C | 0.084 | 0.148 | Y |
| Oki118152314-274 | TTGATGTGAATCGGCTGTACTCC | CACCTCAATACCTGCAAAGGAAAG | + | NC_037104.1 | 9463690 | C/T | 0.475 | 0.176 |  |
| Oki13029548-108 | ACATGAGCACAAATATAAATGTGGCTGTTA | GACAGAATGTTCTACAAGTTGCAACTTC | - | NW_020128861.1 | 484563 | G/T | 0.370 | 0.116 |  |
| Oki97954228-165 | TGGAATTTTACACCCATGATATCCATCT | AATACAGACGTGTTTCACACACTGG | - | NC_037120.1 | 8543754 | G/T | 0.037 | 0.049 |  |
| Okianp168-224 | CTTTGCCAATAACAGCACATTTTCAGA | CCCAGTCAATGTGCCTCTATGATTTC | + | NC_037118.1 | 3745953 | G/T | 0.182 | 0.070 | Y |
| Okicarban140-96 | CCTACAGTCATTGACCTGCTCATT | GAATGCAAAGTCATTGTCATGACAGA | + | NW_020142510.1 | 12661222 | A/G | 0.148 | 0.074 | Y |
| Okinips159-71 | CAGCAATGACATCAACATGCTTGAC | TGTCTAGCAGTATGGGAGAGTCAC | + | NC_037113.1 | 3556482 | G/T | 0.419 | 0.086 |  |
| OkiOts_120255_113 | TGGAGTTGACAAAACATCCGATGTC | CCAGCAGACAGTCATCCTAAAAGAAA | - | NC_037101.1 | 38435724 | T/C | - | - | Y |
| Okirpo2j235-149 | GAATGGCGTTTTGAGTGAGTTTTCA | TTCAGCACATACCTAACAAACTACAGTA | - | NC_037105.1 | 35255211 | T/A | 0.141 | 0.069 |  |
| OkiSClkF2R2120-130 | CCTCCTTTTAACACAAGACTGTTGAAGA | ACACAAAACAAACAAACATTCTCACATG | + | NC_037101.1 | 59903985 | C/T | 0.215 | 0.091 |  |
| OkiSWS1op38-99 | GACTGGGATGTCAGCTGCTATAA | CTTCGAACTGTCTCAAAGCAAGTGATAT | + | NC_037103.1 | 64449728 | G/A | 0.394 | 0.175 |  |
| Ots_100884-287 | CCAGATTCTCCAAGAGTATCAGATAGAAC | TGCAGGAATACAAGGATAGATTGAGG | - | NC_037099.1 | 72097697 | C/T | 0.422 | 0.070 |  |
| Ots_101119-381 | GGAGTAAACAAAACCAGGACACTGT | ACCTGAAAGGTCTAGATGGATTGTTTTA | - | NC_037127.1 | 21772790 | C/T | 0.029 | 0.122 |  |
| Ots_101554-407 | CCTGTATTTCTCCTGTATGTGCATC | AGTGATGTCTCCTTTTAAAAGTTCCAC | - | NC_037117.1 | 12050017 | C/G | 0.424 | 0.177 |  |
| Ots_101704-143 | GAGACCTGTGGTAAACAACTTCTTGA | ACAGCATCTCTGTCAGTTAAACACA | + | NC_037104.1 | 21039383 | A/C | 0.418 | 0.185 |  |
| Ots_102420-494 | AAACAGGGCTACATCATGATGGC | TTGGGTGTAGTCATTGATATGTGGTTTA | - | NC_037099.1 | 14951461 | T/G | 0.347 | 0.124 |  |
| Ots_102457-603 | AACACCGTGCAAACCTTGGTTATC | AGTGGTTAAGGGCGCTGTACT | + | NC_037105.1 | 26992696 | T/C | 0.469 | 0.114 |  |
| Ots_102801-308 | ACTATCATAGTCTGACAGACCCCTT | CAGAATCTCTCCATGATATGCCAATGTG | - | NC_037111.1 | 19904397 | C/A | 0.213 | 0.053 |  |
| Ots_103041-52 | CCCCATCGACATCACCACCCA | AGAGGCCTGTCACTGTCATTACC | - | NC_037104.1 | 15518130 | A/G | 0.392 | 0.157 |  |
| Ots_103122-180 | ATCCATTCCCTGACAATACATTTCACAC | TGGTTGTAAAGGAAAATGAAGGACAAGG | - | NC_037100.1 | 22443878 | C/T | 0.495 | 0.283 |  |
| Ots_104048-194 | CGCTGGTGAAGAAGCTGAGAAAA | TTGTGCTACTCTGTAGCTTTTGTTC | - | NC_037123.1 | 7061854 | C/T | 0.212 | 0.051 |  |
| Ots_104063-743 | GTAATATGTGTACAGCTGACGTGTG | GGAAGGGTCATGGGTGTCCTT | + | NC_037111.1 | 2747251 | G/A | 0.353 | 0.070 |  |
| Ots_104216-527 | TTGCTTGCTGTTCATAACCCGTT | GGAAAAGTCGGGTGAGAAGCAAA | - | NC_037102.1 | 61018092 | T/G | 0.014 | 0.155 |  |
| Ots_104415-347 | TTTCGGCGTACCCTACCAATACT | CTGGTGTTGCTAGAAAGAGTACCC | - | NC_037113.1 | 5993649 | C/T | 0.440 | 0.072 |  |
| Ots_105105-613 | CGCAGTACAAGTGCAGAGAATGAC | AACCACATAGGATTTTATTTGAAGAGAAAACA | + | NC_037103.1 | 77202120 | G/C | 0.356 | 0.187 |  |
| Ots_105401-325 | ATTTGACCAGTTTGCCACCTCAA | TTCGATGTAAACACATGTCCAAGTGA | - | NC_037100.1 | 18305267 | T/G | 0.077 | 0.072 | Y |
| Ots_105897-124 | ATCTTTGGCAACAGGGACACATT | TGTTTAAAGTGACTTCAAAGAACACCAA | - | NC_037099.1 | 45479613 | C/T | 0.032 | 0.098 | Y |
| Ots_106313-729 | ACATCATTTGCTGCAGAGTCAAAAC | CAGGAGGATGCTTATGTGCAGATAC | + | NC_037107.1 | 16517833 | T/C | 0.243 | 0.122 |  |
| Ots_106419b-618 | TGCAATTAGCAATTAGTGCCTTGCC | CCCCTCCAACCGGAAGTCCAG | - | NC_037109.1 | 11736968 | T/G | 0.500 | 0.223 |  |
| Ots_106747-239 | CATGCATCAGCCCAGACTTGC | CCATTCTACTCCAACCACACCATGTA | - | NC_037120.1 | 9568130 | C/A | 0.406 | 0.052 |  |
| Ots_107074-116 | CCATCTTTTCCATGGCTGTGTGTA | CTCCTGTAAGGCTGCTACGG | - | NC_037121.1 | 8142848 | T/A | 0.497 | 0.149 |  |
| Ots_107285-93 | GTTCATAATCCGCTTCGCTGAATC | ATACATTTCAAGCAACGTGATCAAACTG | + | NC_037100.1 | 6690909 | A/T | 0.318 | 0.089 |  |
| Ots_107806-821 | GACAAGTCCTTGGAGTGATTGGATAC | AATCAAATTTGCAGTGCTGAATTAGAGA | + | NC_037097.1 | 56999741 | A/T | 0.421 | 0.077 |  |
| Ots_108007-227 | GAAAACAAAAGTGTTTCTCTCATTGGACAA | TGATTCTTAATGGATAAGGCCAGGTTC | - | NC_037106.1 | 25247839 | T/A | 0.428 | 0.124 |  |
| Ots_108390-329 | GTTGTGAAAAGAATGGAGGTTTGTTACT | TTTCCCCTAACAAAACATTTATCGACAC | + | NC_037125.1 | 19105883 | G/C | 0.282 | 0.175 | Y |
| Ots_108820-336 | ATTCCTTTGGTTGGTTGTTTTTATCCTT | ACAAAAACACTGTGATACAACGACACAC | - | NC_037124.1 | 12014613 | A/G | 0.388 | 0.152 |  |
| Ots_109693-392 | TGACATGATAATTACAAGGGACTCTCTG | GCATTAGGTGAGGCCTTTCTGG | - | NC_037127.1 | 9369378 | T/G | 0.247 | 0.081 |  |
| Ots_110381-498 | CCATCGAGGGTCGCCTATC | GGCCCAGAACTCAAAAATGTTTGTC | - | NC_037115.1 | 36960028 | A/G | 0.494 | 0.138 |  |
| Ots_110495-446 | ATGGTCGTATGAAAAGTTCCGCAT | TCAGCTAATTCTGCTGCACTAATGTTAT | + | NC_037106.1 | 41631468 | G/C | 0.387 | 0.083 |  |
| Ots_110551-64 | CTTTGGTTGGGAAGAGGGAAAGATT | TCATTGTTTGCTCTGGATTGTAGAACTG | - | NW_020142581.1 | 1270104 | C/A | 0.320 | 0.065 | Y |
| Ots_110689-218 | AAACTAGAGTCCAGTGTTATGTTAATGTCT | TAGGCCATAATATTCTGTTTCCCTCTCT | - | NW_020129679.1 | 353587 | T/G | 0.387 | 0.025 | Y |
| Ots_111666-257 | CTATGCTTATACAATTTTGGGTTTGAATGT | CCAGCTCGAACTGATCGTTAATGTTAG | - | NC_037105.1 | 83075472 | C/T | 0.497 | 0.152 |  |
| Ots_111681-77 | CCCGTCGTACCAAGACACCAT | CTGTGCATCATGCACCATTACAATGTAG | + | NC_037101.1 | 9439742 | A/C | 0.444 | 0.078 |  |
| Ots_112208-722 | GTCAGTCAGCATCTACGTCCAAC | GATCAGGCTGGGCTAATTTTTGCTA | - | NC_037111.1 | 13348552 | C/A | 0.496 | 0.166 |  |
| Ots_112301-43 | AAAGCATGGCTGCCCTAGAAC | CTTTACTCTTCCTTCTCTCTGCATTCA | - | NC_037099.1 | 33896915 | C/T | 0.407 | 0.114 |  |
| Ots_112820-111 | GAAGCAAGCAATGGGTAATGAGAA | AGAGTTAGCAGTGGTATGGTGGTA | + | NC_037106.1 | 18438064 | G/A | 0.460 | 0.066 |  |
| Ots_115987-325 | CATTTCTTCTTGGATGGATTGGTCAC | TAGATACTCAAGTTATCTGCTGGCCATA | - | NC_037102.1 | 66387317 | T/G | 0.500 | 0.132 |  |
| Ots_117242-340 | CTGGTCCTCCCTGTCTCTATCTACTA | ATGAAAGAGAAAGACATAAAGAGAAAGGGA | + | NC_037112.1 | 29941952 | T/C | 0.313 | 0.149 |  |
| Ots_117259-271 | CACAGAGGAAAGTGGATGGGCTA | CTCCACTGCCCCTCCATGAAA | - | NC_037112.1 | 11947135 | T/G | 0.499 | 0.202 |  |
| Ots_117370-471 | AGAGGAAAGGGATTTCTCTGTTCATT | GAAGGTACAAACCAAGGCTTTACTCAG | - | NC_037120.1 | 12157474 | T/G | 0.062 | 0.079 |  |
| Ots_118175-68 | CCTTACGTCCTAGGTAGGAAACAACTTA | AAGTATGCACTCCTGCTTTTAGGG | - | NC_037128.1 | 7631574 | C/T | 0.341 | 0.138 |  |
| Ots_118938-282 | AATTAAGGCACAGTGCCAGTGTAG | CAATATTCCATGGAAACCAACCAAAGT | - | NC_037099.1 | 48430243 | C/T | 0.484 | 0.090 |  |
| Ots_120950-417 | AGGGAACAGACAGGTCACCATC | AGCTTTAATAGCCACAATAATCTGAGTG | - | NC_037101.1 | 31561077 | T/A | 0.434 | 0.118 |  |
| Ots_122414-368 | CTCAGGAAAACCTGTTTGGTTTGT | AGAAGGCCGGGTCGGATG | + | NC_037124.1 | 28489780 | G/A | 0.404 | 0.104 |  |
| Ots_123048-288 | AACTAGCTGAGGTCAATTTAACTTGACA | AGGCCCAGTGTAAGTTATAGACCCTTAA | + | NC_037118.1 | 9478478 | G/T | 0.406 | 0.098 |  |
| Ots_124774-477 | TTGTGTTTTTATTCCATTCCAATAGTACCA | GGAATATCGCCATAGAGATACAGAACG | + | NC_037111.1 | 10921418 | G/A | 0.431 | 0.074 |  |
| Ots_127236-148 | GGAGAACTTGCACTGAATGTGAAAGTA | ACCACCATAAAGTATGTCCACTTGAATA | - | NC_037101.1 | 61557992 | T/A | 0.063 | 0.266 |  |
| Ots_127760-194 | TGACGTTATAGAGGATAGTTTGGAGGAA | GTTGCATTTCGACCAAAAGTCCTAT | + | NC_037097.1 | 70642724 | G/A | 0.224 | 0.314 |  |
| Ots_128757-368 | TATCTCCGAAGACTAGATGGATTCCATT | CCTGGTAACACACGCCTCAAT | - | NC_037103.1 | 6643830 | A/G | 0.330 | 0.181 |  |
| Ots_129144-472 | GCTGGTTCTTGATTCTGTACACTGT | CACAGATACGAACCACCAGAGTG | - | NC_037106.1 | 30541697 | C/A | 0.142 | 0.075 |  |
| Ots_129170-683 | TGTGGATTTCATTCGGGCACTC | TGAGTCATGGTTTCTAACAGCTAGG | + | NC_037104.1 | 47390818 | G/T | 0.239 | 0.067 |  |
| Ots_129458-451 | AATACTTTTCTGGTGATTGCCTGCT | AACAGAATAGTGACAAACCTATGGACAG | - | NC_037097.1 | 15863134 | C/T | 0.266 | 0.161 |  |
| Ots_130720-444 | GCAGGTTACGTATACCTTCCCTCATA | TTACATCTCTGGAACAATTACGGTCA | - | NC_037126.1 | 24569258 | A/G | 0.499 | 0.117 |  |
| Ots_131460-584 | GGTCATAGTGAATGGGATAGCTATCAA | ACAATGAGGGAGCTCATACATTCAGA | - | NC_037103.1 | 42945148 | C/T | 0.368 | 0.103 |  |
| Ots_131802-393 | CTGGACTATAGGAAAGATGTACGTTAAGAA | CATGTGCTTAGCTGCTATGTTTTGTTTA | + | NC_037103.1 | 35733706 | G/A | 0.167 | 0.058 |  |
| Ots_131906-268 | CACAGAAGCACCACAATCAAGTT | CATGTTTTGTCATACTCATGGTATAGGG | - | NC_037108.1 | 21773920 | T/A | 0.380 | 0.070 |  |
| Ots_94857-232 | CCATTTCATAGATAGAGCGATGGACTAC | CGTGAGAATATCATATCGTGAGCCCTA | - | NC_037099.1 | 26623024 | C/T | 0.421 | 0.046 | Y |
| Ots_94903-218 | TGACCCGACATATGTGAATGCG | AGGCATTTATTCCAGTCCTGTTTTG | + | NC_037102.1 | 36076090 | A/C | 0.478 | 0.093 |  |
| Ots_96222-136 | ATTGGCGCAACATATGTATTAAGCA | TGGAGCATCAATTATCGTGATAGTGTA | - | NC_037125.1 | 23142347 | C/T | 0.469 | 0.087 |  |
| Ots_96500-136 | CTGTGACAAAATATACAGGTCTGGTCT | GAGCTGTCTGTCTTTTTAATCACTTGC | - | NC_037100.1 | 53468710 | T/G | 0.495 | 0.128 |  |
| Ots_96899-357 | CAGAAACCTGTCCCTTCCAACTG | GCTGGCTAAAAATCCCATTAGTGAATTA | - | NC_037108.1 | 67888781 | T/A | 0.150 | 0.154 |  |
| Ots_97077-111 | ATACAAAATGACTCTGGGATTCAGATGT | CCTGTAAGATTACAAACCAGCAGTACAC | - | NC_037103.1 | 70085972 | T/G | 0.116 | 0.106 |  |
| Ots_98409-223 | ACACAACAACAAGGAAGTTCTTTATTTGAC | GAGACTCTGCGTTTCTGGAATGT | + | NC_037106.1 | 51246592 | G/A | 0.130 | 0.191 |  |
| Ots_99550-153 | AACAGAAACAAGACTTACAATTGCAACC | CTTGGAAGCTCAACTTCTAGCTCAT | - | NW_020129190.1 | 241292 | C/T | 0.409 | 0.100 |  |
| Ots_aldb_177M-177 | CATGTATATTTAGTTGCGATCAGGTGAC | CGTAGGAGAAGGAGAGCTTCCAA | - | NC_037104.1 | 16272491 | T/A | 0.396 | 0.116 |  |
| Ots_AldB1-348 | GAGAACAAGGCCTGCAAGAATGA | GTCCACAGGTGAGTAGGCCAAA | + | NC_037117.1 | 6480172 | G/A | 0.193 | 0.087 |  |
| Ots_AldoB4-183 | TTATCACATGTGGACACAGTATATGGAC | TGTGCATGCCATGAGAACTTTGT | + | NC_037117.1 | 6477138 | A/T | 0.106 | 0.057 |  |
| Ots_apoc1-857 | GCTCTGTCTTGTCTGGTGACG | TCAGCTCATTGTGTTCCAGGTTATT | + | NC_037099.1 | 5756354 | G/A | 0.247 | 0.076 |  |
| Ots_ARNT-950 | CATCTCTACAACAAATCCACTGGCT | GAGAAAGCGAGTTAATCAGGGAAAAGAG | + | NW_020129131.1 | 53224 | C/A | 0.447 | 0.174 |  |
| Ots_arp-436 | CCTGGAGAAGTACGTTTTAAACTAACAT | CAACCTGTGAAGACAGCAACCA | - | NC_037103.1 | 65259188 | T/A | 0.227 | 0.055 |  |
| Ots_aspat-196 | GGCAGCATGTCATCGACCAAATA | ACTTAATTCCAACTGATGAATATGACCAAC | + | NC_037108.1 | 68043508 | C/G | 0.252 | 0.172 |  |
| Ots_Cath_D141-147 | AGTAGAACTACAACCAGTTGGCTAC | CACACATGGATTTTGCCTGTCTAAAA | + | NC_037121.1 | 35379188 | G/A | 0.061 | 0.039 |  |
| Ots_CirpA-121 | AGTGCTCCCACTTAGCATTCCTA | ATCAGTAGCGACATGTTTCCATTTAATC | + | NC_037101.1 | 56708050 | G/A | 0.500 | 0.133 |  |
| Ots_CRB211-201 | TATGATCGGCTAAAACAGTAACAATTGC | TTTGAAGTTTCTTACCACCACCTTGA | - | NC_037129.1 | 28420240 | C/A | 0.013 | 0.026 |  |
| Ots_crRAD11620-55 | TTATGGGATAGAACAGGAGCTTAAACAG | TTTACGATATAAACTGAGTAGTGTCTTGGT | - | NC_037098.1 | 36061414 | G/A | 0.333 | 0.405 |  |
| Ots_crRAD117-35 | CAGGTTCATGCTCTACAACATTTGTG | GGGTGATGTTGCATTATTAAAGATACTCT | - | NC_037101.1 | 81352698 | C/T | 0.378 | 0.182 |  |
| Ots_crRAD12037-39 | TGCAGGAACTTGCTATGCTTCTAT | GTGTGTAGATTGATGAGGAACAACTTTCA | - | NC_037114.1 | 9770315 | T/C | 0.490 | 0.061 |  |
| Ots_crRAD12711-50 | GGCTGTTATAGCGCGGACTC | GGAAGAAAGCCGTGACATTGACT | - | NC_037102.1 | 13429979 | C/T | 0.247 | 0.093 |  |
| Ots_crRAD13725-51 | CAGGGATCCTCAGTGTTTGCAG | AAGTAGTTATTATGCGGTATTGACCTGT | + | NC_037102.1 | 12958481 | C/A | 0.398 | 0.316 |  |
| Ots_crRAD15076-99 | GAGCTGCAAAGCTCCACATCA | ACACCAAACGTGTTTGCCTATTTTC | + | NC_037102.1 | 43054946 | T/G | 0.112 | 0.079 |  |
| Ots_crRAD16110-38 | TGGTGATCTGAGCTAGGTCTACG | CGGCTAGACCTAAGGATCGTTCA | + | NC_037115.1 | 38916636 | C/T | 0.234 | 0.119 |  |
| Ots_crRAD16540-50 | GATGTGTATTCGTCGACCGGAT | CTTTTCTGCTCACGCATTCTCAGTA | - | NC_037113.1 | 11570673 | A/G | 0.431 | 0.088 |  |
| Ots_crRAD17324-46 | TCAAGGAGTCGCTAGAGCACAATAA | GCGAACCGGACGAATGTGC | - | NC_037097.1 | 3522286 | G/A | 0.478 | 0.093 |  |
| Ots_crRAD17527-62 | CAGGTTGCCGCTGGATTTATTG | GCGGAAGTAGTGAAGGTAGACAATG | + | NC_037109.1 | 36124810 | C/T | 0.395 | 0.074 |  |
| Ots_crRAD18336-118 | AGAAATTGAGAGATTTTATGAACCATTGTCACA | GGGATATATTATTGTGGCACAAAATCAAAAGT | + | NC_037108.1 | 31040214 | C/A | 0.082 | 0.067 |  |
| Ots_crRAD18492-65 | GTTTGTCAGACGAATCATTGGGTCTA | TGAATATTTTCATTTTCCATGCTCGTCTCA | + | NC_037110.1 | 43620612 | C/T | 0.059 | 0.080 |  |
| Ots_crRAD18937-67 | AGGAGTTGAACTGGAGCAGGTA | ACATTCAATATGCACAAACACATCAAGT | - | NC_037106.1 | 50635181 | C/T | 0.280 | 0.164 |  |
| Ots_crRAD20262-155 | CAGCCTCTGCTGAGTTTGAG | TCAGTCTCTAATAGACCGTCTGGA | + | NC_037115.1 | 47358626 | T/A | 0.479 | 0.152 |  |
| Ots_crRAD20376-45 | GCAAAAGGTTACAAACCAATGACTGA | TTTCTCTTCTCCTCCTTGGACACAA | + | NC_037107.1 | 41283403 | C/A | 0.177 | 0.158 |  |
| Ots_crRAD20887-81 | GGTAGTTGGGCACCCTTTTACTTC | ACACACAATGTTTCTGGTTCAATGAATA | + | NW_020142527.1 | 183263 | G/A | 0.451 | 0.089 | Y |
| Ots_crRAD21115-24 | TGCAGGTGGGACTTAAACACA | TTGATATTCTGTGCTATCTGCCTCTG | + | NC_037097.1 | 27727076 | C/T | 0.294 | 0.119 |  |
| Ots_crRAD21752-66 | CAGGTCTAGGCATGGATCAAAGT | ACACACACACATGGCGTTTGA | - | NC_037110.1 | 43620486 | T/C | 0.457 | 0.137 |  |
| Ots_crRAD22960-100 | TGCAGGACAGAAATCAGGTCT | GAGGCAGATGACACGCTTAGG | + | NC_037113.1 | 15937728 | G/T | 0.484 | 0.125 |  |
| Ots_crRAD23631-48 | GTTACCTCTGCCATCCTAACCTC | GCTGTGACTGGCAGAAATGTATTTAG | + | NC_037097.1 | 73494810 | G/A | 0.015 | 0.069 |  |
| Ots_crRAD24807-74 | GAGAGCAGGGTAGATACCTGTTAGAC | GGTAGGTCTACGGGAATAGAGGAA | + | NC_037100.1 | 44035017 | A/T | 0.345 | 0.065 |  |
| Ots_crRAD25367-54 | GCGTCATGCTTATTACTGTGGGAT | AACATTGTGGGACATATTGTTCACATC | - | NC_037126.1 | 34187799 | A/C | 0.495 | 0.065 |  |
| Ots_crRAD255-59 | AGGAGCTGTGATGGGAAGATCTA | ACATAAATGGGCCTTTGTTTTACGC | - | NC_037126.1 | 6975904 | A/G | 0.307 | 0.100 |  |
| Ots_crRAD26165-69 | AGCTCAGCCACAATCTTCTCCTC | CTTCCTCCTCCAAAAACAATAACCC | + | NC_037106.1 | 52149501 | C/T | 0.488 | 0.401 |  |
| Ots_crRAD26488-63 | CTACAACATCCGTAGTGTACGACCTTA | GTTGAACACTAGGCAGGCTACG | - | NC_037116.1 | 39321622 | C/A | 0.485 | 0.263 |  |
| Ots_crRAD26541-47 | CCTACCCAAGTTACTTGACTGC | GGTTAAAAGAGTTCCATTGGTGAGTG | + | NC_037105.1 | 28118984 | G/A | 0.403 | 0.225 |  |
| Ots_crRAD27247-62 | CTGCACAGTGTATTCCTCCTTCTATG | ATGCTTCACAGGTCCTTATTGAACC | + | NC_037105.1 | 19867753 | T/C | 0.463 | 0.096 |  |
| Ots_crRAD27515-69 | CAGATGGTGCAGGCCGAA | ACTAGTTAGGGATAGATGGGACGCA | - | NC_037098.1 | 31417111 | A/T | 0.195 | 0.056 |  |
| Ots_crRAD27563-53 | TTCGGATGTACCGATCCTGTACT | GGCAATACATTGCAATGTCCGTACTC | + | NW_020128804.1 | 13235990 | G/T | 0.412 | 0.104 | Y |
| Ots_crRAD2806-42 | GGGTTAGCTTTTAGGCAGTTTGG | ACTAAACAACATCTATTTGACTTCATGCCA | - | NC_037113.1 | 15937591 | T/G | 0.493 | 0.125 |  |
| Ots_crRAD2835-56 | GTTCATGCTCTACAACATCTGTACCC | GGGTGTAAAGTTTGAACAGACCCT | - | NC_037097.1 | 63263430 | C/T | 0.304 | 0.121 |  |
| Ots_crRAD30341-48 | TGACTTGGGTAATCAGTTGAACGTTG | GTTCGGACGCAACATACGTTTTC | - | NC_037115.1 | 4216370 | A/T | 0.484 | 0.095 |  |
| Ots_crRAD32203-75 | GGCATACACACCGGACCATAAAAA | AGAAATGTAACACCTTAGAGCATCGAAT | - | NC_037112.1 | 23965978 | C/T | 0.179 | 0.095 |  |
| Ots_crRAD32330-33 | AGGACTTTGCAGTCTTGCTGAA | CACCATAGAGCCCCACAGTATAAAAC | + | NC_037124.1 | 7140007 | C/T | 0.425 | 0.075 | Y |
| Ots_crRAD32437-51 | TGCTGCAGGTGGATACCTCT | ACCATTGGTGCCACTCCGA | + | NW_020128922.1 | 141245 | C/T | 0.232 | 0.116 |  |
| Ots_crRAD32693-41 | GACTTTGATAGTCAGTTGAACGGTGTT | AACGGGCTCGGGAGAGGTA | + | NC_037109.1 | 58574924 | C/T | 0.299 | 0.068 |  |
| Ots_crRAD33054-62 | GGAATTATGGCCTTTGTCTCCGA | CATACTCCTCCCATGGAAGGAAATC | + | NW_020128803.1 | 689137 | T/A | 0.237 | 0.129 |  |
| Ots_crRAD33491-73 | CACCACCAAATTGGCAACAAAATCT | GATATCAATTTGCTGTGGGCTCATTC | + | NC_037114.1 | 6100851 | T/C | 0.484 | 0.303 | Y |
| Ots_crRAD35313-66 | GCAGGAAGAGTTCAGAGAAATCTTGAAAATAT | GCTAGTCACAACAAAAAGGCTGATT | - | NC_037109.1 | 70298983 | T/C | 0.499 | 0.106 |  |
| Ots_crRAD36072-29 | TGCAGGACCAACTTTCTCATACAA | CCTCCTCTAGAGAGGTCAGGAAAG | + | NW_020128813.1 | 617716 | T/C | 0.495 | 0.156 | Y |
| Ots_crRAD36170-52 | CGCCAGTCTGCCTCTGGTA | CAATACCCTATTATAAAAGCCCTAATCCTCAAC | + | NW_020128814.1 | 6417859 | C/T | 0.210 | 0.109 |  |
| Ots_crRAD36328-96 | CAGGGTTGAAATATGCTTCTCTTTGTTAACTA | GCCAGAGAAAATGCAGAGTGCAAA | + | NC_037100.1 | 6705203 | C/T | 0.447 | 0.136 |  |
| Ots_crRAD38095-86 | GGTCGTTGTGCCGCAACA | TTGACCGCGAAAACGTCTATCATAT | + | NC_037097.1 | 32570621 | G/A | 0.307 | 0.105 |  |
| Ots_crRAD42058-55 | CTGACACAATGAGTCGCTCGAAT | GTCACGACAGCCTGGGTTTTAT | + | NC_037102.1 | 9723313 | A/T | 0.500 | 0.088 |  |
| Ots_crRAD42811-155 | GAAGAAGCTGGATGTGAAGGTCT | TGTGCAGTTATGTCACATAATACAATGCC | + | NC_037115.1 | 15221303 | G/A | 0.378 | 0.054 |  |
| Ots_crRAD44016-74 | CTCATCCTGATGACCCTCCAGTA | AAGGAACCAAAGTTCCAGGTCATTT | + | NC_037115.1 | 39015874 | T/A | 0.030 | 0.078 |  |
| Ots_crRAD44588-67 | CTCTGGCCGCAAGTCAGC | GACACCCCTGATAGCCTGGTTA | - | NC_037116.1 | 20333782 | G/A | 0.446 | 0.121 |  |
| Ots_crRAD44604-139 | CTTGCAACTACCAACCTGCTCATTA | CACATGTTGTCTTACCCATTCTGGT | + | NW_020141172.1 | 440 | G/A | 0.294 | 0.042 |  |
| Ots_crRAD45659-62 | GAGTGTGGTGTCAAAAACAGATCTTT | GGGATAGAGAGATTCATGTTTTGTGGTG | + | NC_037109.1 | 9035408 | A/G | 0.306 | 0.196 |  |
| Ots_crRAD45985-75 | CCCACCTGTGGGTTTGACCT | TAGCCTAGTGGTTAGAGCATTGGTACTA | + | NW_020131906.1 | 4771 | T/C | 0.496 | 0.134 |  |
| Ots_crRAD46751-42 | GCAGGAACCTGCTTTAATGCTCTTA | GTGACTGATGATCCTGGATCAGATT | - | NC_037102.1 | 35130855 | G/A | 0.088 | 0.058 |  |
| Ots_crRAD47297-55 | GTAACTCCTCTCCCTGTTCGCTA | AAATGTCAAGGGCAACATGTTTGTTTAT | + | NW_020128840.1 | 978610 | T/C | 0.500 | 0.138 | Y |
| Ots_crRAD48459-154 | GCCTTCCATCCCACCATGC | AATGTTGCTATGTCATAGAGGGTGCA | - | NC_037106.1 | 51757766 | T/G | 0.473 | 0.191 |  |
| Ots_crRAD52613-29 | TGCAGGTTGAGGTCCATAACTAC | TTTCTGGGATCTTATATTTCAGCTCATTAAATC | - | NC_037102.1 | 2124216 | C/T | 0.466 | 0.087 |  |
| Ots_crRAD53756-52 | AGAGGAGCGGTCATCCGTT | AGCAGAGACAAAGACAATGATTAAAAGC | + | NC_037100.1 | 26043358 | C/G | 0.476 | 0.181 |  |
| Ots_crRAD55400-59 | GCAATGAGCCAACCCCTAACC | TTTTGTGATGTTTGTATGTCTAACAGAGAC | + | NC_037108.1 | 18236708 | C/T | 0.171 | 0.167 |  |
| Ots_crRAD55407-30 | TGCAGGATGCTCTATTTGGTTG | CAGTATTCCAAACTTCCAAATGCAGGA | - | NW_020128885.1 | 494289 | C/T | 0.459 | 0.052 |  |
| Ots_crRAD55506-54 | AGGCATGGCAATAAGGCTTTTATGA | CATTCTCTTCCTCCAGTAGTCCTGT | - | NW_020129176.1 | 18057 | G/T | 0.101 | 0.153 |  |
| Ots_crRAD57376-68 | TACGCATGAATGTGTATTTTATAATGTCCT | CCACGGCCCAAAAGTGACTAT | + | NC_037098.1 | 9937516 | T/C | 0.325 | 0.288 |  |
| Ots_crRAD57520-66 | ATACCAATAAGCACAGAGCTGTGTC | AGTGCTAGTCTTAATCCTCTTGCCAT | + | NC_037126.1 | 39954606 | T/G | 0.486 | 0.091 |  |
| Ots_crRAD60614-168 | GCCGTGAGAAACTGGTCAAATTAAGATG | CTCATGTCAAACACTCTGCACACT | + | NW_020128904.1 | 157706 | G/A | 0.316 | 0.080 |  |
| Ots_crRAD60620-51 | GAGTCCGAGGTGCTTAATGCAAA | AAGGGAAAATTCATAATGTTACTTGTGGTT | + | NC_037117.1 | 9425029 | A/G | 0.285 | 0.080 |  |
| Ots_crRAD61523-71 | GTGATCAAGTGCTTGTGTTACGTT | CCTGTCCCATTCAGTATGTAGTTTCAC | - | NC_037107.1 | 35352564 | C/T | 0.401 | 0.173 |  |
| Ots_crRAD63811-40 | CCGGATGTGGAGGTCCTGACA | ATGTGCAGTTTTGACACACAACATAATGTTAC | + | NC_037112.1 | 50604508 | T/G | 0.369 | 0.223 | Y |
| Ots_crRAD66330-61 | AACATTATTTCAATTACTCTCCCAGAAGGA | GGTGGTGAATCGGAGACAAGTTT | + | NC_037101.1 | 17933258 | G/T | 0.362 | 0.084 |  |
| Ots_crRAD69327-158 | CCATTTGACCAACGGAGCCATA | GCATTTCTTTTCCATCAACATATGTTGT | + | NC_037098.1 | 52051634 | A/C | 0.360 | 0.098 |  |
| Ots_crRAD73823-166 | TGTTCCATTATTGTTTGAAAGAGCACGA | CTGTAAAAGAGAATTCCGCCCAGAG | + | NC_037102.1 | 52896578 | G/C | 0.154 | 0.055 |  |
| Ots_crRAD75581-70 | TGCAGGTCGAAAAATATCCTGATTGA | CTATCAGTATCTCTCACACACTCTCCAT | - | NC_037098.1 | 20994028 | T/C | 0.415 | 0.102 | Y |
| Ots_crRAD76512-32 | GGGACAGGGCCCTGGAATA | AGAAAGAGACCCTCCGGAAGTG | + | NC_037107.1 | 25425417 | T/- | 0.477 | 0.128 |  |
| Ots_crRAD80791-34 | GCAGGTTCATGCTCTATAACATCCT | GGGTTTGATGGCACAAGTGGT | + | NC_037105.1 | 26006877 | C/T | 0.230 | 0.146 |  |
| Ots_crRAD88534-155 | GGTCCACTACACAAAGAGTTGCTA | AAAAATATCAAATCCCAGGCCTCCT | + | NC_037100.1 | 32025531 | T/C | 0.427 | 0.049 |  |
| Ots_crRAD9615-69 | AGGGAGTGGGAGGTACTGTAATTG | AGTATTGTTAGAGAGCCAACAGAGTC | + | NC_037107.1 | 25656094 | T/C | 0.135 | 0.188 |  |
| Ots_DDX5-171 | ACAAGAGACCGGGTTCAAATTGG | AGAGAAGGATGACAAGTGAGTGACA | + | NC_037128.1 | 7183341 | C/T | 0.247 | 0.100 |  |
| Ots_EP_529-60 | TTTTTGGGCATGCAAGTGTAGCA | GTTTGAGATTTAGTTCTGTATGGTCTCC | + | NC_037126.1 | 26852549 | C/T | 0.148 | 0.040 |  |
| ots_epic4_001_1 | CAGGCGTTTCAATGTCATGACAAA | TGGGTCGTCAAGGATTTCTTACTGAA | + | NC_037115.1 | 41454063 | A/G | 0.196 | 0.075 |  |
| ots_epic4_002_1 | AGAATATCCAGCGGCAGCAAA | CTAAGTCAGGCAGGTGTCGGA | + | NC_037109.1 | 57001432-57001434 | CAG/- | 0.470 | 0.166 |  |
| ots_epic4_005_1 | CGTTCTCATTGGGTAGACTGTCG | CACTCTGGGTAAGTACCTCACTCT | + | NC_037112.1 | 53657139 | G/A | 0.010 | 0.013 |  |
| ots_epic4_008_1 | GAAAGCCTTGGAAAGGTCGATGG | CAGTGCAGCCCCTTTGCC | - | NW_020128880.1 | 52937 | C/T | 0.426 | 0.275 |  |
| ots_epic4_009_1 | GGCATGGAGTTCAGAAAACAGGTA | TGCAGAATGCTTTGGTAGCTGTC | + | NC_037116.1 | 22554579 | G/A | 0.447 | 0.097 | Y |
| ots_epic4_012_1 | CTGACATCGGCTGCAGAGT | TACTCCCCCGGCTCCGAT | + | NC_037115.1 | 23865297 | A/G | 0.236 | 0.108 |  |
| ots_epic4_015_1 | TCCTTATCCCATCGCGCTCTATC | CTGCAGCTCCAGTATGGACTT | + | NC_037106.1 | 39743907 | G/T | 0.298 | 0.117 |  |
| ots_epic4_016_1 | GGACTCACAGACCATGAGAAGCT | GATCTCTCTGTACTTTGTTTCGTTCATCTTTA | + | NC_037106.1 | 39687111 | C/T | 0.149 | 0.067 |  |
| ots_epic4_019_4 | TATTGGACCTAATGGCGAAGCG | ATCAGCTCAGCCAGCTATATGTTC | + | NC_037114.1 | 15031604 | G/A | 0.244 | 0.071 |  |
| ots_epic4_020_1 | AGCTATTACTAATTTTGCTGGCAATCTG | CCCCCTACCTAAGTGCAAAGTAC | + | NC_037114.1 | 15553440 | G/T | 0.410 | 0.077 |  |
| ots_epic4_029_1 | CTTCCGGTTACTAGTCCACTAGGG | GTCCCCAAGAACACTCCCTCATTATTA | + | NC_037118.1 | 29611612 | G/A | 0.321 | 0.120 |  |
| ots_epic4_031_1 | TAGCCTCGCCAAATAAGTACCC | TGTAAGTTTACCCATCAAACAGCACT | + | NC_037097.1 | [37941334-37941335] | -/G | 0.025 | 0.036 |  |
| ots_epic4_032_1 | GGATGATTCTTTCCCGGGTCTTG | CAGCTCCAGTCCTCCTAACCTT | + | NC_037126.1 | 38215007 | G/C | 0.492 | 0.117 |  |
| ots_epic4_036_1 | CATTGATCATCGCCACAATAGGTG | GGTCGAAAGTCTTCTGTATCTCCATT | + | NC_037129.1 | 18656036 | C/A | 0.109 | 0.056 | Y |
| ots_epic4_037_1 | TTAATTTGGTTTAACAGGCACAGTGAGA | GGGAATAAACCTCGTGTTGATGC | + | NC_037106.1 | [9354306-9354307] | -/A | 0.065 | 0.095 |  |
| ots_epic4_046_1 | CATCCTCTCTCAGCCTATTTCAGAAAG | CAGGGTGTGACACATGTGTAACAA | + | NC_037129.1 | 16483182 | C/T | 0.003 | 0.017 |  |
| ots_epic4_047_1 | GAAGAGGCCCATTTCCTATCCG | CACATACATTCACTCTGGCACATACT | + | NC_037103.1 | 20045719 | C/T | 0.393 | 0.088 |  |
| ots_epic4_050_1 | CTGCATCACTGTGCTTAGAGGTG | AGGTCAGCCTGCAGGAACTAC | + | NC_037103.1 | 71540894 | T/C | 0.031 | 0.071 |  |
| ots_epic4_051_1 | TGCAGTTGTAATGTGTTGGGACTTA | AACATCAACACATGAGTTGGAACC | + | NC_037112.1 | 26025433 | A/T | 0.141 | 0.090 |  |
| ots_epic4_055_1 | GCGTTAAATATCAATAAGGAAGGAGGAC | ATCAGCACTTCTGCAGGAAACTTTA | - | NC_037127.1 | 34551964 | T/A | 0.099 | 0.092 |  |
| ots_epic4_059_1 | CACCCGTCTGGATGTAGGTAAGTAA | CTATCTGAGTGTTCTGGGATGTTGG | + | NC_037118.1 | 24440289 | G/A | 0.186 | 0.069 |  |
| ots_epic4_065_3 | CGGTCCCGTAGGTGTGATGG | GCCTTAGACATGATGCAAGGAGGT | + | NC_037100.1 | 31395469 | A/C | 0.303 | 0.035 |  |
| ots_epic4_070_1 | TGAACTGACTCCGACAACATTCAG | TAGACAGAGAGACCTCCTGGTCA | - | NC_037113.1 | 963266 | G/C | 0.043 | 0.161 |  |
| ots_epic4_071_2 | CATTTCCCAACTGAAGTGGGTTTAC | ACCTCCACATCTGTGGGATTGTC | + | NC_037123.1 | 10876910 | C/T | 0.171 | 0.160 |  |
| ots_epic4_072_1 | TGAATGTCCGGATCTAGTTTTCCTC | GTTGATGTGAAGGAAAACAGCCATC | + | NC_037102.1 | 51488329 | C/G | 0.138 | 0.081 |  |
| ots_epic4_073_1 | GATCTCAGTCCAGCTGAATTCAGT | CTTTGCGCAGCCCCTTTATG | + | NC_037117.1 | 20133201 | T/A | 0.011 | 0.065 |  |
| ots_epic4_078_3 | AAACTCTGACCCGGTTAACACTG | CTGGGTCCGTTGAGAACATACG | + | NC_037115.1 | 47048339 | T/G | 0.451 | 0.165 |  |
| ots_epic4_079_1 | CTTCCTCTAACACCACCTGGTATACA | CCCTTAGGAAGCTGAAAAGATTTCGA | + | NC_037102.1 | 52745980 | C/T | 0.010 | 0.051 |  |
| ots_epic4_082_1 | CTTTCATGGCATCATGCTTTGCATA | CAGACTTAGCTAATTGAGGACACCT | + | NC_037120.1 | 17601033 | C/T | 0.111 | 0.092 |  |
| ots_epic4_085_1 | GTCTCCAACATCGCTTTAGGGTAT | TTCCCTGAGAGAGTTTCTAATAGTTTGC | + | NC_037110.1 | 42216010 | A/G | 0.030 | 0.086 |  |
| ots_epic4_086_1 | ATGATGTGTGTGTGTGGACCATATAAAA | CGGGCACAGAGATAGGTCATTAGTA | + | NC_037101.1 | 30380278 | C/T | 0.022 | 0.036 |  |
| ots_epic4_088_1 | AGTCAGGCGTACCAGACTTTTTAAATG | TCATCCGAGTCTGGTTCGCTA | + | NC_037116.1 | 4401392 | C/T | 0.425 | 0.132 |  |
| ots_epic4_091_1 | GGCGTGAAAGCCCAACGATC | GTGCTATAAGCCAGCCCGTT | - | NC_037112.1 | 63759286 | C/T | 0.161 | 0.066 |  |
| ots_epic4_092_1 | ATCAATCTTTACTAACGGCATGACTTGA | GGGTTTAGTGAATGATTTGTGCCGAATAA | + | NC_037097.1 | 30558419 | A/- | 0.039 | 0.012 |  |
| ots_epic4_096_1 | CTAGCTGGTCTGTTCTTGGTTGG | GAGGTGAGGACGGCTATAGCA | + | NC_037126.1 | 4365539 | C/A | 0.046 | 0.101 |  |
| ots_epic4_107_2 | AGTAAACTCACGTTATACTGCAGCTT | TCAGGAGGCTTTTGGAAATCGATTC | + | NC_037115.1 | 4763935 | T/A | 0.042 | 0.110 |  |
| ots_epic4_110_1 | GAATTGCCATAGAGGCTGCAATC | TGATAGCAAATCTATAATCCGGAATGGC | + | NC_037097.1 | [85529136-85529137] | -/TC | 0.394 | 0.118 |  |
| ots_epic4_115_1 | AAAAAGCATACTTCATACTGGAACAGC | GTAGATCCAGATCAGAATTGTGGAGT | + | NC_037102.1 | 57335158 | T/C | 0.028 | 0.116 |  |
| ots_epic4_116_1 | ATCAAACTTCCTTTTCTAGGTACTTGCT | CGCAGGCAGTATCGTCTTTTGAT | + | NC_037110.1 | [25845274-25845275] | -/A | 0.013 | 0.050 |  |
| ots_epic4_117_2 | CGTTATGCGTTACGTGGTCTAC | AAAGAGGCATTTCTACTGGAAAAACAC | + | NC_037116.1 | 37835678 | G/A | 0.096 | 0.100 | Y |
| ots_epic4_118_1 | AGTCAAATTCACACAGAAATCATTGGCT | ATTCCAGCCAGTGGTTGTTTTAATTC | + | NC_037097.1 | 54739492 | G/C | 0.167 | 0.101 |  |
| ots_epic4_121_1 | AGGAGCTCTCCAAAGAAAATAACCAC | GAACCTATTGAACCCGACCGT | + | NC_037100.1 | 45702090 | G/T | 0.070 | 0.140 |  |
| ots_epic4_123_1 | CTGCGATAGCCGAAAGTCACAA | TTGTTCGAGTGGGTCAATTTTGTCA | + | NW_020142620.1 | 48899 | G/A | 0.050 | 0.078 | Y |
| ots_epic4_124_1 | TAACAGAAGTCGTACGTAAGTGTCAATT | ACCTGTCTGTATCTCCATTTCAACC | + | NC_037105.1 | 64724276 | A/G | 0.149 | 0.068 |  |
| ots_epic4_128_1 | ATGGCAAAATCCTCTGCTATAATGACA | TTGGATGACTTGGACCTGGAAGAT | + | NC_037109.1 | 24427455 | T/A | 0.072 | 0.065 |  |
| ots_epic4_129_1 | TGCAGGGAGATTTAGCAACACTTG | CACTGAGGCAAATCCAAGGAAAAG | + | NC_037120.1 | 19328948 | G/A | 0.493 | 0.067 |  |
| ots_epic4_131_1 | CTCAATAGAGTTGCAGCCTACCG | AAAGCAGTGATATGATGGCATTCTG | - | NW_020142577.1 | 852565 | T/A | 0.302 | 0.094 | Y |
| ots_epic4_132_4 | CGAACTTACACTTGTCCTTGCAATG | TACAGTTGATGCCGACTTTAGCAC | + | NC_037105.1 | 66602757 | C/T | 0.485 | 0.135 |  |
| ots_epic4_133_1 | CATGTTGATAGAAATGAGGAAAAACTGATTTAT | ACATTACCTACCGAGAGAGTTTTCATCC | + | NC_037099.1 | 42434208 | G/A | 0.235 | 0.121 |  |
| ots_epic4_145_1 | ATCTTCCAGATCACGACAAATGGG | CCCCGAGAACCCTGCTAATCT | + | NC_037110.1 | 38622355 | C/- | 0.050 | 0.047 |  |
| ots_epic4_146_1 | CTCACCGGTCAGTTTTATTAGGTACAG | CGTCTTGCCTAGTTTCAAAGGTAC | - | NC_037116.1 | 27119837 | G/T | 0.019 | 0.054 |  |
| ots_epic4_148_1 | TGTGGTGATAGTGAAGAGAGTTGTTTAC | AAAGTTGTGCTTAATGGCAAGCAA | - | NC_037129.1 | 8474800 | G/C | 0.410 | 0.080 |  |
| ots_epic4_149_1 | CTGGAAAGGTGGGTTTTGAAAAGTC | CATGTCTGAATCCTGGCTAGGC | + | NC_037105.1 | 3774260 | A/C | 0.148 | 0.059 |  |
| ots_epic4_154_1 | AGGCAGAGCGAGGTAACTAAAGT | GGCAAGCTTGTGGCGATACATA | + | NC_037110.1 | 3633543 | G/A | 0.260 | 0.189 |  |
| ots_epic4_158_1 | ATGCTGGCCACATGCGT | TTATCAGTCACGGGCCCTCT | - | NC_037128.1 | 2024421 | T/A | 0.476 | 0.044 | Y |
| ots_epic4_159_1 | CCCCTCCAAGAGTGTAAAGAACCA | GAAGAAAGGTTGTACTCTACAGATGTTGTG | - | NW_020128844.1 | 164706 | T/G | 0.005 | 0.037 |  |
| ots_epic4_160_1 | CATTGTGTTGGTTTCATAGGTTGATGG | TATAATAGCACATGTTTTGCATGAAGGG | + | NC_037099.1 | 69468628 | C/T | 0.060 | 0.062 |  |
| ots_epic4_162_1 | GTCAGACTGGGCTACTTTTGAAAGATC | AACCAGGCAGGATATAAGGTTGATT | + | NC_037103.1 | 48024326 | T/C | 0.091 | 0.113 |  |
| ots_epic4_165_1 | ATGTCTTTGGCCCATTTTAAGTCTTTAAT | ACCGGCGACAATGAGTCC | - | NW_020128971.1 | 55553 | C/G | 0.249 | 0.142 |  |
| ots_epic4_170_1 | GTAGTAGAAGCAGCAGTAAGGAAGAGA | CTAACCTGGGCATGAATACCTTCC | + | NC_037124.1 | 40278622 | G/T | 0.032 | 0.035 |  |
| ots_epic4_175_1 | AAGTGAAATGATTTGGACCAAAAGGC | GGTTTTATTGTGGAGCCTTTTGTTGA | + | NC_037124.1 | 15116261 | C/T | 0.312 | 0.207 |  |
| ots_epic4_176_2 | AAAGTTGTGCCCTTGATATAATGAGTCTG | CCTCCCGGTCGCGGTTG | + | NC_037110.1 | 44804264 | T/C | 0.223 | 0.090 |  |
| ots_epic4_181_2 | GAGACACTAACCTTGACACTGCTC | GTGAGTGTGGATACCGTACTGAGA | + | NC_037124.1 | 17815120 | G/T | 0.147 | 0.087 |  |
| ots_epic4_185_2 | CACACCAAATGTGTTGCTACTACT | CTAACTTCCGGTTAACCTCAGACC | + | NC_037104.1 | 69667100 | C/T | 0.122 | 0.080 |  |
| ots_epic4_189_3 | GCTACCACTCTGTCTAGTGTTAGGT | GTGCGGTTCATCAAGCCTAAAAC | + | NC_037105.1 | 75888767 | T/A | 0.101 | 0.049 |  |
| ots_epic4_193_3 | AGGACATGGGCTACCAAAAGGAA | GAAATGGAATAATGTGCCACTGCG | + | NW_020128921.1 | 621871 | A/C | 0.484 | 0.087 | Y |
| ots_epic4_194_1 | TCTCCTCTTTTTACGGCCAGA | TAATATAAATGGTGCCAGAGATGCG | + | NC_037127.1 | 17853113 | T/C | 0.017 | 0.010 |  |
| ots_epic4_195_2 | TCCATGCTGACGTGATGGTATTG | GCACGCAGTCATCAACAATGAATAATT | + | NC_037097.1 | 66175945 | G/A | 0.064 | 0.185 |  |
| ots_epic4_196_1 | CTGACTGGCTCCTCCATAATCACTTA | GTTGATCTGCTCGTCCACCTTTA | + | NC_037099.1 | 11276066 | A/C | 0.436 | 0.119 |  |
| ots_epic4_208_1 | GCGCTGCAGTATATATTGTGAACATTT | GCCCATGAATCACTCTGTCCTTT | + | NC_037097.1 | 20627929 | T/A | 0.045 | 0.059 |  |
| ots_epic4_217_1 | TGATTTTAGCATTAAAAGACTCCAAAAACG | CAGGAGCCAGAATGACTTGGA | + | NC_037120.1 | 6955367 | A/C | 0.375 | 0.073 |  |
| ots_epic4_221_1 | GTTTCATGTTGCCATCATTCAAGCA | AACCCGTAGTCATTTGATTTCTTTGG | + | NW_020128928.1 | 1880812 | C/T | 0.477 | 0.150 | Y |
| ots_epic4_222_1 | GTCAGGATGAATCAATATGGGAATGC | GAATGTAGAGATGGAAACCGGGAAG | - | NC_037118.1 | 23310605 | C/T | 0.119 | 0.275 |  |
| ots_epic4_225_2 | CAAGCAATATTATCAGCTGCAGGGA | TGACAAACCCAAAACCTCTAGAGC | + | NC_037118.1 | 10340952 | A/G | 0.083 | 0.039 |  |
| ots_epic4_226_1 | GAACAGAGATGGGTTGCTAAACTCA | CATCTTGCACCTTCTCAGCATGA | + | NC_037125.1 | 23679878 | C/T | 0.472 | 0.098 |  |
| ots_epic4_228_1 | GTCCACCAGAGATGTTTCTAGATAATTGAA | CTTCAGTTGTGTAATTCCACAGTTTCAT | + | NC_037102.1 | [18306964-18306965] | -/T | 0.144 | 0.143 |  |
| ots_epic4_229_2 | GTCTGCATCTCATTTGAGACAATGCA | CTGAACATTAGCATCAAACCAGCCA | + | NC_037100.1 | 57540434 | T/A | 0.034 | 0.016 |  |
| ots_epic4_230_1 | CATCCGTGAAGAACACCCTTCTTT | ACAGCTGATGAACTGTCAGAAACC | + | NC_037100.1 | 56951781 | G/C | 0.103 | 0.066 |  |
| ots_epic4_231_3 | AACAAGTCGCGGTTGTCATTG | CAATTCTGGGAGTTGAGTTCTGG | + | NC_037106.1 | 23370014 | C/T | 0.253 | 0.094 |  |
| ots_epic4_232_1 | TTTTGAGAGAATAGTCACGTGAGAGGA | AGAAGAAAATAAGACTTGCAGCCCAT | + | NC_037106.1 | 23124117 | C/T | 0.021 | 0.009 |  |
| ots_epic4_233_1 | TATGCAGGAACCAATAGGATACTCGAC | GCATCCTGCATGTGATCTAGAGT | + | NC_037106.1 | 24322231 | G/A | 0.001 | 0.035 |  |
| ots_epic4_235_1 | GTACTTTTTCCACCACTGCATCGC | GCAGGTGACTGCGCACTC | + | NC_037111.1 | 11469298 | T/G | 0.106 | 0.144 |  |
| ots_epic4_236_1 | GGGACTGTGGTGTGGGTATTATTG | TCCCTTTTCTTTTGACCCACATTTTAAATC | + | NC_037124.1 | 29929084 | C/A | 0.318 | 0.168 |  |
| ots_epic4_239_1 | AGACCCTGAAGTTACTTCCCTTCTC | CAGAGACATCTCCTCCTCTCTTAGC | + | NC_037101.1 | 64816474 | C/T | 0.014 | 0.026 |  |
| ots_epic4_240_1 | AGCAGTGAGTATAGAGAGCTACCG | TTTAGTCCTACTGGCAGTGACTGT | + | NC_037101.1 | 66192926 | A/G | 0.033 | 0.017 |  |
| ots_epic4_243_2 | GGCCACTCTAAAATGTGCCACAG | GCCGGATGTGGAGCTCCAG | + | NC_037126.1 | 17930971 | C/T | 0.226 | 0.124 |  |
| ots_epic4_246_1 | CTGACATGCCCCTCCAGCATTA | GCTCTAGCCCTCACCCTGTA | + | NC_037115.1 | 12447297 | A/C | 0.253 | 0.066 |  |
| ots_epic4_247_1 | ATCAATCGAGCTCCACTAATGCC | GGAAAAATGCTATTTGCTTTCAGTGG | + | NC_037102.1 | 71126343 | G/A | 0.294 | 0.180 |  |
| ots_epic4_248_2 | AATATGGATCATAACCGAGGTGCAG | CTACAACCACCAAAAGTTTGCTGT | + | NC_037102.1 | 39678948 | A/T | 0.483 | 0.215 |  |
| ots_epic4_250_1 | AATTATAGCAGGCATCAGAAGTGAAGT | TGTGCAGATATATATGCCTGCATCC | + | NC_037100.1 | 23729195 | G/A | 0.027 | 0.022 |  |
| ots_epic4_256_2 | CAGTTGGACGTACTGCCAAGTTATT | GATAATAAAAGGCCACTCTAAAATGTCACAC | + | NC_037105.1 | 29743931 | C/A | 0.032 | 0.133 |  |
| ots_epic4_257_3 | GCCATACAGCATGTCTGCAATC | CTGGATCTAACTGCAGTTCGTCTTC | + | NC_037106.1 | 12515674 | G/C | 0.472 | 0.061 |  |
| ots_epic4_258_2 | CCCATGGGAGGGTTTTAAAACAGTAG | GAGGACGAGTCGGTTAGATCC | + | NC_037118.1 | 3440725 | A/C | 0.061 | 0.056 | Y |
| Ots_Est1363-500 | ACCAGTCTGGTTCTATAAATTACTTGTGT | ATTCACAACAACGAATAAAAGGCACAG | - | NW_020142651.1 | 175121 | T/A | 0.364 | 0.294 |  |
| Ots_Est803-288 | CTCGTCCGATAGAGATGCCAAT | CCAGTGGGCTTCTATTTTCATTACCA | - | NW_020142539.1 | 4759829 | T/G | 0.366 | 0.112 | Y |
| Ots_ETIF1A-301 | ACAACATGTTTACTCCAATCCCATTG | GGAGAGAAAAGGAGAAATGATTGCCAT | - | NC_037108.1 | 38462857 | C/A | 0.484 | 0.099 |  |
| Ots_FARSLA-220 | GGCCAGGGTAGATTAAGGGTTC | ACTCTTAGATTCTGTTCAGGCTTCCA | - | NC_037105.1 | 82809166 | C/T | 0.436 | 0.352 |  |
| Ots_FGF6A_new-709 | CACATCCCGTCTTTTCACAACGTA | TGTTCATTCATGCGCAATTGGA | + | NC_037102.1 | 37585251 | G/T | 0.297 | 0.144 |  |
| Ots_GCSH-366 | GGCTCCTACGAGAAATTGATCAAATCC | AACAATTACAGGCAAAATAAATAGGGCTAC | + | NC_037108.1 | 65634622 | G/A | 0.491 | 0.169 |  |
| Ots_GH2-501 | TGTAGAATGGCCAAATAAACGAGTATTG | AGGCCAACAGTTCGTAGTTCC | + | NC_037123.1 | 1604553 | T/A | 0.210 | 0.179 |  |
| Ots_GPDH-338 | CAGCAGTGCTGCATAAATGCTC | GTTGGTTTAGTTGATAAGCTGATACACAA | - | NC_037110.1 | 7996596 | C/T | 0.139 | 0.150 |  |
| Ots_GPH-276 | GGTTTTTCAAACCATCTCTCTTTCTCTTG | TTGCCAAGCTTTTACTTCTGCGATAA | + | NC_037107.1 | 40844253 | G/A | 0.115 | 0.173 |  |
| Ots_GST-207 | AGAACTAGAATGAGGAGAACATGCATCA | GGGCTGACATCTTCTCGACAAAG | - | NC_037117.1 | 20087794 | G/A | 0.281 | 0.104 |  |
| Ots_GTH2B-1542 | ACACCCTAATCCCTCAAGAGTTGAT | CAATGAACAAGTGTGTGATTAATAGGGT | - | NC_037121.1 | 26054245 | G/C | 0.411 | 0.173 |  |
| Ots_hnRNPL-82 | TTTCCTTGTTCATCCATCAGGCATAA | ATGTTCCACAAACTGTCATCCCTAC | + | NC_037109.1 | 46418615 | T/A | 0.468 | 0.141 |  |
| Ots_hsc71_3prime-77 | TGCTTTGGTTAGGCACACGATAA | AGGGCAATCTTGCTTGTAGCTTC | + | NC_037105.1 | 53223724 | A/G | 0.493 | 0.098 | Y |
| Ots_hsc71_5prime-453 | TAGTTTGTAACTTCCAACGTGTCAATTT | TTGCGGGACAGGAAATCATTGTAC | - | NC_037105.1 | 41001821 | C/T | 0.376 | 0.117 | Y |
| Ots_hsp27b-138 | GATACTGTAGCCTAGTCCCAGATCAT | TAACAGTCAGAGGATGGTGAAATCATAG | + | NC_037105.1 | 67075478 | C/T | 0.487 | 0.157 |  |
| Ots_IGF_I_1-76 | CAGGGTAGGCCGTCAGTGTAAAATAAGTA | CCTGGCGGCCAGCTCTAACA | - | NC_037113.1 | 17781778 | A/T | 0.112 | 0.238 |  |
| Ots_il_1racp-183 | TCATACTCCTTGTCGTCTGAATGATG | AGAATAACCAACTAGGGCCATTCATT | - | NW_020142577.1 | 82150 | T/G | 0.424 | 0.049 | Y |
| Ots_IL11-501 | GTGTGTCTATGTCTTTCTGCCTTCCA | GGTGGTGGTGTTTCCTTGACG | - | NW_020129160.1 | 112307 | G/A | 0.499 | 0.194 |  |
| Ots_ins_115-115 | AAGAACCCTGCAAGAGGAGAAAAATATA | AGAAATGGCATGTAGGAAACTCATGATC | - | NC_037105.1 | 65957209 | T/C | 0.049 | 0.066 |  |
| Ots_IsoT-419 | CAGCCCTCTGAGATACAGAAAGC | GGCATCTGTTGTGACTCAGGTAA | + | NC_037108.1 | 10323291 | T/A | 0.154 | 0.090 |  |
| Ots_LEI-192 | CGAGAAGTCTCCCTTTGAGAGGT | AGCTGGAGAAGCTGCTGACCTA | - | NW_020128831.1 | 753109 | G/T | 0.069 | 0.116 |  |
| Ots_LWSop-638 | ACTGTCTCCACTTGCGGTAAGA | GATTACTGTCACTATGCTACGGTGTTC | + | NC_037098.1 | 44036657 | A/G | 0.135 | 0.119 |  |
| Ots_mapKpr-388 | GGACATGGCTGAAAACATATGCATATAC | CCTCTCACTGTGCCATTCAACTAT | - | NC_037103.1 | 3593175 | A/T | 0.454 | 0.153 |  |
| Ots_MHC2-163 | ATATCAGATTCAACAGCACTGTGG | TGTCTTGTCCAGTATGGCTCTGT | - | NW_020130363.1 | 58298 | A/C | 0.343 | 0.380 |  |
| Ots_mybp-122 | TTTATTGAACACTCACAGTGAACAGACA | TTTTTGAGTAAAAGAGATGGTGAAATGGAA | + | NC_037109.1 | 31784059 | C/T | 0.485 | 0.135 |  |
| Ots_nelfd-165 | GATCCTGTGGCTGAGTTTATAGGT | TATTTCCACTACATCCTCATCCAAGGTT | + | NC_037098.1 | 27801414 | A/G | 0.427 | 0.120 |  |
| Ots_nkef-126 | AGACTATCCCATCTGTGGCCTAA | TGGGTAGAGTGCAAATTATTATGACCAA | - | NC_037106.1 | 51361376 | C/T | 0.272 | 0.340 |  |
| Ots_nramp-345 | CTGACCTGCATGCTCTGCAATA | TGTCTGTATGTTGACTATTTTCTCTCTTCC | + | NC_037103.1 | 63847460 | A/G | 0.499 | 0.219 |  |
| Ots_ntl-161 | GATTGTCTTGGAGTACTCTTGTCTGA | TTCCATTCCGAAAACCCGAGAC | + | NC_037127.1 | 20616326 | A/T | 0.452 | 0.110 | Y |
| Ots_Ots2-179 | GCAACAATGTAAATGTCAAGTGGGA | TTCAGAATGTTTATCTGCCCTCCGT | + | NC_037128.1 | 5089897 | T/C | 0.498 | 0.085 |  |
| Ots_P53-372 | CTGGGTTGACCCCCAACTAATG | GCTTCTTCAGGTTGATCTCCTCTG | + | NC_037126.1 | 40916634 | G/A | 0.499 | 0.108 |  |
| Ots_parp3-47 | TGGAGTGTTTATTGAACAGTAACAAACC | CTCTGAGTCGGTTCAATGTCTGTC | + | NC_037118.1 | 10728055 | T/C | 0.325 | 0.073 |  |
| Ots_PEMT-285 | ATCCTGGCCCAATTCCAAGATATTCTA | TGGAATGATTGTCATTGGCTTTTGAGA | - | NC_037120.1 | 10785871 | C/T | 0.500 | 0.081 |  |
| Ots_Prl2-501 | TTAATAACCATAGGACAACCATGCATGT | CACAAAAGCGGTTATAATAAGGTTAACTCAAA | - | NC_037097.1 | 46840237 | T/C | 0.481 | 0.068 |  |
| Ots_RAD10261-564 | CCTTTTGGGAGTCTCACTCTATATTGG | GTGATCTTTCTGCTTCTCTTTCCAGTT | + | NC_037099.1 | 57679033 | G/T | 0.173 | 0.151 |  |
| Ots_RAD10583-85 | AGGTGAGGAAATGTACAGAGACTGTGTATTA | AATCAAGCAATAACAAATAACACCTTTGC | - | NC_037119.1 | 11909309 | A/G | 0.499 | 0.116 |  |
| Ots_RAD1072-72 | CAGGATACAGGGTTGTCTCTATGAGG | GGGACGAAATAACCGTCTGTTG | - | NW_020128832.1 | 1105928 | A/G | 0.444 | 0.204 | Y |
| Ots_RAD1282-87 | TGCAGGCAAGGGAAGTTTACTA | TTGAGGGTGGTTGTGTTTTACTGACT | + | NC_037126.1 | 4894246 | T/G | 0.499 | 0.208 |  |
| Ots_RAD14482-78 | ATGATGCGGGCAGCATGGC | CACATGAGGACAAACATCGTACCATC | - | NC_037097.1 | 58313121 | C/G | 0.053 | 0.112 |  |
| Ots_RAD17721-87 | ACACAAAACGCAGAAATAAAAATATAATTCATG | TATATCTCCGTAATTAATAGAACAAAAGGACCA | + | NC_037129.1 | 35443119 | G/A | 0.270 | 0.129 |  |
| Ots_RAD17873-67 | GGTTCATCCATCTATCCAGGCAAGA | ATACATTTCCTCTAACCCTCTGCTACAC | - | NC_037127.1 | 19850775 | A/T | 0.499 | 0.113 | Y |
| Ots_RAD2068-78 | GACATGTGGTCTAGACCATTAAGAGTG | CCATCTGTCTTAGTTAACCATACCAAAAGTAG | - | NW_020128931.1 | 63633 | A/C | 0.234 | 0.090 |  |
| Ots_RAD2357-80 | CAGGCAGGTTTGTGGGCTTTATA | GGCACATTTGGATTCGTTCAGC | - | NW_020128812.1 | 1225143 | A/G | 0.478 | 0.060 | Y |
| Ots_RAD249-66 | CTGTCCTTGAGGCCGCAATC | TTCAGCTCGAACTCATCAAGAAAATAAT | + | NC_037111.1 | 23946495 | T/A | 0.440 | 0.101 |  |
| Ots_RAD3386-57 | CCATGTCTACCTTTCTCCATCAAGT | CCTCCATCTGATCAAGGAGAAGTG | + | NC_037115.1 | 7517544 | G/- | 0.384 | 0.123 |  |
| Ots_RAD3513-79 | CTTTGAGTGAGTCACTGCACCAA | GACCATCACCTCAATCTGGACAAG | + | NC_037097.1 | 81595633 | G/C | 0.460 | 0.087 |  |
| Ots_RAD3737-92 | GCAGGCTTTAAGTAAAGTGTTACAACAT | GCTGTTTTTGACATGGTCACTGTTG | - | NC_037107.1 | 43629558 | C/T | 0.276 | 0.108 |  |
| Ots_RAD3766-75 | AGACTTCACCAAAAACAGGTAAACACT | GTAGAGTAGACGGTCTCATTCAGTACG | - | NC_037121.1 | 20783284 | A/T | 0.044 | 0.371 |  |
| Ots_RAD3858-74 | CAGGGTAGAGAGAAATGCATCAGTTC | CCCTTGAATACTTGTAAGGCACATTG | - | NC_037097.1 | 58198584 | A/T | 0.327 | 0.111 |  |
| Ots_RAD3925-69 | GCAGGTAGAACATACACTGTGTGG | ATGGGAAACTGAGGGTTCATAACATTTTATAT | - | NC_037112.1 | 21782621 | G/T | 0.455 | 0.086 |  |
| Ots_RAD4548-83 | GCAGGCCCTGCTACTAGGTTT | GCTGCACTTCACACACTTTATCAACT | + | NC_037119.1 | 181784 | G/C | 0.029 | 0.069 |  |
| Ots_RAD4999-89 | CAGGCAAAGGCACCATTACTGT | CTCAGCAGTAATCTATAAAACACACAATTTAAC | - | NC_037098.1 | 12784193 | C/T | 0.170 | 0.238 |  |
| Ots_RAD5848-75 | GAATTTGTAGTCTTGCTGAGATCTTCTG | GGCATGACATTTGATAGCCTGTGTAATT | - | NC_037116.1 | 38321500 | C/T | 0.164 | 0.099 |  |
| Ots_RAD6097-74 | CAGGTGTACCCCCTCCTCCAT | TGGCAGTCTTGACACCGGTTATAA | + | NC_037097.1 | 58147682 | G/A | 0.153 | 0.092 |  |
| Ots_RAD6688-83 | TGCAGGTTTATAGATCAGATTCGGA | GAAAGAAGAAGAAACACTAATGTGCATTCTGA | - | NC_037104.1 | 31096214 | C/T | 0.106 | 0.201 |  |
| Ots_RAD7145-85 | TTCCTTCGAGACAATTGTCCCACAA | ACGATGACTCCCGACTCCACT | + | NC_037099.1 | 77753529 | C/A | 0.486 | 0.145 |  |
| Ots_RAD7165-78 | CAGGAGCATCAGGGACTTTTCCTT | GGGAGATGACATTCTAATGAGAGGTTGA | - | NC_037105.1 | 60667129 | A/G | 0.264 | 0.081 | Y |
| Ots_RAD7936-152 | TGCAGGGTAGTAACATCATGGG | TTACTGACACCAGCCATATTCAACGT | - | NW_020128810.1 | 1084356 | A/C | 0.492 | 0.149 | Y |
| Ots_RAD8354-91 | ATTTAAGCTGCAGCATGCTTCT | ATGGTTTTTGGTTGAACCCTATCTCT | + | NC_037122.1 | 19216461 | T/A | 0.327 | 0.093 |  |
| Ots_RAD9039-66 | TGCAGGTGCTGTCACTAGGTAAATA | TCTAACTACTATAGCTTAGCCAGGTGTG | - | NC_037111.1 | 15171690 | A/C | 0.023 | 0.087 |  |
| Ots_RAD9704-89 | TGTCTCATTGTTTAGTTCTCTCTTTCCT | CATGAGAATGAGCATGCATGGTTAA | + | NC_037102.1 | 17120697 | T/C | 0.092 | 0.168 |  |
| Ots_RAD9970-86 | TGCAGGACACACCTTAGAACAATAAA | TTGTGTCTTCTGCAGTAATTACAGTAGAAT | - | NC_037118.1 | 25977767 | A/T | 0.421 | 0.223 |  |
| Ots_RAS1-193 | AAAATGCACTCAATCTTCAAGCAGT | ATGCCAAACAGGTTTTGATCATTTCTG | + | NC_037115.1 | 37925620 | C/A | 0.127 | 0.084 |  |
| Ots_redd1-184 | AGTTGAGACCTTCAGTTCTTAGGGTATA | GGATGTGGAAGCAAGCTAGTGTTT | - | NC_037117.1 | 27483610 | A/G | 0.183 | 0.075 |  |
| Ots_S7-1-336 | CCATGCCATCATAAACAACCTAACAAG | GTTTAGCCTGAGGTATGGTTGAGTA | + | NC_037114.1 | 33195644 | G/A | 0.499 | 0.129 |  |
| Ots_SCLKF2R2-135 | GGAGAAAGGGAAAATTAGTCATTGTTGC | CCTCCTGTGTATGAGTATGTTAAGTTCA | + | NC_037101.1 | 73843437 | A/T | 0.490 | 0.096 | Y |
| Ots_SEXY3-1-1507 | CTTGTGAGGCTCCAGTGTCG | CATCTGGACAAAAATAACACCTATGTAAGAAAG | N/A | N/A | 5-20* | TCAGCGAAGTGGAGAT/- | - | - | Y |
| Ots_SL-1317 | AGCAAAGAGTAGAGGTATCCATGTG | TTTCATCATCCTTCCCAAGATACTTCCT | + | NC_037109.1 | 62174556 | A/G | 0.486 | 0.175 |  |
| Ots_stk6-516 | AACGTGGAATAGTCTTTCACCCA | GCAGTTTGTCTATTAGGTCTCTGCTG | + | NC_037098.1 | 53397399 | G/T | 0.102 | 0.064 |  |
| Ots_SWS1op-359 | TTGCATGGGTTCAATAGCAGGTAAATT | TGTTAGGAGATATCAAAGACATCGAACA | - | NC_037113.1 | 8656175 | T/A | 0.457 | 0.075 |  |
| Ots_TF-179 | CCAATCACATCTACAGCAAGCTGA | TGTAAAAGTAAATGCTGAAAAAGCTTGC | - | NC_037124.1 | 27506903 | A/G | 0.130 | 0.043 |  |
| Ots_TF1-SNP1-702 | AGAACACGAAACAAGTCAGAAATGAC | GCGTAAAGTCGGACAAAGAGCTA | + | NC_037124.1 | 27509693 | A/C | 0.469 | 0.097 |  |
| Ots_TLR3-501 | CTTCGGCTCCAGAAGAACCTAA | GTGTTGCACACATACTCATCCCT | - | NC_037130.1 | 7234189 | C/T | 0.443 | 0.075 |  |
| Ots_Tnsf-501 | GTGCCAGTTGTGTATGGGAGAC | GAGTACTGATAGAAGGAGAAAGGATACAC | - | NC_037124.1 | 27507574 | C/T | 0.466 | 0.136 |  |
| Ots_tpx2-125 | GTGGCCACGTAGTTCTACACAA | TTCCAAATTGAGCACAAAAGCATCTTG | - | NC_037118.1 | 21879673 | G/A | 0.456 | 0.132 |  |
| Ots_txnip-278 | CCCTTATCAAACTGAAGGCGGAT | CCCTCCAACTAGCAAAATAGCAGAG | + | NC_037127.1 | 23141496 | G/A | 0.031 | 0.057 |  |
| Ots_u07_07-288 | TAGACTCTCACCCAGGATCTGGTA | GCATCTAGTACCACTACAAATCTCTTGT | + | NW_020128844.1 | 15617 | G/A | 0.451 | 0.082 |  |
| Ots_u07_18-314 | TTTGCGATAAACAACAAACTGCGT | CATTGGTAGAGACATGCATTTAGCTAGG | - | NC_037126.1 | 36420567 | T/A | 0.346 | 0.101 |  |
| Ots_u07_19-413 | AGAAGGCAGACTGACTGGTTTAGTTTA | CAAGAAAGCTGAGAGCTCTGTGTAAC | + | NC_037104.1 | 56660234 | G/A | 0.106 | 0.061 |  |
| Ots_u07_20-356 | GTAATGAAACTTTCAAGCTAGCATAGCA | ACAATTGAAATCACCAATCCAACAAAAC | - | NC_037099.1 | 39126796 | C/A | 0.012 | 0.032 |  |
| Ots_u07_25-339 | GAGTCTTTCTATTTTTACAGCGTTATAGGC | TCATTATACAGTAACAGGCAGGCCTAG | - | NC_037097.1 | 12626175 | T/C | 0.478 | 0.162 |  |
| Ots_u07_49-271 | ACTGTATAAAATAAATCTGCTGAGGAAGGA | CCTATGAAATATATCTGTTATCTAGGGCTCAAG | + | NC_037108.1 | 31470258 | T/C | 0.425 | 0.133 |  |
| Ots_u07_57-373 | TTCTTGGTCCAAGCAAGTCCAAC | CTACCCTTTGCTTTGCACACTTG | + | NC_037107.1 | 12974126 | T/A | 0.336 | 0.335 |  |
| Ots_u202-190 | GACTCCAGCCATTATCACCTTAACC | GTAGAAGATTGCTGTAGATGGCTATGAC | - | NC_037107.1 | 3872379 | T/A | 0.500 | 0.200 |  |
| Ots_u211-182 | TTGGAATGTAGCCAAGTCATGGAAC | CAGACCCCTTGTAAAGATTCAATCAAAG | - | NC_037101.1 | 29079237 | C/T | 0.497 | 0.183 | Y |
| Ots_U2446-123 | TGTGAGCTCCCATTTCAGCATT | TCAGCTAGACCAGGCCATTTG | - | NC_037125.1 | 23877990 | C/A | 0.465 | 0.129 |  |
| Ots_u4-291 | AATCCACTTGTTATGTGTGTAGGAATTG | ATGGATCCAAGGAGCCCCATTAA | - | NC_037101.1 | 62235849 | T/C | 0.336 | 0.141 |  |
| Ots_U5121-488 | ACTTGACAGGAGTCTGAATTAAACTCT | TTGTCTCCAAACTCTGGGCAAAC | + | NC_037101.1 | 33435348 | C/T | 0.293 | 0.103 |  |
| Ots_U608-861 | GTACGTGTCACAAATGACAACCTATTT | CACATGAATAACAGACGATCCTTACAACAG | + | NC_037099.1 | 493159 | G/A | 0.279 | 0.042 |  |
| Ots_unk526-192 | CATTCTTTTTGAGAATCACTAGCCACAA | TACCACAAGTGCTATAGTCAAGACTGT | - | NC_037112.1 | 30269244 | A/G | 0.262 | 0.096 |  |
| Ots_USMG5-501 | GACAAAATGGGTACGAGAAACTGACA | GTGGTGTACAACCAGTTCACATTCT | - | NC_037128.1 | 2731278 | A/G | 0.163 | 0.089 |  |
| Ots_ZR-494 | TAAGAAATAGGCCTACCAGAAAGTACCA | ACACTGTTTATCAGATTCATGCATGAAA | - | NW_020142510.1 | 14916967 | G/A | 0.476 | 0.225 | Y |

Supplementary Table S3. Number of Chinook salmon used in broodstocks (Brood), number of discrete samples received from the broodstocks (Received), and number of individuals successfully genotyped (Genotyped) for 60 populations of Chinook salmon during 2013-2019. CU is conservation unit number.

|  |  | Northern and Central Coast British Columbia | | | | | | | | | | |
| --- | --- | --- | --- | --- | --- | --- | --- | --- | --- | --- | --- | --- |
|  | CU | 43 | 50 | 45 | 54 | 42 | 39 | 38 | 39 | 37 | 37 | 39 |
| Year |  | Yakoun | Kitsumkalum | Kloiya | Bulkley | Kitimat | Nusatsum | Wannock | Atnarko | Chuckwalla | Kilbella | Salloomt |
| 2013 | Brood | 72 | 46 | 15 | 28 | 541 | 21 | 82 | 896 | 0 | 0 | 4 |
|  | Received | 0 | 137 | 0 | 0 | 0 | 0 | 0 | 894 | 0 | 0 | 0 |
|  | Genotyped | 0 | 135 | 0 | 0 | 0 | 0 | 0 | 793 | 0 | 0 | 0 |
| 2014 | Brood | 49 | 50 | 8 | 30 | 502 | 17 | 86 | 947 | 2 | 10 | 7 |
|  | Received | 0 | 149 | 0 | 0 | 0 | 0 | 0 | 908 | 0 | 0 | 0 |
|  | Genotyped | 0 | 149 | 0 | 0 | 0 | 0 | 0 | 588 | 0 | 0 | 0 |
| 2015 | Brood | 50 | 51 | 0 | 54 | 495 | 8 | 111 | 1,015 | 9 | 16 | 33 |
|  | Received | 0 | 139 | 5 | 0 | 0 | 0 | 0 | 1,022 | 0 | 0 | 0 |
|  | Genotyped | 0 | 137 | 5 | 0 | 0 | 0 | 0 | 863 | 0 | 0 | 0 |
| 2016 | Brood | 41 | 50 | 17 | 27 | 736 | 11 | 81 | 1,013 | 17 | 13 | 19 |
|  | Received | 41 | 142 | 0 | 37 | 750 | 10 | 48 | 1,003 | 18 | 14 | 19 |
|  | Genotyped | 40 | 136 | 0 | 31 | 703 | 10 | 47 | 628 | 5 | 3 | 17 |
| 2017 | Brood | 48 | 35 | 9 | 22 | 685 | 14 | 93 | 938 | 10 | 17 | 13 |
|  | Received | 0 | 0 | 0 | 31 | 0 | 0 | 94 | 942 | 17 | 26 | 0 |
|  | Genotyped | 0 | 0 | 0 | 26 | 0 | 0 | 93 | 865 | 14 | 24 | 0 |
| 2018 | Brood | 89 | 48 | 12 | 15 | 537 | 40 | 95 | 1,075 | 18 | 35 | 25 |
|  | Received | 99 | 0 | 0 | 16 | 552 | 50 | 94 | 1,020 | 25 | 34 | 24 |
|  | Genotyped | 95 | 0 | 0 | 15 | 551 | 40 | 94 | 972 | 25 | 34 | 19 |
| 2019 | Brood | 0 | 44 | 0 | 16 | 656 | 30 | 91 | 976 | 26 | 31 | 28 |
|  | Received | 82 | 0 | 0 | 16 | 620 | 29 | 90 | 971 | 26 | 34 | 26 |
|  | Genotyped | 82 | 0 | 0 | 16 | 608 | 29 | 90 | 946 | 26 | 34 | 26 |

Supplementary Table S3 continued

|  |  | West Coast Vancouver Island | | | | | | | | | | | | |
| --- | --- | --- | --- | --- | --- | --- | --- | --- | --- | --- | --- | --- | --- | --- |
|  | CU | 31 | 31 | 31 | 31 | 31 | 32 | 32 | 31 | 31 | 32 | 32 | 31 | 32 |
| Year |  | Robertson | Nahmint | Nitinat | San Juan | Sarita | Conuma | Burman | Thornton | Toquart | Leiner | Tlupana | Bedwell | Gold |
| 2013 | Brood | 3,570 | 24 | 2,481 | 274 | 175 | 1,340 | 201 | 165 | 76 | 119 | 28 | 24 | 167 |
|  | Received | 4,108 | 28 | 197 | 0 | 275 | 0 | 191 | 0 | 0 | 0 | 31 | 0 | 197 |
|  | Genotyped | 4,105 | 26 | 197 | 0 | 263 | 0 | 190 | 0 | 0 | 0 | 31 | 0 | 188 |
| 2014 | Brood | 4,358 | 15 | 2,398 | 126 | 83 | 1,507 | 165 | 198 | 76 | 38 | 29 | 0 | 161 |
|  | Received | 4,153 | 19 | 300 | 0 | 100 | 297 | 161 | 0 | 0 | 57 | 29 | 0 | 100 |
|  | Genotyped | 3,979 | 14 | 295 | 0 | 97 | 295 | 161 | 0 | 0 | 57 | 29 | 0 | 92 |
| 2015 | Brood | 4,368 | 41 | 2,432 | 136 | 206 | 1,716 | 208 | 195 | 26 | 126 | 0 | 20 | 39 |
|  | Received | 4,731 | 57 | 297 | 95 | 285 | 200 | 249 | 99 | 30 | 88 | 0 | 36 | 91 |
|  | Genotyped | 4,593 | 52 | 285 | 95 | 283 | 200 | 246 | 98 | 24 | 87 | 0 | 23 | 90 |
| 2016 | Brood | 4,674 | 31 | 2,448 | 318 | 208 | 1,634 | 196 | 80 | 18 | 143 | 0 | 8 | 314 |
|  | Received | 4,580 | 73 | 199 | 0 | 283 | 200 | 161 | 84 | 0 | 0 | 0 | 13 | 0 |
|  | Genotyped | 4,505 | 73 | 198 | 0 | 250 | 49 | 152 | 59 | 0 | 0 | 0 | 11 | 0 |
| 2017 | Brood | 4,610 | 47 | 2,257 | 266 | 305 | 1,700 | 191 | 77 | 21 | 101 | 0 | 31 | 122 |
|  | Received | 4,730 | 95 | 0 | 102 | 304 | 0 | 0 | 0 | 22 | 0 | 0 | 31 | 0 |
|  | Genotyped | 4,711 | 40 | 0 | 102 | 300 | 0 | 0 | 0 | 18 | 0 | 0 | 1 | 0 |
| 2018 | Brood | 3,880 | 12 | 3,491 | 47 | 224 | 1,758 | 47 | 83 | 21 | 55 | 0 | 25 | 0 |
|  | Received | 3,718 | 22 | 800 | 47 | 224 | 0 | 0 | 86 | 31 | 0 | 0 | 64 | 0 |
|  | Genotyped | 3,694 | 17 | 762 | 47 | 210 | 0 | 0 | 86 | 31 | 0 | 0 | 30 | 0 |
| 2019 | Brood | 3,936 | 34 | 2,698 | 163 | 296 | 1,632 | 46 | 84 | 6 | 207 | 0 | 16 | 257 |
|  | Received | 4,038 | 35 | 2,749 | 161 | 296 | 300 | 40 | 88 | 14 | 103 | 0 | 30 | 220 |
|  | Genotyped | 4,032 | 35 | 2,724 | 159 | 296 | 298 | 40 | 88 | 14 | 102 | 0 | 29 | 214 |

Supplementary Table S3 continued

|  |  | West Coast Vancouver Island | | | | | East Coast Vancouver Island | | | | | | |
| --- | --- | --- | --- | --- | --- | --- | --- | --- | --- | --- | --- | --- | --- |
|  | CU | 31 | 31 | 31 | 32 | 31 | 25 | 22 | 83 | 25 | 29 | 27 | 27 |
| Year |  | Cypre | Kennedy | Sooke | Tahsis | Tranquil | Chemainus | Cowichan | Nanaimo summer | Nanaimo fall | Nimpkish | Oyster | Big Qualicum |
| 2013 | Brood | 44 | 127 | 0 | 7 | 28 | 7 | 134 | 36 | 173 | 59 | 13 | 894 |
|  | Received | 0 | 0 | 0 | 0 | 0 | 0 | 117 | 0 | 0 | 0 | 0 | 931 |
|  | Genotyped | 0 | 0 | 0 | 0 | 0 | 0 | 97 | 0 | 0 | 0 | 0 | 917 |
| 2014 | Brood | 0 | 0 | 102 | 6 | 0 | 27 | 403 | 124 | 111 | 140 | 39 | 1,525 |
|  | Received | 0 | 0 | 0 | 8 | 0 | 0 | 447 | 0 | 0 | 0 | 0 | 1,537 |
|  | Genotyped | 0 | 0 | 0 | 8 | 0 | 0 | 423 | 0 | 0 | 0 | 0 | 1,367 |
| 2015 | Brood | 50 | 116 | 156 | 29 | 0 | 65 | 403 | 59 | 155 | 159 | 31 | 1,528 |
|  | Received | 57 | 54 | 0 | 78 | 0 | 0 | 408 | 0 | 0 | 0 | 0 | 1,704 |
|  | Genotyped | 42 | 47 | 0 | 77 | 0 | 0 | 396 | 0 | 0 | 0 | 0 | 1,594 |
| 2016 | Brood | 0 | 112 | 88 | 105 | 2 | 12 | 427 | 51 | 105 | 122 | 26 | 1,594 |
|  | Received | 0 | 0 | 0 | 0 | 0 | 14 | 311 | 0 | 0 | 86 | 0 | 1,535 |
|  | Genotyped | 0 | 0 | 0 | 0 | 0 | 14 | 274 | 0 | 0 | 77 | 0 | 932 |
| 2017 | Brood | 0 | 20 | 95 | 69 | 27 | 0 | 332 | 146 | 173 | 69 | 43 | 1,826 |
|  | Received | 0 | 12 | 0 | 0 | 27 | 12 | 337 | 0 | 0 | 0 | 0 | 1,875 |
|  | Genotyped | 0 | 8 | 0 | 0 | 6 | 6 | 309 | 0 | 0 | 0 | 0 | 1,740 |
| 2018 | Brood | 0 | 42 | 146 | 90 | 24 | 16 | 383 | 116 | 255 | 17 | 19 | 2,000 |
|  | Received | 0 | 0 | 0 | 0 | 0 | 0 | 313 | 75 | 0 | 0 | 0 | 2,025 |
|  | Genotyped | 0 | 0 | 0 | 0 | 0 | 0 | 285 | 75 | 0 | 0 | 0 | 1,921 |
| 2019 | Brood | 0 | 14 | 138 | 153 | 0 | 9 | 355 | 59 | 215 | 0 | 39 | 1,744 |
|  | Received | 0 | 0 | 112 | 76 | 0 | 9 | 308 | 59 | 211 | 0 | 39 | 1,749 |
|  | Genotyped | 0 | 0 | 99 | 76 | 0 | 9 | 307 | 59 | 203 | 0 | 33 | 1,732 |

Supplementary Table S3 continued

|  |  | East Coast Vancouver Island | | | | Southern mainland | | | | | | Fraser River | | |
| --- | --- | --- | --- | --- | --- | --- | --- | --- | --- | --- | --- | --- | --- | --- |
|  | CU | 27 | 83 | 27 | 29 | 28 | 20 | 20 | 20 | 20 | 3 | 8 | 11 | 9 |
| Year |  | Little Qualicum | Puntled summer | Punt fall | Quinsam | Phillips | Cheakamus | Ashlu | Shovelnose | Mamquam | Capilano | Nahatlatch | Nechako | Portage |
| 2013 | Brood | 1,054 | 186 | 300 | 1,370 | 71 | 9 | 0 | 0 | 0 | 337 | 0 | 0 | 0 |
|  | Received | 200 | 188 | 301 | 1,372 | 67 | 5 | 0 | 0 | 0 | 532 | 0 | 0 | 0 |
|  | Genotyped | 199 | 186 | 299 | 1,362 | 67 | 5 | 0 | 0 | 0 | 365 | 0 | 0 | 0 |
| 2014 | Brood | 1,013 | 280 | 757 | 1,111 | 58 | 72 | 3 | 23 | 18 | 398 | 0 | 0 | 0 |
|  | Received | 0 | 282 | 776 | 1,110 | 36 | 0 | 0 | 0 | 0 | 0 | 0 | 0 | 0 |
|  | Genotyped | 0 | 279 | 649 | 1,098 | 32 | 0 | 0 | 0 | 0 | 0 | 0 | 0 | 0 |
| 2015 | Brood | 1,214 | 164 | 784 | 1,323 | 57 | 63 | 2 | 21 | 16 | 507 | 0 | 0 | 0 |
|  | Received | 0 | 240 | 809 | 1,409 | 56 | 0 | 0 | 0 | 0 | 510 | 0 | 0 | 0 |
|  | Genotyped | 0 | 232 | 803 | 1,361 | 56 | 0 | 0 | 0 | 0 | 436 | 0 | 0 | 0 |
| 2016 | Brood | 1,051 | 128 | 968 | 1,557 | 44 | 62 | 4 | 14 | 22 | 494 | 0 | 3 | 0 |
|  | Received | 0 | 128 | 957 | 1,551 | 33 | 60 | 4 | 14 | 21 | 587 | 0 | 0 | 0 |
|  | Genotyped | 0 | 122 | 921 | 1,328 | 33 | 39 | 3 | 13 | 20 | 476 | 0 | 0 | 0 |
| 2017 | Brood | 1,375 | 226 | 890 | 1,471 | 37 | 62 | 12 | 8 | 20 | 441 | 0 | 0 | 0 |
|  | Received | 0 | 225 | 872 | 1,460 | 37 | 62 | 12 | 7 | 19 | 437 | 0 | 0 | 0 |
|  | Genotyped | 0 | 219 | 866 | 1,436 | 37 | 49 | 12 | 6 | 18 | 208 | 0 | 0 | 0 |
| 2018 | Brood | 1,325 | 295 | 820 | 1,415 | 39 | 74 | 16 | 22 | 16 | 433 | 0 | 3 | 0 |
|  | Received | 200 | 288 | 820 | 1,412 | 32 | 74 | 16 | 22 | 16 | 421 | 0 | 0 | 0 |
|  | Genotyped | 200 | 286 | 813 | 1,370 | 31 | 74 | 16 | 22 | 16 | 388 | 0 | 0 | 0 |
| 2019 | Brood | 1,287 | 153 | 926 | 1,351 | 40 | 52 | 12 | 15 | 14 | 424 | 4 | 0 | 37 |
|  | Received | 1,280 | 153 | 968 | 1,350 | 41 | 52 | 12 | 15 | 13 | 422 | 4 | 16 | 44 |
|  | Genotyped | 1,280 | 150 | 968 | 1,341 | 41 | 52 | 12 | 15 | 13 | 413 | 4 | 16 | 44 |

Supplementary Table S3 concluded

|  |  | Fraser River | | | | | | | | | | | |
| --- | --- | --- | --- | --- | --- | --- | --- | --- | --- | --- | --- | --- | --- |
|  | CU | 9006 | 3 | 9006 | 3 | 14 | 17 | 17 | 17 | 15 | 15 | 7 | 11 |
| Year |  | Chilliwack summer | Chilliwack fall | Chehalis summer | Harrison | Salmon (S Thompson) | Spius | Nicola | Coldwater | Shuswap lower | Shuswap middle | Maria Slough | Chilko |
| 2013 | Brood | 234 | 758 | 162 | 192 | 45 | 47 | 142 | 62 | 262 | 104 | 0 | 0 |
|  | Received | 0 | 741 | 89 | 138 | 42 | 45 | 130 | 41 | 239 | 103 | 0 | 0 |
|  | Genotyped | 0 | 690 | 86 | 108 | 42 | 45 | 35 | 41 | 144 | 103 | 0 | 0 |
| 2014 | Brood | 250 | 726 | 160 | 226 | 43 | 52 | 120 | 65 | 269 | 97 | 0 | 90 |
|  | Received | 0 | 720 | 0 | 225 | 0 | 52 | 119 | 63 | 244 | 101 | 0 | 89 |
|  | Genotyped | 0 | 698 | 0 | 212 | 0 | 52 | 119 | 63 | 243 | 101 | 0 | 88 |
| 2015 | Brood | 255 | 1,300 | 164 | 254 | 49 | 58 | 119 | 33 | 273 | 109 | 0 | 64 |
|  | Received | 0 | 887 | 0 | 245 | 0 | 58 | 113 | 33 | 251 | 107 | 0 | 62 |
|  | Genotyped | 0 | 874 | 0 | 240 | 0 | 58 | 110 | 33 | 169 | 105 | 0 | 62 |
| 2016 | Brood | 212 | 808 | 160 | 210 | 6 | 46 | 111 | 60 | 293 | 94 | 0 | 51 |
|  | Received | 0 | 806 | 0 | 216 | 0 | 0 | 112 | 0 | 260 | 92 | 0 | 52 |
|  | Genotyped | 0 | 769 | 0 | 213 | 0 | 0 | 107 | 0 | 253 | 88 | 0 | 50 |
| 2017 | Brood | 306 | 849 | 146 | 264 | 36 | 44 | 128 | 54 | 298 | 107 | 0 | 88 |
|  | Received | 0 | 836 | 0 | 264 | 0 | 0 | 131 | 0 | 257 | 105 | 0 | 88 |
|  | Genotyped | 0 | 782 | 0 | 255 | 0 | 0 | 105 | 0 | 209 | 93 | 0 | 67 |
| 2018 | Brood | 310 | 973 | 104 | 247 | 42 | 23 | 138 | 50 | 282 | 105 | 0 | 55 |
|  | Received | 304 | 973 | 109 | 244 | 42 | 25 | 143 | 50 | 278 | 102 | 0 | 55 |
|  | Genotyped | 297 | 970 | 102 | 210 | 42 | 19 | 140 | 49 | 278 | 102 | 0 | 55 |
| 2019 | Brood | 314 | 1,562 | 186 | 248 | 49 | 41 | 161 | 63 | 265 | 101 | 48 | 18 |
|  | Received | 312 | 1,561 | 190 | 248 | 46 | 42 | 162 | 63 | 260 | 100 | 47 | 18 |
|  | Genotyped | 312 | 1,514 | 190 | 242 | 46 | 42 | 162 | 63 | 260 | 99 | 45 | 4 |

Supplementary Table S4. Estimated percentage stock compositions by geographic region (US populations) or Conservation Unit (Canadian populations) for Chinook salmon fisheries sampled in British Columbia during 2018. N is number of individuals genotyped, and N PBT is the number of individuals in the sample subsequently identified via PBT. Standard deviation is in parentheses.

| Conservation Unit | Northern troll (Area 1, 101) | | | | | | Area 1 sport | | | |
| --- | --- | --- | --- | --- | --- | --- | --- | --- | --- | --- |
|  | July 11 | July 16 | July 24 | August 20 | August 26 | August 31 | June | July | August | Sept |
| N | 348 | 202 | 242 | 228 | 52 | 150 | 18 | 158 | 153 | 49 |
| N PBT | 9 | 13 | 25 | 23 | 8 | 6 | 2 | 20 | 38 | 5 |
| Southeast Alaska | 0.0 (0.1) | 0.0 (0.1) | 0.2 (0.5) | 0.4 (0.5) | 0.0 (0.0) | 0.0 (0.2) | 0.0 (0.0) | 0.0 (0.0) | 0.0 (0.0) | 0.0 (0.4) |
| Alsek | 0.0 (0.0) | 0.0 (0.0) | 0.0 (0.0) | 0.5 (0.4) | 3.8 (3.6) | 6.6 (2.0) | 0.0 (0.1) | 0.0 (0.0) | 0.0 (0.1) | 4.1 (2.9) |
| Unuk | 0.0 (0.0) | 0.0 (0.0) | 0.0 (0.1) | 0.4 (0.5) | 0.0 (0.1) | 0.0 (0.0) | 0.0 (0.0) | 0.0 (0.0) | 0.0 (0.0) | 0.0 (0.0) |
| Taku_early timing | 0.0 (0.0) | 0.0 (0.0) | 0.0 (0.3) | 0.0 (0.0) | 0.0 (0.1) | 0.0 (0.0) | 0.1 (1.1) | 0.0 (0.0) | 0.0 (0.0) | 0.0 (0.1) |
| Taku_mid timing | 0.0 (0.0) | 0.0 (0.2) | 0.0 (0.3) | 0.0 (0.1) | 0.0 (0.0) | 0.0 (0.0) | 0.0 (0.4) | 0.0 (0.0) | 0.0 (0.0) | 0.0 (0.0) |
| Taku_late timing | 0.0 (0.0) | 0.0 (0.0) | 0.0 (0.1) | 0.0 (0.0) | 0.0 (0.0) | 0.0 (0.0) | 0.0 (0.0) | 0.0 (0.0) | 0.0 (0.0) | 0.0 (0.0) |
| Stikine_early timing | 0.0 (0.0) | 0.0 (0.0) | 0.7 (0.6) | 0.0 (0.1) | 0.0 (0.0) | 0.0 (0.0) | 0.0 (0.1) | 0.0 (0.0) | 0.0 (0.2) | 0.0 (0.0) |
| Stikine_late timing | 0.0 (0.0) | 0.0 (0.0) | 0.0 (0.0) | 0.2 (0.5) | 0.0 (0.3) | 0.0 (0.0) | 5.6 (5.1) | 0.0 (0.0) | 0.0 (0.0) | 0.0 (0.3) |
| Haida Gwaii-North | 0.6 (0.4) | 0.5 (0.6) | 0.8 (0.5) | 0.9 (0.7) | 0.0 (0.0) | 0.0 (0.0) | 0.0 (0.0) | 1.3 (1.0) | 0.7 (1.0) | 0.0 (0.4) |
| Upper Nass | 0.3 (0.3) | 0.0 (0.1) | 0.0 (0.1) | 0.0 (0.0) | 0.0 (0.0) | 0.0 (0.0) | 0.0 (0.2) | 0.1 (0.3) | 0.0 (0.0) | 0.0 (0.1) |
| Portland Sound-Observatory Inlet-Lower Nass | 0.0 (0.0) | 1.0 (0.5) | 0.0 (0.0) | 0.2 (0.4) | 1.9 (1.9) | 0.0 (0.0) | 0.0 (0.1) | 0.1 (0.5) | 0.0 (0.0) | 0.0 (0.0) |
| Ecstall | 0.0 (0.0) | 0.0 (0.0) | 0.0 (0.0) | 0.0 (0.0) | 0.0 (0.0) | 0.0 (0.0) | 0.0 (0.0) | 0.0 (0.0) | 0.0 (0.0) | 0.0 (0.0) |
| Skeena Estuary | 0.0 (0.0) | 0.0 (0.0) | 0.0 (0.0) | 0.0 (0.0) | 0.0 (0.0) | 0.0 (0.1) | 0.0 (0.0) | 0.0 (0.0) | 0.0 (0.0) | 0.0 (0.0) |
| Lower Skeena | 0.0 (0.0) | 0.0 (0.0) | 0.0 (0.0) | 0.0 (0.0) | 0.0 (0.0) | 0.0 (0.1) | 0.0 (0.2) | 0.0 (0.1) | 0.0 (0.2) | 0.0 (0.2) |
| Kalum_early timing | 0.0 (0.0) | 0.0 (0.0) | 0.0 (0.0) | 0.0 (0.0) | 0.0 (0.0) | 0.0 (0.1) | 0.0 (0.0) | 0.0 (0.0) | 0.0 (0.0) | 0.0 (0.0) |
| Kalum_late timing | 0.0 (0.0) | 1.2 (0.7) | 0.0 (0.1) | 0.0 (0.0) | 0.0 (0.0) | 0.0 (0.0) | 16.7 (7.7) | 2.6 (1.0) | 0.7 (0.6) | 0.0 (0.0) |
| Zymoetz | 0.0 (0.0) | 0.0 (0.0) | 0.0 (0.0) | 0.0 (0.0) | 0.0 (0.1) | 0.0 (0.0) | 0.0 (0.5) | 0.0 (0.0) | 0.0 (0.0) | 0.0 (0.0) |
| Sicintine | 0.0 (0.1) | 0.0 (0.1) | 0.0 (0.0) | 0.0 (0.0) | 0.0 (0.1) | 0.0 (0.0) | 0.6 (1.7) | 0.0 (0.0) | 0.0 (0.0) | 0.0 (0.2) |
| Middle Skeena-mainstem tributaries | 0.0 (0.1) | 0.7 (0.8) | 0.2 (0.1) | 0.0 (0.0) | 0.0 (0.2) | 0.0 (0.0) | 0.8 (5.3) | 0.5 (0.8) | 0.0 (0.2) | 0.0 (0.3) |
| Middle Skeena-large lakes | 1.4 (0.6) | 0.5 (0.6) | 0.0 (0.0) | 0.0 (0.2) | 0.0 (0.0) | 0.0 (0.0) | 3.9 (4.8) | 0.7 (0.9) | 0.0 (0.0) | 0.0 (0.0) |
| Upper Skeena | 0.0 (0.1) | 0.2 (0.4) | 0.1 (0.3) | 0.0 (0.0) | 0.0 (0.0) | 0.0 (0.1) | 0.0 (0.3) | 0.5 (0.7) | 0.0 (0.0) | 0.0 (0.0) |
| Upper Bulkley River | 0.0 (0.0) | 0.0 (0.0) | 0.0 (0.0) | 0.0 (0.0) | 0.0 (0.0) | 0.0 (0.0) | 0.0 (0.0) | 0.0 (0.0) | 0.0 (0.0) | 0.0 (0.0) |
| North and Central Coast-late timing | 0.0 (0.0) | 0.0 (0.0) | 0.0 (0.0) | 0.0 (0.0) | 0.0 (0.0) | 0.7 (0.7) | 0.0 (0.4) | 0.0 (0.1) | 0.0 (0.0) | 0.4 (1.3) |
| North and Central Coast-early timing | 0.1 (0.1) | 0.0 (0.0) | 0.0 (0.0) | 0.2 (0.3) | 0.0 (0.4) | 1.3 (0.7) | 0.0 (0.1) | 0.4 (0.8) | 0.0 (0.0) | 0.0 (0.0) |
| Rivers Inlet | 0.1 (0.2) | 0.1 (0.2) | 0.0 (0.2) | 0.0 (0.0) | 0.0 (0.1) | 0.0 (0.0) | 5.6 (4.3) | 0.4 (0.6) | 0.0 (0.0) | 0.0 (0.1) |
| Wannock | 0.0 (0.0) | 0.0 (0.0) | 0.0 (0.0) | 0.0 (0.0) | 0.0 (0.1) | 0.0 (0.0) | 16.7 (7.6) | 1.3 (0.8) | 2.6 (1.2) | 0.0 (0.0) |
| Bella Coola-Bentinck | 0.0 (0.0) | 1.9 (1.0) | 0.0 (0.0) | 0.0 (0.2) | 0.0 (0.1) | 0.0 (0.0) | 0.0 (2.1) | 2.6 (1.6) | 0.0 (0.0) | 0.0 (0.2) |
| Dean River | 0.1 (0.1) | 0.0 (0.0) | 0.0 (0.0) | 0.0 (0.0) | 0.0 (0.2) | 0.0 (0.0) | 0.0 (0.2) | 0.2 (0.6) | 0.0 (0.1) | 0.0 (0.0) |
| Docee | 0.0 (0.0) | 0.0 (0.0) | 0.0 (0.0) | 0.0 (0.1) | 0.0 (0.0) | 0.0 (0.0) | 0.0 (0.0) | 0.0 (0.0) | 0.0 (0.0) | 0.0 (0.0) |
| Klinaklini_SU_1.3 | 0.3 (0.2) | 0.0 (0.0) | 0.0 (0.0) | 0.0 (0.0) | 0.0 (0.1) | 0.0 (0.0) | 0.0 (1.4) | 1.3 (0.9) | 0.7 (0.6) | 0.0 (0.0) |
| Southern Mainland-Southern Fjords_FA_0.x | 0.0 (0.0) | 0.3 (0.4) | 0.0 (0.0) | 0.0 (0.0) | 0.0 (0.1) | 0.0 (0.1) | 0.0 (0.4) | 0.6 (0.6) | 0.0 (0.0) | 0.0 (0.0) |
| Southern Mainland-Georgia Strait_FA_0.x | 0.0 (0.1) | 0.0 (0.2) | 0.0 (0.1) | 0.0 (0.2) | 0.0 (0.1) | 0.0 (0.1) | 0.0 (0.7) | 0.0 (0.1) | 0.0 (0.1) | 0.0 (0.1) |
| Upper Fraser River_SP_1.3 | 0.0 (0.1) | 0.0 (0.2) | 0.0 (0.0) | 0.0 (0.0) | 0.0 (0.1) | 0.5 (0.6) | 0.0 (0.5) | 0.0 (0.2) | 0.0 (0.2) | 0.0 (0.4) |
| Middle Fraser River_SU_1.3 | 1.4 (0.6) | 0.0 (0.0) | 0.1 (0.2) | 0.0 (0.0) | 0.0 (0.3) | 0.1 (0.4) | 0.0 (0.5) | 0.0 (0.0) | 0.0 (0.0) | 0.0 (0.5) |
| Middle Fraser River_SP_1.3 | 0.0 (0.1) | 1.1 (0.8) | 0.3 (0.4) | 0.0 (0.1) | 0.0 (0.1) | 0.0 (0.2) | 0.0 (0.3) | 0.0 (0.0) | 0.0 (0.1) | 0.0 (0.6) |
| Middle Fraser River-Portage_FA_1.3 | 0.0 (0.0) | 0.0 (0.0) | 0.0 (0.0) | 0.0 (0.0) | 0.0 (0.0) | 0.0 (0.0) | 0.0 (0.0) | 0.0 (0.0) | 0.0 (0.0) | 0.0 (0.0) |
| Middle Fraser-Fraser Canyon_SP_1.3 | 0.1 (0.2) | 0.0 (0.0) | 0.0 (0.0) | 0.0 (0.0) | 0.0 (0.0) | 0.0 (0.0) | 0.0 (0.0) | 0.0 (0.0) | 0.0 (0.0) | 0.0 (0.0) |
| North Thompson_SP_1.3 | 0.0 (0.0) | 0.0 (0.0) | 0.0 (0.0) | 0.0 (0.0) | 0.0 (0.0) | 0.0 (0.0) | 0.0 (0.1) | 0.0 (0.0) | 0.0 (0.0) | 0.0 (0.0) |
| North Thompson_SU_1.3 | 0.6 (0.4) | 0.1 (0.2) | 0.0 (0.0) | 0.0 (0.1) | 0.0 (0.5) | 0.0 (0.0) | 0.0 (0.6) | 0.6 (0.7) | 0.0 (0.0) | 0.0 (0.1) |
| Shuswap River_SU_0.3 | 3.0 (0.9) | 3.7 (1.4) | 1.8 (0.7) | 0.0 (0.0) | 0.0 (0.1) | 0.0 (0.0) | 11.1 (6.6) | 5.1 (1.8) | 0.0 (0.1) | 0.0 (0.0) |
| South Thompson-Bessette Creek_SU_1.2 | 0.0 (0.0) | 0.0 (0.0) | 0.0 (0.0) | 0.0 (0.0) | 0.0 (0.0) | 0.0 (0.0) | 0.0 (0.1) | 0.0 (0.0) | 0.0 (0.1) | 0.0 (0.0) |
| South Thompson_SU_0.3 | 20.4 (2.1) | 25.1 (3.1) | 25.4 (3.1) | 7.8 (1.8) | 1.9 (2.5) | 4.0 (1.7) | 11.1 (6.0) | 15.1 (2.4) | 9.8 (2.5) | 0.0 (0.0) |
| South Thompson_SU_1.3 | 0.0 (0.0) | 0.0 (0.0) | 0.0 (0.1) | 0.0 (0.2) | 0.0 (0.0) | 0.0 (0.0) | 0.0 (0.5) | 0.0 (0.0) | 0.0 (0.0) | 0.0 (0.0) |
| Lower Thompson_SP_1.2 | 0.0 (0.0) | 0.0 (0.0) | 0.0 (0.0) | 0.0 (0.0) | 0.0 (0.1) | 0.0 (0.1) | 0.0 (0.3) | 0.0 (0.2) | 0.0 (0.2) | 0.0 (0.1) |
| Lower Fraser River_SP_1.3 | 0.0 (0.0) | 0.0 (0.0) | 0.0 (0.0) | 0.0 (0.0) | 0.0 (0.0) | 0.0 (0.0) | 0.0 (0.0) | 0.0 (0.0) | 0.0 (0.0) | 0.0 (0.0) |
| Lower Fraser River_SU_1.3 | 0.0 (0.0) | 0.0 (0.0) | 0.0 (0.0) | 0.0 (0.0) | 0.0 (0.4) | 0.0 (0.0) | 0.0 (0.0) | 0.0 (0.0) | 1.3 (1.0) | 0.0 (0.0) |
| Lower Fraser River-Upper Pitt_SU_1.3 | 0.0 (0.0) | 0.0 (0.0) | 0.0 (0.0) | 0.0 (0.0) | 0.0 (0.4) | 0.0 (0.0) | 0.0 (0.6) | 0.6 (0.7) | 0.0 (0.0) | 0.0 (0.2) |
| Maria Slough_SU_0.3 | 0.0 (0.0) | 0.0 (0.0) | 0.0 (0.0) | 0.0 (0.1) | 0.0 (0.0) | 0.0 (0.0) | 0.0 (0.0) | 0.0 (0.0) | 0.0 (0.0) | 0.0 (0.0) |
| Lower Fraser River_FA_0.3 | 0.3 (0.3) | 0.5 (0.4) | 0.8 (0.7) | 0.9 (0.5) | 1.9 (1.9) | 1.3 (1.1) | 0.0 (0.1) | 0.0 (0.0) | 0.7 (0.8) | 0.0 (0.4) |
| East Vancouver Island-North_FA_0.x | 0.0 (0.0) | 0.6 (0.6) | 0.8 (0.6) | 0.5 (0.3) | 0.0 (0.0) | 0.9 (0.7) | 16.7 (7.8) | 1.3 (0.9) | 3.3 (1.3) | 0.0 (0.8) |
| East Vancouver Island-Qualicum and Puntledge_FA_0.x | 0.5 (0.3) | 0.3 (0.5) | 2.0 (1.0) | 0.5 (0.7) | 1.9 (1.4) | 2.6 (1.8) | 0.0 (0.5) | 1.9 (1.1) | 0.0 (0.0) | 0.0 (0.0) |
| East Vancouver Island-Nanaimo and Chemainus_FA_0.x | 0.0 (0.0) | 0.3 (0.4) | 0.0 (0.2) | 0.0 (0.2) | 0.0 (0.0) | 0.0 (0.0) | 0.0 (0.0) | 0.0 (0.0) | 0.0 (0.0) | 0.0 (0.0) |
| East Vancouver Island-Nanaimo_SP_1.x | 0.0 (0.0) | 0.0 (0.0) | 0.0 (0.0) | 0.0 (0.0) | 0.0 (0.0) | 0.0 (0.0) | 0.0 (0.0) | 0.0 (0.0) | 0.0 (0.0) | 0.0 (0.1) |
| East Vancouver Island-Georgia Strait_SU_0.3 | 0.0 (0.0) | 0.0 (0.0) | 0.0 (0.0) | 0.0 (0.0) | 0.0 (0.2) | 0.0 (0.0) | 0.0 (0.0) | 0.0 (0.0) | 0.0 (0.0) | 0.0 (0.4) |
| East Vancouver Island-Cowichan and Koksilah_FA_0.x | 0.0 (0.1) | 0.5 (0.7) | 0.5 (0.5) | 0.2 (0.1) | 0.0 (0.0) | 0.3 (0.5) | 0.0 (0.0) | 0.0 (0.0) | 0.0 (0.0) | 0.0 (0.0) |
| West Vancouver Island-Nootka and Kyuquot_FA_0.x | 0.0 (0.1) | 0.2 (0.3) | 1.1 (1.1) | 0.6 (1.0) | 0.0 (0.1) | 0.0 (0.6) | 0.0 (1.6) | 4.9 (2.0) | 0.8 (1.5) | 2.2 (2.5) |
| West Vancouver Island-North_FA_0.x | 0.0 (0.0) | 0.0 (0.0) | 0.0 (0.0) | 0.0 (0.0) | 0.0 (0.0) | 0.0 (0.0) | 0.0 (0.4) | 0.0 (0.0) | 0.0 (0.1) | 1.6 (1.7) |
| West Vancouver Island-South_FA_0.x | 4.3 (1.0) | 9.3 (2.3) | 13.6 (2.7) | 18.2 (2.6) | 25.0 (6.6) | 5.3 (1.5) | 0.0 (1.3) | 21.7 (3.3) | 39.1 (3.2) | 18.2 (5.3) |
| Okanagan_1.x | 0.0 (0.0) | 1.9 (1.3) | 0.8 (1.6) | 1.2 (1.6) | 0.0 (0.0) | 0.2 (0.8) | 0.1 (0.7) | 0.0 (0.0) | 2.6 (1.5) | 1.5 (3.2) |
| Juan de Fuca | 0.0 (0.0) | 0.5 (0.4) | 0.0 (0.0) | 0.0 (0.0) | 0.0 (0.0) | 0.0 (0.0) | 0.0 (0.0) | 0.0 (0.0) | 0.0 (0.0) | 0.0 (0.0) |
| Coastal Washington | 17.1 (1.9) | 18.5 (2.9) | 15.5 (2.4) | 20.3 (2.2) | 17.8 (4.8) | 27.5 (3.9) | 5.6 (4.6) | 8.2 (2.1) | 15.0 (3.0) | 29.6 (6.7) |
| North Puget Sound | 0.0 (0.2) | 0.3 (0.5) | 0.0 (0.0) | 0.0 (0.0) | 1.4 (1.8) | 1.3 (1.2) | 0.0 (0.4) | 0.0 (0.1) | 0.0 (0.0) | 0.0 (0.3) |
| South Puget Sound | 2.0 (0.9) | 2.2 (1.0) | 2.1 (0.8) | 0.0 (0.0) | 0.3 (0.5) | 0.8 (1.0) | 0.0 (1.5) | 0.0 (0.0) | 0.0 (0.0) | 0.0 (0.3) |
| Lower Columbia River | 5.5 (1.6) | 5.9 (1.7) | 4.3 (1.2) | 1.7 (0.7) | 1.9 (2.6) | 1.9 (1.2) | 0.0 (0.0) | 5.7 (1.9) | 1.9 (1.0) | 4.1 (2.7) |
| Mid Columbia River_SP | 0.0 (0.0) | 0.0 (0.0) | 0.0 (0.0) | 0.0 (0.0) | 0.2 (0.8) | 0.5 (0.6) | 0.0 (0.1) | 0.0 (0.1) | 0.0 (0.0) | 0.0 (0.0) |
| Upper Columbia River_SP | 0.0 (0.1) | 0.0 (0.0) | 0.0 (0.0) | 0.0 (0.0) | 0.0 (0.0) | 0.0 (0.2) | 0.0 (0.1) | 0.0 (0.0) | 0.0 (0.0) | 0.0 (0.0) |
| Upper Columbia River_SU_FA | 10.5 (1.6) | 8.7 (2.4) | 15.7 (3.2) | 15.6 (2.8) | 11.5 (5.4) | 13.2 (3.3) | 4.4 (4.6) | 11.6 (2.7) | 8.1 (2.8) | 16.8 (6.8) |
| Snake River_FA | 3.5 (1.1) | 3.5 (1.7) | 1.2 (1.7) | 6.4 (1.7) | 0.0 (0.6) | 0.0 (0.0) | 1.1 (2.1) | 3.6 (1.6) | 6.3 (2.4) | 0.0 (0.0) |
| Snake River_SP_SU | 0.0 (0.0) | 0.0 (0.0) | 0.0 (0.0) | 0.4 (0.4) | 0.0 (0.2) | 0.8 (0.8) | 0.0 (1.1) | 0.0 (0.1) | 0.0 (0.1) | 0.0 (0.2) |
| North & Central Oregon | 20.0 (2.3) | 7.9 (1.6) | 4.1 (1.1) | 12.2 (2.3) | 11.3 (5.6) | 18.9 (3.4) | 0.0 (1.4) | 4.2 (1.8) | 1.3 (1.0) | 0.4 (1.8) |
| Upper Willamette River | 0.1 (0.2) | 0.6 (0.5) | 1.1 (0.8) | 0.9 (0.6) | 0.0 (0.0) | 0.9 (0.6) | 0.0 (0.0) | 0.0 (0.1) | 0.0 (0.3) | 0.0 (0.0) |
| South Oregon coastal | 7.3 (1.7) | 1.8 (1.0) | 6.6 (1.8) | 9.4 (1.8) | 19.0 (6.5) | 10.0 (2.7) | 0.0 (0.4) | 2.7 (1.6) | 5.3 (1.3) | 20.9 (6.7) |
| California Klamath Trinity | 0.0 (0.0) | 0.0 (0.0) | 0.0 (0.1) | 0.0 (0.0) | 0.0 (0.0) | 0.0 (0.0) | 0.0 (0.7) | 0.0 (0.0) | 0.0 (0.0) | 0.0 (0.0) |
| California Central Valley_Fall | 0.0 (0.0) | 0.0 (0.0) | 0.0 (0.0) | 0.0 (0.0) | 0.0 (0.0) | 0.0 (0.0) | 0.0 (0.7) | 0.0 (0.0) | 0.0 (0.0) | 0.0 (0.0) |
| California Central Valley_Spring | 0.0 (0.0) | 0.0 (0.0) | 0.0 (0.0) | 0.0 (0.0) | 0.0 (0.0) | 0.0 (0.0) | 0.0 (0.3) | 0.0 (0.1) | 0.0 (0.0) | 0.0 (0.1) |
| Coastal California | 0.0 (0.0) | 0.0 (0.0) | 0.0 (0.0) | 0.0 (0.0) | 0.0 (0.0) | 0.0 (0.0) | 0.0 (0.0) | 0.0 (0.0) | 0.0 (0.0) | 0.0 (0.0) |

Supplementary Table S4 continued

| Conservation Unit | Area 3/4 sport | | Tyee test | Area 11 sport | | | Area 12 sport | | |
| --- | --- | --- | --- | --- | --- | --- | --- | --- | --- |
|  | July | August | July | June | July | August | June | July | August |
| Sample size | 10 | 31 | 58 | 57 | 57 | 59 | 94 | 141 | 91 |
| N PBT | 4 | 8 | 3 | 20 | 15 | 27 | 21 | 29 | 28 |
| Southeast Alaska | 0.0 (0.1) | 0.0 (0.3) | 0.0 (0.0) | 0.0 (0.2) | 0.0 (0.0) | 0.0 (0.2) | 0.0 (0.1) | 0.0 (0.1) | 0.0 (0.1) |
| Alsek | 0.0 (1.0) | 0.0 (0.1) | 0.0 (0.2) | 0.0 (0.2) | 0.0 (0.1) | 0.0 (0.0) | 0.0 (0.1) | 0.0 (0.1) | 0.0 (0.0) |
| Unuk | 0.0 (0.0) | 0.1 (0.0) | 0.0 (0.1) | 0.0 (0.2) | 0.0 (0.0) | 0.0 (0.0) | 0.0 (0.0) | 0.0 (0.1) | 0.0 (0.0) |
| Taku_early timing | 0.0 (0.3) | 0.0 (0.0) | 0.0 (0.0) | 0.0 (0.2) | 0.0 (0.0) | 0.0 (0.1) | 0.0 (0.1) | 0.0 (0.1) | 0.0 (0.0) |
| Taku_mid timing | 0.0 (0.0) | 0.0 (0.0) | 0.0 (0.1) | 0.0 (0.0) | 0.0 (0.0) | 0.0 (0.2) | 0.0 (0.1) | 0.0 (0.0) | 0.0 (0.0) |
| Taku_late timing | 0.0 (0.2) | 0.0 (0.0) | 0.0 (0.0) | 0.0 (0.0) | 0.0 (0.0) | 0.0 (0.0) | 0.0 (0.0) | 0.0 (0.0) | 0.0 (0.0) |
| Stikine_early timing | 0.0 (0.1) | 0.0 (0.0) | 0.0 (0.1) | 0.0 (0.1) | 0.0 (0.1) | 0.0 (0.0) | 0.0 (0.1) | 0.0 (0.0) | 0.0 (0.0) |
| Stikine_late timing | 0.0 (0.0) | 0.0 (0.0) | 0.0 (0.0) | 0.0 (0.4) | 0.0 (0.2) | 0.0 (0.1) | 0.3 (0.6) | 0.0 (0.0) | 0.0 (0.0) |
| Haida Gwaii-North | 0.0 (0.0) | 0.0 (0.0) | 0.0 (0.0) | 0.0 (0.0) | 0.0 (0.0) | 0.0 (0.0) | 0.0 (0.0) | 0.0 (0.0) | 0.0 (0.0) |
| Upper Nass | 0.0 (0.0) | 3.2 (3.4) | 0.0 (0.1) | 0.0 (0.8) | 0.0 (0.1) | 0.0 (0.0) | 0.0 (0.1) | 0.0 (0.1) | 0.0 (0.1) |
| Portland Sound-Observatory Inlet-Lower Nass | 0.0 (0.3) | 3.1 (2.8) | 0.0 (0.2) | 0.0 (0.0) | 0.0 (0.0) | 0.0 (0.3) | 0.0 (0.0) | 0.0 (0.0) | 0.0 (0.0) |
| Ecstall | 0.0 (0.9) | 3.2 (2.6) | 1.7 (1.4) | 0.0 (0.0) | 0.0 (0.0) | 0.0 (0.0) | 0.0 (0.0) | 0.0 (0.0) | 0.0 (0.0) |
| Skeena Estuary | 10.0 (8.7) | 0.0 (0.0) | 0.0 (0.0) | 0.0 (0.0) | 0.0 (0.0) | 0.0 (0.0) | 0.0 (0.0) | 0.0 (0.0) | 0.0 (0.0) |
| Lower Skeena | 0.0 (1.0) | 0.0 (1.2) | 0.0 (0.0) | 0.0 (0.4) | 0.0 (0.0) | 0.0 (0.1) | 0.1 (0.3) | 0.0 (0.1) | 0.0 (0.1) |
| Kalum_early timing | 0.0 (0.0) | 0.0 (0.2) | 0.0 (0.0) | 0.0 (0.0) | 0.0 (0.0) | 0.0 (0.0) | 0.0 (0.0) | 0.0 (0.0) | 0.0 (0.0) |
| Kalum_late timing | 0.0 (0.0) | 0.0 (0.0) | 30.4 (6.9) | 0.0 (0.0) | 0.0 (0.1) | 0.0 (0.0) | 0.0 (0.4) | 0.5 (0.6) | 0.0 (0.0) |
| Zymoetz | 0.0 (0.0) | 0.0 (0.0) | 4.2 (3.0) | 0.1 (0.2) | 0.0 (0.0) | 0.0 (0.0) | 0.6 (1.0) | 0.2 (0.5) | 0.0 (0.0) |
| Sicintine | 0.0 (0.0) | 0.0 (0.0) | 0.2 (1.1) | 0.0 (0.0) | 0.0 (0.1) | 0.0 (0.0) | 0.0 (0.0) | 0.0 (0.0) | 0.0 (0.0) |
| Middle Skeena-mainstem tributaries | 0.0 (0.1) | 0.1 (0.7) | 0.1 (0.2) | 0.0 (0.1) | 0.0 (0.0) | 0.0 (0.0) | 0.0 (0.0) | 0.0 (0.0) | 0.0 (0.0) |
| Middle Skeena-large lakes | 0.0 (0.0) | 0.0 (0.1) | 62.7 (8.4) | 1.7 (1.7) | 0.0 (0.0) | 0.0 (0.0) | 0.0 (0.1) | 0.0 (0.0) | 0.0 (0.0) |
| Upper Skeena | 0.0 (0.1) | 0.0 (0.3) | 0.8 (2.8) | 0.0 (0.0) | 0.0 (0.0) | 0.0 (0.0) | 0.0 (0.0) | 0.0 (0.0) | 0.0 (0.0) |
| Upper Bulkley River | 0.0 (0.0) | 0.0 (0.1) | 0.0 (0.1) | 0.0 (0.0) | 0.7 (1.0) | 0.0 (0.0) | 1.5 (1.5) | 0.7 (0.7) | 0.1 (0.1) |
| North and Central Coast-late timing | 0.0 (0.0) | 9.5 (5.1) | 0.0 (0.0) | 0.4 (0.6) | 0.0 (0.2) | 0.0 (0.2) | 0.0 (0.1) | 0.0 (0.0) | 0.0 (0.1) |
| North and Central Coast-early timing | 0.0 (0.2) | 0.1 (1.3) | 0.0 (0.2) | 5.2 (3.2) | 0.0 (0.0) | 0.6 (1.2) | 6.0 (2.6) | 0.2 (0.7) | 0.0 (0.0) |
| Rivers Inlet | 0.0 (0.1) | 0.0 (0.1) | 0.0 (0.1) | 0.0 (0.1) | 0.0 (0.0) | 0.0 (0.0) | 0.0 (0.0) | 0.0 (0.0) | 0.0 (0.0) |
| Wannock | 0.0 (0.1) | 3.2 (3.2) | 0.0 (0.0) | 0.0 (0.1) | 0.0 (0.0) | 0.5 (1.0) | 1.1 (1.0) | 0.0 (0.0) | 0.0 (0.3) |
| Bella Coola-Bentinck | 0.0 (0.9) | 0.0 (0.3) | 0.0 (0.0) | 0.0 (0.2) | 0.0 (0.0) | 0.0 (0.0) | 0.0 (0.1) | 0.0 (0.1) | 0.0 (0.0) |
| Dean River | 0.0 (0.3) | 0.0 (0.4) | 0.0 (0.1) | 0.0 (0.0) | 0.0 (0.0) | 0.0 (0.0) | 0.0 (0.0) | 0.0 (0.0) | 0.0 (0.1) |
| Docee | 0.0 (0.0) | 0.0 (0.0) | 0.0 (0.0) | 1.3 (2.4) | 0.0 (0.1) | 0.5 (1.9) | 9.6 (3.1) | 2.8 (1.6) | 1.1 (1.0) |
| Klinaklini_SU_1.3 | 0.0 (0.0) | 0.0 (0.0) | 0.0 (0.0) | 3.5 (2.5) | 0.0 (0.0) | 0.0 (0.4) | 4.3 (2.2) | 0.0 (0.0) | 0.0 (0.0) |
| Southern Mainland-Southern Fjords_FA_0.x | 0.0 (0.0) | 0.0 (0.0) | 0.0 (0.0) | 0.0 (0.1) | 0.0 (0.3) | 0.0 (0.2) | 3.5 (2.0) | 2.0 (1.2) | 0.0 (0.1) |
| Southern Mainland-Georgia Strait_FA_0.x | 0.0 (0.6) | 0.0 (1.9) | 0.0 (0.0) | 0.0 (0.1) | 0.0 (0.5) | 0.0 (0.3) | 0.2 (0.8) | 0.0 (0.3) | 0.0 (0.1) |
| Upper Fraser River_SP_1.3 | 0.0 (0.8) | 0.0 (0.3) | 0.0 (0.2) | 0.0 (0.3) | 0.0 (0.2) | 0.0 (0.4) | 0.6 (1.0) | 0.7 (0.8) | 0.0 (0.2) |
| Middle Fraser River_SU_1.3 | 0.0 (2.5) | 0.0 (0.3) | 0.0 (0.0) | 0.0 (0.0) | 0.0 (0.4) | 0.0 (0.2) | 2.4 (2.0) | 0.0 (0.1) | 0.0 (0.3) |
| Middle Fraser River_SP_1.3 | 0.0 (1.3) | 0.0 (0.2) | 0.0 (0.1) | 0.0 (0.0) | 0.0 (0.2) | 0.0 (0.0) | 0.0 (0.0) | 0.0 (0.0) | 0.0 (0.0) |
| Middle Fraser River-Portage_FA_1.3 | 0.0 (0.0) | 0.0 (0.0) | 0.0 (0.0) | 0.0 (0.0) | 0.0 (0.0) | 0.0 (0.0) | 0.0 (0.0) | 0.0 (0.0) | 0.0 (0.0) |
| Middle Fraser-Fraser Canyon_SP_1.3 | 0.0 (0.0) | 0.0 (0.0) | 0.0 (0.0) | 0.0 (0.0) | 0.0 (0.1) | 0.0 (0.0) | 0.0 (0.0) | 0.0 (0.0) | 0.0 (0.0) |
| North Thompson_SP_1.3 | 0.0 (0.0) | 0.0 (0.2) | 0.0 (0.0) | 0.0 (0.0) | 0.0 (0.1) | 0.0 (0.3) | 0.0 (0.1) | 0.0 (0.2) | 1.1 (1.2) |
| North Thompson_SU_1.3 | 0.0 (1.7) | 0.0 (0.0) | 0.0 (0.0) | 0.0 (0.0) | 0.0 (0.0) | 0.0 (0.6) | 4.3 (2.0) | 12.4 (3.2) | 4.4 (1.8) |
| Shuswap River_SU_0.3 | 10.0 (7.5) | 0.0 (0.0) | 0.0 (0.2) | 0.0 (0.0) | 0.0 (0.0) | 0.0 (0.0) | 0.0 (0.0) | 0.0 (0.0) | 0.0 (0.0) |
| South Thompson-Bessette Creek_SU_1.2 | 0.0 (0.5) | 0.0 (0.0) | 0.0 (0.0) | 0.0 (0.5) | 1.8 (2.0) | 0.0 (0.0) | 2.1 (1.5) | 6.0 (2.2) | 21.6 (3.8) |
| South Thompson_SU_0.3 | 0.0 (0.0) | 6.5 (4.4) | 0.0 (0.1) | 0.0 (0.1) | 0.0 (0.0) | 0.0 (0.0) | 0.0 (0.0) | 0.0 (0.0) | 0.0 (0.0) |
| South Thompson_SU_1.3 | 0.0 (0.0) | 0.0 (0.3) | 0.0 (0.1) | 0.0 (0.4) | 0.0 (0.1) | 0.0 (0.1) | 0.0 (0.1) | 0.0 (0.1) | 0.0 (0.1) |
| Lower Thompson_SP_1.2 | 0.0 (0.0) | 0.0 (0.5) | 0.0 (0.0) | 0.0 (0.0) | 0.0 (0.0) | 0.0 (0.0) | 0.0 (0.0) | 0.7 (0.8) | 0.0 (0.1) |
| Lower Fraser River_SP_1.3 | 0.0 (0.0) | 0.0 (1.2) | 0.0 (0.0) | 0.0 (0.3) | 0.0 (0.3) | 0.0 (0.0) | 0.0 (0.1) | 0.0 (0.0) | 0.0 (0.3) |
| Lower Fraser River_SU_1.3 | 0.0 (1.0) | 0.0 (0.0) | 0.0 (0.0) | 3.5 (2.2) | 0.0 (0.0) | 0.0 (0.0) | 1.1 (0.9) | 0.0 (0.1) | 0.0 (0.1) |
| Lower Fraser River-Upper Pitt_SU_1.3 | 0.0 (0.1) | 0.0 (0.0) | 0.0 (0.0) | 0.0 (0.0) | 0.0 (0.0) | 0.0 (0.0) | 0.0 (0.0) | 0.0 (0.0) | 0.4 (1.1) |
| Maria Slough_SU_0.3 | 0.0 (0.0) | 0.0 (0.3) | 0.0 (0.0) | 0.0 (0.0) | 0.0 (0.1) | 0.0 (0.0) | 1.1 (0.9) | 4.3 (1.7) | 2.2 (2.0) |
| Lower Fraser River_FA_0.3 | 0.0 (0.5) | 3.2 (2.6) | 0.0 (0.0) | 5.3 (3.6) | 5.3 (3.3) | 10.2 (2.7) | 3.2 (1.7) | 5.7 (1.8) | 8.8 (2.9) |
| East Vancouver Island-North_FA_0.x | 0.0 (0.2) | 12.9 (5.8) | 0.0 (0.0) | 5.3 (2.2) | 0.0 (0.1) | 2.2 (2.3) | 8.8 (2.7) | 9.9 (2.8) | 11.0 (3.1) |
| East Vancouver Island-Qualicum and Puntledge_FA_0.x | 29.9 (12.3) | 6.5 (4.1) | 0.0 (0.0) | 0.0 (0.0) | 0.0 (0.0) | 0.3 (1.4) | 0.0 (0.0) | 0.0 (0.0) | 0.0 (0.0) |
| East Vancouver Island-Nanaimo and Chemainus_FA_0.x | 0.0 (0.0) | 0.0 (0.0) | 0.0 (0.0) | 0.0 (0.0) | 0.0 (0.0) | 0.0 (0.0) | 0.0 (0.0) | 0.0 (0.0) | 0.0 (0.3) |
| East Vancouver Island-Nanaimo_SP_1.x | 0.0 (0.0) | 0.0 (0.0) | 0.0 (0.0) | 0.0 (0.2) | 0.0 (0.0) | 0.9 (1.3) | 2.7 (1.9) | 1.5 (1.5) | 0.0 (0.1) |
| East Vancouver Island-Georgia Strait_SU_0.3 | 0.0 (0.1) | 0.0 (0.2) | 0.0 (0.0) | 32.1 (5.4) | 36.8 (5.6) | 1.7 (1.3) | 12.7 (2.7) | 9.2 (2.4) | 1.4 (1.2) |
| East Vancouver Island-Cowichan and Koksilah_FA_0.x | 0.1 (0.2) | 0.0 (0.7) | 0.0 (0.0) | 0.0 (0.0) | 1.0 (1.3) | 0.0 (0.8) | 0.7 (0.8) | 0.1 (0.1) | 0.0 (0.0) |
| West Vancouver Island-Nootka and Kyuquot_FA_0.x | 0.0 (0.4) | 2.2 (2.4) | 0.0 (0.3) | 32.8 (5.6) | 31.7 (6.8) | 78.0 (4.7) | 15.0 (3.9) | 22.0 (3.3) | 33.7 (5.0) |
| West Vancouver Island-North_FA_0.x | 0.0 (0.0) | 0.0 (0.0) | 0.0 (0.1) | 0.0 (0.0) | 0.5 (0.6) | 0.0 (0.0) | 0.0 (0.0) | 0.0 (0.0) | 0.6 (1.1) |
| West Vancouver Island-South_FA_0.x | 10.0 (8.6) | 13.9 (7.0) | 0.0 (0.2) | 0.0 (0.1) | 0.0 (0.0) | 0.0 (0.0) | 3.2 (1.6) | 0.7 (0.6) | 0.0 (0.0) |
| Okanagan_1.x | 6.9 (7.2) | 0.0 (0.0) | 0.0 (0.0) | 0.0 (0.0) | 0.0 (0.1) | 0.0 (0.1) | 0.0 (0.0) | 0.0 (0.0) | 1.1 (1.1) |
| Juan de Fuca | 0.0 (0.0) | 0.0 (0.0) | 0.0 (0.0) | 0.0 (0.0) | 5.5 (3.6) | 1.2 (1.4) | 4.9 (4.2) | 7.6 (2.1) | 5.0 (2.9) |
| Coastal Washington | 0.0 (0.0) | 0.0 (0.2) | 0.0 (0.1) | 0.0 (0.0) | 0.0 (0.0) | 0.2 (0.5) | 0.0 (0.0) | 0.0 (0.0) | 0.5 (1.0) |
| North Puget Sound | 15.9 (13.0) | 15.1 (6.9) | 0.0 (0.1) | 3.5 (2.1) | 14.0 (3.4) | 2.9 (1.8) | 4.1 (2.2) | 7.6 (2.3) | 4.4 (2.2) |
| South Puget Sound | 4.1 (5.0) | 1.0 (5.0) | 0.0 (0.1) | 0.0 (0.0) | 1.5 (1.1) | 0.7 (1.2) | 3.9 (2.9) | 1.2 (1.2) | 0.0 (0.0) |
| Lower Columbia River | 9.8 (8.1) | 0.0 (0.0) | 0.0 (0.0) | 0.0 (0.1) | 0.0 (0.1) | 0.0 (0.1) | 0.0 (0.0) | 0.0 (0.0) | 0.0 (0.1) |
| Mid Columbia River_SP | 0.1 (0.0) | 0.0 (0.0) | 0.0 (0.1) | 0.1 (0.4) | 1.3 (1.8) | 0.0 (0.0) | 2.1 (1.2) | 3.3 (1.8) | 1.1 (1.7) |
| Upper Columbia River_SP | 0.0 (0.3) | 0.0 (0.3) | 0.0 (0.2) | 1.7 (1.4) | 0.0 (0.6) | 0.0 (0.0) | 0.0 (0.0) | 0.3 (0.4) | 0.5 (1.6) |
| Upper Columbia River_SU_FA | 3.1 (4.0) | 12.7 (6.0) | 0.0 (0.0) | 0.0 (0.4) | 0.0 (0.1) | 0.0 (0.3) | 0.0 (0.1) | 0.0 (0.0) | 0.0 (0.0) |
| Snake River_FA | 0.0 (0.0) | 0.2 (2.7) | 0.0 (0.0) | 0.0 (0.4) | 0.0 (0.0) | 0.0 (0.0) | 0.0 (0.2) | 0.0 (0.0) | 1.1 (1.2) |
| Snake River_SP_SU | 0.0 (2.5) | 0.0 (0.5) | 0.0 (0.3) | 3.5 (2.7) | 0.0 (0.1) | 0.0 (0.1) | 0.0 (0.1) | 0.7 (0.6) | 0.0 (0.0) |
| North & Central Oregon | 0.0 (1.0) | 3.2 (2.8) | 0.0 (0.3) | 0.0 (0.2) | 0.0 (0.1) | 0.0 (0.7) | 0.0 (0.1) | 0.0 (0.0) | 0.0 (0.1) |
| Upper Willamette River | 0.1 (0.1) | 0.0 (0.0) | 0.0 (0.1) | 0.0 (0.9) | 0.0 (0.0) | 0.0 (0.1) | 0.0 (0.0) | 0.0 (0.0) | 0.0 (0.0) |
| South Oregon coastal | 0.0 (0.4) | 0.0 (0.4) | 0.0 (0.2) | 0.0 (0.1) | 0.0 (0.2) | 0.0 (0.0) | 0.0 (0.1) | 0.0 (0.1) | 0.0 (0.1) |
| California Klamath Trinity | 0.0 (0.0) | 0.0 (0.1) | 0.0 (0.0) | 0.0 (0.1) | 0.0 (0.1) | 0.0 (0.0) | 0.0 (0.1) | 0.0 (0.0) | 0.0 (0.1) |
| California Central Valley_Fall | 0.0 (2.1) | 0.0 (0.1) | 0.0 (0.5) | 0.0 (0.0) | 0.0 (0.1) | 0.0 (0.0) | 0.0 (0.0) | 0.0 (0.0) | 0.0 (0.0) |
| California Central Valley_Spring | 0.0 (0.5) | 0.0 (0.0) | 0.0 (0.0) | 0.0 (0.2) | 0.0 (0.0) | 0.0 (0.2) | 0.0 (0.1) | 0.0 90.1) | 0.0 (0.1) |
| Coastal California | 0.0 (0.0) | 0.0 (0.0) | 0.0 (0.0) | 0.0 (0.2) | 0.0 (0.1) | 0.0 (0.0) | 0.0 (0.1) | 0.0 (0.1) | 0.0 (0.0) |

Supplementary Table S4 continued

| Conservation Unit | Strait of Georgia-north sport | | | | | | | | | |
| --- | --- | --- | --- | --- | --- | --- | --- | --- | --- | --- |
|  | February | | March | | April | | | May | | |
|  | Legal | Sublegal | Legal | Sublegal | Legal | Sublegal | Unknown | Legal | Sublegal | Unknown |
| Sample size | 7 | 5 | 28 | 14 | 5 | 5 | 11 | 39 | 1 | 31 |
| N PBT | 1 | 1 | 4 | 2 | 2 | 0 | 4 | 3 | 0 | 7 |
| Southeast Alaska | 0.0 (0.9) | 0.0 (1.1) | 0.0 (0.3) | 0.0 (0.2) | 0.0 (0.8) | 0.0 (6.0) | 0.0 (0.7) | 0.0 (0.4) | 0.0 (1.2) | 0.0 (0.8) |
| Alsek | 0.0 (0.9) | 0.0 (2.7) | 0.0 (0.0) | 0.0 (0.1) | 0.0 (2.8) | 0.0 (0.5) | 0.0 (0.3) | 0.0 (0.2) | 0.0 (4.5) | 0.0 (0.0) |
| Unuk | 0.0 (0.0) | 0.0 (0.0) | 0.0 (0.0) | 0.0 (0.0) | 0.0 (0.0) | 0.0 (0.0) | 0.0 (0.0) | 0.0 (0.0) | 0.0 (0.0) | 0.0 (0.0) |
| Taku_early timing | 0.0 (0.3) | 0.0 (0.0) | 0.0 (0.0) | 0.0 (0.4) | 0.0 (5.6) | 0.0 (0.0) | 0.0 (0.6) | 0.0 (0.2) | 0.0 (4.7) | 0.0 (0.2) |
| Taku_mid timing | 0.0 (1.1) | 0.0 (1.0) | 0.0 (0.0) | 0.0 (0.8) | 0.0 (0.1) | 0.0 (0.2) | 0.0 (0.4) | 0.0 (0.0) | 0.0 (0.4) | 0.0 (0.3) |
| Taku_late timing | 0.0 (2.1) | 0.0 (0.1) | 0.0 (0.0) | 0.0 (0.1) | 0.0 (1.4) | 0.0 (0.1) | 0.0 (0.1) | 0.0 (0.0) | 0.0 (1.3) | 0.0 (0.0) |
| Stikine_early timing | 0.0 (0.5) | 0.0 (0.2) | 0.0 (0.1) | 0.0 (0.0) | 0.0 (2.4) | 0.0 (0.1) | 0.0 (0.2) | 0.0 (0.1) | 0.0 (0.1) | 0.0 (0.1) |
| Stikine_late timing | 0.0 (0.0) | 0.0 (0.4) | 0.0 (0.1) | 0.0 (0.1) | 0.0 (2.0) | 0.0 (0.0) | 0.0 (1.0) | 0.0 (0.1) | 0.0 (1.8) | 0.0 (0.1) |
| Haida Gwaii-North | 0.0 (0.0) | 0.0 (0.0) | 0.0 (0.0) | 0.0 (0.0) | 0.0 (0.0) | 0.0 (0.1) | 0.0 (0.0) | 0.0 (0.0) | 0.0 (2.2) | 0.0 (0.0) |
| Upper Nass | 0.0 (1.0) | 0.0 (0.2) | 0.0 (0.4) | 0.0 (0.7) | 0.0 (2.3) | 0.0 (2.3) | 0.0 (0.3) | 0.0 (0.1) | 0.0 (9.1) | 0.0 (0.3) |
| Portland Sound-Observatory Inlet-Lower Nass | 0.0 (0.5) | 0.0 (0.3) | 0.0 (0.4) | 0.0 (0.2) | 0.0 (0.0) | 0.0 (0.1) | 0.0 (0.3) | 0.0 (0.0) | 0.0 (6.0) | 0.0 (1.1) |
| Ecstall | 0.0 (0.1) | 0.0 (0.0) | 0.0 (0.0) | 0.0 (0.0) | 0.0 (0.0) | 0.0 (0.0) | 0.0 (0.0) | 0.0 (0.0) | 0.0 (0.0) | 0.0 (0.0) |
| Skeena Estuary | 0.0 (0.0) | 0.0 (0.0) | 0.0 (0.0) | 0.0 (0.1) | 0.0 (0.0) | 0.0 (0.2) | 0.0 (0.0) | 0.0 (0.0) | 0.0 (0.0) | 0.0 (0.0) |
| Lower Skeena | 0.0 (0.1) | 0.0 (4.5) | 0.0 (0.5) | 0.0 (0.1) | 0.0 (0.5) | 0.0 (8.2) | 0.0 (0.7) | 0.0 (0.9) | 0.0 (2.1) | 0.0 (0.2) |
| Kalum_early timing | 0.0 (0.0) | 0.0 (4.0) | 0.0 (0.0) | 0.0 (0.0) | 0.0 (2.2) | 0.0 (0.0) | 0.0 (0.0) | 0.0 (0.0) | 0.0 (3.0) | 0.0 (0.0) |
| Kalum_late timing | 0.0 (0.0) | 0.0 (0.0) | 0.0 (0.0) | 0.0 (0.1) | 0.0 (0.1) | 0.0 (0.2) | 0.0 (0.1) | 0.0 (0.0) | 0.0 (4.0) | 0.0 (0.0) |
| Zymoetz | 0.0 (0.0) | 0.0 (0.0) | 0.0 (0.0) | 0.0 (0.0) | 0.0 (0.3) | 0.0 (0.2) | 0.0 (0.0) | 0.0 (0.0) | 0.0 (0.7) | 0.0 (0.0) |
| Sicintine | 0.0 (0.0) | 0.0 (0.0) | 0.0 (0.1) | 0.0 (0.0) | 0.0 (0.0) | 0.0 (0.0) | 0.0 (0.0) | 0.0 (0.0) | 0.0 (0.0) | 0.0 (0.0) |
| Middle Skeena-mainstem tributaries | 0.0 (0.4) | 0.0 (2.3) | 0.0 (0.7) | 0.0 (0.7) | 0.0 (0.8) | 0.0 (1.1) | 0.0 (0.3) | 0.0 (0.0) | 0.0 (4.4) | 0.0 (1.1) |
| Middle Skeena-large lakes | 0.0 (0.6) | 0.0 (2.8) | 0.0 (0.0) | 0.0 (0.1) | 0.0 (0.5) | 0.0 (0.8) | 0.0 (0.1) | 0.0 (0.0) | 0.0 (5.4) | 0.0 (0.0) |
| Upper Skeena | 0.0 (0.2) | 0.0 (2.6) | 0.0 (0.5) | 0.0 (0.1) | 0.0 (2.4) | 0.0 (2.2) | 0.0 (0.7) | 0.0 (0.0) | 0.0 (0.5) | 0.0 (0.0) |
| Upper Bulkley River | 0.0 (0.0) | 0.0 (0.0) | 0.0 (0.0) | 0.0 (0.0) | 0.0 (0.0) | 0.0 (1.3) | 0.0 (0.0) | 0.0 (0.0) | 0.0 (0.1) | 0.0 (0.0) |
| North and Central Coast-late timing | 0.0 (0.0) | 0.0 (0.1) | 0.0 (0.0) | 0.0 (0.0) | 0.0 (0.0) | 0.0 (0.2) | 0.0 (0.0) | 0.0 (0.0) | 0.0 (5.8) | 0.0 (0.0) |
| North and Central Coast-early timing | 0.0 (0.1) | 0.0 (1.5) | 0.0 (0.0) | 0.0 (0.2) | 0.0 (0.4) | 0.0 (0.0) | 0.0 (0.2) | 0.0 (0.3) | 0.0 (4.8) | 0.0 (0.0) |
| Rivers Inlet | 0.0 (4.3) | 0.0 (0.3) | 0.0 (0.2) | 0.0 (0.6) | 0.0 (0.0) | 0.0 (0.0) | 0.0 (0.0) | 0.0 (0.3) | 0.0 (0.0) | 0.0 (0.0) |
| Wannock | 0.0 (0.1) | 0.0 (0.1) | 0.0 (0.0) | 0.0 (0.0) | 0.0 (0.0) | 0.0 (0.7) | 0.0 (0.1) | 0.0 (0.0) | 0.0 (0.0) | 0.0 (0.0) |
| Bella Coola-Bentinck | 0.0 (0.2) | 0.0 (0.0) | 0.0 (0.1) | 0.0 (0.6) | 0.0 (1.7) | 0.0 (3.9) | 0.0 (0.1) | 0.0 (0.3) | 0.0 (3.4) | 0.0 (0.1) |
| Dean River | 0.0 (0.0) | 0.0 (0.0) | 0.0 (0.0) | 0.0 (0.8) | 0.0 (0.0) | 0.0 (0.2) | 0.0 (0.5) | 0.0 (0.0) | 0.0 (0.8) | 0.0 (0.1) |
| Docee | 0.0 (0.0) | 0.0 (0.0) | 0.0 (0.0) | 0.0 (0.0) | 0.0 (0.0) | 0.0 (0.0) | 0.0 (0.0) | 0.0 (0.0) | 0.0 (0.1) | 0.0 (0.0) |
| Klinaklini_SU_1.3 | 0.0 (0.5) | 0.0 (0.0) | 0.0 (0.0) | 0.0 (0.1) | 0.0 (0.5) | 0.0 (0.0) | 0.0 (0.0) | 0.0 (0.1) | 0.0 (1.8) | 0.0 (0.7) |
| Southern Mainland-Southern Fjords_FA_0.x | 0.0 (0.0) | 0.0 (0.0) | 0.0 (0.4) | 0.0 (1.8) | 0.0 (0.0) | 0.0 (0.0) | 0.0 (0.4) | 0.0 (0.0) | 0.0 (0.0) | 0.0 (0.0) |
| Southern Mainland-Georgia Strait_FA_0.x | 0.0 (1.1) | 0.0 (0.6) | 3.6 (4.1) | 0.0 (0.5) | 1.4 (9.1) | 0.0 (0.9) | 9.1 (6.8) | 38.5 (7.7) | 0.0 (4.7) | 3.2 (3.2) |
| Upper Fraser River_SP_1.3 | 0.0 (1.0) | 0.0 (0.9) | 0.0 (0.5) | 0.0 (0.5) | 0.0 (3.3) | 0.0 (5.2) | 0.0 (0.8) | 0.0 (1.0) | 0.0 (7.4) | 0.0 (0.8) |
| Middle Fraser River_SU_1.3 | 0.0 (0.3) | 0.0 (1.3) | 0.0 (1.0) | 0.0 (0.0) | 0.0 (0.0) | 0.0 (3.6) | 0.0 (2.1) | 0.0 (0.1) | 0.0 (3.1) | 0.0 (0.2) |
| Middle Fraser River_SP_1.3 | 0.0 (0.8) | 0.0 (1.1) | 0.0 (0.7) | 0.0 (3.0) | 0.0 (0.7) | 0.0 (3.8) | 0.0 (3.0) | 0.0 (0.7) | 0.0 (16.0) | 0.0 (0.6) |
| Middle Fraser River-Portage_FA_1.3 | 0.0 (0.0) | 0.0 (0.0) | 0.0 (0.2) | 0.0 (0.0) | 0.0 (0.0) | 0.0 (0.0) | 0.0 (0.8) | 0.0 (0.0) | 0.0 (0.4) | 0.0 (0.1) |
| Middle Fraser-Fraser Canyon_SP_1.3 | 0.0 (0.0) | 0.0 (0.0) | 0.0 (0.0) | 0.0 (0.9) | 0.0 (0.1) | 0.0 (0.0) | 0.0 (0.0) | 0.0 (0.0) | 0.0 (0.0) | 0.0 (0.0) |
| North Thompson_SP_1.3 | 0.0 (0.0) | 0.0 (0.5) | 0.0 (0.6) | 0.0 (0.0) | 0.0 (0.2) | 0.0 (0.1) | 0.0 (0.0) | 0.0 (0.0) | 0.0 (0.0) | 0.0 (0.1) |
| North Thompson_SU_1.3 | 0.0 (0.7) | 0.0 (0.0) | 0.0 (0.1) | 0.0 (0.1) | 0.0 (0.4) | 0.0 (0.7) | 0.0 (0.0) | 0.0 (0.6) | 0.0 (0.3) | 0.0 (0.6) |
| Shuswap River_SU_0.3 | 0.0 (0.8) | 0.0 (0.0) | 0.0 (0.0) | 0.0 (0.0) | 0.0 (1.9) | 0.0 (0.4) | 0.0 (0.4) | 0.0 (0.1) | 0.0 (0.0) | 0.0 (0.0) |
| South Thompson-Bessette Creek_SU_1.2 | 0.0 (0.0) | 0.0 (0.0) | 0.0 (0.0) | 0.0 (0.0) | 0.0 (0.0) | 0.0 (0.0) | 0.0 (0.1) | 0.0 (0.4) | 0.0 (1.7) | 0.0 (0.0) |
| South Thompson_SU_0.3 | 0.0 (1.1) | 0.0 (0.0) | 0.0 (0.0) | 0.0 (2.0) | 0.0 (0.1) | 0.0 (0.7) | 0.0 (0.3) | 0.0 (0.0) | 0.0 (1.1) | 0.0 (0.0) |
| South Thompson_SU_1.3 | 0.0 (0.0) | 0.0 (1.9) | 0.0 (0.0) | 0.0 (0.1) | 0.0 (0.0) | 0.0 (2.2) | 0.0 (1.1) | 0.0 (0.5) | 0.0 (0.0) | 0.0 (0.0) |
| Lower Thompson_SP_1.2 | 0.0 (1.0) | 20.0 (16.8) | 0.0 (1.1) | 0.0 (0.1) | 0.0 (1.6) | 0.0 (0.3) | 0.0 (0.3) | 0.0 (0.2) | 0.0 (10.1) | 0.0 (0.2) |
| Lower Fraser River_SP_1.3 | 0.0 (0.0) | 0.0 (0.0) | 3.6 (2.4) | 0.0 (4.0) | 0.0 (0.0) | 0.0 (0.1) | 0.0 (1.1) | 0.0 (0.0) | 0.0 (0.1) | 0.0 (0.1) |
| Lower Fraser River_SU_1.3 | 0.0 (0.6) | 0.0 (0.1) | 0.0 (0.0) | 0.0 (0.4) | 0.0 (0.0) | 0.0 (0.6) | 0.0 (0.6) | 0.0 (0.4) | 0.0 (0.6) | 0.0 (0.0) |
| Lower Fraser River-Upper Pitt_SU_1.3 | 0.0 (0.4) | 0.0 (0.0) | 0.0 (0.5) | 0.0 (0.0) | 0.0 (0.0) | 0.0 (0.1) | 0.0 (0.0) | 0.0 (0.0) | 0.0 (0.0) | 0.0 (0.0) |
| Maria Slough_SU_0.3 | 0.0 (0.0) | 0.0 (0.0) | 0.0 (0.0) | 0.0 (0.0) | 0.0 (0.4) | 0.0 (0.1) | 0.0 (0.1) | 0.0 (0.2) | 0.0 (1.5) | 0.0 (0.5) |
| Lower Fraser River_FA_0.3 | 0.0 (0.9) | 0.0 (2.1) | 3.6 (2.7) | 7.1 (8.0) | 0.0 (0.7) | 40.0 (18.4) | 0.0 (0.1) | 12.8 (4.0) | 100.0 (28.5) | 9.7 (5.0) |
| East Vancouver Island-North_FA_0.x | 0.0 (0.8) | 0.0 (0.1) | 0.0 (0.0) | 0.0 (0.5) | 0.0 (0.0) | 0.0 (0.5) | 0.0 (0.9) | 7.7 (5.1) | 0.0 (6.8) | 0.0 (0.1) |
| East Vancouver Island-Qualicum and Puntledge_FA_0.x | 0.0 (0.0) | 4.7 (8.6) | 32.8 (9.6) | 40.2 (11.1) | 50.5 (16.9) | 0.0 (1.3) | 54.0 (15.4) | 8.6 (5.1) | 0.0 (0.4) | 30.0 (7.6) |
| East Vancouver Island-Nanaimo and Chemainus_FA_0.x | 0.0 (0.7) | 1.9 (7.7) | 0.0 (0.0) | 0.0 (0.0) | 0.0 (0.0) | 19.6 (15.3) | 0.0 (0.0) | 0.0 (0.2) | 0.0 (0.0) | 0.1 (1.6) |
| East Vancouver Island-Nanaimo_SP_1.x | 0.0 (0.0) | 0.0 (0.5) | 0.0 (0.0) | 0.0 (0.0) | 0.0 (0.7) | 0.0 (0.0) | 0.0 (0.0) | 0.0 (0.0) | 0.0 (0.4) | 0.0 (0.0) |
| East Vancouver Island-Georgia Strait_SU_0.3 | 14.3 (8.2) | 0.0 (0.1) | 14.3 (7.5) | 37.9 (10.8) | 0.0 (0.0) | 20.4 (17.5) | 18.2 (9.3) | 0.0 (0.0) | 0.0 (4.4) | 6.5 (4.8) |
| East Vancouver Island-Cowichan and Koksilah_FA_0.x | 28.6 (15.4) | 53.4 (18.9) | 17.2 (6.8) | 0.5 (0.2) | 48.0 (18.3) | 0.0 (0.0) | 0.0 (0.7) | 19.6 (6.6) | 0.0 (0.8) | 34.4 (7.8) |
| West Vancouver Island-Nootka and Kyuquot_FA_0.x | 0.0 (2.3) | 0.0 (0.7) | 0.0 (1.0) | 0.0 (0.5) | 0.0 (3.0) | 0.0 (1.8) | 0.0 (0.9) | 0.0 (0.5) | 0.0 (9.3) | 0.0 (0.1) |
| West Vancouver Island-North_FA_0.x | 0.0 (0.1) | 0.0 (0.6) | 0.0 (0.0) | 0.0 (0.0) | 0.0 (0.2) | 0.0 (0.2) | 0.0 (0.1) | 0.0 (0.0) | 0.0 (0.6) | 0.0 (0.0) |
| West Vancouver Island-South_FA_0.x | 0.0 (1.1) | 0.0 (4.2) | 0.0 (0.8) | 0.0 (1.1) | 0.0 (0.5) | 0.0 (1.7) | 0.0 (1.8) | 0.0 (0.3) | 0.0 (1.4) | 0.0 (0.0) |
| Okanagan_1.x | 0.0 (2.3) | 0.0 (0.0) | 0.0 (0.0) | 0.0 (0.0) | 0.0 (0.0) | 0.0 (0.0) | 0.0 (0.1) | 0.0 (0.0) | 0.0 (0.4) | 0.0 (0.0) |
| Juan de Fuca | 0.0 (0.0) | 0.0 (0.0) | 0.0 (0.0) | 0.0 (0.5) | 0.0 (0.1) | 0.0 (0.4) | 0.0 (0.1) | 2.6 (2.4) | 0.0 (2.6) | 0.0 (0.0) |
| Coastal Washington | 0.0 (0.6) | 0.0 (4.4) | 0.0 (0.0) | 0.0 (1.5) | 0.0 (2.0) | 0.0 (1.7) | 0.0 (0.5) | 0.0 (0.0) | 0.0 (9.5) | 0.0 (0.2) |
| North Puget Sound | 14.2 (12.7} | 4.9 (12.0) | 11.7 (6.1) | 1.5 (3.4) | 0.0 (1.8) | 0.6 (5.1) | 9.1 (8.5) | 8.0 (5.5) | 0.0 (0.0) | 0.0 (0.0) |
| South Puget Sound | 42.9 (16.3) | 15.1 (17.4) | 10.3 (5.0) | 12.8 (9.1) | 0.0 (0.0) | 19.5 (12.0) | 9.1 (8.2) | 2.3 (4.7) | 0.0 (2.3) | 16.1 (5.3) |
| Lower Columbia River | 0.0 (0.1) | 0.0 (1.3) | 0.0 (0.3) | 0.0 (0.1) | 0.0 (0.7) | 0.0 (0.0) | 0.0 (1.2) | 0.0 (0.5) | 0.0 (2.9) | 0.0 (0.3) |
| Mid Columbia River_SP | 0.0 (0.0) | 0.0 (0.0) | 2.7 (2.9) | 0.0 (0.9) | 0.0 (0.1) | 0.0 (0.4) | 0.0 (0.0) | 0.0 (0.0) | 0.0 (0.1) | 0.0 (0.2) |
| Upper Columbia River_SP | 0.0 (0.5) | 0.0 (0.0) | 0.0 (0.1) | 0.0 (0.3) | 0.0 (0.3) | 0.0 (0.2) | 0.0 (2.6) | 0.0 (0.0) | 0.0 (9.3) | 0.0 (0.0) |
| Upper Columbia River_SU_FA | 0.0 (0.2) | 0.0 (1.6) | 0.0 (0.2) | 0.0 (0.4) | 0.0 (0.1) | 0.0 (1.0) | 0.0 (0.2) | 0.0 (0.4) | 0.0 (1.7) | 0.0 (0.6) |
| Snake River_FA | 0.0 (0.0) | 0.0 (0.0) | 0.0 (0.3) | 0.0 (0.0) | 0.0 (0.0) | 0.0 (0.5) | 0.0 (0.0) | 0.0 (0.0) | 0.0 (0.0) | 0.0 (0.0) |
| Snake River_SP_SU | 0.0 (1.4) | 0.0 (1.7) | 0.0 (0.2) | 0.0 (0.3) | 0.0 (0.5) | 0.0 (1.9) | 0.0 (0.4) | 0.0 (0.8) | 0.0 (3.2) | 0.0 (0.2) |
| North & Central Oregon | 0.0 (0.2) | 0.0 (4.1) | 0.0 (0.2) | 0.0 (0.1) | 0.0 (1.8) | 0.0 (0.7) | 0.0 (0.6) | 0.0 (0.1) | 0.0 (0.1) | 0.0 (0.1) |
| Upper Willamette River | 0.0 (1.5) | 0.0 (1.1) | 0.0 (0.0) | 0.0 (0.5) | 0.0 (1.3) | 0.0 (0.0) | 0.0 (0.0) | 0.0 (0.0) | 0.0 (0.0) | 0.0 (0.1) |
| South Oregon coastal | 0.0 (0.5) | 0.0 (1.3) | 0.0 (0.1) | 0.0 (0.3) | 0.0 (0.3) | 0.0 (1.2) | 0.0 (0.7) | 0.0 (0.0) | 0.0 (9.0) | 0.0 (0.1) |
| California Klamath Trinity | 0.0 (0.3) | 0.0 (0.0) | 0.0 (0.0) | 0.0 (0.0) | 0.0 (0.5) | 0.0 (4.5) | 0.0 (0.1) | 0.0 (0.0) | 0.0 (7.2) | 0.0 (0.1) |
| California Central Valley_Fall | 0.0 (0.0) | 0.0 (6.3) | 0.0 (0.1) | 0.0 (0.4) | 0.0 (4.5) | 0.0 (0.5) | 0.0 (0.5) | 0.0 (0.3) | 0.0 (3.4) | 0.0 (0.3) |
| California Central Valley_Spring | 0.0 (0.2) | 0.0 (0.1) | 0.0 (0.0) | 0.0 (0.0) | 0.0 (0.0) | 0.0 (1.1) | 0.0 (0.5) | 0.0 (0.1) | 0.0 (0.1) | 0.0 (0.1) |
| Coastal California | 0.0 (0.0) | 0.0 (1.3) | 0.0 (0.0) | 0.0 (0.0) | 0.0 (0.3) | 0.0 (0.4) | 0.0 (0.0) | 0.0 (0.0) | 0.0 (0.0) | 0.0 (0.0) |

Supplementary Table S4 continued

| Conservation Unit | Strait of Georgia-north sport | | | | | | | | | |
| --- | --- | --- | --- | --- | --- | --- | --- | --- | --- | --- |
|  | June | | | July | | | August | | September | |
|  | Legal | Sublegal | Unknown | Legal | Sublegal | Unknown | Legal | Sublegal | Legal | Sublegal |
| Sample size | 198 | 5 | 58 | 203 | 23 | 12 | 149 | 16 | 41 | 5 |
| N PBT | 55 | 2 | 14 | 40 | 6 | 3 | 33 | 4 | 6 | 1 |
| Southeast Alaska | 0.0 (0.1) | 0.0 (2.0) | 0.0 (0.2) | 0.0 (0.1) | 0.0 (0.0) | 0.0 (0.1) | 0.0 (0.1) | 0.0 (0.6) | 0.0 (0.1) | 0.0 (0.2) |
| Alsek | 0.0 (0.0) | 0.0 (2.4) | 0.0 (0.1) | 0.0 (0.0) | 0.0 (0.7) | 0.0 (0.3) | 0.0 (0.0) | 0.0 (0.1) | 0.0 (0.6) | 0.0 (1.4) |
| Unuk | 0.0 (0.0) | 0.0 (0.8) | 0.0 (0.0) | 0.0 (0.0) | 0.0 (0.0) | 0.0 (0.0) | 0.0 (0.0) | 0.0 (0.0) | 0.0 (0.0) | 0.0 (0.0) |
| Taku_early timing | 0.0 (0.0) | 0.0 (0.6) | 0.0 (0.0) | 0.0 (0.0) | 0.0 (0.0) | 0.0 (0.1) | 0.0 (0.0) | 0.0 (0.0) | 0.0 (0.1) | 0.0 (2.0) |
| Taku_mid timing | 0.0 (0.0) | 0.0 (0.5) | 0.0 (0.2) | 0.0 (0.0) | 0.0 (1.2) | 0.0 (0.4) | 0.0 (0.0) | 0.0 (0.0) | 0.0 (0.1) | 0.0 (1.3) |
| Taku_late timing | 0.0 (0.0) | 0.0 (0.5) | 0.0 (0.0) | 0.0 (0.0) | 0.0 (0.0) | 0.0 (1.5) | 0.0 (0.0) | 0.0 (0.1) | 0.0 (0.0) | 0.0 (0.5) |
| Stikine_early timing | 0.0 (0.1) | 0.0 (1.1) | 0.0 (0.0) | 0.0 (0.1) | 0.0 (0.0) | 0.0 (0.8) | 0.0 (0.3) | 0.0 (1.7) | 0.0 (0.2) | 0.0 (0.9) |
| Stikine_late timing | 0.0 (0.0) | 0.0 (1.0) | 0.0 (0.0) | 0.0 (0.0) | 0.0 (0.0) | 0.0 (2.2) | 0.0 (0.0) | 0.0 (0.5) | 0.0 (0.3) | 0.0 (0.5) |
| Haida Gwaii-North | 0.0 (0.0) | 0.0 (0.0) | 0.0 (0.0) | 0.0 (0.0) | 0.0 (0.0) | 0.0 (0.0) | 0.0 (0.0) | 0.0 (0.0) | 0.0 (0.1) | 0.0 (0.1) |
| Upper Nass | 0.0 (0.0) | 0.0 (0.9) | 0.0 (0.0) | 0.0 (0.0) | 0.0 (0.2) | 0.0 (0.4) | 0.0 (0.0) | 0.0 (0.1) | 0.0 (0.0) | 0.0 (2.2) |
| Portland Sound-Observatory Inlet-Lower Nass | 0.0 (0.0) | 0.0 (6.5) | 0.0 (0.0) | 0.0 (0.1) | 0.0 (0.1) | 0.0 (0.0) | 0.0 (0.0) | 0.0 (1.2) | 0.0 (0.0) | 0.0 (0.4) |
| Ecstall | 0.0 (0.0) | 0.0 (0.2) | 0.0 (0.0) | 0.0 (0.0) | 0.0 (0.0) | 0.0 (0.0) | 0.0 (0.0) | 0.0 (0.0) | 0.0 (0.0) | 0.0 (0.0) |
| Skeena Estuary | 0.0 (0.0) | 0.0 (0.0) | 0.0 (0.1) | 0.0 (0.0) | 0.0 (0.0) | 0.0 (0.0) | 0.0 (0.0) | 0.0 (0.0) | 0.0 (0.1) | 0.0 (0.1) |
| Lower Skeena | 0.0 (0.0) | 0.0 (4.3) | 0.0 (0.1) | 0.0 (0.0) | 0.0 (0.3) | 0.0 (0.8) | 0.0 (0.2) | 0.0 (0.1) | 0.0 (0.1) | 0.0 (0.9) |
| Kalum_early timing | 0.0 (0.0) | 0.0 (0.1) | 0.0 (0.0) | 0.0 (0.0) | 0.0 (0.0) | 0.0 (0.0) | 0.0 (0.0) | 0.0 (0.0) | 0.0 (0.0) | 0.0 (0.0) |
| Kalum_late timing | 0.0 (0.0) | 0.0 (1.5) | 0.0 (0.1) | 0.0 (0.0) | 0.0 (0.0) | 0.0 (0.1) | 0.0 (0.0) | 0.0 (0.0) | 0.0 (0.0) | 0.0 (0.0) |
| Zymoetz | 0.0 (0.0) | 0.0 (0.0) | 0.0 (0.0) | 0.0 (0.0) | 0.0 (0.0) | 0.0 (0.0) | 0.0 (0.0) | 0.0 (0.0) | 0.0 (0.0) | 0.0 (0.0) |
| Sicintine | 0.0 (0.0) | 0.0 (0.9) | 0.0 (0.0) | 0.0 (0.0) | 0.0 (0.0) | 0.0 (0.0) | 0.0 (0.0) | 0.0 (0.0) | 0.0 (0.0) | 0.0 (0.0) |
| Middle Skeena-mainstem tributaries | 0.0 (0.0) | 0.0 (2.5) | 0.0 (0.2) | 0.0 (0.0) | 0.0 (0.2) | 0.0 (0.4) | 0.0 (0.1) | 0.0 (0.6) | 0.0 (0.1) | 0.0 (1.3) |
| Middle Skeena-large lakes | 0.0 (0.1) | 0.0 (1.0) | 0.0 (0.1) | 0.0 (0.0) | 0.0 (0.1) | 0.0 (0.0) | 0.0 (0.1) | 0.0 (0.5) | 0.0 (0.0) | 0.0 (1.1) |
| Upper Skeena | 0.0 (0.0) | 0.0 (1.8) | 0.0 (0.1) | 0.0 (0.0) | 0.0 (0.7) | 0.0 (0.4) | 0.0 (0.1) | 0.0 (0.6) | 0.0 (0.1) | 0.0 (2.1) |
| Upper Bulkley River | 0.0 (0.0) | 0.0 (0.0) | 0.0 (0.0) | 0.0 (0.0) | 0.0 (0.0) | 0.0 (0.0) | 0.0 (0.0) | 0.0 (0.0) | 0.0 (0.0) | 0.0 (0.0) |
| North and Central Coast-late timing | 0.0 (0.1) | 0.0 (0.0) | 0.0 (0.0) | 0.0 (0.0) | 0.0 (0.0) | 0.0 (0.5) | 0.0 (0.0) | 0.0 (0.0) | 0.0 (0.1) | 0.0 (3.5) |
| North and Central Coast-early timing | 0.0 (0.0) | 0.0 (0.1) | 0.0 (0.0) | 0.0 (0.1) | 0.0 (0.3) | 0.0 (0.2) | 0.0 (0.0) | 0.0 (0.2) | 0.0 (0.3) | 0.0 (0.3) |
| Rivers Inlet | 0.0 (0.1) | 0.0 (1.2) | 0.0 (0.0) | 0.0 (0.0) | 0.0 (0.0) | 0.0 (0.2) | 0.0 (0.1) | 0.0 (0.8) | 0.0 (0.0) | 0.0 (0.2) |
| Wannock | 0.0 (0.0) | 0.0 (0.0) | 0.0 (0.0) | 0.0 (0.0) | 0.0 (0.0) | 0.0 (0.0) | 0.0 (0.0) | 0.0 (0.0) | 0.0 (0.0) | 0.0 (0.0) |
| Bella Coola-Bentinck | 0.0 (0.0) | 0.0 (0.0) | 0.0 (0.4) | 0.0 (0.0) | 0.0 (0.0) | 0.0 (0.6) | 0.0 (0.1) | 0.0 (0.3) | 0.0 (0.1) | 0.0 (0.7) |
| Dean River | 0.0 (0.0) | 0.0 (0.1) | 0.0 (0.1) | 0.0 (0.0) | 0.0 (0.4) | 0.0 (0.7) | 0.0 (0.0) | 0.0 (0.2) | 0.0 (0.0) | 0.0 (1.3) |
| Docee | 0.0 (0.0) | 0.0 (0.1) | 0.0 (0.0) | 0.0 (0.0) | 0.0 (0.0) | 0.0 (0.0) | 0.0 (0.0) | 0.0 (0.0) | 0.0 (0.0) | 0.0 (0.1) |
| Klinaklini_SU_1.3 | 0.6 (0.4) | 0.0 (1.9) | 0.0 (0.0) | 0.0 (0.0) | 0.0 (0.1) | 0.0 (0.4) | 0.6 (0.5) | 0.0 (0.0) | 0.0 (0.6) | 0.0 (2.0) |
| Southern Mainland-Southern Fjords_FA_0.x | 0.1 (0.3) | 0.0 (0.0) | 0.0 (0.0) | 0.0 (0.1) | 0.0 (0.0) | 0.0 (0.0) | 0.0 (0.0) | 0.0 (0.1) | 0.0 (0.0) | 0.0 (0.0) |
| Southern Mainland-Georgia Strait_FA_0.x | 3.8 (1.7) | 0.1 (2.1) | 1.7 (1.9) | 1.5 (0.8) | 4.2 (2.7) | 0.0 (0.4) | 0.0 (0.1) | 0.0 (1.0) | 0.0 (1.0) | 0.0 (5.4) |
| Upper Fraser River_SP_1.3 | 0.0 (0.1) | 0.0 (0.6) | 0.0 (0.2) | 0.0 (0.0) | 0.0 (2.0) | 0.0 (3.8) | 0.0 (0.1) | 0.0 (0.3) | 0.0 (0.4) | 0.0 (1.1) |
| Middle Fraser River_SU_1.3 | 0.5 (0.5) | 0.0 (3.5) | 0.0 (0.0) | 0.0 (0.1) | 4.0 (3.1) | 0.0 (0.6) | 0.7 (0.9) | 0.0 (1.2) | 1.6 (1.7) | 0.0 (1.6) |
| Middle Fraser River_SP_1.3 | 0.0 (0.0) | 0.0 (1.1) | 0.0 (0.4) | 0.0 (0.0) | 0.2 (0.3) | 0.0 (0.4) | 0.0 (0.2) | 0.0 (0.7) | 0.8 (1.9) | 0.0 (2.1) |
| Middle Fraser River-Portage_FA_1.3 | 0.0 (0.0) | 0.0 (0.0) | 0.0 (0.0) | 0.0 (0.0) | 0.0 (0.4) | 0.0 (0.0) | 0.0 (0.0) | 0.0 (0.1) | 0.0 (0.0) | 0.0 (0.0) |
| Middle Fraser-Fraser Canyon_SP_1.3 | 0.0 (0.0) | 0.0 (0.0) | 0.0 (0.2) | 0.0 (0.0) | 0.0 (0.5) | 0.0 (0.0) | 0.0 (0.0) | 0.0 (0.0) | 0.0 (0.0) | 0.0 (0.7) |
| North Thompson_SP_1.3 | 0.0 (0.1) | 0.0 (0.4) | 0.0 (0.0) | 0.0 (0.0) | 0.0 (0.0) | 0.0 (0.0) | 0.0 (0.0) | 0.0 (0.1) | 0.0 (0.0) | 0.0 (0.4) |
| North Thompson_SU_1.3 | 0.0 (0.0) | 0.0 (2.0) | 0.0 (0.3) | 0.5 (0.5) | 4.2 (4.0) | 0.0 (1.4) | 0.0 (0.0) | 0.0 (0.1) | 0.0 (0.1) | 0.0 (0.6) |
| Shuswap River_SU_0.3 | 0.0 (0.0) | 0.0 (0.0) | 0.0 (0.2) | 3.5 (1.3) | 0.0 (0.0) | 0.0 (0.4) | 2.7 (1.1) | 6.3 (6.2) | 0.0 (0.0) | 0.0 (0.0) |
| South Thompson-Bessette Creek_SU_1.2 | 0.0 (0.0) | 0.0 (0.0) | 0.0 (0.0) | 0.0 (0.0) | 0.0 (0.1) | 0.0 (0.4) | 0.0 (0.0) | 0.0 (0.0) | 0.0 (0.0) | 0.0 (1.6) |
| South Thompson_SU_0.3 | 0.0 (0.0) | 0.0 (0.1) | 1.7 (1.8) | 0.0 (0.1) | 0.0 (0.1) | 0.0 (0.0) | 9.4 (2.0) | 25.0 (7.7) | 19.5 (5.8) | 0.0 (2.6) |
| South Thompson_SU_1.3 | 0.0 (0.0) | 0.0 (0.4) | 0.0 (0.0) | 0.5 (0.4) | 0.0 (0.0) | 0.0 (0.2) | 0.0 (0.0) | 0.0 (0.1) | 0.0 (0.0) | 0.0 (5.0) |
| Lower Thompson_SP_1.2 | 0.0 (0.2) | 0.0 (2.2) | 0.0 (0.0) | 0.0 (0.0) | 0.0 (0.9) | 0.0 (0.8) | 0.0 (0.0) | 0.0 (1.1) | 0.0 (0.0) | 0.0 (1.4) |
| Lower Fraser River_SP_1.3 | 0.0 (0.0) | 0.0 (0.4) | 0.0 (0.0) | 0.0 (0.0) | 0.0 (0.2) | 0.0 (0.0) | 0.0 (0.0) | 0.0 (0.0) | 0.0 (0.0) | 0.0 (0.0) |
| Lower Fraser River_SU_1.3 | 0.0 (0.0) | 0.0 (1.0) | 0.0 (0.2) | 0.5 (0.6) | 0.0 (0.0) | 0.0 (0.4) | 0.0 (0.0) | 0.0 (0.3) | 0.0 (0.0) | 0.0 (0.1) |
| Lower Fraser River-Upper Pitt_SU_1.3 | 0.5 (0.5) | 0.0 (0.2) | 0.0 (0.0) | 0.5 (0.5) | 0.0 (1.4) | 0.0 (0.9) | 0.0 (0.0) | 0.0 (0.0) | 0.0 (0.4) | 0.0 (0.0) |
| Maria Slough_SU_0.3 | 0.0 (0.0) | 0.0 (0.0) | 0.0 (0.0) | 0.0 (0.0) | 0.0 (0.1) | 0.0 (0.0) | 0.0 (0.0) | 0.0 (0.0) | 0.0 (0.0) | 0.0 (0.0) |
| Lower Fraser River_FA_0.3 | 47.5 (3.2) | 20.0 (12.3) | 27.6 (5.7) | 44.3 (4.3) | 16.7 (6.1) | 25.0 (9.3) | 34.2 (4.3) | 6.3 (6.1) | 34.1 (8.7) | 0.0 (0.1) |
| East Vancouver Island-North_FA_0.x | 1.0 (0.9) | 0.0 (0.8) | 1.7 (1.6) | 0.0 (0.0) | 0.0 (0.5) | 0.0 (0.3) | 3.4 (1.2) | 0.0 (0.5) | 4.9 (4.0) | 0.0 (1.4) |
| East Vancouver Island-Qualicum and Puntledge_FA_0.x | 14.9 (2.5) | 19.6 (14.5) | 21.3 (6.2) | 9.5 (2.1) | 45.8 (9.5) | 8.4 (6.0) | 17.8 (3.4) | 33.0 (13.4) | 6.2 (4.9) | 59.7 (18.8) |
| East Vancouver Island-Nanaimo and Chemainus_FA_0.x | 0.6 (0.8) | 0.0 (0.1) | 0.0 (0.0) | 0.0 (0.0) | 0.0 (0.0) | 0.0 (0.0) | 1.0 (1.0) | 0.0 (0.0) | 0.0 (0.1) | 0.0 (0.0) |
| East Vancouver Island-Nanaimo_SP_1.x | 0.0 (0.0) | 0.0 (0.0) | 0.0 (0.4) | 0.0 (0.0) | 0.0 (0.1) | 0.0 (0.0) | 0.0 (0.0) | 0.0 (0.0) | 0.0 (0.1) | 0.0 (0.5) |
| East Vancouver Island-Georgia Strait_SU_0.3 | 1.4 (0.9) | 20.0 (11.6) | 3.4 (2.1) | 2.5 (1.2) | 8.3 (5.3) | 0.0 (0.5) | 2.5 (1.4) | 0.0 (0.0) | 0.0 (0.1) | 0.0 (0.1) |
| East Vancouver Island-Cowichan and Koksilah_FA_0.x | 16.5 (2.4) | 40.4 (15.7) | 21.9 (6.4) | 14.3 (2.4) | 0.0 (0.0) | 33.2 (10.2) | 15.0 (3.0) | 23.2 (12.0) | 18.2 (6.4) | 0.3 (0.0) |
| West Vancouver Island-Nootka and Kyuquot_FA_0.x | 0.0 (0.0) | 0.0 (2.2) | 0.0 (0.0) | 0.0 (0.0) | 0.0 (0.3) | 0.0 (0.6) | 0.0 (0.1) | 0.0 (0.3) | 0.0 (0.4) | 0.0 (0.7) |
| West Vancouver Island-North_FA_0.x | 0.0 (0.0) | 0.0 (1.9) | 0.0 (0.2) | 0.0 (0.0) | 0.0 (0.1) | 0.0 (0.1) | 0.0 (0.0) | 0.0 (1.2) | 0.0 (0.0) | 0.0 (1.0) |
| West Vancouver Island-South_FA_0.x | 0.0 (0.1) | 0.0 (5.9) | 0.0 (0.1) | 0.7 (0.5) | 0.0 (0.6) | 0.0 (2.0) | 1.3 (0.9) | 0.0 (1.2) | 0.0 (0.5) | 0.0 (1.5) |
| Okanagan_1.x | 0.0 (0.0) | 0.0 (0.2) | 0.0 (0.0) | 0.0 (0.0) | 0.0 (0.2) | 0.0 (0.1) | 0.0 (0.0) | 0.0 (0.0) | 0.0 (0.2) | 0.0 (0.0) |
| Juan de Fuca | 0.5 (0.6) | 0.0 (0.1) | 0.0 (0.0) | 0.0 (0.0) | 0.0 (0.1) | 0.0 (0.0) | 0.0 (0.0) | 0.0 (0.0) | 0.0 (0.0) | 0.0 (0.8) |
| Coastal Washington | 0.0 (0.0) | 0.0 (1.9) | 0.0 (0.0) | 0.0 (0.0) | 0.0 (0.0) | 0.0 (0.7) | 0.0 (0.0) | 0.0 (1.2) | 0.0 (0.3) | 0.0 (0.2) |
| North Puget Sound | 1.7 (1.0) | 0.0 (0.2) | 2.7 (1.9) | 7.2 (1.9) | 5.5 (6.2) | 0.0 (0.1) | 2.4 (1.5) | 3.4 (6.5) | 3.9 (4.0) | 40.0 (17.8) |
| South Puget Sound | 9.9 (2.0) | 0.0 (0.0) | 17.9 (4.4) | 14.6 (3.0) | 11.2 (6.5) | 33.3 (12.9) | 7.7 (2.5) | 2.8 (5.7) | 10.8 (4.4) | 0.6 (5.0) |
| Lower Columbia River | 0.5 (0.5) | 0.0 (0.1) | 0.0 (0.0) | 0.0 (0.0) | 0.0 (0.0) | 0.0 (0.0) | 0.7 (0.6) | 0.0 (0.3) | 0.0 (0.1) | 0.0 (0.0) |
| Mid Columbia River_SP | 0.0 (0.0) | 0.0 (0.4) | 0.0 (0.2) | 0.0 (0.0) | 0.0 (0.0) | 0.0 (0.1) | 0.0 (0.2) | 0.0 (0.0) | 0.0 (0.3) | 0.0 (0.0) |
| Upper Columbia River_SP | 0.0 (0.0) | 0.0 (2.0) | 0.0 (0.0) | 0.0 (0.1) | 0.0 (0.0) | 0.0 (0.2) | 0.0 (0.1) | 0.0 (0.5) | 0.0 (0.0) | 0.0 (0.3) |
| Upper Columbia River_SU_FA | 0.0 (0.0) | 0.0 (1.6) | 0.0 (0.2) | 0.0 (0.1) | 0.0 (0.0) | 0.0 (0.4) | 0.2 (0.5) | 0.0 (0.0) | 0.0 (0.0) | 0.0 (2.4) |
| Snake River_FA | 0.0 (0.0) | 0.0 (0.0) | 0.0 (0.0) | 0.0 (0.0) | 0.0 (0.0) | 0.0 (0.4) | 0.5 (0.6) | 0.0 (0.0) | 0.0 (0.0) | 0.0 (0.1) |
| Snake River_SP_SU | 0.0 (0.3) | 0.0 (2.2) | 0.0 (0.1) | 0.0 (0.1) | 0.0 (0.5) | 0.0 (0.5) | 0.0 (0.0) | 0.0 (0.9) | 0.0 (0.3) | 0.0 (4.2) |
| North & Central Oregon | 0.0 (0.0) | 0.0 (1.3) | 0.0 (0.3) | 0.0 (0.0) | 0.0 (0.1) | 0.0 (0.1) | 0.0 (0.0) | 0.0 (0.2) | 0.0 (0.0) | 0.0 (0.1) |
| Upper Willamette River | 0.0 (0.0) | 0.0 (1.3) | 0.0 (0.0) | 0.0 (0.0) | 0.0 (0.1) | 0.0 (0.7) | 0.0 (0.0) | 0.0 (0.1) | 0.0 (0.2) | 0.0 (0.1) |
| South Oregon coastal | 0.0 (0.2) | 0.0 (0.8) | 0.0 (0.1) | 0.0 (0.1) | 0.0 (0.5) | 0.0 (0.3) | 0.0 (0.1) | 0.0 (0.7) | 0.0 (0.0) | 0.0 (0.1) |
| California Klamath Trinity | 0.0 (0.0) | 0.0 (0.8) | 0.0 (0.1) | 0.0 (0.0) | 0.0 (0.0) | 0.0 (0.8) | 0.0 (0.0) | 0.0 (0.1) | 0.0 (0.3) | 0.0 (0.4) |
| California Central Valley_Fall | 0.0 (0.0) | 0.0 (1.5) | 0.0 (0.8) | 0.0 (0.0) | 0.0 (0.7) | 0.0 (1.4) | 0.0 (0.2) | 0.0 (0.3) | 0.0 (0.1) | 0.0 (0.6) |
| California Central Valley_Spring | 0.0 (0.0) | 0.0 (3.7) | 0.0 (0.0) | 0.0 (0.0) | 0.0 (0.0) | 0.0 (0.7) | 0.0 (0.0) | 0.0 (0.0) | 0.0 (0.0) | 0.0 (2.8) |
| Coastal California | 0.0 (0.0) | 0.0 (0.0) | 0.0 (0.1) | 0.0 (0.0) | 0.0 (0.1) | 0.0 (0.1) | 0.0 (0.0) | 0.0 (0.0) | 0.0 (0.0) | 0.0 (0.0) |

Supplementary Table S4 continued

| Conservation Unit | Strait of Georgia-north sport | | | Strait of Georgia-south sport | | | | | |
| --- | --- | --- | --- | --- | --- | --- | --- | --- | --- |
|  | October | November | December | January | | February | | March | |
|  | Legal | Legal | Legal | Legal | Sublegal | Legal | Sublegal | Legal | Sublegal |
| Sample size | 8 | 4 | 6 | 9 | 47 | 10 | 37 | 57 | 40 |
| N PBT | 1 | 0 | 2 | 0 | 5 | 1 | 4 | 2 | 2 |
| Southeast Alaska | 0.0 (0.7) | 0.0 (0.2) | 0.0 (0.1) | 0.0 (0.4) | 0.0 (0.1) | 0.0 (0.9) | 0.0 (0.3) | 0.0 (0.2) | 0.0 (0.3) |
| Alsek | 0.0 (1.0) | 0.0 (6.7) | 0.0 (3.2) | 0.0 (6.0) | 0.0 (0.4) | 0.0 (1.0) | 0.0 (0.3) | 0.0 (0.5) | 0.0 (0.1) |
| Unuk | 0.0 (0.2) | 0.0 (0.0) | 0.0 (0.0) | 0.0 (0.1) | 0.0 (0.1) | 0.0 (0.1) | 0.0 (0.0) | 0.0 (0.0) | 0.0 (0.0) |
| Taku_early timing | 0.0 (0.0) | 0.0 (0.6) | 0.0 (0.2) | 0.0 (0.0) | 0.0 (0.4) | 0.0 (0.0) | 0.0 (0.4) | 0.0 (0.0) | 0.0 (0.0) |
| Taku_mid timing | 0.0 (0.0) | 0.0 (0.2) | 0.0 (0.0) | 0.0 (0.9) | 0.0 (0.0) | 0.0 (2.2) | 0.0 (0.8) | 0.0 (0.1) | 0.0 (0.1) |
| Taku_late timing | 0.0 (0.0) | 0.0 (1.1) | 0.0 (0.2) | 0.0 (0.1) | 0.0 (0.0) | 0.0 (0.3) | 0.0 (0.0) | 0.0 (0.0) | 0.0 (0.0) |
| Stikine_early timing | 0.0 (0.6) | 0.0 (0.4) | 0.0 (2.0) | 0.0 (0.0) | 0.0 (0.0) | 0.0 (0.0) | 0.0 (0.0) | 0.0 (0.0) | 0.0 (0.3) |
| Stikine_late timing | 0.0 (0.2) | 0.0 (0.0) | 0.0 (0.0) | 0.0 (0.0) | 0.0 (0.0) | 0.0 (2.1) | 0.0 (0.0) | 0.0 (0.0) | 0.0 (0.0) |
| Haida Gwaii-North | 0.0 (0.0) | 0.0 (0.6) | 0.0 (0.0) | 0.0 (0.0) | 0.0 (0.0) | 0.0 (0.0) | 0.0 (0.0) | 0.0 (0.0) | 0.0 (0.1) |
| Upper Nass | 0.0 (1.2) | 0.0 (0.1) | 0.0 (2.1) | 0.0 (0.3) | 0.0 (0.1) | 0.0 (0.8) | 0.0 (0.5) | 0.0 (0.3) | 0.0 (0.0) |
| Portland Sound-Observatory Inlet-Lower Nass | 0.0 (1.7) | 0.0 (2.7) | 0.0 (0.5) | 0.0 (1.5) | 0.0 (0.0) | 0.0 (0.2) | 0.0 (0.0) | 0.0 (0.1) | 0.0 (0.1) |
| Ecstall | 0.0 (0.0) | 0.0 (0.0) | 0.0 (0.0) | 0.0 (0.0) | 0.0 (0.1) | 0.0 (0.0) | 0.0 (0.0) | 0.0 (0.0) | 0.0 (0.0) |
| Skeena Estuary | 0.0 (1.9) | 0.0 (0.0) | 0.0 (0.3) | 0.0 (0.6) | 0.0 (0.0) | 0.0 (0.1) | 0.0 (0.1) | 0.0 (0.0) | 0.0 (0.0) |
| Lower Skeena | 0.0 (0.6) | 0.0 (0.9) | 0.0 (1.4) | 0.0 (0.7) | 0.0 (0.1) | 0.0 (0.1) | 0.0 (1.3) | 0.0 (0.1) | 0.0 (0.0) |
| Kalum_early timing | 0.0 (0.0) | 0.0 (0.0) | 0.0 (2.2) | 0.0 (0.0) | 0.0 (0.0) | 0.0 (0.0) | 0.0 (0.0) | 0.0 (0.0) | 0.0 (0.0) |
| Kalum_late timing | 0.0 (2.9) | 0.0 (0.0) | 0.0 (0.1) | 0.0 (0.4) | 0.0 (0.1) | 0.0 (0.0) | 0.0 (0.0) | 0.0 (0.1) | 0.0 (0.1) |
| Zymoetz | 0.0 (0.8) | 0.0 (0.0) | 0.0 (0.0) | 0.0 (0.0) | 0.0 (0.0) | 0.0 (0.0) | 0.0 (0.0) | 0.0 (0.0) | 0.0 (0.1) |
| Sicintine | 0.0 (0.0) | 0.0 (0.6) | 0.0 (0.0) | 0.0 (0.0) | 0.0 (0.0) | 0.0 (0.1) | 0.0 (0.0) | 0.0 (0.0) | 0.0 (0.0) |
| Middle Skeena-mainstem tributaries | 0.0 (0.6) | 0.0 (0.3) | 0.0 (2.0) | 0.0 (2.1) | 0.0 (0.3) | 0.0 (0.6) | 0.0 (0.2) | 0.0 (0.0) | 0.0 (0.0) |
| Middle Skeena-large lakes | 0.0 (0.1) | 0.0 (0.5) | 0.0 (0.0) | 0.0 (0.0) | 0.0 (0.0) | 0.0 (0.0) | 0.0 (0.0) | 0.0 (0.0) | 0.0 (0.0) |
| Upper Skeena | 0.0 (0.3) | 0.0 (0.8) | 0.0 (0.7) | 0.0 (0.0) | 0.0 (0.2) | 0.0 (1.3) | 0.0 (0.4) | 0.0 (0.2) | 0.0 (0.1) |
| Upper Bulkley River | 0.0 (0.2) | 0.0 (0.3) | 0.0 (0.0) | 0.0 (0.0) | 0.0 (0.0) | 0.0 (0.0) | 0.0 (0.0) | 0.0 (0.0) | 0.0 (0.4) |
| North and Central Coast-late timing | 0.0 (0.0) | 0.0 (0.0) | 0.0 (0.0) | 0.0 (0.0) | 0.0 (0.0) | 0.0 (0.0) | 0.0 (0.1) | 0.0 (0.0) | 0.0 (0.7) |
| North and Central Coast-early timing | 0.0 (0.9) | 0.0 (0.0) | 0.0 (0.0) | 0.0 (0.2) | 0.0 (0.0) | 0.0 (0.2) | 0.0 (0.0) | 0.0 (0.0) | 0.0 (0.0) |
| Rivers Inlet | 0.0 (0.7) | 0.0 (0.2) | 0.0 (0.3) | 0.0 (0.4) | 0.0 (0.0) | 0.0 (1.1) | 0.0 (0.0) | 0.0 (0.0) | 0.0 (0.0) |
| Wannock | 0.0 (1.8) | 0.0 (0.0) | 0.0 (0.2) | 0.0 (0.1) | 0.0 (0.0) | 0.0 (0.3) | 0.0 (0.0) | 0.0 (0.0) | 0.0 (0.0) |
| Bella Coola-Bentinck | 0.0 (0.2) | 0.0 (4.6) | 0.0 (1.0) | 0.0 (1.6) | 0.0 (0.3) | 0.0 (1.5) | 0.0 (0.3) | 0.0 (0.0) | 0.0 (0.1) |
| Dean River | 0.0 (0.6) | 0.0 (2.6) | 0.0 (0.0) | 0.0 (1.3) | 0.0 (0.0) | 0.0 (0.2) | 0.0 (0.0) | 0.0 (0.0) | 0.0 (0.0) |
| Docee | 0.0 (0.0) | 0.0 (0.0) | 0.0 (0.0) | 0.0 (0.8) | 0.0 (0.0) | 0.0 (0.0) | 0.0 (008) | 0.0 (0.0) | 0.0 (0.1) |
| Klinaklini_SU_1.3 | 0.0 (0.0) | 0.0 (0.5) | 0.0 (0.0) | 0.0 (0.0) | 0.0 (0.0) | 0.0 (1.2) | 0.0 (0.0) | 0.0 (0.0) | 0.0 (0.6) |
| Southern Mainland-Southern Fjords_FA_0.x | 0.0 (0.0) | 0.0 (0.0) | 0.0 (0.3) | 0.0 (0.0) | 0.0 (0.0) | 0.0 (0.1) | 0.0 (0.0) | 0.0 (0.1) | 0.0 (0.2) |
| Southern Mainland-Georgia Strait_FA_0.x | 12.5 (10.0) | 0.0 (2.4) | 16.7 (12.1) | 11.1 (8.0) | 4.3 (2.8) | 0.0 (0.4) | 2.7 (3.9) | 0.0 (0.0) | 2.5 (2.7) |
| Upper Fraser River_SP_1.3 | 0.0 (3.2) | 0.0 (5.6) | 0.0 (1.4) | 0.0 (2.5) | 0.0 (0.3) | 0.0 (2.1) | 0.0 (0.1) | 0.0 (0.1) | 0.0 (0.5) |
| Middle Fraser River_SU_1.3 | 0.0 (2.4) | 0.0 (3.3) | 0.0 (1.2) | 0.0 (0.6) | 0.0 (0.4) | 0.0 (2.0) | 0.0 (0.1) | 0.0 (0.3) | 0.0 (0.0) |
| Middle Fraser River_SP_1.3 | 0.0 (0.1) | 0.0 (1.1) | 0.0 (3.5) | 0.0 (3.1) | 0.0 (0.1) | 0.0 (3.8) | 0.0 (0.2) | 0.0 (0.0) | 0.0 (0.1) |
| Middle Fraser River-Portage_FA_1.3 | 0.0 (0.0) | 0.0 (0.0) | 0.0 (0.7) | 0.0 (0.1) | 0.0 (0.0) | 0.0 (0.0) | 0.0 (0.0) | 0.0 (0.2) | 0.0 (0.0) |
| Middle Fraser-Fraser Canyon_SP_1.3 | 0.0 (0.3) | 0.0 (0.2) | 0.0 (1.6) | 0.0 (0.0) | 0.0 (0.0) | 0.0 (0.1) | 0.0 (0.0) | 0.0 (0.5) | 0.0 (0.0) |
| North Thompson_SP_1.3 | 0.0 (0.0) | 0.0 (0.1) | 0.0 (0.0) | 0.0 (0.3) | 0.0 (0.4) | 0.0 (0.1) | 0.0 (0.0) | 0.0 (0.1) | 0.0 (0.3) |
| North Thompson_SU_1.3 | 0.0 (0.8) | 0.0 (0.5) | 0.0 (2.9) | 0.0 (0.6) | 0.0 (0.3) | 0.0 (0.1) | 0.0 (0.3) | 0.0 (0.0) | 0.0 (0.0) |
| Shuswap River_SU_0.3 | 0.0 (0.0) | 0.0 (0.2) | 0.0 (0.1) | 0.0 (0.5) | 0.0 (0.0) | 0.0 (0.1) | 0.0 (0.0) | 0.0 (0.0) | 0.0 (0.1) |
| South Thompson-Bessette Creek_SU_1.2 | 0.0 (1.7) | 0.0 (0.4) | 0.0 (0.6) | 0.0 (0.6) | 0.0 (0.0) | 0.0 (0.0) | 0.0 (0.0) | 0.0 (0.0) | 0.0 (0.3) |
| South Thompson_SU_0.3 | 0.0 (0.3) | 0.0 (1.6) | 0.0 (0.4) | 0.0 (0.6) | 0.0 (0.5) | 0.0 (1.6) | 0.0 (0.0) | 0.0 (0.3) | 0.0 (0.8) |
| South Thompson_SU_1.3 | 0.0 (0.3) | 0.0 (0.1) | 0.0 (1.0) | 0.0 (0.3) | 0.0 (0.7) | 0.0 (0.6) | 0.0 (0.1) | 0.0 (0.4) | 0.0 (1.0) |
| Lower Thompson_SP_1.2 | 0.0 (0.6) | 0.0 (0.4) | 0.0 (0.1) | 0.0 (0.3) | 0.0 (0.6) | 0.0 (4.2) | 0.0 (0.3) | 0.0 (0.4) | 0.0 (0.0) |
| Lower Fraser River_SP_1.3 | 0.0 (0.0) | 0.0 (0.3) | 0.0 (0.0) | 0.0 (0.0) | 0.0 (0.0) | 0.0 (0.0) | 0.0 (0.0) | 0.0 (0.0) | 0.0 (0.1) |
| Lower Fraser River_SU_1.3 | 0.0 (0.0) | 0.0 (1.3) | 0.0 (0.0) | 0.0 (0.2) | 0.0 (0.2) | 0.0 (0.0) | 0.0 (0.0) | 0.0 (0.0) | 0.0 (0.0) |
| Lower Fraser River-Upper Pitt_SU_1.3 | 0.0 (0.0) | 0.0 (4.0) | 0.0 (0.0) | 0.0 (0.0) | 0.0 (0.0) | 0.0 (0.0) | 0.0 (0.0) | 0.0 (0.0) | 0.0 (0.0) |
| Maria Slough_SU_0.3 | 0.0 (0.0) | 0.0 (0.3) | 0.0 (0.0) | 0.0 (0.0) | 0.0 (0.0) | 0.0 (0.0) | 0.0 (0.0) | 0.0 (0.0) | 0.0 (0.0) |
| Lower Fraser River_FA_0.3 | 12.5 (7.2) | 0.0 (0.3) | 16.7 (11.4) | 0.0 (0.4) | 0.0 (0.1) | 0.0 (0.0) | 2.7 (3.1) | 8.8 (3.6) | 5.0 (2.8) |
| East Vancouver Island-North_FA_0.x | 0.0 (1.5) | 0.0 (0.4) | 0.0 (0.7) | 0.0 (0.8) | 0.0 (0.1) | 0.0 (0.2) | 0.0 (0.2) | 0.0 (0.0) | 2.5 (2.1) |
| East Vancouver Island-Qualicum and Puntledge_FA_0.x | 0.0 (0.1) | 0.1 (0.0) | 0.0 (1.2) | 0.0 (0.0) | 16.2 (6.7) | 0.0 (0.0) | 7.6 (4.3) | 0.0 (0.0) | 7.9 (3.5) |
| East Vancouver Island-Nanaimo and Chemainus_FA_0.x | 24.9 (12.6) | 6.6 (17.9) | 0.0 (0.0) | 0.0 (0.1) | 0.1 (0.2) | 0.0 (0.0) | 0.0 (0.0) | 0.0 (0.0) | 0.0 (0.2) |
| East Vancouver Island-Nanaimo_SP_1.x | 0.0 (0.2) | 0.0 (0.0) | 0.0 (0.0) | 0.0 (0.0) | 0.0 (0.0) | 0.0 (0.3) | 0.0 (0.1) | 0.0 (0.0) | 0.0 (0.0) |
| East Vancouver Island-Georgia Strait_SU_0.3 | 0.0 (0.7) | 0.0 (0.9) | 16.7 (11.6) | 0.0 (0.0) | 4.2 (2.5) | 10.0 (8.2) | 0.0 (0.0) | 0.0 (0.5) | 0.0 (0.2) |
| East Vancouver Island-Cowichan and Koksilah_FA_0.x | 37.6 (15.5) | 68.4 (22.0) | 33.3 (19.6) | 0.0 (0.0) | 6.4 (4.0) | 10.0 (8.5) | 8.7 (4.4) | 10.5 (4.1) | 19.7 (6.3) |
| West Vancouver Island-Nootka and Kyuquot_FA_0.x | 0.0 (0.7) | 0.0 (0.7) | 0.0 (3.3) | 0.0 (3.4) | 0.0 (0.0) | 0.0 (0.4) | 0.0 (0.3) | 0.0 (0.2) | 0.0 (0.4) |
| West Vancouver Island-North_FA_0.x | 0.0 (0.2) | 0.0 (0.9) | 0.0 (0.6) | 0.0 (0.6) | 0.0 (0.0) | 0.0 (3.8) | 0.0 (0.0) | 0.0 (0.0) | 0.0 (0.1) |
| West Vancouver Island-South_FA_0.x | 0.0 (1.6) | 0.0 (5.2) | 0.0 (0.5) | 0.0 (0.4) | 0.0 (0.1) | 0.0 (0.9) | 0.0 (0.7) | 0.0 (0.1) | 0.0 (0.7) |
| Okanagan_1.x | 0.0 (0.0) | 0.0 (0.0) | 0.0 (0.0) | 0.0 (0.0) | 0.0 (0.1) | 0.0 (0.0) | 0.0 (0.2) | 0.0 (0.0) | 0.0 (0.0) |
| Juan de Fuca | 0.0 (0.8) | 0.0 (0.0) | 0.0 (0.1) | 0.0 (0.1) | 0.0 (0.1) | 0.0 (0.0) | 0.0 (0.0) | 0.0 (0.0) | 0.0 (0.0) |
| Coastal Washington | 0.0 (0.0) | 0.0 (0.7) | 0.0 (3.7) | 0.0 (0.1) | 0.0 (0.1) | 0.0 (0.6) | 0.0 (0.1) | 0.0 (0.0) | 0.0 (0.2) |
| North Puget Sound | 0.0 (0.9) | 0.0 (0.9) | 0.0 (0.0) | 34.1 (14.2) | 4.6 (4.2) | 0.0 (0.0) | 15.2 (7.0) | 17.5 (6.9) | 26.2 (9.3) |
| South Puget Sound | 12.5 (11.4) | 25.0 (14.6) | 16.7 (11.7) | 54.8 (15.5) | 62.2 (8.8) | 79.9 (12.6) | 63.1 (9.0) | 59.0 (6.8) | 36.1 (7.8) |
| Lower Columbia River | 0.0 (0.4) | 0.0 (1.0) | 0.0 (1.6) | 0.0 (0.6) | 0.3 (0.0) | 0.0 (0.1) | 0.0 (0.0) | 1.7 (1.7) | 0.0 (0.0) |
| Mid Columbia River_SP | 0.0 (1.7) | 0.0 (0.3) | 0.0 (0.0) | 0.0 (4.6) | 1.8 (2.0) | 0.1 (0.6) | 0.0 (0.0) | 2.5 (3.9) | 0.0 (0.4) |
| Upper Columbia River_SP | 0.0 (0.9) | 0.0 (0.6) | 0.0 (1.6) | 0.0 (0.0) | 0.0 (0.0) | 0.0 (0.2) | 0.0 (0.2) | 0.0 (0.1) | 0.0 (0.1) |
| Upper Columbia River_SU_FA | 0.0 (1.1) | 0.0 (0.2) | 0.0 (2.9) | 0.0 (0.3) | 0.0 (0.1) | 0.0 (0.0) | 0.0 (0.0) | 0.0 (0.1) | 0.0 (0.2) |
| Snake River_FA | 0.0 (0.0) | 0.0 (0.8) | 0.0 (0.1) | 0.0 (0.0) | 0.0 (0.0) | 0.0 (0.1) | 0.0 (0.0) | 0.0 (0.0) | 0.0 (0.0) |
| Snake River_SP_SU | 0.0 (1.9) | 0.0 (3.2) | 0.0 (3.9) | 0.0 (1.8) | 0.0 (0.1) | 0.0 (0.6) | 0.0 (0.4) | 0.0 (0.3) | 0.0 (0.3) |
| North & Central Oregon | 0.0 (0.1) | 0.0 (0.2) | 0.0 (0.1) | 0.0 (0.3) | 0.0 (0.1) | 0.0 (1.1) | 0.0 (0.0) | 0.0 (0.2) | 0.0 (0.1) |
| Upper Willamette River | 0.0 (0.0) | 0.0 (0.0) | 0.0 (2.4) | 0.0 (1.0) | 0.0 (0.1) | 0.0 (0.0) | 0.0 (0.0) | 0.0 (0.0) | 0.0 (0.0) |
| South Oregon coastal | 0.0 (0.6) | 0.0 (3.6) | 0.0 (0.3) | 0.0 (1.2) | 0.0 (0.0) | 0.0 (1.4) | 0.0 (0.0) | 0.0 (0.1) | 0.0 (0.2) |
| California Klamath Trinity | 0.0 (0.3) | 0.0 (0.6) | 0.0 (0.4) | 0.0 (0.1) | 0.0 (0.2) | 0.0 (0.2) | 0.0 (0.0) | 0.0 (0.2) | 0.0 (0.0) |
| California Central Valley_Fall | 0.0 (2.2) | 0.0 (3.4) | 0.0 (2.6) | 0.0 (0.1) | 0.0 (0.3) | 0.0 (0.1) | 0.0 (0.3) | 0.0 (0.1) | 0.0 (0.1) |
| California Central Valley_Spring | 0.0 (0.7) | 0.0 (1.2) | 0.0 (0.2) | 0.0 (0.0) | 0.0 (0.1) | 0.0 (0.0) | 0.0 (0.0) | 0.0 (0.0) | 0.0 (0.0) |
| Coastal California | 0.0 (0.0) | 0.0 (0.6) | 0.0 (0.0) | 0.0 (0.0) | 0.0 (0.0) | 0.0 (0.0) | 0.0 (0.1) | 0.0 (0.0) | 0.0 (0.0) |

Supplementary Table S4 continued

| Conservation Unit | Strait of Georgia-south sport | | | | | | | | | |
| --- | --- | --- | --- | --- | --- | --- | --- | --- | --- | --- |
|  | April | | May | | June | | July | | August | |
|  | Legal | Sublegal | Legal | Sublegal | Legal | Sublegal | Legal | Sublegal | Legal | Sublegal |
| Sample size | 154 | 86 | 144 | 50 | 85 | 21 | 101 | 33 | 64 | 48 |
| N PBT | 34 | 24 | 7 | 9 | 13 | 3 | 7 | 3 | 10 | 2 |
| Southeast Alaska | 0.0 (0.0) | 0.0 (0.2) | 0.0 (0.0) | 0.0 (0.0) | 0.0 (0.0) | 0.0 (0.5) | 0.0 (0.1) | 0.0 (1.9) | 0.0 (0.2) | 0.0 (0.1) |
| Alsek | 0.0 (0.1) | 0.0 (0.0) | 0.0 (0.0) | 0.0 (0.0) | 0.0 (0.0) | 0.0 (0.2) | 0.0 (0.1) | 0.0 (0.0) | 0.0 (0.1) | 0.0 (0.1) |
| Unuk | 0.0 (0.0) | 0.0 (0.0) | 0.0 (0.0) | 0.0 (0.0) | 0.0 (0.0) | 0.0 (0.0) | 0.0 (0.0) | 0.0 (0.0) | 0.0 (0.0) | 0.0 (0.0) |
| Taku_early timing | 0.0 (0.1) | 0.0 (0.0) | 0.0 (0.1) | 0.0 (0.0) | 0.0 (0.1) | 0.0 (0.0) | 0.0 (0.0) | 0.0 (0.3) | 0.0 (0.2) | 0.0 (0.0) |
| Taku_mid timing | 0.0 (0.0) | 0.0 (0.0) | 0.0 (0.0) | 0.0 (0.0) | 0.0 (0.0) | 0.0 (2.3) | 0.0 (0.1) | 0.0 (1.3) | 0.0 (0.2) | 0.0 (0.0) |
| Taku_late timing | 0.0 (0.0) | 0.0 (0.0) | 0.0 (0.0) | 0.0 (0.0) | 0.0 (0.0) | 0.0 (0.0) | 0.0 (0.0) | 0.0 (0.0) | 0.0 (0.0) | 0.0 (0.0) |
| Stikine_early timing | 0.0 (0.0) | 0.0 (0.2) | 0.0 (0.1) | 0.0 (0.0) | 0.0 (0.1) | 0.0 (1.0) | 0.0 (0.0) | 0.0 (0.1) | 0.0 (0.0) | 0.0 (0.2) |
| Stikine_late timing | 0.0 (0.0) | 0.0 (0.0) | 0.0 (0.0) | 0.0 (0.0) | 0.0 (0.1) | 0.0 (0.1) | 0.0 (0.0) | 0.0 (0.2) | 0.0 (0.1) | 0.0 (0.1) |
| Haida Gwaii-North | 0.0 (0.0) | 0.0 (0.0) | 0.0 (0.0) | 0.0 (0.0) | 0.0 (0.0) | 0.0 (0.0) | 0.0 (0.0) | 0.0 (0.0) | 0.0 (0.0) | 0.0 (0.0) |
| Upper Nass | 0.0 (0.0) | 0.0 (0.1) | 0.0 (0.0) | 0.0 (0.2) | 0.0 (0.2) | 0.0 (0.3) | 0.0 (0.1) | 0.0 (0.4) | 0.0 (0.2) | 0.0 (0.4) |
| Portland Sound-Observatory Inlet-Lower Nass | 0.0 (0.0) | 0.0 (0.0) | 0.0 (0.1) | 0.0 (0.0) | 0.0 (0.0) | 0.0 (0.1) | 0.0 (0.0) | 0.0 (0.4) | 0.0 (0.0) | 0.0 (0.1) |
| Ecstall | 0.0 (0.0) | 0.0 (0.0) | 0.0 (0.1) | 0.0 (0.0) | 0.0 (0.1) | 0.0 (0.0) | 0.0 (0.0) | 0.0 (0.4) | 0.0 (0.0) | 0.0 (0.0) |
| Skeena Estuary | 0.0 (0.0) | 0.0 (0.0) | 0.0 (0.0) | 0.0 (0.3) | 0.0 (0.0) | 0.0 (0.0) | 0.0 (0.0) | 0.0 (0.1) | 0.0 (0.0) | 0.0 (0.0) |
| Lower Skeena | 0.0 (0.1) | 0.0 (0.2) | 0.0 (0.0) | 0.0 (0.3) | 0.0 (0.1) | 0.0 (0.1) | 0.0 (0.0) | 0.0 (0.4) | 0.0 (0.1) | 0.0 (0.1) |
| Kalum_early timing | 0.0 (0.0) | 0.0 (0.0) | 0.0 (0.0) | 0.0 (0.0) | 0.0 (0.0) | 0.0 (0.2) | 0.0 (0.0) | 0.0 (0.0) | 0.0 (0.0) | 0.0 (0.0) |
| Kalum_late timing | 0.0 (0.0) | 0.0 (0.0) | 0.0 (0.0) | 0.0 (0.0) | 0.0 (0.0) | 0.0 (0.2) | 0.0 (0.0) | 0.0 (0.0) | 0.0 (0.0) | 0.0 (0.2) |
| Zymoetz | 0.0 (0.0) | 0.0 (0.1) | 0.0 (0.0) | 0.0 (0.0) | 0.0 (0.0) | 0.0 (0.0) | 0.0 (0.0) | 0.0 (0.0) | 0.0 (0.0) | 0.0 (0.0) |
| Sicintine | 0.0 (0.0) | 0.0 (0.0) | 0.0 (0.0) | 0.0 (0.0) | 0.0 (0.0) | 0.0 (0.0) | 0.0 (0.0) | 0.0 (0.0) | 0.0 (0.0) | 0.0 (0.0) |
| Middle Skeena-mainstem tributaries | 0.0 (0.2) | 0.0 (0.4) | 0.0 (0.2) | 0.0 (0.1) | 0.0 (0.1) | 0.0 (0.5) | 0.0 (0.1) | 0.0 (0.7) | 0.0 (0.5) | 0.0 (0.1) |
| Middle Skeena-large lakes | 0.0 (0.2) | 0.0 (0.1) | 0.0 (0.0) | 0.0 (0.1) | 0.0 (0.0) | 0.0 (0.0) | 0.0 (0.0) | 0.0 (0.8) | 0.0 (0.4) | 0.0 (0.0) |
| Upper Skeena | 0.0 (0.0) | 0.0 (0.2) | 0.0 (0.1) | 0.0 (0.0) | 0.0 (0.2) | 0.0 (0.1) | 0.0 (0.0) | 0.0 (0.1) | 0.0 (0.0) | 0.0 (0.0) |
| Upper Bulkley River | 0.0 (0.0) | 0.0 (0.0) | 0.0 (0.0) | 0.0 (0.0) | 0.0 (0.0) | 0.0 (0.0) | 0.0 (0.0) | 0.0 (0.0) | 0.0 (0.0) | 0.0 (0.0) |
| North and Central Coast-late timing | 0.0 (0.0) | 0.0 (0.1) | 0.0 (0.0) | 0.0 (0.1) | 0.0 (0.4) | 0.0 (0.0) | 0.0 (0.0) | 0.0 (0.1) | 0.0 (0.0) | 0.0 (0.0) |
| North and Central Coast-early timing | 0.0 (0.0) | 0.0 (0.0) | 0.0 (0.0) | 0.0 (0.0) | 0.0 (0.0) | 0.0 (0.1) | 0.0 (0.0) | 0.0 (0.1) | 0.0 (0.0) | 0.0 (0.0) |
| Rivers Inlet | 0.0 (0.0) | 0.0 (0.0) | 0.0 (0.1) | 0.0 (0.6) | 0.0 (0.2) | 0.0 (0.0) | 0.0 (0.0) | 0.0 (0.2) | 0.0 (0.3) | 0.0 (0.0) |
| Wannock | 0.0 (0.0) | 0.0 (0.0) | 0.0 (0.0) | 0.0 (0.0) | 0.0 (0.0) | 0.0 (0.0) | 0.0 (0.0) | 0.0 (0.0) | 0.0 (0.0) | 0.0 (0.0) |
| Bella Coola-Bentinck | 0.0 (0.0) | 0.0 (0.1) | 0.0 (0.0) | 0.0 (0.1) | 0.0 (0.2) | 0.0 (0.0) | 0.0 (0.2) | 0.0 (0.4) | 0.0 (0.0) | 0.0 (0.4) |
| Dean River | 0.0 (0.0) | 0.0 (0.0) | 0.0 (0.0) | 0.0 (0.0) | 0.0 (0.0) | 0.0 (0.1) | 0.0 (0.0) | 0.0 (0.1) | 0.0 (0.0) | 0.0 (0.6) |
| Docee | 0.0 (0.0) | 0.0 (0.0) | 0.0 (0.0) | 0.0 (0.1) | 0.0 (0.0) | 0.0 (0.0) | 0.0 (0.0) | 0.0 (0.1) | 0.0 (0.0) | 0.0 (0.0) |
| Klinaklini_SU_1.3 | 0.0 (0.0) | 0.0 (0.0) | 0.0 (0.0) | 0.0 (0.0) | 0.0 (0.0) | 0.0 (0.1) | 0.0 (0.0) | 0.0 (0.3) | 0.0 (0.1) | 0.0 (0.1) |
| Southern Mainland-Southern Fjords_FA_0.x | 0.0 (0.0) | 0.0 (0.0) | 0.0 (0.0) | 0.0 (0.0) | 0.0 (0.0) | 0.0 (0.8) | 0.0 (0.0) | 0.0 (0.0) | 0.0 (0.0) | 0.0 (0.4) |
| Southern Mainland-Georgia Strait_FA_0.x | 0.0 (0.1) | 1.2 (1.0) | 0.2 (0.5) | 4.0 (2.5) | 2.0 (1.9) | 14.3 (7.5) | 0.0 (0.1) | 0.0 (0.4) | 0.0 (0.3) | 0.0 (0.4) |
| Upper Fraser River_SP_1.3 | 0.0 (0.1) | 0.0 (0.3) | 0.5 (0.8) | 0.0 (0.4) | 0.0 (0.2) | 0.0 (0.5) | 0.0 (0.1) | 0.0 (0.5) | 0.0 (0.4) | 0.0 (0.7) |
| Middle Fraser River_SU_1.3 | 0.0 (0.0) | 0.0 (0.1) | 0.0 (0.0) | 0.0 (0.1) | 0.0 (0.0) | 0.0 (0.7) | 0.9 (0.8) | 0.0 (0.4) | 0.0 (0.0) | 6.2 (3.4) |
| Middle Fraser River_SP_1.3 | 0.0 (0.2) | 0.0 (0.3) | 0.2 (0.6) | 0.0 (0.1) | 0.0 (0.3) | 0.0 (0.3) | 0.0 (0.1) | 0.0 (1.0) | 0.0 (0.4) | 0.0 (0.5) |
| Middle Fraser River-Portage_FA_1.3 | 0.0 (0.0) | 0.0 (0.0) | 0.0 (0.0) | 0.0 (0.0) | 0.0 (0.0) | 0.0 (0.0) | 0.0 (0.1) | 0.0 (0.6) | 0.0 (0.0) | 0.0 (0.0) |
| Middle Fraser-Fraser Canyon_SP_1.3 | 0.0 (0.0) | 0.0 (0.0) | 0.0 (0.0) | 0.0 (0.1) | 0.0 (0.0) | 0.0 (0.7) | 0.0 (0.0) | 3.0 (2.6) | 0.0 (0.0) | 0.0 (0.0) |
| North Thompson_SP_1.3 | 0.0 (0.0) | 0.0 (0.0) | 0.0 (0.1) | 0.0 (0.0) | 0.0 (0.0) | 0.0 (0.0) | 0.0 (0.0) | 0.0 (0.0) | 0.0 (0.0) | 0.0 (0.0) |
| North Thompson_SU_1.3 | 0.0 (0.0) | 0.0 (0.2) | 0.0 (0.0) | 0.0 (0.2) | 0.0 (0.3) | 4.8 (4.2) | 0.1 (0.1) | 0.0 (0.1) | 0.0 (0.1) | 0.0 (0.2) |
| Shuswap River_SU_0.3 | 0.0 (0.0) | 0.0 (0.0) | 0.0 (0.0) | 0.0 (0.0) | 0.0 (0.0) | 0.0 (0.1) | 2.0 (1.2) | 6.1 (3.5) | 4.7 (2.4) | 6.7 (4.2) |
| South Thompson-Bessette Creek_SU_1.2 | 0.0 (0.0) | 0.0 (0.2) | 0.0 (0.0) | 0.0 (0.1) | 0.0 (0.1) | 0.0 (0.0) | 0.0 (0.0) | 0.0 (0.0) | 0.0 (0.0) | 0.0 (0.0) |
| South Thompson_SU_0.3 | 0.0 (0.0) | 0.0 (0.0) | 0.0 (0.0) | 0.0 (0.2) | 0.0 (0.1) | 0.0 (0.3) | 0.0 (0.0) | 0.0 (0.0) | 25.0 (5.2) | 26.6 (6.0) |
| South Thompson_SU_1.3 | 0.0 (0.0) | 0.0 (0.0) | 0.0 (0.0) | 0.0 (0.1) | 0.0 (0.0) | 0.0 (0.6) | 0.0 (0.0) | 0.0 (0.0) | 0.0 (0.0) | 0.0 (0.0) |
| Lower Thompson_SP_1.2 | 0.0 (0.1) | 0.0 (0.1) | 0.0 (0.1) | 0.0 (0.1) | 1.2 (1.3) | 0.0 (1.8) | 0.0 (0.0) | 0.0 (0.2) | 0.0 (0.0) | 2.1 (1.7) |
| Lower Fraser River_SP_1.3 | 0.0 (0.0) | 0.0 (0.0) | 0.0 (0.0) | 0.0 (0.0) | 0.0 (0.0) | 0.0 (0.0) | 0.0 (0.2) | 3.0 (2.5) | 0.0 (0.2) | 0.0 (0.0) |
| Lower Fraser River_SU_1.3 | 0.0 (0.0) | 0.0 (0.0) | 0.0 (0.0) | 0.0 (0.0) | 0.0 (0.0) | 0.0 (0.0) | 0.0 (0.0) | 0.0 (0.0) | 0.0 (0.0) | 0.0 (0.0) |
| Lower Fraser River-Upper Pitt_SU_1.3 | 0.0 (0.1) | 0.0 (0.0) | 0.0 (0.1) | 0.0 (0.2) | 0.0 (0.0) | 0.0 (0.3) | 0.0 (0.0) | 0.0 (0.0) | 0.0 (0.0) | 0.0 (0.0) |
| Maria Slough_SU_0.3 | 0.0 (0.0) | 0.0 (0.0) | 0.0 (0.0) | 0.0 (0.0) | 0.0 (0.0) | 0.0 (0.0) | 0.0 (0.0) | 0.0 (0.0) | 0.0 (0.0) | 0.0 (0.0) |
| Lower Fraser River_FA_0.3 | 67.5 (4.2) | 40.7 (5.7) | 32.6 (4.1) | 24.0 (5.9) | 25.8 (4.6) | 4.8 (4.9) | 16.8 (3.2) | 9.1 (4.2) | 32.8 (5.3) | 6.3 (2.9) |
| East Vancouver Island-North_FA_0.x | 0.0 (0.0) | 0.0 (0.1) | 0.0 (0.0) | 0.0 (0.1) | 1.2 (1.1) | 0.0 (0.4) | 0.0 (0.0) | 0.0 (0.1) | 0.0 (0.1) | 0.0 (0.0) |
| East Vancouver Island-Qualicum and Puntledge_FA_0.x | 2.0 (1.1) | 5.3 (2.3) | 3.8 (1.8) | 5.9 (3.2) | 5.9 (2.5) | 6.8 (8.3) | 2.4 (1.4) | 3.4 (3.8) | 7.2 (3.5) | 3.2 (3.5) |
| East Vancouver Island-Nanaimo and Chemainus_FA_0.x | 0.3 (0.7) | 0.0 (0.0) | 0.4 (1.0) | 0.0 (0.1) | 0.0 (0.3) | 7.4 (4.3) | 1.0 (0.8) | 0.0 (0.0) | 0.0 (0.0) | 0.0 (0.0) |
| East Vancouver Island-Nanaimo_SP_1.x | 0.0 (0.0) | 0.0 (0.0) | 0.0 (0.0) | 0.0 (0.0) | 0.0 (0.0) | 0.0 (0.0) | 0.0 (0.0) | 0.0 (0.1) | 0.0 (0.0) | 0.0 (0.0) |
| East Vancouver Island-Georgia Strait_SU_0.3 | 0.8 (0.7) | 0.0 (0.1) | 0.5 (0.6) | 0.0 (0.1) | 1.2 (1.1) | 0.0 (0.1) | 0.0 (0.1) | 0.0 (0.1) | 0.0 (0.0) | 0.0 (0.0) |
| East Vancouver Island-Cowichan and Koksilah_FA_0.x | 6.6 (1.9) | 14.4 (3.9) | 5.6 (2.3) | 8.1 (3.4) | 12.2 (4.2) | 14.4 (7.4) | 13.5 (3.4) | 8.7 (4.7) | 6.9 (3.4) | 5.3 (3.4) |
| West Vancouver Island-Nootka and Kyuquot_FA_0.x | 0.0 (0.0) | 0.0 (0.1) | 0.0 (0.1) | 0.0 (0.7) | 0.0 (0.2) | 0.0 (0.8) | 0.0 (0.0) | 0.0 (0.1) | 0.0 (0.1) | 0.2 (0.3) |
| West Vancouver Island-North_FA_0.x | 0.0 (0.0) | 0.0 (0.0) | 0.0 (0.0) | 0.0 (0.3) | 0.0 (0.0) | 0.0 (0.7) | 0.0 (0.0) | 0.0 (0.0) | 0.0 (0.0) | 0.0 (0.1) |
| West Vancouver Island-South_FA_0.x | 0.0 (0.1) | 0.0 (0.2) | 0.0 (0.1) | 0.0 (0.2) | 0.0 (0.2) | 0.0 (0.9) | 1.0 (0.8) | 0.0 (0.7) | 0.0 (0.1) | 10.2 (4.5) |
| Okanagan_1.x | 0.0 (0.0) | 0.0 (0.0) | 0.0 (0.0) | 0.0 (0.1) | 0.0 (0.0) | 0.0 (0.0) | 0.0 (0.0) | 0.0 (0.0) | 0.0 (0.0) | 0.0 (0.0) |
| Juan de Fuca | 0.0 (0.0) | 1.2 (0.8) | 0.0 (0.0) | 0.0 (0.0) | 0.0 (0.0) | 0.0 (0.1) | 0.0 (0.0) | 3.0 (2.9) | 0.0 (0.0) | 0.0 (0.0) |
| Coastal Washington | 0.0 (0.2) | 0.0 (0.1) | 0.0 (0.1) | 0.0 (0.0) | 0.0 (0.1) | 0.0 (0.0) | 0.0 (0.1) | 0.0 (0.1) | 0.0 (0.1) | 0.1 (1.0) |
| North Puget Sound | 5.4 (2.1) | 11.1 (4.0) | 8.6 (3.3) | 2.3 (2.3) | 14.5 (3.9) | 4.8 (4.0) | 9.9 (3.2) | 6.2 (9.3) | 3.4 (2.7) | 0.1 (0.1) |
| South Puget Sound | 16.0 (3.2) | 26.0 (4.7) | 45.4 (4.4) | 55.6 (6.4) | 36.1 (5.4) | 42.8 (9.9) | 52.5 (5.9) | 57.4 (9.1) | 19.8 (5.3) | 30.7 (4.9) |
| Lower Columbia River | 0.0 (0.0) | 0.0 (0.2) | 0.0 (0.1) | 0.0 (0.0) | 0.0 (0.0) | 0.0 (0.0) | 0.0 (0.1) | 0.0 (0.5) | 0.0 (0.0) | 0.0 (0.1) |
| Mid Columbia River_SP | 0.0 (0.1) | 0.1 (1.4) | 2.2 (1.4) | 0.0 (0.4) | 0.0 (0.1) | 0.0 (0.1) | 0.0 (0.0) | 0.0 (0.3) | 0.2 (0.9) | 0.2 (0.7) |
| Upper Columbia River_SP | 0.0 (0.1) | 0.0 (0.0) | 0.0 (0.0) | 0.0 (0.0) | 0.0 (0.0) | 0.0 (0.0) | 0.0 (0.1) | 0.0 (0.5) | 0.0 (0.0) | 0.0 (0.2) |
| Upper Columbia River_SU_FA | 0.0 (0.0) | 0.0 (0.0) | 0.0 (0.0) | 0.0 (0.1) | 0.0 (0.0) | 0.0 (0.2) | 0.0 (0.0) | 0.0 (0.0) | 0.0 (0.1) | 0.1 (1.0) |
| Snake River_FA | 0.0 (0.0) | 0.0 (0.0) | 0.0 (0.0) | 0.0 (0.0) | 0.0 (0.5) | 0.0 (0.0) | 0.0 (0.0) | 0.0 (0.0) | 0.0 (0.0) | 2.0 (1.4) |
| Snake River_SP_SU | 0.0 (0.1) | 0.0 (0.0) | 0.0 (0.0) | 0.0 (0.0) | 0.0 (0.4) | 0.0 (1.2) | 0.0 (0.1) | 0.0 (0.6) | 0.0 (0.2) | 0.0 (0.2) |
| North & Central Oregon | 0.0 (0.0) | 0.0 (0.0) | 0.0 (0.0) | 0.0 (0.2) | 0.0 (0.0) | 0.0 (1.1) | 0.0 (0.2) | 0.0 (0.0) | 0.0 (0.0) | 0.0 (1.0) |
| Upper Willamette River | 1.3 (1.0) | 0.0 (0.0) | 0.0 (0.0) | 0.0 (0.1) | 0.0 (0.0) | 0.0 (0.6) | 0.0 (0.1) | 0.0 (0.2) | 0.0 (0.0) | 0.0 (0.1) |
| South Oregon coastal | 0.0 (0.1) | 0.0 (0.0) | 0.0 (0.0) | 0.0 (0.1) | 0.0 (0.1) | 0.0 (1.0) | 0.0 (0.1) | 0.0 (0.0) | 0.0 (0.1) | 0.0 (0.0) |
| California Klamath Trinity | 0.0 (0.0) | 0.0 (0.0) | 0.0 (0.0) | 0.0 (0.0) | 0.0 (0.1) | 0.0 (0.0) | 0.0 (0.0) | 0.0 (0.9) | 0.0 (0.0) | 0.0 (0.0) |
| California Central Valley_Fall | 0.0 (0.0) | 0.0 (0.1) | 0.0 (0.1) | 0.0 (0.6) | 0.0 (0.2) | 0.0 (0.2) | 0.0 (0.1) | 0.0 (0.2) | 0.0 (0.0) | 0.0 (0.2) |
| California Central Valley_Spring | 0.0 (0.0) | 0.0 (0.1) | 0.0 (0.1) | 0.0 (0.0) | 0.0 (0.0) | 0.0 (0.0) | 0.0 (0.0) | 0.0 (0.0) | 0.0 (0.0) | 0.0 (0.0) |
| Coastal California | 0.0 (0.2) | 0.0 (0.0) | 0.0 (0.0) | 0.0 (0.0) | 0.0 (0.0) | 0.0 (0.2) | 0.0 (0.0) | 0.0 (0.0) | 0.0 (0.0) | 0.0 (0.0) |

Supplementary Table S4 continued

| Conservation Unit | Strait of Georgia-south sport | | | | | | | | Capilano derby | JDF sport |
| --- | --- | --- | --- | --- | --- | --- | --- | --- | --- | --- |
|  | September | | October | | November | | December | | July | January |
|  | Legal | Sublegal | Legal | Sublegal | Legal | Sublegal | Legal | Sublegal |  |  |
| Sample size | 28 | 19 | 6 | 47 | 4 | 9 | 12 | 34 | 49 | 7 |
| N PBT | 9 | 3 | 0 | 0 | 0 | 0 | 2 | 1 | 34 | 0 |
| Southeast Alaska | 0.0 (0.1) | 0.0 (0.0) | 0.0 (2.8) | 0.0 (0.0) | 0.0 (0.7) | 0.0 (0.2) | 0.0 (0.9) | 0.0 (0.3) | 0.0 (0.2) | 0.0 (1.7) |
| Alsek | 0.0 (0.0) | 0.0 (0.0) | 0.0 (0.0) | 0.0 (0.0) | 0.0 (0.2) | 0.0 (0.4) | 0.0 (1.5) | 0.0 (0.2) | 0.0 (0.1) | 0.0 (0.2) |
| Unuk | 0.0 (0.1) | 0.0 (0.1) | 0.0 (0.0) | 0.0 (0.0) | 0.0 (0.0) | 0.0 (0.1) | 0.0 (0.0) | 0.0 (0.2) | 0.0 (0.0) | 0.0 (0.2) |
| Taku_early timing | 0.0 (0.0) | 0.0 (0.0) | 0.0 (1.0) | 0.0 (0.0) | 0.0 (3.0) | 0.0 (0.1) | 0.0 (0.1) | 0.0 (0.2) | 0.0 (0.0) | 0.0 (0.1) |
| Taku_mid timing | 0.0 (0.1) | 0.0 (0.2) | 0.0 (0.0) | 0.0 (0.0) | 0.0 (1.3) | 0.0 (0.0) | 0.0 (0.2) | 0.0 (1.3) | 0.0 (0.1) | 0.0 (2.7) |
| Taku_late timing | 0.0 (0.0) | 0.0 (0.0) | 0.0 (0.0) | 0.0 (0.0) | 0.0 (0.2) | 0.0 (0.3) | 0.0 (1.4) | 0.0 (0.0) | 0.0 (0.1) | 0.0 (0.3) |
| Stikine_early timing | 0.0 (0.1) | 0.0 (0.3) | 0.0 (0.0) | 0.0 (0.0) | 0.0 (5.0) | 0.0 (2.1) | 0.0 (0.1) | 0.0 (0.1) | 0.0 (0.2) | 0.0 (0.0) |
| Stikine_late timing | 0.0 (0.0) | 0.0 (0.5) | 0.0 (0.3) | 0.0 (0.0) | 0.0 (0.1) | 0.0 (0.3) | 0.0 (1.1) | 0.0 (0.7) | 0.0 (0.0) | 0.0 (0.2) |
| Haida Gwaii-North | 0.0 (0.0) | 0.0 (0.0) | 0.0 (0.0) | 0.0 (0.0) | 0.0 (0.0) | 0.0 (0.0) | 0.0 (0.2) | 0.0 (0.0) | 0.0 (0.0) | 0.0 (0.0) |
| Upper Nass | 0.0 (0.2) | 0.0 (0.7) | 0.0 (5.9) | 0.0 (0.4) | 0.0 (2.0) | 0.0 (1.7) | 0.0 (0.7) | 0.0 (0.0) | 0.0 (0.1) | 0.0 (0.0) |
| Portland Sound-Observatory Inlet-Lower Nass | 0.0 (0.1) | 0.0 (0.1) | 0.0 (0.3) | 0.0 (0.1) | 0.0 (1.8) | 0.0 (0.0) | 0.0 (1.1) | 0.0 (0.3) | 0.0 (0.1) | 0.0 (1.7) |
| Ecstall | 0.0 (0.0) | 0.0 (0.3) | 0.0 (0.0) | 0.0 (0.0) | 0.0 (0.0) | 0.0 (0.0) | 0.0 (0.0) | 0.0 (0.1) | 0.0 (0.0) | 0.0 (1.7) |
| Skeena Estuary | 0.0 (0.0) | 0.0 (0.0) | 0.0 (0.0) | 0.0 (0.0) | 0.0 (0.0) | 0.0 (0.2) | 0.0 (0.0) | 0.0 (0.0) | 0.0 (0.0) | 0.0 (0.0) |
| Lower Skeena | 0.0 (0.1) | 0.0 (0.4) | 0.0 (0.1) | 0.0 (0.1) | 0.0 (0.4) | 0.0 (1.3) | 0.0 (1.5) | 0.0 (0.2) | 0.0 (0.7) | 0.0 (1.6) |
| Kalum_early timing | 0.0 (0.0) | 0.0 (0.0) | 0.0 (0.0) | 0.0 (0.0) | 0.0 (0.7) | 0.0 (1.5) | 0.0 (0.1) | 0.0 (0.0) | 0.0 (0.2) | 0.0 (0.7) |
| Kalum_late timing | 0.0 (0.0) | 0.0 (0.0) | 0.0 (0.0) | 0.0 (0.0) | 0.0 (0.0) | 0.0 (0.6) | 0.0 (0.0) | 0.0 (0.0) | 0.0 (0.0) | 0.0 (0.1) |
| Zymoetz | 0.0 (0.0) | 0.0 (0.0) | 0.0 (0.0) | 0.0 (0.0) | 0.0 (0.9) | 0.0 (0.0) | 0.0 (0.8) | 0.0 (0.0) | 0.0 (0.1) | 0.0 (0.1) |
| Sicintine | 0.0 (0.0) | 0.0 (0.0) | 0.0 (0.0) | 0.0 (0.0) | 0.0 (0.0) | 0.0 (0.0) | 0.0 (0.0) | 0.0 (0.0) | 0.0 (0.2) | 0.0 (0.3) |
| Middle Skeena-mainstem tributaries | 0.0 (0.2) | 0.0 (0.8) | 0.0 (0.3) | 0.0 (0.1) | 0.0 (2.4) | 0.0 (0.4) | 0.0 (1.9) | 0.0 (0.7) | 0.0 (0.3) | 0.0 (0.0) |
| Middle Skeena-large lakes | 0.0 (0.1) | 0.0 (0.5) | 0.0 (0.9) | 0.0 (0.1) | 0.0 (2.8) | 0.0 (3.0) | 0.0 (0.0) | 0.0 (0.1) | 0.0 (0.0) | 0.0 (0.2) |
| Upper Skeena | 0.0 (0.1) | 0.0 (0.6) | 0.0 (0.1) | 0.0 (0.2) | 0.0 (0.0) | 0.0 (0.7) | 0.0 (3.2) | 0.0 (0.1) | 0.0 (0.0) | 0.0 (0.0) |
| Upper Bulkley River | 0.0 (0.0) | 0.0 (0.0) | 0.0 (0.1) | 0.0 (0.1) | 0.0 (0.0) | 0.0 (0.3) | 0.0 (0.0) | 0.0 (0.1) | 0.0 (0.0) | 0.0 (0.0) |
| North and Central Coast-late timing | 0.0 (1.1) | 0.0 (0.0) | 0.0 (0.8) | 0.0 (0.0) | 0.0 (1.6) | 0.0 (0.0) | 0.0 (0.0) | 0.0 (0.1) | 0.0 (0.0) | 0.0 (0.3) |
| North and Central Coast-early timing | 0.0 (0.0) | 0.0 (0.5) | 0.0 (0.0) | 0.0 (0.3) | 0.0 (1.0) | 0.0 (1.3) | 0.0 (1.7) | 0.0 (0.0) | 0.0 (0.1) | 0.0 (0.1) |
| Rivers Inlet | 0.0 (0.4) | 0.0 (0.4) | 0.0 (0.5) | 0.0 (0.0) | 0.0 (0.6) | 0.0 (0.7) | 0.0 (0.5) | 0.0 (0.3) | 0.0 (0.0) | 0.0 (0.0) |
| Wannock | 0.0 (0.0) | 0.0 (0.0) | 0.0 (0.0) | 0.0 (0.0) | 0.0 (0.0) | 0.0 (1.5) | 0.0 (0.1) | 0.0 (0.0) | 0.0 (0.0) | 0.0 (1.4) |
| Bella Coola-Bentinck | 0.0 (0.1) | 0.0 (0.7) | 0.0 (2.5) | 0.0 (0.0) | 0.0 (1.1) | 0.0 (0.1) | 0.0 (0.0) | 0.0 (0.0) | 0.0 (0.0) | 0.0 (0.0) |
| Dean River | 0.0 (1.1) | 0.0 (0.0) | 0.0 (0.2) | 0.0 (0.0) | 0.0 (0.1) | 0.0 (0.0) | 0.0 (0.2) | 0.0 (0.1) | 0.0 (0.1) | 0.0 (0.0) |
| Docee | 0.0 (0.0) | 0.0 (0.0) | 0.0 (0.0) | 0.0 (0.0) | 0.0 (0.0) | 0.0 (0.1) | 0.0 (0.0) | 0.0 (0.0) | 0.0 (0.0) | 0.0 (3.9) |
| Klinaklini_SU_1.3 | 0.0 (0.0) | 0.0 (0.0) | 0.0 (0.0) | 0.0 (0.1) | 0.0 (0.2) | 0.0 (0.5) | 0.0 (0.0) | 0.0 (0.2) | 0.0 (0.1) | 0.0 (3.2) |
| Southern Mainland-Southern Fjords_FA_0.x | 0.0 (0.0) | 0.0 (0.1) | 0.0 (0.0) | 0.0 (0.1) | 0.0 (0.0) | 0.0 (0.3) | 0.0 (0.0) | 0.0 (0.6) | 0.0 (0.0) | 0.0 (0.0) |
| Southern Mainland-Georgia Strait_FA_0.x | 0.0 (0.6) | 0.0 (0.3) | 33.3 (16.9) | 0.0 (0.8) | 1.2 (1.9) | 0.0 (0.3) | 0.0 (1.6) | 2.9 (2.3) | 0.0 (0.2) | 0.0 (0.0) |
| Upper Fraser River_SP_1.3 | 0.0 (0.4) | 0.0 (1.0) | 0.0 (0.8) | 1.9 (2.1) | 0.0 (5.0) | 1.2 (3.2) | 0.0 (3.0) | 0.0 (0.2) | 0.0 (0.3) | 0.0 (0.5) |
| Middle Fraser River_SU_1.3 | 0.0 (0.4) | 0.0 (0.1) | 0.0 (0.7) | 2.1 (2.6) | 0.0 (3.1) | 0.0 (0.6) | 0.0 (0.9) | 0.0 (0.2) | 0.0 (0.2) | 0.0 (0.0) |
| Middle Fraser River_SP_1.3 | 0.0 (0.5) | 0.0 (0.2) | 0.0 (1.5) | 2.4 (2.7) | 0.0 (1.2) | 9.9 (12.2) | 0.0 (0.0) | 0.0 (0.2) | 0.0 (0.3) | 0.0 (1.6) |
| Middle Fraser River-Portage_FA_1.3 | 0.0 (0.0) | 0.0 (0.0) | 0.0 (0.0) | 0.0 (0.0) | 0.0 (0.0) | 0.0 (0.6) | 0.0 (0.0) | 0.0 (0.0) | 0.0 (0.0) | 0.0 (0.9) |
| Middle Fraser-Fraser Canyon_SP_1.3 | 0.0 (0.0) | 0.0 (0.0) | 0.0 (0.0) | 0.0 (0.0) | 0.0 (0.3) | 0.0 (0.0) | 0.0 (0.0) | 0.0 (0.0) | 0.0 (0.0) | 0.0 (4.4) |
| North Thompson_SP_1.3 | 0.0 (0.1) | 0.0 (0.0) | 0.0 (0.9) | 2.1 (1.7) | 0.0 (1.3) | 22.2 (9.8) | 0.0 (0.0) | 0.0 (0.1) | 0.0 (0.0) | 0.0 (0.0) |
| North Thompson_SU_1.3 | 0.0 (0.6) | 0.0 (0.0) | 0.0 (1.4) | 2.1 (1.8) | 0.0 (4.4) | 0.0 (0.1) | 0.0 (0.5) | 0.0 (0.2) | 0.0 (0.0) | 0.0 (0.0) |
| Shuswap River_SU_0.3 | 0.0 (0.2) | 0.0 (0.0) | 0.0 (0.5) | 2.1 (2.0) | 0.0 (0.0) | 0.0 (0.0) | 0.0 (0.1) | 0.0 (0.1) | 0.0 (0.0) | 0.0 (0.0) |
| South Thompson-Bessette Creek_SU_1.2 | 0.0 (0.0) | 0.0 (0.0) | 0.0 (0.2) | 0.0 (0.0) | 0.0 (0.8) | 0.0 (0.0) | 0.0 (0.1) | 0.0 (0.1) | 0.0 (0.0) | 0.0 (1.4) |
| South Thompson_SU_0.3 | 3.6 (3.3) | 10.5 (6.7) | 0.0 (0.7) | 0.0 (0.0) | 0.0 (0.5) | 0.0 (0.0) | 0.0 (1.1) | 2.9 (3.5) | 0.0 (0.0) | 0.0 (0.0) |
| South Thompson_SU_1.3 | 0.0 (0.1) | 0.0 (0.0) | 0.0 (1.0) | 0.0 (0.0) | 0.0 (0.0) | 0.0 (0.3) | 0.0 (0.0) | 0.0 (0.0) | 0.0 (0.1) | 0.0 (0.1) |
| Lower Thompson_SP_1.2 | 0.0 (0.1) | 10.5 (7.6) | 0.0 (1.2) | 0.0 (0.3) | 0.0 (3.5) | 0.0 (0.0) | 0.0 (2.6) | 0.0 (0.0) | 0.0 (0.1) | 0.0 (0.6) |
| Lower Fraser River_SP_1.3 | 0.0 (0.2) | 0.0 (0.0) | 0.0 (0.3) | 0.0 (0.1) | 0.0 (0.0) | 0.0 (0.0) | 0.0 (0.1) | 0.0 (0.0) | 0.0 (0.0) | 0.0 (2.3) |
| Lower Fraser River_SU_1.3 | 0.0 (0.3) | 0.0 (0.1) | 0.0 (0.2) | 0.0 (0.1) | 0.0 (0.0) | 0.0 (1.2) | 0.0 (0.1) | 0.0 (0.0) | 0.0 (0.0) | 0.0 (0.6) |
| Lower Fraser River-Upper Pitt_SU_1.3 | 0.0 (0.0) | 0.0 (0.1) | 0.0 (0.1) | 0.0 (0.0) | 0.0 (0.0) | 0.0 (0.2) | 0.0 (0.0) | 0.0 (0.0) | 0.0 (0.0) | 0.0 (0.3) |
| Maria Slough_SU_0.3 | 0.0 (0.2) | 0.0 (0.0) | 0.0 (0.0) | 0.0 (0.0) | 0.0 (0.0) | 0.0 (0.0) | 0.0 (0.0) | 0.0 (0.0) | 0.0 (0.0) | 0.0 (1.7) |
| Lower Fraser River_FA_0.3 | 53.6 (8.6) | 15.8 (6.9) | 0.0 (1.2) | 2.1 (1.7) | 0.0 (0.7) | 0.0 (2.0) | 8.3 (5.8) | 5.9 (3.5) | 100.0 (1.6) | 0.0 (2.4) |
| East Vancouver Island-North_FA_0.x | 0.0 (0.0) | 0.0 (1.1) | 0.0 (0.4) | 0.0 (0.4) | 0.0 (0.4) | 0.0 (1.4) | 0.0 (0.2) | 0.0 (0.3) | 0.0 (0.0) | 0.0 (1.1) |
| East Vancouver Island-Qualicum and Puntledge_FA_0.x | 14.3 (6.0) | 5.4 (4.7) | 0.0 (0.5) | 20.0 (5.1) | 22.7 (17.9) | 0.0 (0.0) | 16.7 (9.3) | 9.4 (4.8) | 0.0 (0.0) | 0.0 (1.1) |
| East Vancouver Island-Nanaimo and Chemainus_FA_0.x | 0.0 (0.4) | 0.1 (0.0) | 0.0 (0.3) | 3.9 (2.4) | 0.8 (0.1) | 11.0 (8.4) | 0.0 (0.0) | 0.0 (0.0) | 0.0 (0.0) | 0.0 (0.0) |
| East Vancouver Island-Nanaimo_SP_1.x | 0.0 (0.0) | 0.0 (0.0) | 0.0 (1.9) | 0.0 (0.0) | 0.0 (1.2) | 0.0 (0.0) | 0.0 (0.0) | 0.0 (0.0) | 0.0 (0.0) | 0.0 (0.0) |
| East Vancouver Island-Georgia Strait_SU_0.3 | 0.0 (0.3) | 0.0 (0.1) | 0.0 (1.1) | 0.0 (0.0) | 0.0 (1.2) | 22.3 (9.0) | 0.0 (0.0) | 0.0 (0.0) | 0.0 (0.3) | 0.0 (0.7) |
| East Vancouver Island-Cowichan and Koksilah_FA_0.x | 14.2 (7.4) | 26.1 (8.4) | 0.0 (0.2) | 10.1 (4.2) | 0.0 (0.0) | 11.1 (8.8) | 0.0 (0.7) | 5.0 (4.2) | 0.0 (0.0) | 0.0 (0.2) |
| West Vancouver Island-Nootka and Kyuquot_FA_0.x | 0.0 (0.5) | 0.0 (0.2) | 0.0 (0.4) | 0.0 (0.0) | 0.0 (0.9) | 0.0 (1.2) | 0.0 (0.9) | 0.0 (0.3) | 0.0 (0.1) | 0.0 (0.2) |
| West Vancouver Island-North_FA_0.x | 0.0 (0.0) | 0.0 (1.1) | 0.0 (0.0) | 0.0 (0.0) | 0.0 (0.8) | 0.0 (0.0) | 0.0 (0.1) | 0.0 (0.3) | 0.0 (0.0) | 0.0 (0.0) |
| West Vancouver Island-South_FA_0.x | 0.0 (0.9) | 0.0 (0.1) | 0.0 (2.6) | 12.8 (4.0) | 0.0 (1.5) | 0.0 (0.8) | 0.0 (1.0) | 2.9 (2.0) | 0.0 (0.2) | 0.0 (0.0) |
| Okanagan_1.x | 0.0 (0.0) | 0.0 (0.2) | 0.0 (0.0) | 0.0 (0.0) | 0.0 (0.0) | 0.0 (0.0) | 0.0 (0.0) | 0.0 (0.0) | 0.0 (0.0) | 0.0 (0.0) |
| Juan de Fuca | 0.0 (0.1) | 0.0 (0.5) | 0.0 (0.0) | 0.0 (0.0) | 0.0 (0.0) | 0.0 (0.0) | 0.0 (0.0) | 0.0 (0.0) | 0.0 (0.1) | 0.0 (0.6) |
| Coastal Washington | 0.0 (0.0) | 0.0 (0.5) | 0.0 (0.0) | 0.0 (0.5) | 0.0 (0.8) | 0.0 (0.0) | 0.0 (0.4) | 0.0 (0.1) | 0.0 (0.1) | 0.0 (0.0) |
| North Puget Sound | 0.0 (0.4) | 0.3 (2.0) | 14.4 (9.8) | 10.2 (4.9) | 70.9 (21.5) | 14.3 (12.0) | 55.7 (13.6) | 32.3 (8.2) | 0.0 (0.0) | 47.3 (19.4) |
| South Puget Sound | 14.3 (7.1) | 22.4 (8.6) | 49.9 (17.9) | 23.8 (6.2) | 1.7 (6.5) | 7.9 (11.0) | 19.1 (12.5) | 38.6 (9.1) | 0.0 (0.2) | 43.0 (16.1) |
| Lower Columbia River | 0.0 (0.0) | 0.1 (0.1) | 0.0 (0.0) | 0.0 (0.1) | 0.0 (0.0) | 0.0 (1.5) | 0.0 (0.2) | 0.0 (0.1) | 0.0 (0.1) | 0.0 (0.1) |
| Mid Columbia River_SP | 0.0 (0.1) | 2.7 (5.1) | 0.0 (0.3) | 0.0 (0.1) | 0.7 (0.7) | 0.0 (0.1) | 0.2 (0.0) | 0.0 (0.1) | 0.0 (0.0) | 9.5 (13.6) |
| Upper Columbia River_SP | 0.0 (0.3) | 0.0 (1.0) | 0.0 (0.8) | 0.0 (0.1) | 0.0 (0.8) | 0.0 (0.7) | 0.0 (0.0) | 0.0 (0.4) | 0.0 (0.2) | 0.0 (0.0) |
| Upper Columbia River_SU_FA | 0.0 (0.1) | 0.0 (0.4) | 0.0 (0.8) | 0.0 (0.7) | 0.0 (0.5) | 0.0 (0.6) | 0.0 (0.3) | 0.0 (0.0) | 0.0 (0.2) | 0.0 (0.0) |
| Snake River_FA | 0.0 (0.0) | 0.0 (1.5) | 0.0 (0.0) | 0.0 (0.1) | 0.0 (0.4) | 0.0 (0.1) | 0.0 (0.0) | 0.0 (0.0) | 0.0 (0.0) | 0.0 (0.0) |
| Snake River_SP_SU | 0.0 (0.2) | 0.0 (0.3) | 0.0 (2.6) | 0.0 (0.3) | 0.0 (1.2) | 0.0 (3.4) | 0.0 (0.7) | 0.0 (0.2) | 0.0 (1.2) | 0.0 (2.1) |
| North & Central Oregon | 0.0 (0.5) | 0.0 (0.1) | 0.0 (0.4) | 0.0 (0.1) | 0.0 (2.3) | 0.0 (0.3) | 0.0 (0.5) | 0.0 (0.0) | 0.0 (0.0) | 0.0 (0.0) |
| Upper Willamette River | 0.0 (0.0) | 5.2 (5.3) | 0.0 (1.5) | 0.0 (0.1) | 0.0 (7.0) | 0.0 (0.0) | 0.0 (0.0) | 0.0 (0.0) | 0.0 (0.0) | 0.0 (1.7) |
| South Oregon coastal | 0.0 (0.1) | 0.0 (0.4) | 0.0 (2.0) | 0.0 (0.0) | 0.0 (1.8) | 0.0 (0.6) | 0.0 (0.5) | 0.0 (0.4) | 0.0 (0.4) | 0.0 (0.0) |
| California Klamath Trinity | 0.0 (0.0) | 0.0 (0.2) | 0.0 (0.0) | 0.0 (0.0) | 0.0 (0.3) | 0.0 (0.8) | 0.0 (0.0) | 0.0 (0.0) | 0.0 (0.0) | 0.0 (0.5) |
| California Central Valley_Fall | 0.0 (0.1) | 0.0 (0.4) | 0.0 (0.4) | 0.0 (0.4) | 0.0 (1.3) | 0.0 (2.5) | 0.0 (0.7) | 0.0 (0.1) | 0.0 (0.2) | 0.0 (0.0) |
| California Central Valley_Spring | 0.0 (0.3) | 0.0 (0.1) | 0.0 (0.1) | 0.0 (0.2) | 0.0 (0.0) | 0.0 (0.1) | 0.0 (0.4) | 0.0 (0.2) | 0.0 (0.0) | 0.0 (0.4) |
| Coastal California | 0.0 (0.0) | 0.0 (0.0) | 0.0 (0.2) | 0.0 (0.0) | 0.0 (0.0) | 0.0 (0.0) | 0.0 (0.00 | 0.0 (0.4) | 0.0 (0.0) | 0.0 (0.2) |

Supplementary Table S4 continued

| Conservation Unit | Juan de Fuca Strait sport | | | | | | | | | WCVI-north sport |
| --- | --- | --- | --- | --- | --- | --- | --- | --- | --- | --- |
|  | February | March | April | May | June | July | August | Sept. | October | June |
| Sample size | 29 | 45 | 33 | 46 | 51 | 190 | 260 | 81 | 33 | 11 |
| N PBT | 0 | 0 | 0 | 0 | 4 | 14 | 14 | 8 | 1 | 2 |
| Southeast Alaska | 0.0 (0.1) | 0.0 (0.3) | 0.0 (0.1) | 0.0 (0.8) | 0.0 (0.8) | 0.0 (0.0) | 0.0 (0.1) | 0.0 (0.1) | 0.0 (0.4) | 0.0 (0.4) |
| Alsek | 0.0 (0.0) | 0.0 (0.0) | 0.0 (0.3) | 0.0 (0.1) | 0.0 (0.1) | 0.0 (0.0) | 0.0 (0.0) | 0.0 (0.0) | 0.0 (0.2) | 0.0 (0.6) |
| Unuk | 0.0 (0.0) | 0.0 (0.0) | 0.0 (0.2) | 0.0 (0.0) | 0.0 (0.0) | 0.0 (0.0) | 0.0 (0.0) | 0.0 (0.0) | 0.0 (0.0) | 0.0 (0.2) |
| Taku_early timing | 0.0 (0.3) | 0.0 (0.1) | 0.0 (0.0) | 0.0 (0.1) | 0.0 (0.2) | 0.0 (0.0) | 0.0 (0.0) | 0.0 (0.3) | 0.0 (0.2) | 0.0 (0.1) |
| Taku_mid timing | 0.0 (0.2) | 0.0 (0.1) | 0.0 (0.2) | 0.0 (0.1) | 0.0 (0.2) | 0.0 (0.0) | 0.0 (0.0) | 0.0 (0.0) | 0.0 (0.0) | 0.0 (0.0) |
| Taku_late timing | 0.0 (1.1) | 0.0 (0.1) | 0.0 (0.0) | 0.0 (0.1) | 0.0 (0.9) | 0.0 (0.0) | 0.0 (0.0) | 0.0 (0.0) | 0.0 (0.0) | 0.0 (0.0) |
| Stikine_early timing | 0.0 (0.0) | 0.0 (0.0) | 0.0 (0.2) | 0.0 (0.0) | 0.0 (0.1) | 0.0 (0.0) | 0.0 (0.1) | 0.0 (0.0) | 0.0 (0.4) | 0.0 (1.3) |
| Stikine_late timing | 0.0 (0.0) | 0.0 (0.3) | 0.0 (0.0) | 0.0 (0.0) | 0.0 (0.0) | 0.0 (0.1) | 0.0 (0.0) | 0.0 (0.1) | 0.0 (0.0) | 0.0 (0.0) |
| Haida Gwaii-North | 0.0 (0.0) | 0.0 (0.2) | 0.0 (1.8) | 0.0 (0.0) | 0.0 (0.0) | 0.0 (0.0) | 0.0 (0.0) | 0.0 (0.0) | 0.0 (0.0) | 0.0 (0.0) |
| Upper Nass | 0.0 (0.0) | 0.0 (0.0) | 0.0 (0.0) | 0.0 (0.0) | 0.0 (0.0) | 0.0 (0.1) | 0.0 (0.1) | 0.0 (0.0) | 0.0 (0.2) | 0.0 (1.8) |
| Portland Sound-Observatory Inlet-Lower Nass | 0.0 (0.0) | 0.0 (0.0) | 0.0 (0.0) | 0.0 (0.0) | 0.0 (0.0) | 0.0 (0.0) | 0.0 (0.0) | 0.0 (0.2) | 0.0 (0.2) | 0.0 (0.0) |
| Ecstall | 0.0 (0.0) | 0.0 (0.0) | 0.0 (0.3) | 0.0 (0.2) | 0.0 (0.0) | 0.0 (0.0) | 0.0 (0.0) | 0.0 (0.0) | 0.0 (0.0) | 0.0 (0.0) |
| Skeena Estuary | 0.0 (0.4) | 0.0 (0.0) | 0.0 (0.0) | 0.0 (0.0) | 0.0 (0.0) | 0.0 (0.0) | 0.0 (0.0) | 0.0 (0.1) | 0.0 (0.0) | 0.0 (0.0) |
| Lower Skeena | 0.0 (0.0) | 0.0 (0.1) | 0.0 (0.0) | 0.0 (0.2) | 0.0 (0.1) | 0.0 (0.0) | 0.0 (0.0) | 0.0 (0.1) | 0.0 (0.0) | 0.0 (0.7) |
| Kalum_early timing | 0.0 (0.1) | 0.0 (0.3) | 0.0 (0.2) | 0.0 (0.2) | 0.0 (0.0) | 0.0 (0.0) | 0.0 (0.0) | 0.0 (0.0) | 0.0 (0.0) | 0.0 (0.0) |
| Kalum_late timing | 0.0 (0.1) | 0.0 (0.1) | 0.0 (0.4) | 0.0 (0.3) | 0.0 (0.0) | 0.0 (0.0) | 0.0 (0.0) | 0.0 (0.0) | 0.0 (0.0) | 0.0 (0.0) |
| Zymoetz | 0.0 (0.0) | 0.0 (0.0) | 0.0 (0.0) | 0.0 (0.0) | 0.0 (0.8) | 0.0 (0.0) | 0.0 (0.0) | 0.0 (0.0) | 0.0 (0.1) | 0.0 (0.0) |
| Sicintine | 0.0 (0.3) | 0.0 (0.3) | 0.0 (0.1) | 0.0 (0.3) | 0.0 (0.5) | 0.0 (0.0) | 0.0 (0.0) | 0.0 (0.0) | 0.0 (0.0) | 0.0 (0.0) |
| Middle Skeena-mainstem tributaries | 0.0 (0.0) | 0.0 (0.1) | 0.0 (0.0) | 0.0 (0.0) | 0.0 (0.0) | 0.0 (0.0) | 0.0 (0.0) | 0.0 (0.0) | 0.0 (0.1) | 0.0 (0.7) |
| Middle Skeena-large lakes | 0.0 (0.5) | 0.0 (0.0) | 0.0 (0.0) | 0.0 (0.0) | 0.0 (0.0) | 0.0 (0.0) | 0.0 (0.0) | 0.0 (0.0) | 0.0 (0.1) | 0.0 (0.0) |
| Upper Skeena | 0.0 (0.2) | 0.0 (0.2) | 0.0 (0.0) | 0.0 (0.1) | 0.0 (0.1) | 0.0 (0.0) | 0.0 (0.2) | 0.0 (0.1) | 0.0 (0.2) | 0.0 (0.1) |
| Upper Bulkley River | 0.0 (0.1) | 0.0 (0.1) | 0.0 (0.0) | 0.0 (0.1) | 0.0 (0.0) | 0.0 (0.0) | 0.0 (0.0) | 0.0 (0.0) | 0.0 (0.0) | 0.0 (0.0) |
| North and Central Coast-late timing | 0.0 (0.1) | 0.0 (0.2) | 0.0 (0.1) | 0.0 (0.0) | 0.0 (0.0) | 0.0 (0.0) | 0.0 (0.0) | 0.0 (0.0) | 0.0 (0.0) | 0.0 (0.0) |
| North and Central Coast-early timing | 0.0 (0.0) | 0.0 (0.4) | 0.0 (0.0) | 0.0 (0.0) | 0.0 (0.3) | 0.0 (0.0) | 0.0 (0.0) | 0.0 (0.0) | 0.0 (0.0) | 0.0 (0.0) |
| Rivers Inlet | 0.0 (0.0) | 0.0 (0.0) | 0.0 (0.0) | 0.0 (0.0) | 0.0 (0.0) | 0.0 (0.0) | 0.0 (0.0) | 0.0 (0.2) | 0.0 (0.2) | 0.0 (0.4) |
| Wannock | 0.0 (0.5) | 0.0 (0.0) | 0.0 (1.7) | 0.0 (0.1) | 0.0 (0.2) | 0.0 (0.0) | 0.0 (0.0) | 0.0 (0.1) | 0.0 (0.0) | 0.0 (0.1) |
| Bella Coola-Bentinck | 0.0 (0.9) | 0.0 (0.0) | 0.0 (0.0) | 0.0 (0.0) | 0.0 (0.2) | 0.0 (0.0) | 0.0 (0.0) | 0.0 (0.2) | 0.0 (0.1) | 0.0 (0.0) |
| Dean River | 0.0 (1.2) | 0.0 (0.0) | 0.0 (0.0) | 0.0 (0.0) | 0.0 (0.0) | 0.0 (0.0) | 0.0 (0.0) | 0.0 (0.1) | 0.0 (0.0) | 0.0 (0.3) |
| Docee | 0.0 (0.3) | 0.0 (0.0) | 0.0 (0.0) | 0.0 (0.0) | 0.0 (0.1) | 0.0 (0.0) | 0.0 (0.0) | 0.0 (0.0) | 0.0 (0.0) | 0.0 (0.1) |
| Klinaklini_SU_1.3 | 0.0 (0.6) | 0.0 (0.5) | 0.0 (0.3) | 0.0 (0.3) | 0.0 (0.0) | 0.0 (0.0) | 0.0 (0.0) | 0.0 (0.0) | 0.0 (0.1) | 0.0 (0.0) |
| Southern Mainland-Southern Fjords_FA_0.x | 0.0 (0.0) | 0.0 (0.0) | 0.0 (0.0) | 0.0 (0.0) | 0.0 (0.0) | 0.0 (0.0) | 0.0 (0.0) | 0.0 (0.0) | 0.0 (0.0) | 0.0 (0.0) |
| Southern Mainland-Georgia Strait_FA_0.x | 0.0 (0.1) | 0.0 (0.0) | 0.0 (0.0) | 0.0 (0.0) | 0.0 (0.0) | 0.0 (0.0) | 0.0 (0.0) | 0.0 (0.2) | 0.0 (0.4) | 0.0 (0.5) |
| Upper Fraser River_SP_1.3 | 0.0 (0.0) | 0.0 (0.0) | 0.0 (0.0) | 0.0 (0.0) | 3.9 (4.2) | 2.5 (1.4) | 0.0 (0.1) | 0.0 (0.2) | 0.0 (0.5) | 0.0 (1.7) |
| Middle Fraser River_SU_1.3 | 0.0 (0.0) | 0.0 (0.1) | 0.0 (0.0) | 0.0 (0.0) | 0.0 (0.0) | 4.5 (2.2) | 1.1 (0.6) | 0.0 (0.4) | 0.0 (0.2) | 0.0 (0.3) |
| Middle Fraser River_SP_1.3 | 0.0 (0.5) | 0.0 (0.1) | 0.0 (0.1) | 0.0 (0.2) | 7.8 (4.8) | 0.3 (0.2) | 0.1 (0.3) | 0.0 (0.5) | 0.0 (0.2) | 0.0 (1.9) |
| Middle Fraser River-Portage_FA_1.3 | 0.0 (0.1) | 0.0 (0.0) | 0.0 (0.0) | 0.0 (0.0) | 0.0 (0.0) | 0.0 (0.2) | 0.0 (0.0) | 0.0 (0.0) | 0.0 (0.2) | 0.0 (0.0) |
| Middle Fraser-Fraser Canyon_SP_1.3 | 0.0 (0.1) | 0.0 (0.3) | 0.0 (0.2) | 0.0 (0.1) | 0.0 (0.1) | 0.0 (0.0) | 0.0 (0.0) | 0.0 (0.0) | 0.0 (0.0) | 0.0 (0.0) |
| North Thompson_SP_1.3 | 0.0 (0.0) | 0.0 (0.0) | 0.0 (0.0) | 0.0 (0.0) | 0.0 (0.2) | 2.1 (1.1) | 0.0 (0.0) | 0.0 (0.0) | 0.0 (0.0) | 0.0 (0.2) |
| North Thompson_SU_1.3 | 0.0 (0.1) | 0.0 (0.0) | 0.0 (0.0) | 0.0 (0.0) | 2.0 (2.2) | 2.2 (0.8) | 0.3 (0.5) | 0.0 (0.1) | 0.0 (0.3) | 0.0 (0.0) |
| Shuswap River_SU_0.3 | 0.0 (0.4) | 0.0 (0.0) | 0.0 (0.4) | 0.0 (0.0) | 11.4 (4.7) | 15.3 (2.5) | 3.8 (1.3) | 0.0 (0.0) | 0.0 (0.0) | 0.0 (0.2) |
| South Thompson-Bessette Creek_SU_1.2 | 0.0 (1.4) | 0.0 (0.1) | 0.0 (0.1) | 0.0 (0.0) | 0.0 (0.1) | 0.0 (0.0) | 0.0 (0.0) | 0.0 (0.0) | 0.0 (0.2) | 0.0 (0.0) |
| South Thompson_SU_0.3 | 0.0 (0.6) | 0.0 (0.0) | 0.0 (0.0) | 0.0 (0.0) | 0.4 (0.7) | 4.2 (1.4) | 35.0 (2.7) | 8.6 (3.2) | 0.0 (0.0) | 0.0 (1.7) |
| South Thompson_SU_1.3 | 0.0 (0.0) | 0.0 (0.4) | 0.0 (0.2) | 0.0 (0.0) | 2.0 (1.7) | 0.5 (0.5) | 0.0 (0.0) | 0.0 (0.0) | 0.0 (0.0) | 9.1 (9.2) |
| Lower Thompson_SP_1.2 | 0.0 (0.0) | 0.0 (0.0) | 0.0 (0.0) | 0.0 (0.0) | 2.0 (1.5) | 0.0 (0.0) | 0.0 (0.1) | 0.0 (0.1) | 0.0 (1.5) | 0.0 (1.3) |
| Lower Fraser River_SP_1.3 | 0.0 (1.0) | 0.0 (0.0) | 0.0 (0.0) | 0.0 (0.0) | 0.0 (0.0) | 0.0 (0.0) | 0.0 (0.0) | 0.0 (0.0) | 0.0 (0.0) | 0.0 (0.0) |
| Lower Fraser River_SU_1.3 | 0.0 (0.0) | 0.0 (0.0) | 0.0 (0.0) | 0.0 (0.0) | 0.0 (0.4) | 0.0 (0.0) | 0.0 (0.1) | 0.0 (0.0) | 0.0 (0.4) | 0.0 (0.0) |
| Lower Fraser River-Upper Pitt_SU_1.3 | 0.0 (0.0) | 0.0 (0.0) | 0.0 (0.1) | 0.0 (0.0) | 0.0 (0.0) | 0.0 (0.2) | 0.0 (0.0) | 0.0 (0.0) | 0.0 (0.0) | 0.0 (0.0) |
| Maria Slough_SU_0.3 | 0.0 (0.0) | 0.0 (0.1) | 0.0 (0.7) | 0.0 (0.4) | 0.0 (0.1) | 0.0 (0.0) | 0.0 (0.0) | 0.0 (0.0) | 0.0 (0.2) | 0.0 (0.0) |
| Lower Fraser River_FA_0.3 | 3.4 (4.1) | 0.0 (0.7) | 0.0 (0.6) | 0.0 (0.9) | 7.8 (3.3) | 3.7 (1.5) | 5.0 (1.9) | 19.8 (5.1) | 0.0 (0.3) | 0.0 (0.8) |
| East Vancouver Island-North_FA_0.x | 0.0 (0.5) | 0.0 (0.1) | 0.0 (0.7) | 0.0 (0.1) | 0.0 (0.1) | 0.0 (0.0) | 0.0 (0.1) | 0.0 (0.0) | 0.0 (0.5) | 0.0 (0.9) |
| East Vancouver Island-Qualicum and Puntledge_FA_0.x | 0.0 (0.3) | 1.7 (1.5) | 0.0 (0.4) | 0.0 (0.6) | 0.0 (0.2) | 0.0 (0.1) | 2.5 (1.3) | 0.0 (0.1) | 0.0 (0.1) | 0.0 (0.0) |
| East Vancouver Island-Nanaimo and Chemainus_FA_0.x | 0.0 (0.0) | 0.0 (0.0) | 0.0 (0.0) | 0.0 (0.0) | 0.0 (0.0) | 0.0 (0.1) | 0.0 (0.0) | 0.0 (0.1) | 0.0 (0.3) | 0.0 (0.0) |
| East Vancouver Island-Nanaimo_SP_1.x | 0.0 (0.0) | 0.0 (0.0) | 0.0 (0.0) | 0.0 (0.0) | 0.0 (0.0) | 0.0 (0.2) | 0.0 (0.0) | 0.0 (0.0) | 0.0 (0.0) | 0.0 (0.0) |
| East Vancouver Island-Georgia Strait_SU_0.3 | 0.0 (0.0) | 0.0 (0.0) | 0.0 (0.0) | 0.0 (0.2) | 0.0 (0.0) | 0.0 (0.0) | 0.0 (0.0) | 0.0 (0.1) | 0.0 (0.0) | 0.0 (0.0) |
| East Vancouver Island-Cowichan and Koksilah_FA_0.x | 10.3 (5.1) | 0.5 (1.6) | 0.0 (0.3) | 0.0 (0.0) | 1.9 (1.7) | 1.0 (0.7) | 1.7 (0.9) | 11.1 (3.2) | 3.4 (3.0) | 0.0 (0.0) |
| West Vancouver Island-Nootka and Kyuquot_FA_0.x | 0.0 (0.0) | 0.0 (0.0) | 0.0 (0.1) | 0.0 (0.0) | 0.0 (0.2) | 0.0 (0.0) | 0.0 (0.0) | 0.0 (0.1) | 0.0 (0.2) | 13.6 (13.7) |
| West Vancouver Island-North_FA_0.x | 0.0 (0.1) | 0.0 (0.1) | 0.0 (0.1) | 0.0 (0.1) | 0.0 (0.2) | 0.0 (0.0) | 0.0 (0.0) | 0.0 (0.0) | 0.0 (0.0) | 0.0 (2.0) |
| West Vancouver Island-South_FA_0.x | 0.0 (0.6) | 0.0 (0.3) | 0.0 (0.2) | 0.0 (0.4) | 2.0 (1.9) | 4.7 (1.4) | 13.1 (2.0) | 24.7 (4.4) | 0.0 (0.1) | 31.9 (16.7) |
| Okanagan_1.x | 0.0 (0.0) | 0.0 (0.1) | 0.0 (0.6) | 0.0 (0.0) | 0.0 (0.1) | 0.0 (0.0) | 0.0 (0.0) | 0.1 (0.5) | 0.0 (0.0) | 0.0 (0.6) |
| Juan de Fuca | 0.0 (1.1) | 0.0 (0.0) | 0.0 (0.1) | 0.0 (0.0) | 0.0 (0.1) | 5.8 (1.4) | 0.8 (0.5) | 0.0 (0.0) | 0.0 (0.3) | 0.0 (0.0) |
| Coastal Washington | 0.0 (0.0) | 0.0 (0.0) | 0.0 (0.0) | 0.0 (0.0) | 0.0 (0.0) | 0.0 (0.0) | 0.6 (0.4) | 0.0 (0.5) | 0.0 (0.0) | 0.0 (0.0) |
| North Puget Sound | 15.2 (7.2) | 16.7 (9.7) | 35.9 (7.4) | 27.9 (6.9) | 11.5 (5.0) | 9.0 (2.7) | 9.4 (2.2) | 11.9 (3.6) | 31.8 (7.0) | 18.8 (11.6) |
| South Puget Sound | 68.5 (8.6) | 76.5 (8.0) | 64.1 (8.5) | 69.9 (7.4) | 39.5 (6.9) | 37.7 (3.5) | 24.6 (2.6) | 20.0 (4.0) | 57.8 (7.9) | 26.6 (11.6) |
| Lower Columbia River | 0.0 (0.2) | 1.6 (2.0) | 0.0 (0.4) | 2.2 (2.1) | 2.8 (2.7) | 2.6 (0.9) | 1.0 (0.7) | 1.4 (1.4) | 0.4 (0.7) | 0.0 (0.1) |
| Mid Columbia River_SP | 2.4 (3.8) | 0.7 (1.1) | 0.0 (0.6) | 0.0 (2.1) | 3.0 (2.6) | 1.2 (1.3) | 0.5 (0.6) | 1.3 (1.5) | 3.1 (3.2) | 0.0 (0.0) |
| Upper Columbia River_SP | 0.1 (0.0) | 0.0 (0.1) | 0.0 (0.0) | 0.0 (0.1) | 0.0 (0.1) | 0.0 (0.0) | 0.0 (0.0) | 0.0 (0.0) | 0.0 (0.0) | 0.0 (0.1) |
| Upper Columbia River_SU_FA | 0.0 (0.1) | 0.0 (0.0) | 0.0 (0.5) | 0.0 (0.5) | 0.0 (0.0) | 1.1 (0.7) | 0.0 (0.1) | 0.7 (1.1) | 0.0 (0.4) | 0.0 (0.0) |
| Snake River_FA | 0.0 (0.0) | 0.0 (0.0) | 0.0 (0.0) | 0.0 (0.0) | 0.0 (0.0) | 0.0 (0.1) | 0.0 (0.0) | 0.5 (1.3) | 0.0 (0.0) | 0.0 (0.0) |
| Snake River_SP_SU | 0.0 (0.9) | 0.0 (0.3) | 0.0 (0.0) | 0.0 (0.0) | 0.0 (0.0) | 0.0 (0.0) | 0.0 (0.0) | 0.0 (0.0) | 0.0 (0.0) | 0.0 (0.2) |
| North & Central Oregon | 0.0 (0.1) | 0.0 (1.6) | 0.0 (0.0) | 0.0 (0.0) | 0.0 (0.0) | 0.5 (0.6) | 0.0 (0.1) | 0.0 (0.0) | 0.0 (0.2) | 0.0 (0.1) |
| Upper Willamette River | 0.0 (0.3) | 2.2 (2.6) | 0.0 (0.2) | 0.0 (0.1) | 2.0 (1.5) | 0.0 (0.0) | 0.0 (0.0) | 0.0 (0.2) | 0.0 (0.2) | 0.0 (0.0) |
| South Oregon coastal | 0.0 (0.0) | 0.0 (0.2) | 0.0 (0.1) | 0.0 (0.0) | 0.0 (0.0) | 0.0 (0.0) | 0.0 (0.0) | 0.0 (0.0) | 0.0 (0.0) | 0.0 (0.9) |
| California Klamath Trinity | 0.0 (0.2) | 0.0 (0.1) | 0.0 (0.2) | 0.0 (0.2) | 0.0 (0.0) | 0.0 (0.0) | 0.0 (0.0) | 0.0 (0.1) | 0.0 (0.0) | 0.0 (0.0) |
| California Central Valley_Fall | 0.0 (0.0) | 0.0 (0.0) | 0.0 (0.0) | 0.0 (0.0) | 0.0 (0.1) | 1.0 (0.8) | 0.3 (0.5) | 0.0 (0.1) | 3.4 (2.2) | 0.0 (0.5) |
| California Central Valley_Spring | 0.0 (0.0) | 0.0 (0.1) | 0.0 (0.0) | 0.0 (0.0) | 0.0 (0.0) | 0.0 (0.0) | 0.0 (0.0) | 0.0 (0.0) | 0.0 (0.0) | 0.0 (0.0) |
| Coastal California | 0.0 (0.3) | 0.0 (0.3) | 0.0 (0.0) | 0.0 (0.2) | 0.0 (0.3) | 0.0 (0.0) | 0.0 (0.0) | 0.0 (0.0) | 0.0 (0.0) | 0.0 (0.0) |

Supplementary Table S4 concluded

| Conservation Unit | WCVI-north sport | | WCVI-south sport | | | | Taaq-wiihak troll | | |
| --- | --- | --- | --- | --- | --- | --- | --- | --- | --- |
|  |  |  |  |  |  |  | NWVI | SWVI | |
|  | July | August | June | July | August | September | July | July | August |
| Sample size | 454 | 321 | 68 | 207 | 308 | 57 | 23 | 8 | 20 |
| N PBT | 26 | 54 | 0 | 4 | 58 | 21 | 0 | 2 | 9 |
| Southeast Alaska | 0.0 (0.0) | 0.0 (0.0) | 0.0 (0.2) | 0.0 (0.0) | 0.0 (0.0) | 0.0 (0.0) | 0.0 (0.3) | 0.0 (1.1) | 0.0 (0.1) |
| Alsek | 0.0 (0.0) | 0.0 (0.1) | 0.0 (0.1) | 0.0 (0.0) | 0.0 (0.0) | 0.0 (0.1) | 0.0 (0.5) | 0.0 (0.1) | 0.0 (0.3) |
| Unuk | 0.0 (0.0) | 0.0 (0.0) | 0.0 (0.0) | 0.0 (0.0) | 0.0 (0.0) | 0.0 (0.0) | 0.0 (0.0) | 0.0 (0.0) | 0.0 (0.0) |
| Taku_early timing | 0.0 (0.0) | 0.0 (0.0) | 0.0 (0.2) | 0.0 (0.0) | 0.0 (0.0) | 0.0 (0.1) | 0.0 (0.4) | 0.0 (0.4) | 0.0 (0.0) |
| Taku_mid timing | 0.0 (0.0) | 0.0 (0.0) | 0.0 (0.0) | 0.0 (0.0) | 0.0 (0.0) | 0.0 (0.1) | 0.0 (0.6) | 0.0 (0.5) | 0.0 (0.0) |
| Taku_late timing | 0.0 (0.0) | 0.0 (0.0) | 0.0 (0.0) | 0.0 (0.0) | 0.0 (0.0) | 0.0 (0.0) | 0.0 (0.0) | 0.0 (0.2) | 0.0 (0.3) |
| Stikine_early timing | 0.0 (0.0) | 0.0 (0.0) | 0.0 (0.1) | 0.0 (0.0) | 0.0 (0.0) | 0.0 (0.1) | 0.0 (0.1) | 0.0 (0.0) | 0.0 (0.2) |
| Stikine_late timing | 0.0 (0.0) | 0.0 (0.0) | 0.0 (0.0) | 0.0 (0.0) | 0.0 (0.0) | 0.0 (0.3) | 0.0 (0.1) | 0.0 (1.7) | 0.0 (0.1) |
| Haida Gwaii-North | 0.0 (0.0) | 0.0 (0.0) | 0.0 (0.0) | 0.0 (0.2) | 0.0 (0.1) | 0.0 (0.0) | 0.0 (0.0) | 0.0 (0.1) | 0.0 (0.0) |
| Upper Nass | 0.0 (0.0) | 0.0 (0.0) | 0.0 (0.1) | 0.0 (0.1) | 0.0 (0.0) | 0.0 (0.5) | 0.0 (0.5) | 0.0 (0.4) | 0.0 (0.5) |
| Portland Sound-Observatory Inlet-Lower Nass | 0.0 (0.0) | 0.0 (0.0) | 0.0 (0.0) | 0.0 (0.1) | 0.0 (0.0) | 0.0 (0.4) | 0.0 (1.0) | 0.0 (0.1) | 0.0 (0.4) |
| Ecstall | 0.0 (0.0) | 0.0 (0.1) | 0.0 (0.0) | 0.0 (0.0) | 0.0 (0.0) | 0.0 (0.0) | 0.0 (0.0) | 0.0 (1.0) | 0.0 (0.0) |
| Skeena Estuary | 0.0 (0.0) | 0.0 (0.0) | 0.0 (0.0) | 0.0 (0.0) | 0.0 (0.0) | 0.0 (0.2) | 0.0 (0.0) | 0.0 (0.0) | 0.0 (0.0) |
| Lower Skeena | 0.0 (0.1) | 0.0 (0.0) | 0.0 (0.2) | 0.0 (0.0) | 0.0 (0.1) | 0.0 (0.2) | 0.0 (0.3) | 0.0 (0.5) | 0.0 (0.4) |
| Kalum_early timing | 0.0 (0.0) | 0.0 (0.0) | 0.0 (0.0) | 0.0 (0.0) | 0.0 (0.0) | 0.0 (0.0) | 0.0 (0.3) | 0.0 (0.3) | 0.0 (0.4) |
| Kalum_late timing | 0.0 (0.0) | 0.0 (0.0) | 0.0 (0.0) | 0.0 (0.0) | 0.0 (0.1) | 0.0 (0.0) | 0.0 (0.0) | 0.0 (0.0) | 0.0 (0.1) |
| Zymoetz | 0.0 (0.0) | 0.0 (0.0) | 0.0 (0.0) | 0.0 (0.0) | 0.0 (0.0) | 0.0 (0.0) | 0.0 (0.0) | 0.0 (1.5) | 0.0 (0.0) |
| Sicintine | 0.0 (0.0) | 0.0 (0.0) | 0.0 (0.0) | 0.0 (0.0) | 0.0 (0.0) | 0.0 (0.0) | 0.0 (0.0) | 0.0 (0.1) | 0.0 (1.2) |
| Middle Skeena-mainstem tributaries | 0.0 (0.1) | 0.0 (0.0) | 0.0 (0.1) | 0.0 (0.1) | 0.0 (0.1) | 0.0 (0.1) | 0.0 (0.4) | 0.0 (0.0) | 0.0 (0.4) |
| Middle Skeena-large lakes | 0.1 (0.2) | 0.0 (0.0) | 0.0 (0.0) | 0.0 (0.0) | 0.0 (0.0) | 0.0 (0.0) | 0.0 (0.1) | 0.0 (1.3) | 0.0 (0.0) |
| Upper Skeena | 0.1 (0.1) | 0.0 (0.0) | 0.0 (0.0) | 0.0 (0.0) | 0.0 (0.0) | 0.0 (0.0) | 0.0 (0.1) | 0.0 (1.6) | 0.0 (0.0) |
| Upper Bulkley River | 0.0 (0.0) | 0.0 (0.0) | 0.0 (0.0) | 0.0 (0.0) | 0.0 (0.0) | 0.0 (0.0) | 0.0 (0.0) | 0.0 (0.7) | 0.0 (0.0) |
| North and Central Coast-late timing | 1.1 (0.5) | 1.9 (0.7) | 0.0 (0.0) | 0.0 (0.0) | 0.0 (0.0) | 0.0 (0.0) | 0.0 (0.0) | 0.0 (0.0) | 0.0 (0.0) |
| North and Central Coast-early timing | 0.0 (0.0) | 0.0 (0.0) | 0.0 (0.0) | 0.0 (0.0) | 0.0 (0.0) | 0.0 (0.4) | 0.0 (0.0) | 0.0 (0.3) | 0.0 (0.0) |
| Rivers Inlet | 0.0 (0.0) | 0.0 (0.0) | 0.0 (0.1) | 0.0 (0.0) | 0.0 (0.0) | 0.0 (0.0) | 0.0 (1.0) | 0.0 (0.6) | 0.0 (0.0) |
| Wannock | 0.0 (0.0) | 0.0 (0.0) | 0.0 (0.0) | 0.0 (0.0) | 0.0 (0.0) | 0.0 (0.0) | 0.0 (0.0) | 0.0 (0.0) | 0.0 (0.0) |
| Bella Coola-Bentinck | 0.0 (0.0) | 0.0 (0.0) | 0.0 (0.1) | 0.0 (0.1) | 0.0 (0.0) | 0.0 (0.2) | 0.0 (0.4) | 0.0 (0.3) | 0.0 (0.0) |
| Dean River | 0.0 (0.0) | 0.0 (0.0) | 0.0 (0.0) | 0.0 (0.0) | 0.0 (0.0) | 0.0 (0.0) | 0.0 (0.1) | 0.0 (2.8) | 0.0 (0.6) |
| Docee | 0.0 (0.0) | 0.0 (0.0) | 0.0 (0.0) | 0.0 (0.0) | 0.0 (0.0) | 0.0 (0.0) | 0.0 (0.0) | 0.0 (0.0) | 0.0 (0.0) |
| Klinaklini_SU_1.3 | 0.0 (0.0) | 0.0 (0.0) | 0.0 (0.0) | 0.0 (0.1) | 0.0 (0.0) | 0.0 (0.0) | 0.0 (0.0) | 0.0 (0.0) | 0.0 (0.1) |
| Southern Mainland-Southern Fjords_FA_0.x | 0.0 (0.0) | 0.0 (0.0) | 0.0 (0.2) | 0.0 (0.0) | 0.0 (0.0) | 0.0 (0.2) | 0.0 (0.1) | 0.0 (0.0) | 0.0 (0.1) |
| Southern Mainland-Georgia Strait_FA_0.x | 0.2 (0.3) | 0.0 (0.0) | 0.0 (0.1) | 0.0 (0.1) | 0.0 (0.0) | 0.0 (0.3) | 0.0 (0.7) | 0.0 (0.1) | 0.0 (0.3) |
| Upper Fraser River_SP_1.3 | 0.0 (0.1) | 0.0 (0.1) | 7.4 (2.7) | 0.5 (0.5) | 0.0 (0.0) | 0.0 (0.1) | 0.0 (0.3) | 0.0 (0.8) | 0.0 (1.6) |
| Middle Fraser River_SU_1.3 | 0.9 (0.4) | 0.0 (0.0) | 0.0 (0.1) | 1.0 (0.8) | 0.3 (0.3) | 0.0 (0.2) | 0.0 (0.1) | 0.0 (0.1) | 0.0 (0.4) |
| Middle Fraser River_SP_1.3 | 0.0 (0.0) | 0.0 (0.0) | 1.5 (1.6) | 0.0 (0.1) | 0.0 (0.0) | 0.0 (0.2) | 0.0 (0.5) | 0.0 (0.3) | 0.0 (0.1) |
| Middle Fraser River-Portage_FA_1.3 | 0.0 (0.0) | 0.0 (0.0) | 0.0 (0.0) | 0.0 (0.1) | 0.0 (0.0) | 0.0 (0.0) | 0.0 (0.0) | 0.0 (0.0) | 0.0 (0.0) |
| Middle Fraser-Fraser Canyon_SP_1.3 | 0.0 (0.0) | 0.0 (0.0) | 0.0 (0.0) | 0.0 (0.0) | 0.0 (0.0) | 0.0 (0.0) | 0.0 (1.0) | 0.0 (0.1) | 0.0 (0.0) |
| North Thompson_SP_1.3 | 0.2 (0.2) | 0.0 (0.0) | 0.0 (0.0) | 1.4 (0.7) | 0.0 (0.0) | 0.0 (0.0) | 0.0 (0.4) | 0.0 (2.5) | 0.0 (0.0) |
| North Thompson_SU_1.3 | 0.2 (0.3) | 0.0 (0.0) | 0.0 (0.2) | 1.5 (0.9) | 0.3 (0.3) | 0.0 (0.2) | 0.0 (1.2) | 0.0 (0.2) | 0.0 (0.1) |
| Shuswap River_SU_0.3 | 2.2 (0.7) | 0.0 (0.0) | 2.9 (1.5) | 12.4 (2.1) | 1.3 (0.8) | 0.0 (0.0) | 4.3 (4.1) | 0.0 (0.0) | 0.0 (0.1) |
| South Thompson-Bessette Creek_SU_1.2 | 0.0 (0.0) | 0.0 (0.0) | 0.0 (0.0) | 0.0 (0.0) | 0.0 (0.0) | 0.0 (0.0) | 0.0 (0.0) | 0.0 (0.0) | 0.0 (0.0) |
| South Thompson_SU_0.3 | 14.6 (1.4) | 10.9 (1.4) | 2.9 (1.8) | 8.9 (1.9) | 21.1 (2.2) | 15.8 (5.2) | 39.1 (9.8) | 0.0 (0.3) | 15.0 (7.5) |
| South Thompson_SU_1.3 | 0.2 (0.2) | 0.0 (0.0) | 0.0 (0.0) | 0.0 (0.0) | 0.0 (0.0) | 0.0 (0.0) | 0.0 (0.0) | 0.0 (0.0) | 0.0 (0.0) |
| Lower Thompson_SP_1.2 | 0.0 (0.0) | 0.0 (0.0) | 0.0 (0.3) | 0.0 (0.0) | 0.0 (0.0) | 0.0 (0.1) | 0.0 (0.9) | 0.0 (2.0) | 0.0 (2.2) |
| Lower Fraser River_SP_1.3 | 0.0 (0.0) | 0.0 (0.0) | 0.0 (0.0) | 0.0 (0.0) | 0.0 (0.0) | 0.0 (0.0) | 0.0 (0.0) | 0.0 (1.2) | 0.0 (0.2) |
| Lower Fraser River_SU_1.3 | 0.0 (0.0) | 0.0 (0.0) | 0.0 (0.0) | 0.0 (0.0) | 0.0 (0.0) | 0.0 (0.2) | 0.0 (0.5) | 0.0 (0.4) | 0.0 (0.0) |
| Lower Fraser River-Upper Pitt_SU_1.3 | 0.0 (0.0) | 0.0 (0.0) | 1.5 (1.2) | 0.0 (0.1) | 0.0 (0.0) | 0.0 (0.0) | 0.0 (0.1) | 0.0 (0.1) | 0.0 (1.1) |
| Maria Slough_SU_0.3 | 0.2 (0.3) | 0.0 (0.1) | 0.0 (0.0) | 0.0 (0.0) | 0.0 (0.0) | 0.0 (0.0) | 0.0 (0.0) | 0.0 (0.0) | 0.0 (0.0) |
| Lower Fraser River_FA_0.3 | 2.2 (0.7) | 1.6 (0.7) | 2.9 (1.7) | 4.4 (1.2) | 3.6 (1.1) | 2.0 (2.1) | 4.3 (3.5) | 0.0 (0.7) | 0.0 (0.8) |
| East Vancouver Island-North_FA_0.x | 0.0 (0.0) | 0.0 (0.0) | 0.0 (0.0) | 0.0 (0.1) | 0.0 (0.0) | 0.0 (0.1) | 0.0 (0.4) | 0.0 (0.1) | 0.0 (0.3) |
| East Vancouver Island-Qualicum and Puntledge_FA_0.x | 2.0 (0.6) | 1.7 (0.7) | 0.0 (0.0) | 0.0 (0.0) | 1.8 (0.9) | 1.8 (2.2) | 0.0 (0.1) | 0.0 (0.0) | 0.3 (0.1) |
| East Vancouver Island-Nanaimo and Chemainus_FA_0.x | 0.0 (0.0) | 0.0 (0.0) | 0.0 (0.0) | 0.0 (0.0) | 0.0 (0.0) | 0.0 (0.0) | 0.0 (0.1) | 0.0 (0.3) | 0.0 (0.0) |
| East Vancouver Island-Nanaimo_SP_1.x | 0.0 (0.0) | 0.0 (0.0) | 0.0 (0.0) | 0.0 (0.0) | 0.0 (0.0) | 0.0 (0.0) | 0.0 (0.0) | 0.0 (0.0) | 0.0 (0.1) |
| East Vancouver Island-Georgia Strait_SU_0.3 | 0.0 (0.0) | 0.0 (0.0) | 0.0 (0.0) | 0.5 (0.5) | 0.0 (0.0) | 0.0 (0.0) | 0.0 (0.4) | 0.0 (0.0) | 0.0 (0.0) |
| East Vancouver Island-Cowichan and Koksilah_FA_0.x | 0.8 (0.4) | 1.8 (0.7) | 0.0 (0.0) | 1.9 (1.0) | 1.4 (0.9) | 0.0 (0.0) | 0.0 (0.0) | 0.0 (0.1) | 4.7 (3.5) |
| West Vancouver Island-Nootka and Kyuquot_FA_0.x | 14.3 (1.7) | 14.1 (2.3) | 0.0 (0.3) | 2.3 (1.1) | 0.6 (0.4) | 0.0 (0.1) | 0.0 (0.3) | 10.9 (12.3) | 2.1 (5.3) |
| West Vancouver Island-North_FA_0.x | 0.0 (0.0) | 0.0 (0.0) | 0.0 (0.0) | 0.0 (0.0) | 0.0 (0.0) | 0.0 (0.2) | 0.0 (0.4) | 0.0 (0.4) | 0.0 (0.5) |
| West Vancouver Island-South_FA_0.x | 15.2 (1.9) | 39.7 (3.3) | 1.5 (1.4) | 12.7 (2.2) | 46.2 (2.4) | 80.4 (6.3) | 0.0 (0.1) | 39.1 (13.6) | 67.9 (10.0) |
| Okanagan_1.x | 0.1 (0.2) | 0.0 (0.0) | 0.0 (0.0) | 1.0 (0.7) | 0.0 (0.0) | 0.0 (0.0) | 0.0 (0.0) | 0.0 (0.7) | 0.0 (0.0) |
| Juan de Fuca | 0.7 (0.3) | 0.0 (0.0) | 7.4 (3.2) | 1.4 (0.9) | 0.3 (0.3) | 0.0 (0.0) | 0.0 (0.0) | 0.0 (1.3) | 5.0 (5.1) |
| Coastal Washington | 2.0 (0.7) | 4.4 (1.0) | 0.0 (0.0) | 0.5 (0.6) | 1.9 (0.8) | 0.0 (0.1) | 4.3 (5.2) | 0.0 (0.0) | 0.0 (0.2) |
| North Puget Sound | 4.7 (1.5) | 1.5 (0.4) | 9.3 (4.7) | 9.7 (1.7) | 2.5 (1.1) | 0.0 (0.0) | 0.1 (0.0) | 0.9 (6.4) | 5.0 (3.8) |
| South Puget Sound | 9.5 (1.4) | 7.3 (1.2) | 41.7 (6.2) | 23.5 (2.8) | 11.1 (1.8) | 0.0 (0.0) | 8.6 (5.4) | 17.5 (9.2) | 0.0 (0.3) |
| Lower Columbia River | 7.2 (1.0) | 2.6 (1.1) | 7.4 (3.0) | 9.6 (2.2) | 2.8 (0.9) | 0.0 (0.0) | 4.0 (3.4) | 0.0 (0.1) | 0.0 (0.0) |
| Mid Columbia River_SP | 0.1 (0.5) | 0.0 (0.0) | 0.4 (1.3) | 0.1 (0.4) | 1.8 (0.7) | 0.0 (0.0) | 0.4 (0.7) | 6.6 (14.0) | 0.1 (0.3) |
| Upper Columbia River_SP | 0.0 (0.1) | 0.0 (0.0) | 0.0 (0.0) | 0.0 (0.0) | 0.0 (0.0) | 0.0 (1.0) | 0.0 (0.1) | 0.0 (0.5) | 0.0 (0.0) |
| Upper Columbia River_SU_FA | 7.2 (1.3) | 5.2 (1.4) | 3.0 (1.8) | 0.6 (0.6) | 2.3 (1.0) | 0.0 (0.0) | 17.4 (8.1) | 25.0 (13.4) | 0.0 (0.9) |
| Snake River_FA | 3.9 (1.0) | 2.0 (0.9) | 1.4 (2.2) | 4.7 (1.6) | 0.0 (0.0) | 0.0 (0.0) | 0.0 (0.0) | 0.0 (0.1) | 0.0 (0.3) |
| Snake River_SP_SU | 0.0 (0.1) | 0.0 (0.0) | 0.0 (0.2) | 0.0 (0.1) | 0.0 (0.0) | 0.0 (0.6) | 0.0 (0.5) | 0.0 (0.7) | 0.0 (1.3) |
| North & Central Oregon | 5.0 (1.1) | 2.2 (0.7) | 0.0 (0.1) | 0.0 (0.0) | 0.0 (0.0) | 0.0 (0.1) | 13.1 (8.1) | 0.0 (1.5) | 0.0 (0.4) |
| Upper Willamette River | 0.0 (0.0) | 0.0 (0.0) | 1.5 (1.6) | 0.0 (0.0) | 0.0 (0.0) | 0.0 (0.1) | 0.0 (0.0) | 0.0 (0.0) | 0.0 (0.0) |
| South Oregon coastal | 4.4 (1.0) | 3.2 (1.0) | 2.5 (1.9) | 0.5 (0.5) | 0.3 (0.3) | 0.0 (0.2) | 4.3 (4.2) | 0.0 (4.4 | 0.0 (0.1) |
| California Klamath Trinity | 0.0 (0.0) | 0.0 (0.0) | 0.4 (1.6) | 0.0 (0.1) | 0.0 (0.0) | 0.0 (0.0) | 0.0 (0.1) | 0.0 (0.4) | 0.0 (0.0) |
| California Central Valley_Fall | 0.4 (0.3) | 0.0 (0.0) | 4.4 (2.1) | 1.0 (0.6) | 0.2 (0.4) | 0.0 (0.2) | 0.0 (1.7) | 0.0 (1.1) | 0.0 (0.9) |
| California Central Valley_Spring | 0.0 (0.1) | 0.0 (0.0) | 0.0 (0.0) | 0.0 (0.0) | 0.1 (0.3) | 0.0 (0.0) | 0.0 (0.0) | 0.0 (0.1) | 0.0 (0.0) |
| Coastal California | 0.0 (0.0) | 0.0 (0.0) | 0.0 (0.0) | 0.0 (0.0) | 0.0 (0.0) | 0.0 (0.0) | 0.0 (0.0) | 0.0 (0.2) | 0.0 (1.5) |

Supplementary Table S5. Estimated percentage stock compositions by geographic region (US populations) or Conservation Unit (Canadian populations) for Chinook salmon fisheries sampled in British Columbia during 2019. N is number of individuals genotyped, and N PBT is the number of individuals in the sample subsequently identified via PBT. Standard deviation is in parentheses.

| Conservation Unit | Area F troll | | Area F freezer | Area 1 sport | | | | | Area 3 sport | | |
| --- | --- | --- | --- | --- | --- | --- | --- | --- | --- | --- | --- |
|  | Aug 24-Sept 03 | Sept 04-24 | Aug 20-Sept23 | May | June | July | Aug | Sept | June | July | August |
| Sample size | 298 | 261 | 102 | 33 | 124 | 126 | 149 | 50 | 90 | 58 | 18 |
| N PBT | 19 | 6 | 8 | 6 | 10 | 26 | 36 | 6 | 6 | 5 | 6 |
| Southeast Alaska | 0.0 (0.1) | 0.0 (0.1) | 0.0 (0.1) | 0.0 (0.1) | 0.0 (0.1) | 0.0 (0.1) | 0.0 (0.1) | 0.0 (0.0) | 0.0 (0.0) | 0.0 (0.0) | 0.0 (0.1) |
| Alsek | 0.0 (0.0) | 0.4 (0.4) | 0.0 (0.4) | 0.0 (0.1) | 0.0 (0.0) | 0.0 (0.0) | 0.0 (0.0) | 0.0 (0.0) | 0.0 (0.0) | 0.0 (0.1) | 0.0 (0.0) |
| Unuk | 0.0 (0.1) | 0.0 (0.0) | 0.0 (0.0) | 3.2 (3.8) | 0.4 (1.2) | 0.0 (0.1) | 0.0 (0.0) | 0.0 (0.0) | 5.1 (2.7) | 0.0 (0.1) | 0.0 (0.0) |
| Taku_early timing | 0.0 (0.0) | 0.0 (0.0) | 0.0 (0.6) | 0.0 (0.4) | 0.0 (0.1) | 0.0 (0.0) | 0.0 (0.0) | 0.0 (0.3) | 0.0 (0.0) | 0.0 (0.0) | 0.0 (1.6) |
| Taku_mid timing | 0.0 (0.0) | 0.0 (0.0) | 0.0 (0.2) | 0.0 (0.2) | 0.0 (0.1) | 0.0 (0.0) | 0.0 (0.0) | 0.0 (0.0) | 0.0 (0.0) | 0.0 (0.0) | 0.0 (0.0) |
| Taku_late timing | 0.0 (0.0) | 0.0 (0.0) | 0.0 (0.0) | 0.0 (0.0) | 0.0 (0.0) | 0.0 (0.0) | 0.0 (0.0) | 0.0 (0.0) | 0.0 (0.0) | 0.0 (0.1) | 0.0 (0.0) |
| Stikine_early timing | 0.0 (0.1) | 0.0 (0.0) | 0.0 (0.1) | 0.5 (1.2) | 0.0 (0.1) | 0.0 (0.0) | 0.0 (0.0) | 0.0 (0.0) | 0.3 (0.3) | 0.0 (0.2) | 0.0 (0.0) |
| Stikine_late timing | 0.0 (0.0) | 0.0 (0.0) | 0.0 (0.0) | 1.5 (3.5) | 0.0 (0.2) | 0.0 (0.1) | 0.0 (0.0) | 0.0 (0.0) | 0.5 (0.7) | 0.0 (0.1) | 0.0 (0.0) |
| Haida Gwaii-North | 0.3 (0.4) | 0.0 (0.0) | 0.0 (0.0) | 0.0 (0.0) | 0.0 (0.0) | 0.0 (0.0) | 0.7 (0.9) | 0.0 (0.0) | 0.0 (0.0) | 0.0 (0.1) | 0.0 (0.0) |
| Upper Nass | 0.0 (0.2) | 0.0 (0.0) | 0.0 (0.0) | 0.0 (0.6) | 1.6 (1.1) | 0.0 (0.0) | 0.0 (0.0) | 0.0 (0.1) | 20.1 (4.3) | 1.0 (1.2) | 16.7 (8.0) |
| Portland Sound-Observatory Inlet-Lower Nass | 0.0 (0.0) | 0.0 (0.0) | 0.0 (0.1) | 0.0 (0.0) | 4.4 (2.2) | 0.0 (0.0) | 0.0 (0.0) | 0.0 (0.0) | 12.8 (4.7) | 5.6 (3.3) | 0.0 (0.6) |
| Ecstall | 0.0 (0.1) | 0.0 (0.0) | 0.0 (0.0) | 0.0 (0.1) | 0.8 (0.9) | 0.0 (0.0) | 0.0 (0.0) | 0.0 (0.0) | 3.3 (1.7) | 6.9 (2.8) | 0.0 (0.0) |
| Skeena Estuary | 0.0 (0.0) | 0.0 (0.0) | 0.0 (0.0) | 0.0 (0.0) | 0.0 ( 0.0) | 0.0 (0.0) | 0.0 (0.0) | 0.0 (0.0) | 1.1 (1.2) | 10.3 (4.8) | 5.6 (3.6) |
| Lower Skeena | 0.0 (0.1) | 0.0 (0.0) | 0.0 (0.1) | 0.2 (1.8) | 1.5 (1.4) | 0.0 (0.0) | 0.0 (0.0) | 0.0 (0.2) | 2.7 (2.0) | 0.0 (0.0) | 0.0 (0.2) |
| Kalum_early timing | 0.0 (0.0) | 0.0 (0.0) | 0.0 (0.0) | 3.0 (2.1) | 0.0 (0.0) | 0.0 (0.0) | 0.0 (0.0) | 0.0 (0.0) | 0.0 (0.0) | 0.0 (0.0) | 0.0 (0.0) |
| Kalum_late timing | 0.0 (0.0) | 0.0 (0.0) | 0.0 (0.0) | 0.0 (0.0) | 4.7 (2.0) | 4.7 (1.6) | 0.0 (0.0) | 0.0 (0.0) | 6.4 (3.0) | 29.6 (6.0) | 13.5 (6.4) |
| Zymoetz | 0.0 (0.0) | 0.0 (0.0) | 0.0 (0.0) | 0.0 (0.0) | 1.8 (1.2) | 0.0 (0.0) | 0.0 (0.0) | 0.0 (0.0) | 4.6 (2.5) | 0.0 (0.0) | 0.0 (0.0) |
| Sicintine | 0.0 (0.0) | 0.0 (0.0) | 0.0 (0.0) | 0.0 (0.0) | 0.0 (0.0) | 0.0 (0.0) | 0.0 (0.0) | 0.0 (0.0) | 0.0 (0.0) | 0.0 (0.0) | 0.0 (0.0) |
| Middle Skeena-mainstem tributaries | 0.0 (0.0) | 0.0 (0.0) | 0.0 (0.3) | 0.6 (1.7) | 4.0 (1.6) | 0.8 (0.6) | 0.0 (0.1) | 0.0 (0.3) | 10.4 (3.5) | 0.0 (0.0) | 0.0 (0.3) |
| Middle Skeena-large lakes | 0.0 (0.0) | 0.0 (0.0) | 0.0 (0.2) | 6.8 (5.6) | 0.7 (1.0) | 0.0 (0.0) | 0.0 (0.0) | 0.0 (0.0) | 3.0 (2.3) | 7.8 (3.0) | 3.1 (3.7) |
| Upper Skeena | 0.0 (0.0) | 0.0 (0.0) | 0.0 (0.0) | 4.1 (3.7) | 2.5 (2.1) | 0.0 (0.0) | 0.0 (0.1) | 0.0 (0.5) | 3.2 (2.7) | 2.5 (2.4) | 0.0 (0.7) |
| Upper Bulkley River | 0.0 (0.0) | 0.0 (0.0) | 0.0 (0.0) | 0.0 (0.0) | 0.0 (0.0) | 0.0 (0.0) | 0.0 (0.0) | 0.0 (0.0) | 0.0 (0.2) | 0.0 (0.1) | 0.0 (0.7) |
| North and Central Coast-late timing | 0.7 (0.4) | 0.0 (0.0) | 1.0 (1.1) | 0.0 (0.2) | 0.8 (0.7) | 0.0 (0.0) | 0.7 (0.6) | 0.0 (0.0) | 4.8 (2.6) | 1.4 (1.6) | 0.0 (0.2) |
| North and Central Coast-early timing | 0.0 (0.0) | 0.0 (0.1) | 0.0 (0.1) | 0.0 (0.2) | 1.1 (1.6) | 0.0 (0.0) | 0.0 (0.0) | 0.0 (0.1) | 2.2 (1.5) | 0.2 (1.6) | 0.0 (0.1) |
| Rivers Inlet | 0.0 (0.0) | 0.0 (0.0) | 0.0 (0.1) | 0.0 (0.3) | 2.3 (1.4) | 0.1 (0.5) | 0.0 (0.0) | 0.0 (0.2) | 0.1 (0.0) | 0.0 (0.1) | 0.0 (0.0) |
| Wannock | 0.0 (0.0) | 0.0 (0.0) | 0.0 (0.1) | 0.0 (0.0) | 0.0 (0.0) | 2.4 (1.3) | 4.0 (1.5) | 0.0 (0.0) | 0.0 (0.0) | 5.2 (2.3) | 5.6 (4.2) |
| Bella Coola-Bentinck | 0.0 (0.1) | 0.0 (0.1) | 0.0 (0.2) | 3.0 (3.2) | 5.7 (1.9) | 3.9 (1.9) | 0.0 (0.1) | 0.0 (0.0) | 2.6 (1.8) | 0.1 (0.2) | 0.0 (0.0) |
| Dean River | 0.0 (0.0) | 0.0 (0.0) | 0.0 (0.0) | 1.2 (2.2) | 0.8 (0.7) | 0.0 (0.0) | 0.0 (0.1) | 0.0 (0.1) | 0.0 (0.0) | 0.0 (0.2) | 0.0 (0.1) |
| Docee | 0.3 (0.4) | 0.0 (0.0) | 0.0 (0.1) | 0.0 (0.0) | 0.0 (0.0) | 0.0 (0.0) | 1.3 (0.9) | 0.0 (0.0) | 0.0 (0.1) | 0.0 (0.0) | 0.0 (0.0) |
| Klinaklini_SU_1.3 | 0.0 (0.0) | 0.0 (0.0) | 0.0 (0.0) | 3.0 (3.6) | 1.5 (1.1) | 0.7 (0.6) | 0.0 (0.0) | 0.0 (0.0) | 2.2 (1.3) | 1.7 (1.6) | 0.0 (0.2) |
| Southern Mainland-Southern Fjords_FA_0.x | 0.0 (0.0) | 0.0 (0.0) | 0.0 (0.1) | 0.0 (0.0) | 0.0 (0.0) | 0.0 (0.0) | 0.0 (0.0) | 0.0 (0.0) | 0.0 (0.0) | 1.7 (1.6) | 0.0 (0.0) |
| Southern Mainland-Georgia Strait_FA_0.x | 0.0 (0.0) | 0.0 (0.0) | 0.0 (0.4) | 0.0 (0.5) | 0.0 (0.2) | 0.1 (0.3) | 0.0 (0.1) | 0.0 (0.2) | 2.2 (1.7) | 0.0 (0.1) | 0.0 (4.0) |
| Upper Fraser River_SP_1.3 | 0.0 (0.0) | 0.1 (0.3) | 0.0 (0.1) | 0.0 (1.2) | 0.0 (0.0) | 0.6 (0.9) | 0.0 (0.2) | 0.0 (0.7) | 0.0 (0.5) | 0.0 (0.5) | 0.0 (0.1) |
| Middle Fraser River_SU_1.3 | 0.0 (0.0) | 0.6 (0.6) | 0.0 (0.0) | 0.0 (0.7) | 0.0 (0.0) | 0.6 (0.6) | 0.0 (0.1) | 0.0 (0.7) | 0.0 (0.2) | 0.0 (0.2) | 0.0 (0.5) |
| Middle Fraser River_SP_1.3 | 0.0 (0.1) | 0.1 (0.2) | 0.0 (0.3) | 0.0 (0.5) | 0.0 (0.0) | 0.4 (0.9) | 0.0 (0.0) | 0.0 (0.4) | 0.0 (0.2) | 0.0 (0.1) | 0.0 (1.1) |
| Middle Fraser River-Portage_FA_1.3 | 0.0 (0.0) | 0.8 (0.5) | 0.0 (0.0) | 0.0 (0.0) | 0.0 (0.0) | 0.0 (0.0) | 0.0 (0.0) | 0.0 (0.0) | 0.0 (0.0) | 0.0 (0.0) | 0.0 (0.1) |
| Middle Fraser-Fraser Canyon_SP_1.3 | 0.0 (0.0) | 0.0 (0.0) | 0.0 (0.1) | 0.0 (0.0) | 0.0 (0.0) | 0.0 (0.0) | 0.0 (0.0) | 0.0 (0.0) | 0.0 (0.0) | 0.0 (0.0) | 0.0 (0.0) |
| North Thompson_SP_1.3 | 0.0 (0.0) | 0.0 (0.0) | 0.0 (0.0) | 0.0 (0.0) | 0.0 (0.0) | 0.0 (0.0) | 0.0 (0.0) | 0.0 (0.0) | 0.0 (0.0) | 0.0 (0.0) | 0.0 (0.1) |
| North Thompson_SU_1.3 | 0.0 (0.0) | 0.0 (0.0) | 0.0 (0.0) | 3.0 (3.8) | 0.8 (0.7) | 0.0 (0.1) | 0.0 (0.0) | 0.0 (0.1) | 0.0 (0.0) | 0.0 (0.0) | 0.0 (0.1) |
| Shuswap River_SU_0.3 | 0.0 (0.1) | 0.0 (0.0) | 0.0 (0.0) | 2.9 (2.9) | 3.9 (1.9) | 4.1 (1.8) | 1.3 (0.8) | 0.0 (0.0) | 0.0 (0.0) | 0.0 (0.0) | 0.0 (0.0) |
| South Thompson-Bessette Creek_SU_1.2 | 0.0 (0.0) | 0.0 (0.0) | 0.0 (0.0) | 0.0 (0.0) | 0.2 (0.3) | 0.0 (0.0) | 0.0 (0.0) | 0.0 (0.0) | 0.0 (0.0) | 0.0 (0.0) | 0.0 (0.5) |
| South Thompson_SU_0.3 | 12.7 (2.3) | 3.8 (1.0) | 7.8 (1.9) | 15.2 (5.4) | 26.6 (3.9) | 24.5 (4.6) | 9.4 (2.7) | 4.0 (2.9) | 2.2 (1.5) | 1.7 (1.6) | 0.0 (0.0) |
| South Thompson_SU_1.3 | 0.0 (0.0) | 0.0 (0.0) | 0.0 (0.0) | 0.0 (0.1) | 0.0 (0.0() | 0.0 (0.0) | 0.0 (0.0) | 0.0 (0.0) | 0.0 (0.1) | 0.0 (0.1) | 0.0 (0.1) |
| Lower Thompson_SP_1.2 | 0.0 (0.0) | 0.0 (0.0) | 0.0 (0.1) | 0.0 (0.5) | 0.0 (0.0) | 0.0 (0.0) | 0.0 (0.1) | 0.0 (0.1) | 0.0 (0.1) | 0.0 (0.0) | 0.0 (0.1) |
| Lower Fraser River_SP_1.3 | 0.3 (0.4) | 0.0 (0.0) | 0.0 (0.0) | 0.0 (0.0) | 0.0 (0.0) | 0.0 (0.0) | 0.0 (0.0) | 0.0 (0.0) | 0.0 (0.0) | 0.0 (0.0) | 0.0 (0.0) |
| Lower Fraser River_SU_1.3 | 0.0 (0.0) | 0.0 (0.0) | 0.0 (0.0) | 0.0 (0.0) | 0.0 (0.0) | 0.0 (0.0) | 0.7 (0.6) | 0.0 (0.0) | 0.0 (0.0) | 0.0 (0.0) | 0.0 (0.0) |
| Lower Fraser River-Upper Pitt_SU_1.3 | 0.0 (0.0) | 0.0 (0.0) | 0.0 (0.0) | 0.0 (0.0) | 0.0 (0.1) | 0.0 (0.1) | 0.0 (0.0) | 0.0 (0.0) | 0.0 (0.0) | 0.0 (0.0) | 0.0 (0.0) |
| Maria Slough_SU_0.3 | 0.0 (0.0) | 0.0 (0.0) | 0.0 (0.0) | 0.0 (0.0) | 0.0 (0.0) | 0.0 (0.0) | 0.0 (0.0) | 0.0 (0.0) | 0.0 (0.0) | 0.0 (0.0) | 0.0 (0.0) |
| Lower Fraser River_FA_0.3 | 0.0 (0.1) | 0.0 (0.0) | 0.0 (0.0) | 0.0 (0.2) | 0.0 (0.1) | 0.8 (0.8) | 0.7 (0.6) | 0.0 (0.1) | 0.0 (0.0) | 0.0 (0.0) | 0.0 (0.7) |
| East Vancouver Island-North_FA_0.x | 0.0 (0.0) | 0.0 (0.0) | 0.0 (0.1) | 0.0 (0.1) | 0.0 (0.0) | 1.6 (1.3) | 1.3 (1.1) | 0.0 (0.0) | 2.2 (2.2) | 8.6 (3.2) | 27.8 (10.2) |
| East Vancouver Island-Qualicum and Puntledge_FA_0.x | 1.4 (0.7) | 0.0 (0.0) | 0.0 (0.0) | 0.0 (0.1) | 0.7 (0.6) | 0.0 (0.0) | 0.0 (0.2) | 0.0 (0.2) | 0.2 (0.2) | 0.0 (0.1) | 5.5 (5.9) |
| East Vancouver Island-Nanaimo and Chemainus_FA_0.x | 0.0 (0.0) | 0.0 (0.0) | 0.0 (0.0) | 0.0 (0.0) | 0.1 (0.5) | 0.0 (0.0) | 0.0 (0.0) | 0.0 (0.1) | 0.0 (0.0) | 0.0 (0.0) | 0.0 (0.0) |
| East Vancouver Island-Nanaimo_SP_1.x | 0.0 (0.0) | 0.0 (0.0) | 0.0 (0.0) | 0.0 (0.0) | 0.0 (0.0) | 0.0 (0.0) | 0.0 (0.0) | 0.0 (0.0) | 0.0 (0.0) | 0.0 (0.0) | 0.0 (0.0) |
| East Vancouver Island-Georgia Strait_SU_0.3 | 0.0 (0.0) | 0.0 (0.1) | 0.0 (0.3) | 0.0 (0.0) | 0.0 (0.0) | 0.0 (0.0) | 0.0 (0.0) | 0.0 (0.1) | 0.0 (0.1) | 0.0 (0.0) | 0.0 (0.3) |
| East Vancouver Island-Cowichan and Koksilah_FA_0.x | 0.0 (0.0) | 0.4 (0.4) | 0.0 (0.0) | 0.0 (0.0) | 0.0 (0.2) | 0.0 (0.0) | 0.0 (0.0) | 0.0 (0.0) | 2.0 (1.0) | 0.0 (0.0) | 0.0 (0.0) |
| West Vancouver Island-Nootka and Kyuquot_FA_0.x | 0.0 (0.0) | 0.2 (0.2) | 0.0 (0.0) | 24.2 (7.3) | 4.8 (2.0) | 6.3 (2.4) | 0.7 (0.4) | 0.0 (0.1) | 1.1 (1.1) | 0.0 (0.1) | 0.0 (0.5) |
| West Vancouver Island-North_FA_0.x | 0.0 (0.0) | 0.0 (0.0) | 0.0 (0.1) | 0.0 (0.0) | 0.0 (0.1) | 0.0 (0.0) | 0.0 (0.0) | 0.0 (0.0) | 0.0 (0.0) | 0.0 (0.0) | 0.0 (0.1) |
| West Vancouver Island-South_FA_0.x | 9.4 (1.7) | 2.9 (1.0) | 16.7 (3.6) | 15.2 (5.0) | 8.6 (3.0) | 23.1 (4.4) | 36.9 (4.4) | 18.0 (5.7) | 3.3 (1.6) | 12.1 (4.4) | 11.1 (7.1) |
| Okanagan_1.x | 0.1 (0.3) | 0.1 (0.4) | 0.1 (0.6) | 0.0 (0.0) | 0.6 (1.5) | 0.0 (0.0) | 0.1 (0.4) | 0.0 (0.5) | 0.0 (0.0) | 0.1 (0.4) | 0.0 (0.3) |
| Juan de Fuca | 0.0 (0.0) | 0.0 (0.0) | 0.0 (0.0) | 0.0 (0.0) | 0.8 (1.0) | 0.0 (0.0) | 0.0 (0.0) | 0.0 (0.0) | 0.0 (0.0) | 0.0 (0.0) | 0.0 (0.0) |
| Coastal Washington | 18.6 (2.4) | 25.0 (2.7) | 31.8 (4.6) | 0.0 (0.5) | 3.2 (1.7) | 4.7 (1.5) | 10.9 (2.5) | 19.4 (5.8) | 1.1 (1.4) | 0.0 (0.1) | 5.6 (4.6) |
| North Puget Sound | 0.0 (0.0) | 0.4 (0.3) | 0.0 (0.0) | 0.0 (0.1) | 0.8 (0.9) | 0.0 (0.0) | 0.0 (0.0) | 0.0 (0.1) | 0.0 (0.0) | 0.0 (0.2) | 0.0 (0.2) |
| South Puget Sound | 0.0 (0.0) | 0.0 (0.0) | 0.0 (0.1) | 0.0 (0.4) | 0.0 (0.0) | 0.0 (0.0) | 0.0 (0.0) | 0.0 (0.0) | 0.0 (0.0) | 0.0 (0.0) | 0.0 (0.3) |
| Lower Columbia River | 2.7 (1.1) | 4.2 (1.4) | 2.0 (1.2) | 0.0 (0.4) | 1.6 (1.0) | 1.3 (1.1) | 1.3 (1.0) | 4.0 (2.4) | 0.0 (0.0) | 0.0 (0.0) | 0.0 (0.0) |
| Mid Columbia River_SP | 0.0 (0.0) | 0.0 (0.0) | 0.0 (0.0) | 0.0 (0.0) | 0.0 (0.1) | 0.2 (0.8) | 0.0 (0.1) | 0.0 (0.0) | 0.0 (0.1) | 0.0 (0.1) | 0.0 (0.4) |
| Upper Columbia River_SP | 0.0 (0.0) | 0.0 (0.0) | 0.0 (0.0) | 0.0 (0.1) | 0.0 (0.0) | 0.0 (0.0) | 0.0 (0.0) | 0.0 (0.0) | 0.0 (0.1) | 0.0 (0.1) | 0.0 (0.0) |
| Upper Columbia River_SU_FA | 26.6 (2.8) | 22.2 (2.7) | 16.5 (3.8) | 9.1 (5.4) | 7.4 (2.8) | 13.7 (3.2) | 14.5 (3.3) | 20.4 (5.9) | 0.0 (0.0) | 3.4 (2.1) | 0.0 (0.2) |
| Snake River_FA | 7.1 (1.3) | 3.8 (1.6) | 0.1 (0.8) | 0.0 (1.1) | 3.3 (1.9) | 1.3 (1.2) | 2.9 (1.8) | 5.6 (4.7) | 0.0 (0.0) | 0.0 (0.0) | 0.0 (0.0) |
| Snake River_SP_SU | 0.3 (0.3) | 0.4 (0.3) | 0.0 (0.0) | 0.0 (0.2) | 0.0 (0.1) | 0.0 (0.1) | 0.0 (0.1) | 0.0 (0.4) | 0.0 (0.2) | 0.0 (0.1) | 0.0 (1.5) |
| North & Central Oregon | 12.9 (1.8) | 21.6 (2.7) | 17.5 (4.2) | 1.8 (2.5) | 1.8 (1.4) | 1.1 (0.8) | 4.9 (2.0) | 26.8 (6.8) | 0.0 (0.2) | 0.0 (0.1) | 3.6 (4.4) |
| Upper Willamette River | 0.6 (0.4) | 0.0 (0.0) | 0.0 (0.0) | 0.0 (0.2) | 0.0 (0.0) | 1.6 (1.0) | 0.0 (0.0) | 0.0 (0.0) | 0.0 (0.0) | 0.0 (0.4) | 0.0 (0.2) |
| South Oregon coastal | 5.5 (1.5) | 12.8 (2.3) | 6.6 (2.7) | 1.2 (2.5) | 0.1 (0.5) | 1.3 (1.0) | 7.7 (2.0) | 1.8 (2.8) | 0.0 (0.0) | 0.0 (0.3) | 2.0 (4.0) |
| California Klamath Trinity | 0.0 (0.1) | 0.0 (0.0) | 0.0 (0.2) | 0.0 (0.0) | 0.0 (0.2) | 0.0 (0.0) | 0.0 (0.0) | 0.0 (0.0) | 0.0 (0.2) | 0.0 (0.0) | 0.0 (0.1) |
| California Central Valley_Fall | 0.0 (0.0) | 0.0 (0.0) | 0.0 (0.0) | 0.0 (0.3) | 0.0 (0.1) | 0.0 (0.0) | 0.0 (0.1) | 0.0 (0.1) | 0.0 (0.1) | 0.0 (0.4) | 0.0 (0.0) |
| California Central Valley_Spring | 0.0 (0.0) | 0.0 (0.0) | 0.0 (0.0) | 0.0 (0.1) | 0.0 (0.1) | 0.0 (0.1) | 0.0 (0.0) | 0.0 (0.0) | 0.0 (0.0) | 0.0 (0.1) | 0.0 (0.1) |
| Coastal California | 0.0 (0.0) | 0.0 (0.0) | 0.0 (0.0) | 0.0 (0.0) | 0.0 (0.0) | 0.0 (0.0) | 0.0 (0.0) | 0.0 (0.1) | 0.0 (0.0) | 0.0 (0.0) | 0.0 (0.0) |

Supplementary Table S5 continued

| Conservation Unit | Area 3/4 sport | | | | Tyee test | Bella Bella FSC | Area 8 Gillnet | | Area 9 sport | | |
| --- | --- | --- | --- | --- | --- | --- | --- | --- | --- | --- | --- |
|  | May | June | July | August | Seasonal | June | July-August | | June | July | August |
| Sample size | 66 | 164 | 106 | 34 | 422 | 116 | 180 | | 97 | 78 | 18 |
| N PBT | 21 | 35 | 20 | 5 | 11 | 40 | 46 | | 35 | 26 | 9 |
| Southeast Alaska | 0.0 (0.7) | 0.0 (0.1) | 0.0 (0.1) | 0.0 (0.0) | 0.0 (0.0) | 0.0 (0.2) | 0.0 (0.0) | | 0.0 (0.0) | 0.0 (0.2) | 0.0 (0.4) |
| Alsek | 0.0 (0.3) | 0.0 (0.1) | 0.0 (0.0) | 0.0 (0.0) | 0.0 (0.0) | 0.0 (0.0) | 0.0 (0.0) | | 0.0 (0.0) | 0.0 (0.1) | 0.0 (0.1) |
| Unuk | 0.0 (0.0) | 0.3 (0.7) | 0.0 (0.0) | 0.0 (0.0) | 0.0 (0.0) | 0.0 (0.0) | 0.0 (0.0) | | 0.0 (0.0) | 0.0 (0.5) | 0.0 (0.0) |
| Taku_early timing | 0.0 (0.1) | 0.0 (0.0) | 0.0 (0.0) | 0.0 (0.0) | 0.0 (0.0) | 0.0 (0.0) | 0.0 (0.0) | | 0.0 (0.0) | 0.0 (0.0) | 0.0 (1.2) |
| Taku_mid timing | 0.0 (0.0) | 0.0 (0.0) | 0.0 (0.1) | 0.0 (0.1) | 0.0 (0.0) | 0.0 (0.1) | 0.0 (0.0) | | 0.0 (0.0) | 0.0 (0.0) | 0.0 (0.8) |
| Taku_late timing | 0.0 (0.4) | 0.0 (0.0) | 0.0 (0.0) | 0.0 (0.0) | 0.0 (0.0) | 0.0 (0.0) | 0.0 (0.0) | | 0.0 (0.3) | 0.0 (0.0) | 0.0 (0.0) |
| Stikine_early timing | 0.0 (0.0) | 0.0 (0.0) | 0.0 (0.1) | 0.0 (0.8) | 0.0 (0.0) | 0.0 (0.0) | 0.0 (0.1) | | 0.0 (0.0) | 0.0 (0.1) | 0.0 (1.7) |
| Stikine_late timing | 0.0 (0.0) | 0.0 (0.0) | 0.0 (0.0) | 0.0 (0.5) | 0.0 (0.0) | 0.0 (0.1) | 0.0 (0.1) | | 0.0 (0.0) | 0.0 (0.2) | 0.0 (0.0) |
| Haida Gwaii-North | 0.0 (0.4) | 0.0 (0.0) | 0.0 (0.0) | 0.0 (0.0) | 0.0 (0.0) | 0.0 (0.0) | 0.0 (0.0) | | 0.0 (0.0) | 0.0 (0.0) | 0.0 (0.0) |
| Upper Nass | 0.0 (1.4) | 5.2 (1.9) | 0.9 (0.8) | 0.0 (0.1) | 0.5 (0.3) | 0.0 (0.0) | 1.1 (0.7) | | 0.0 (0.1) | 0.0 (0.0) | 0.0 (0.2) |
| Portland Sound-Observatory Inlet-Lower Nass | 0.0 (0.7) | 4.1 (1.9) | 0.0 (0.1) | 2.9 (3.4) | 0.0 (0.0) | 0.0 (0.0) | 0.0 (0.1) | | 1.0 (1.4) | 0.0 (0.0) | 0.0 (0.0) |
| Ecstall | 0.0 (0.0) | 1.2 (0.9) | 0.0 (0.0) | 2.9 (2.4) | 1.9 (0.8) | 0.0 (0.0) | 0.0 (0.0) | | 0.0 (0.4) | 0.0 (0.0) | 0.0 (0.0) |
| Skeena Estuary | 0.0 (0.0) | 0.6 (0.6) | 1.9 (1.2) | 2.9 (3.0) | 0.0 (0.0) | 0.0 (0.0) | 0.0 (0.0) | | 0.0 (0.0) | 0.0 (0.0) | 0.0 (0.0) |
| Lower Skeena | 0.4 (0.7) | 1.4 (1.1) | 0.0 (0.0) | 0.0 (0.5) | 4.3 (1.0) | 0.0 (0.0) | 0.0 (0.0) | | 1.0 (1.2) | 0.0 (0.1) | 0.0 (0.5) |
| Kalum_early timing | 0.1 (0.8) | 0.0 (0.0) | 0.0 (0.0) | 0.0 (0.0) | 0.0 (0.0) | 0.0 (0.0) | 0.0 (0.0) | | 0.0 (0.0) | 0.0 (0.0) | 0.0 (0.0) |
| Kalum_late timing | 2.4 (2.3) | 6.2 (1.9) | 5.7 (1.9) | 5.9 (3.2) | 27.3 (2.4) | 0.0 (0.0) | 0.0 (0.0) | | 0.0 (0.0) | 0.0 (0.0) | 0.0 (0.0) |
| Zymoetz | 0.5 (0.9) | 2.0 (1.4) | 0.0 (0.0) | 0.0 (0.0) | 3.2 (0.9) | 0.0 (0.0) | 0.0 (0.0) | | 0.0 (0.1) | 0.0 (0.0) | 0.0 (0.3) |
| Sicintine | 0.0 (0.0) | 0.0 (0.0) | 0.0 (0.0) | 0.0 (0.0) | 0.0 (0.2) | 0.0 (0.0) | 0.0 (0.0) | | 0.0 (0.0) | 0.0 (0.0) | 0.0 (0.0) |
| Middle Skeena-mainstem tributaries | 6.1 (2.7) | 8.2 (2.1) | 0.3 (0.7) | 0.1 (0.3) | 11.8 (2.2) | 0.0 (0.1) | 0.0 (0.0) | | 0.0 (0.2) | 0.0 (0.0) | 0.0 (0.3) |
| Middle Skeena-large lakes | 2.5 (2.3) | 0.2 (0.6) | 7.2 (2.7) | 0.0 (0.2) | 46.2 (2.5) | 0.0 (0.0) | 0.0 (0.1) | | 0.0 (0.0) | 2.6 (1.4) | 0.0 (0.3) |
| Upper Skeena | 0.0 (0.0) | 3.1 (1.6) | 0.0 (0.1) | 2.8 (3.2) | 3.6 (1.2) | 0.0 (0.0) | 0.0 (0.0) | 0.0 (0.1) | | 0.0 (0.0) | 0.0 (0.7) |
| Upper Bulkley River | 1.4 (1.0) | 0.0 (0.0) | 0.0 (0.1) | 0.0 (0.0) | 0.0 (0.0) | 0.0 (0.0) | 0.0 (0.0) | 0.0 (0.2) | | 0.0 (0.0) | 0.0 (0.0) |
| North and Central Coast-late timing | 3.3 (2.3) | 0.0 (0.0) | 1.9 (1.1) | 0.0 (0.0) | 0.1 (0.2) | 2.6 (1.1) | 0.0 (0.0) | 0.0 (0.0) | | 0.0 (0.0) | 0.0 (0.2) |
| North and Central Coast-early timing | 9.0 (4.1) | 6.9 (1.6) | 1.9 (1.2) | 0.0 (0.0) | 0.0 (0.2) | 3.2 (1.6) | 0.0 (0.2) | 2.0 (1.7) | | 0.0 (0.2) | 0.0 (2.1) |
| Rivers Inlet | 0.0 (0.0) | 1.2 (1.1) | 1.1 (0.8) | 0.0 (0.3) | 0.0 (0.0) | 0.0 (0.0) | 0.0 (0.1) | 3.1 (1.8) | | 4.7 (2.2) | 0.0 (0.0) |
| Wannock | 0.0 (0.0) | 0.0 (0.0) | 0.0 (0.0) | 5.9 (3.2) | 0.0 (0.0) | 0.0 (0.1) | 0.0 (0.0) | 0.0 (0.0) | | 0.0 (0.0) | 5.6 (6.2) |
| Bella Coola-Bentinck | 4.5 (2.1) | 4.0 (1.7) | 4.2 (2.5) | 0.0 (0.0) | 0.0 (0.0) | 29.0 (4.5) | 90.5 (2.2) | 10.4 (2.6) | | 11.9 (3.8) | 0.0 (0.2) |
| Dean River | 0.0 (0.0) | 0.0 (0.0) | 0.2 (0.6) | 0.0 (0.0) | 0.0 (0.0) | 4.5 (1.9) | 8.3 (2.0) | 3.2 (1.9) | | 1.3 (1.4) | 0.0 (0.0) |
| Docee | 0.0 (0.1) | 0.0 (0.0) | 0.0 (0.0) | 2.9 (2.5) | 0.0 (0.0) | 0.0 (0.0) | 0.0 (0.0) | 0.0 (0.1) | | 0.0 (0.1) | 0.0 (0.0) |
| Klinaklini_SU_1.3 | 0.0 (0.0) | 0.0 (0.1) | 3.9 (2.3) | 0.0 (0.3) | 0.5 (0.3) | 2.1 (1.2) | 0.0 (0.0) | 1.0 (1.4) | | 2.8 (2.2) | 0.0 (0.1) |
| Southern Mainland-Southern Fjords_FA_0.x | 0.0 (0.0) | 1.3 (0.6) | 2.8 (1.4) | 0.0 (0.1) | 0.0 (0.0) | 0.9 (0.9) | 0.0 (0.1) | 2.1 (1.5) | | 2.6 (1.3) | 0.0 (0.1) |
| Southern Mainland-Georgia Strait_FA_0.x | 0.0 (0.1) | 0.0 (0.0) | 0.1 (0.2) | 0.0 (1.1) | 0.0 (0.0) | 0.1 (0.3) | 0.0 (0.0) | 3.1 (1.8) | | 6.3 (3.0) | 0.0 (0.3) |
| Upper Fraser River_SP_1.3 | 0.0 (0.2) | 0.6 (0.6) | 0.0 (0.2) | 0.0 (0.9) | 0.0 (0.0) | 0.0 (0.2) | 0.0 (0.1) | 0.0 (0.1) | | 0.0 (0.3) | 0.0 (0.9) |
| Middle Fraser River_SU_1.3 | 0.0 (0.1) | 0.0 (0.1) | 0.0 (0.0) | 0.0 (0.0) | 0.0 (0.0) | 0.0 (0.1) | 0.0 (0.0) | 0.0 (0.2) | | 0.0 (0.4) | 0.0 (0.1) |
| Middle Fraser River_SP_1.3 | 0.0 (0.1) | 0.0 (0.1) | 0.9 (1.1) | 0.0 (0.3) | 0.0 (0.0) | 0.0 (0.1) | 0.0 (0.1) | 0.0 (0.1) | | 0.0 (0.3) | 0.0 (1.0) |
| Middle Fraser River-Portage_FA_1.3 | 0.0 (0.0) | 0.0 (0.0) | 0.0 (0.0) | 0.0 (0.0) | 0.0 (0.0) | 0.0 (0.0) | 0.0 (0.0) | 0.0 (0.0) | | 0.0 (0.0) | 0.0 (0.0) |
| Middle Fraser-Fraser Canyon_SP_1.3 | 0.0 (0.0) | 0.0 (0.0) | 0.0 (0.0) | 0.0 (0.0) | 0.0 (0.0) | 0.0 (0.0) | 0.0 (0.0) | 0.0 (0.1) | | 0.0 (0.1) | 0.0 (0.0) |
| North Thompson_SP_1.3 | 0.0 (0.0) | 0.0 (0.0) | 0.0 (0.0) | 0.0 (0.0) | 0.0 (0.0) | 0.0 (0.1) | 0.0 (0.0) | 0.1 (0.4) | | 0.0 (0.1) | 0.0 (0.0) |
| North Thompson_SU_1.3 | 0.0 (0.2) | 0.0 (0.0) | 0.0 (0.0) | 0.0 (0.3) | 0.0 (0.0) | 0.0 (0.0) | 0.0 (0.0) | 0.7 (1.2) | | 0.0 (0.1) | 0.0 (1.1) |
| Shuswap River_SU_0.3 | 2.8 (2.0) | 0.0 (0.0) | 1.9 (1.0) | 0.0 (0.0) | 0.0 (0.0) | 0.9 (0.9) | 0.0 (0.0) | 2.1 (1.4) | | 0.0 (0.5) | 0.0 (0.1) |
| South Thompson-Bessette Creek_SU_1.2 | 0.1 (1.2) | 0.0 (0.0) | 0.0 (0.0) | 0.0 (0.4) | 0.0 (0.0) | 0.0 (0.0) | 0.0 (0.0) | 0.0 (0.0) | | 0.0 (0.0) | 0.0 (0.5) |
| South Thompson_SU_0.3 | 3.1 (1.6) | 5.1 (1.6) | 18.9 (3.4) | 26.4 (6.7) | 0.5 (0.3) | 3.4 (1.5) | 0.0 (0.0) | 1.0 (0.9) | | 5.1 (2.0) | 16.7 (6.6) |
| South Thompson_SU_1.3 | 0.1 (0.6) | 0.0 (0.0) | 0.0 (0.0) | 0.0 (0.0) | 0.0 (0.0) | 0.0 (0.0) | 0.0 (0.1) | 0.0 (0.4) | | 0.0 (0.0) | 0.0 (0.1) |
| Lower Thompson_SP_1.2 | 0.0 (0.3) | 0.0 (0.0) | 0.0 (0.1) | 0.0 (0.6) | 0.0 (0.1) | 0.0 (0.3) | 0.0 (0.0) | 0.0 (0.3) | | 0.0 (0.0) | 0.0 (0.2) |
| Lower Fraser River_SP_1.3 | 0.0 (0.0) | 0.0 (0.0) | 0.0 (0.0) | 0.0 (0.0) | 0.0 (0.0) | 0.0 (0.0) | 0.0 (0.0) | 0.0 (0.0) | | 0.0 (0.0) | 0.0 (0.0) |
| Lower Fraser River_SU_1.3 | 0.0 (0.0) | 0.0 (0.1) | 0.0 (0.2) | 0.0 (0.3) | 0.0 (0.0) | 0.0 (0.0) | 0.0 (0.0) | 0.0 (0.0) | | 0.0 (0.0) | 0.0 (0.0) |
| Lower Fraser River-Upper Pitt_SU_1.3 | 0.0 (0.0) | 0.0 (0.0) | 0.0 (0.0) | 0.0 (0.1) | 0.0 (0.0) | 0.9 (0.8) | 0.0 (0.0) | 1.0 (0.9) | | 0.0 (0.0) | 0.0 (0.0) |
| Maria Slough_SU_0.3 | 0.0 (0.0) | 0.0 (0.0) | 0.0 (0.0) | 0.0 (0.0) | 0.0 (0.0) | 0.0 (0.1) | 0.0 (0.0) | 0.0 (0.0) | | 0.0 (0.1) | 0.0 (0.3) |
| Lower Fraser River_FA_0.3 | 1.5 (1.5) | 1.2 (0.7) | 0.0 (0.0) | 0.0 (0.1) | 0.0 (0.0) | 0.9 (0.8) | 0.0 (0.0) | 1.0 (1.0) | | 3.8 (2.0) | 5.6 (3.7) |
| East Vancouver Island-North_FA_0.x | 13.6 (4.0) | 2.4 (1.1) | 4.7 (1.9) | 8.8 (3.5) | 0.0 (0.0) | 10.3 (2.9) | 0.0 (0.0) | 5.2 (2.4) | | 10.2 (3.9) | 16.7 (9.7) |
| East Vancouver Island-Qualicum and Puntledge_FA_0.x | 6.1 (3.5) | 6.4 (1.7) | 5.7 (2.2) | 5.9 (3.6) | 0.0 (0.0) | 5.9 (2.3) | 0.0 (0.0) | 9.9 (3.0) | | 15.0 (3.7) | 27.8 (8.5) |
| East Vancouver Island-Nanaimo and Chemainus_FA_0.x | 0.0 (0.0) | 0.0 (0.0) | 0.0 (0.0) | 0.0 (0.0) | 0.0 (0.0) | 0.0 (0.0) | 0.0 (0.0) | 0.0 (0.0) | | 0.0 (0.1) | 0.0 (0.1) |
| East Vancouver Island-Nanaimo_SP_1.x | 0.0 (0.1) | 0.0 (0.0) | 0.0 (0.0) | 0.0 (0.0) | 0.0 (0.0) | 0.0 (0.0) | 0.0 (0.0) | 0.0 (0.0) | | 0.0 (0.0) | 0.0 (0.0) |
| East Vancouver Island-Georgia Strait_SU_0.3 | 0.0 (0.0) | 0.0 (0.0) | 0.0 (0.0) | 0.0 (0.0) | 0.0 (0.0) | 0.0 (0.0) | 0.0 (0.3) | 0.0 (0.0) | | 0.0 (0.0) | 0.0 (0.0) |
| East Vancouver Island-Cowichan and Koksilah_FA_0.x | 0.0 (0.0) | 0.8 (0.6) | 0.0 (0.0) | 0.0 (0.0) | 0.0 (0.0) | 0.9 (1.0) | 0.0 (0.0) | 2.4 (2.0) | | 0.0 (0.0) | 0.0 (0.0) |
| West Vancouver Island-Nootka and Kyuquot_FA_0.x | 6.1 (3.5) | 0.6 (0.5) | 0.6 (0.9) | 3.2 (3.2) | 0.0 (0.0) | 9.0 (2.4) | 0.0 (0.1) | 8.2 (3.1) | | 2.6 (1.3) | 0.0 (1.1) |
| West Vancouver Island-North_FA_0.x | 0.0 (0.0) | 0.0 (0.0) | 0.0 (0.0) | 0.0 (0.0) | 0.0 (0.1) | 0.0 (0.1) | 0.0 (0.0) | 0.0 (0.0) | | 0.0 (0.0) | 0.0 (0.0) |
| West Vancouver Island-South_FA_0.x | 19.7 (4.5) | 13.1 (3.0) | 15.4 (3.6) | 23.3 (6.4) | 0.0 (0.0) | 16.0 (3.2) | 0.0 (0.1) | 27.9 (4.3) | | 12.8 (3.8) | 22.2 (8.8) |
| Okanagan_1.x | 0.0 (0.0) | 0.5 (1.1) | 0.0 (0.0) | 0.0 (0.0) | 0.0 (0.0) | 0.0 (0.0) | 0.0 (0.0) | 0.0 (0.0) | | 0.0 (0.0) | 0.0 (0.0) |
| Juan de Fuca | 3.0 (2.1) | 1.2 (0.7) | 0.0 (0.0) | 0.0 (0.0) | 0.0 (0.0) | 0.0 (0.0) | 0.0 (0.0) | 0.0 (0.0) | | 0.0 (0.0) | 0.0 (0.0) |
| Coastal Washington | 0.0 (0.0) | 0.6 (0.5) | 1.9 (1.5) | 0.0 (0.1) | 0.0 (0.0) | 0.0 (0.1) | 0.0 (0.2) | 1.0 (0.8) | | 0.0 (0.0) | 0.0 (0.4) |
| North Puget Sound | 1.4 (1.4) | 5.1 (1.7) | 5.2 (2.9) | 0.0 (0.2) | 0.0 (0.0) | 4.1 (1.8) | 0.0 (0.0) | 2.1 (1.2) | | 0.1 (0.9) | 0.0 (0.1) |
| South Puget Sound | 3.0 (2.1) | 8.5 (2.4) | 1.3 (1.5) | 0.0 (0.2) | 0.0 (0.0) | 3.4 (1.6) | 0.0 (0.0) | 8.2 (2.0) | | 9.4 (3.0) | 0.0 (0.1) |
| Lower Columbia River | 3.0 (1.7) | 1.2 (0.7) | 0.0 (0.0) | 0.0 (0.1) | 0.0 (0.0) | 0.0 (0.0) | 0.0 (0.1) | 0.0 (0.0) | | 2.1 (1.6) | 0.0 (0.0) |
| Mid Columbia River_SP | 0.1 (1.0) | 0.0 (0.1) | 0.1 (0.1) | 0.0 (0.4) | 0.0 (0.0) | 0.2 (0.8) | 0.0 (0.0) | 0.0 (0.1) | | 2.5 (1.6) | 0.0 (0.1) |
| Upper Columbia River_SP | 0.0 (0.1) | 0.0 (0.0) | 0.0 (0.0) | 0.0 (0.0) | 0.0 (0.0) | 0.0 (0.0) | 0.0 (0.0) | 0.0 (0.0) | | 0.0 (0.1) | 0.0 (0.0) |
| Upper Columbia River_SU_FA | 2.9 (2.1) | 4.8 (1.9) | 11.1 (3.0) | 0.3 (0.6) | 0.0 (0.0) | 0.0 (0.1) | 0.0 (0.1) | 0.0 (0.1) | | 2.3 (1.5) | 5.5 (4.8) |
| Snake River_FA | 0.2 (0.2) | 2.1 (1.4) | 0.2 (0.5) | 5.5 (4.1) | 0.0 (0.0) | 0.9 (0.9) | 0.0 (0.0) | 2.0 (1.7) | | 1.5 (1.6) | 0.0 (0.0) |
| Snake River_SP_SU | 0.0 (0.2) | 0.0 (0.0) | 0.0 (0.1) | 0.0 (0.3) | 0.0 (0.1) | 0.0 (0.1) | 0.0 (0.0) | 0.0 (0.1) | | 0.0 (0.1) | 0.0 (0.4) |
| North & Central Oregon | 0.0 (0.0) | 0.0 (0.0) | 0.0 (0.0) | 0.0 (0.2) | 0.0 (0.0) | 0.0 (0.1) | 0.0 (0.0) | 0.0 (0.1) | | 0.0 (0.2) | 0.0 (0.2) |
| Upper Willamette River | 1.5 (1.4) | 0.0 (0.0) | 0.0 (0.0) | 0.0 (0.1) | 0.0 (0.0) | 0.0 (0.0) | 0.0 (0.0) | 0.0 (0.1) | | 0.4 (0.8) | 0.0 (0.1) |
| South Oregon coastal | 0.0 (0.0) | 0.0 (0.0) | 0.0 (0.2) | 0.0 (0.1) | 0.0 (0.0) | 0.0 (0.1) | 0.0 (0.0) | 0.0 (0.1) | | 0.0 (0.1) | 0.0 (0.1) |
| California Klamath Trinity | 0.0 (0.1) | 0.0 (0.0) | 0.0 (0.1) | 0.0 (0.1) | 0.0 (0.0) | 0.0 (0.0) | 0.0 (0.0) | 0.0 (0.1) | | 0.0 (0.1) | 0.0 (0.1) |
| California Central Valley_Fall | 1.5 (1.2) | 0.0 (0.1) | 0.0 (0.0) | 0.0 (0.4) | 0.0 (0.0) | 0.8 (0.6) | 0.0 (0.0) | 0.0 (0.1) | | 0.0 (0.1) | 0.0 (0.0) |
| California Central Valley_Spring | 0.0 (0.1) | 0.0 (0.0) | 0.0 (0.0) | 0.0 (0.0) | 0.0 (0.0) | 0.1 (0.1) | 0.0 (0.0) | 0.0 (0.0) | | 0.0 (0.0) | 0.0 (0.0) |
| Coastal California | 0.0 (0.0) | 0.0 (0.0) | 0.0 (0.0) | 0.0 (0.0) | 0.0 (0.0) | 0.0 (0.0) | 0.0 (0.0) | 0.0 (0.1) | | 0.0 (0.0) | 0.0 (0.0) |

Supplementary Table S5 continued

| Conservation Unit | Area 10 sport | | | Area 11 sport | | | Area 111 sport | | |
| --- | --- | --- | --- | --- | --- | --- | --- | --- | --- |
|  | June | July | August | June | July | August | June | July | August |
| Sample size | 42 | 25 | 2 | 270 | 287 | 80 | 7 | 32 | 17 |
| N PBT | 11 | 10 | 1 | 75 | 83 | 32 | 1 | 11 | 5 |
| Southeast Alaska | 0.0 (0.0) | 0.0 (0.1) | 0.0 (0.5) | 0.0 (0.0) | 0.0 (0.0) | 0.0 (0.2) | 0.0 (0.4) | 0.0 (0.1) | 0.0 (0.2) |
| Alsek | 0.0 (0.1) | 0.0 (0.2) | 0.0 (6.4) | 0.0 (0.0) | 0.0 (0.0) | 0.0 (0.6) | 0.0 (1.2) | 0.0 (0.3) | 0.0 (0.0) |
| Unuk | 0.0 (0.1) | 0.0 (0.2) | 0.0 (0.0) | 0.0 (0.1) | 0.0 (0.0) | 0.0 (0.0) | 0.0 (0.0) | 0.0 (0.0) | 0.0 (0.0) |
| Taku_early timing | 0.0 (0.1) | 0.0 (0.0) | 0.0 (0.1) | 0.0 (0.1) | 0.0 (0.0) | 0.0 (0.0) | 0.0 (0.1) | 0.0 (0.1) | 0.0 (0.0) |
| Taku_mid timing | 0.0 (0.0) | 0.0 (0.2) | 0.0 (0.5) | 0.0 (0.0) | 0.0 (0.0) | 0.0 (0.0) | 0.0 (1.5) | 0.0 (0.0) | 0.0 (0.2) |
| Taku_late timing | 0.0 (0.2) | 0.0 (0.0) | 0.0 (0.1) | 0.0 (0.0) | 0.0 (0.0) | 0.0 (0.1) | 0.0 (0.2) | 0.0 (0.0) | 0.0 (0.0) |
| Stikine_early timing | 0.0 (0.0) | 0.0 (0.2) | 0.0 (0.2) | 0.0 (0.0) | 0.0 (0.0) | 0.0 (0.1) | 0.0 (0.0) | 0.0 (0.4) | 0.0 (0.1) |
| Stikine_late timing | 0.0 (0.0) | 0.0 (0.7) | 0.0 (0.1) | 0.0 (0.0) | 0.0 (0.0) | 0.0 (0.1) | 0.0 (0.6) | 0.0 (0.2) | 0.0 (0.0) |
| Haida Gwaii-North | 0.0 (0.0) | 0.0 (0.0) | 0.0 (0.0) | 0.0 (0.0) | 0.0 (0.0) | 0.0 (0.0) | 0.0 (0.3) | 0.0 (0.0) | 0.0 (0.0) |
| Upper Nass | 0.0 (0.1) | 0.0 (0.5) | 0.0 (3.4) | 0.7 (0.7) | 0.0 (0.0) | 0.0 (0.1) | 0.0 (1.5) | 0.0 (0.4) | 0.0 (0.9) |
| Portland Sound-Observatory Inlet-Lower Nass | 2.1 (3.0) | 0.0 (0.1) | 0.0 (3.3) | 0.1 (0.2) | 0.0 (0.0) | 0.0 (0.0) | 0.0 (1.5) | 0.0 (0.0) | 0.0 (1.2) |
| Ecstall | 0.0 (0.0) | 0.0 (0.0) | 0.0 (0.1) | 0.0 (0.2) | 0.0 (0.0) | 0.0 (0.0) | 0.0 (0.9) | 0.0 (0.0) | 0.0 (0.0) |
| Skeena Estuary | 0.0 (0.0) | 0.0 (0.0) | 0.0 (0.0) | 0.0 (0.0) | 0.0 (0.0) | 0.0 (0.0) | 0.0 (0.1) | 0.0 (0.0) | 0.0 (0.0) |
| Lower Skeena | 0.0 (0.1) | 0.0 (0.3) | 0.0 (2.6) | 0.5 (0.4) | 0.3 (0.2) | 0.0 (0.3) | 0.0 (0.1) | 0.0 (0.0) | 0.0 (0.0) |
| Kalum_early timing | 0.0 (0.4) | 0.0 (0.1) | 0.0 (2.5) | 0.0 (0.0) | 0.0 (0.0) | 0.0 (0.0) | 0.0 (0.0) | 0.0 (0.0) | 0.0 (0.0) |
| Kalum_late timing | 0.0 (0.1) | 0.0 (0.0) | 0.0 (0.1) | 0.0 (0.1) | 0.0 (0.0) | 0.0 (0.0) | 0.0 (0.0) | 0.0 (0.0) | 0.0 (0.0) |
| Zymoetz | 0.0 (0.1) | 0.0 (0.2) | 0.0 (0.1) | 0.0 (0.0) | 0.0 (0.0) | 0.0 (0.1) | 0.0 (0.4) | 0.0 (0.1) | 0.0 (0.0) |
| Sicintine | 0.0 (0.1( | 0.0 (0.0) | 0.0 (0.0) | 0.0 (0.0) | 0.0 (0.1) | 0.0 (0.0) | 0.0 (0.7) | 0.0 (0.0) | 0.0 (0.5) |
| Middle Skeena-mainstem tributaries | 0.0 (0.1) | 0.0 (0.1) | 0.0 (6.3) | 0.5 (0.5) | 0.0 (0.1) | 0.0 (0.2) | 0.0 (0.7) | 0.0 (0.1) | 0.0 (0.4) |
| Middle Skeena-large lakes | 0.0 (0.0) | 0.0 (0.7) | 0.0 (0.2) | 0.0 (0.0) | 0.0 (0.0) | 0.0 (0.0) | 0.0 (0.0) | 0.0 (0.0) | 0.0 (0.0) |
| Upper Skeena | 0.0 (0.2) | 0.0 (0.5) | 0.0 (3.8) | 0.0 (0.2) | 0.0 (0.0) | 0.0 (0.1) | 0.0 (1.1) | 0.0 (0.0) | 0.0 (0.5) |
| Upper Bulkley River | 0.0 (0.1) | 0.0 (0.0) | 0.0 (1.2) | 0.0 (0.0) | 0.0 (0.0) | 0.0 (0.0) | 0.0 (0.0) | 0.0 (0.0) | 0.0 (0.0) |
| North and Central Coast-late timing | 2.4 (2.4) | 0.0 (0.0) | 0.0 (4.9) | 1.9 (0.9) | 2.8 (0.8) | 1.3 (1.0) | 0.0 (0.1) | 0.0 (0.0) | 0.0 (0.0) |
| North and Central Coast-early timing | 0.0 (1.0) | 0.0 (0.0) | 0.0 (0.7) | 0.0 (0.2) | 0.0 (0.0) | 0.0 (0.1) | 0.0 (0.0) | 0.0 (0.0) | 0.0 (0.1) |
| Rivers Inlet | 3.1 (2.9) | 0.0 (0.3) | 0.0 (3.3) | 1.9 (0.7) | 0.4 (0.4) | 0.0 (0.0) | 0.0 (0.7) | 0.0 (0.0) | 0.0 (0.0) |
| Wannock | 0.0 (0.0) | 0.0 (0.0) | 0.0 (0.0) | 0.4 (0.3) | 0.0 (0.0) | 0.0 (0.0) | 0.0 (0.0) | 0.0 (0.0) | 0.0 (0.0) |
| Bella Coola-Bentinck | 9.0 (5.0) | 0.0 (0.6) | 0.0 (7.7) | 1.5 (0.9) | 1.1 (0.8) | 0.0 (0.5) | 0.0 (0.6) | 0.0 (0.1) | 0.0 (0.1) |
| Dean River | 0.0 (0.0) | 0.0 (0.0) | 0.0 (0.2) | 1.1 (0.6) | 0.0 (0.0) | 0.0 (0.0) | 0.0 (0.1) | 0.0 (0.2) | 0.0 (0.0) |
| Docee | 0.0 (0.0) | 0.0 (0.0) | 0.0 (0.0) | 0.0 (0.0) | 0.0 (0.0) | 0.0 (0.0) | 0.0 (0.3) | 0.0 (0.0) | 0.0 (0.0) |
| Klinaklini_SU_1.3 | 7.1 (3.3) | 0.0 (0.0) | 0.0 (0.2) | 1.5 (0.8) | 1.7 (0.8) | 0.0 (0.0) | 0.0 (0.7) | 0.0 (0.0) | 0.0 (0.2) |
| Southern Mainland-Southern Fjords_FA_0.x | 0.0 (0.0) | 4.0 (3.8) | 0.0 (0.4) | 0.4 (0.3) | 0.7 (0.4) | 0.0 (0.0) | 0.0 (0.1) | 0.0 (0.0) | 0.0 (0.0) |
| Southern Mainland-Georgia Strait_FA_0.x | 0.0 (0.4) | 0.0 (0.0) | 0.0 (3.4) | 0.0 (0.1) | 0.3 (0.3) | 1.2 (1.0) | 0.0 (0.5) | 0.0 (0.2) | 0.0 (0.6) |
| Upper Fraser River_SP_1.3 | 0.0 (0.6) | 0.0 (0.5) | 0.0 (1.7) | 0.0 (0.0) | 0.0 (0.1) | 0.0 (0.1) | 0.0 (0.6) | 0.0 (0.3) | 0.0 (1.0) |
| Middle Fraser River_SU_1.3 | 0.0 (0.2) | 0.0 (0.2) | 0.0 (2.1) | 0.0 (0.1) | 0.0 (0.0) | 0.0 (0.1) | 0.0 (0.2) | 0.0 (0.0) | 0.0 (0.5) |
| Middle Fraser River_SP_1.3 | 0.0 (1.3) | 0.0 (0.2) | 0.0 (1.4) | 0.0 (0.0) | 0.0 (0.0) | 0.0 (0.3) | 0.0 (1.2) | 0.0 (0.5) | 0.0 (0.4) |
| Middle Fraser River-Portage_FA_1.3 | 0.0 (0.0) | 0.0 (0.0) | 0.0 (0.7) | 0.0 (0.0) | 0.0 (0.0) | 0.0 (0.0) | 0.0 (0.0) | 0.0 (0.0) | 0.0 (0.0) |
| Middle Fraser-Fraser Canyon_SP_1.3 | 0.0 (0.0) | 0.0 (0.0) | 0.0 (0.2) | 0.0 (0.0) | 0.0 (0.0) | 0.0 (0.0) | 0.0 (0.3) | 0.0 (0.0) | 0.0 (0.1) |
| North Thompson_SP_1.3 | 0.0 (0.0) | 0.0 (0.0) | 0.0 (3.7) | 0.0 (0.0) | 0.0 (0.0) | 0.0 (0.0) | 0.0 (0.1) | 0.0 (0.2) | 0.0 (1.3) |
| North Thompson_SU_1.3 | 0.0 (0.2) | 0.0 (0.5) | 0.0 (0.1) | 0.0 (0.0) | 0.0 (0.0) | 0.0 (0.0) | 0.0 (0.9) | 0.0 (0.1) | 0.0 (0.2) |
| Shuswap River_SU_0.3 | 0.0 (0.3) | 0.0 (0.0) | 0.0 (0.0) | 0.4 (0.5) | 0.0 (0.0) | 0.0 (0.0) | 0.0 (0.0) | 0.0 (1.0) | 0.0 (0.2) |
| South Thompson-Bessette Creek_SU_1.2 | 0.0 (0.0) | 0.0 (0.4) | 0.0 (1.4) | 0.0 (0.0) | 0.0 (0.0) | 0.0 (0.0) | 0.0 (0.0) | 0.0 (0.0) | 0.0 (0.0) |
| South Thompson_SU_0.3 | 0.0 (0.0) | 0.0 (0.2) | 0.0 (2.5) | 0.4 (0.4) | 0.7 (0.5) | 6.3 (2.1) | 0.0 (0.0) | 0.0 (0.0) | 0.0 (0.0) |
| South Thompson_SU_1.3 | 0.0 (0.0) | 0.0 (0.0) | 0.0 (0.4) | 0.0 (0.0) | 0.0 (0.0) | 0.0 (0.0) | 0.0 (0.9) | 0.0 (0.6) | 0.0 (0.7) |
| Lower Thompson_SP_1.2 | 0.0 (0.6) | 0.0 (0.3) | 0.0 (1.7) | 0.0 (0.0) | 0.0 (0.0) | 0.0 (0.1) | 0.0 (0.2) | 0.0 (0.6) | 0.0 (0.8) |
| Lower Fraser River_SP_1.3 | 0.0 (0.0) | 0.0 (0.2) | 0.0 (1.0) | 0.0 (0.0) | 0.0 (0.0) | 0.0 (0.0) | 0.0 (0.1) | 0.0 (0.0) | 0.0 (0.0) |
| Lower Fraser River_SU_1.3 | 0.0 (0.4) | 0.0 (0.0) | 0.0 (0.4) | 0.0 (0.0) | 0.0 (0.0) | 0.0 (0.0) | 0.0 (0.0) | 0.0 (0.1) | 0.0 (0.3) |
| Lower Fraser River-Upper Pitt_SU_1.3 | 2.4 (2.2) | 0.0 (0.1) | 0.0 (1.9) | 0.7 (0.5) | 0.3 (0.4) | 0.0 (0.1) | 0.0 (0.0) | 0.0 (0.1) | 0.0 (0.2) |
| Maria Slough_SU_0.3 | 0.0 (0.0) | 0.0 (0.0) | 0.0 (0.0) | 0.0 (0.0) | 0.0 (0.0) | 0.0 (0.0) | 0.0 (0.0) | 0.0 (0.0) | 0.0 (0.0) |
| Lower Fraser River_FA_0.3 | 0.0 (0.3) | 0.0 (0.0) | 0.0 (10.2) | 0.0 (0.0) | 0.3 (0.3) | 0.0 (0.0) | 0.0 (0.0) | 0.0 (0.1) | 0.0 (0.6) |
| East Vancouver Island-North_FA_0.x | 4.8 (3.1) | 16.0 (6.1) | 100.0 (23.6) | 2.6 (0.9) | 5.6 (1.5) | 10.0 (3.6) | 0.0 (2.6) | 6.3 (3.9) | 11.8 (7.1) |
| East Vancouver Island-Qualicum and Puntledge_FA_0.x | 4.8 (3.3) | 4.0 (3.0) | 0.0 (0.7) | 4.0 (1.4) | 5.0 (1.1) | 0.0 (0.0) | 15.3 (13.4) | 0.0 (0.2) | 11.8 (7.4) |
| East Vancouver Island-Nanaimo and Chemainus_FA_0.x | 0.0 (0.2) | 0.0 (0.0) | 0.0 (2.2) | 0.0 (0.0) | 0.0 (0.0) | 0.0 (0.0) | 0.0 (0.0) | 0.0 (0.0) | 0.0 (0.5) |
| East Vancouver Island-Nanaimo_SP_1.x | 0.0 (0.0) | 0.0 (0.0) | 0.0 (0.0) | 0.0 (0.0) | 0.0 (0.0) | 0.0 (0.0) | 0.0 (0.0) | 0.0 (0.0) | 0.0 (0.0) |
| East Vancouver Island-Georgia Strait_SU_0.3 | 0.0 (0.2) | 0.0 (0.3) | 0.0 (0.5) | 0.4 (0.4) | 0.0 (0.1) | 0.0 (0.0) | 0.0 (0.5) | 0.0 (0.0) | 0.0 (0.0) |
| East Vancouver Island-Cowichan and Koksilah_FA_0.x | 0.0 (0.0) | 0.0 (0.0) | 0.0 (0.2) | 0.0 (0.0) | 1.0 (0.7) | 0.0 (0.0) | 13.2 (11.5) | 0.0 (0.0) | 0.0 (0.0) |
| West Vancouver Island-Nootka and Kyuquot_FA_0.x | 42.9 (6.3) | 36.0 (10.4) | 0.0 (5.3) | 40.2 (3.1) | 42.5 (2.6) | 5.0 (2.3) | 57.1 (17.3) | 46.9 (8.7) | 0.0 (1.0) |
| West Vancouver Island-North_FA_0.x | 0.0 (0.0) | 0.0 (0.0) | 0.0 (0.2) | 0.0 (0.0) | 0.0 (0.0) | 0.0 (0.0) | 0.0 (0.5) | 0.0 (0.1) | 0.0 (0.1) |
| West Vancouver Island-South_FA_0.x | 21.4 (6.4) | 40.0 (9.0) | 0.0 (5.1) | 31.7 (2.7) | 33.8 (2.7) | 63.1 (5.6) | 0.0 (4.5) | 43.7 (9.1) | 64.7 (11.5) |
| Okanagan_1.x | 0.0 (0.0) | 0.0 (0.0) | 0.0 (0.0) | 0.0 (0.0) | 0.0 (0.0) | 0.2 (0.4) | 0.0 (0.2) | 0.0 (0.0) | 0.0 (0.0) |
| Juan de Fuca | 0.0 (0.0) | 0.0 (0.0) | 0.0 (1.2) | 0.4 (0.3) | 0.3 (0.2) | 0.0 (0.0) | 0.0 (0.0) | 0.0 (0.0) | 0.0 (0.0) |
| Coastal Washington | 0.0 (0.3) | 0.0 (1.1) | 0.0 (0.2) | 0.4 (0.3) | 0.5 (0.4) | 1.9 (1.7) | 0.0 (0.1) | 0.0 (0.0) | 0.0 (0.1) |
| North Puget Sound | 0.0 (0.4) | 0.0 (0.0) | 0.0 (0.0) | 3.1 (1.5) | 0.4 (0.4) | 2.3 (1.9) | 0.0 (1.8) | 0.1 (0.5) | 0.5 (1.7) |
| South Puget Sound | 0.0 (0.3) | 0.0 (0.0) | 0.0 (1.8) | 1.9 (0.7) | 1.7 (0.8) | 1.2 (1.2) | 0.0 (1.3) | 3.0 (2.5) | 5.4 (5.1) |
| Lower Columbia River | 0.0 (0.5) | 0.0 (0.2) | 0.0 (5.3) | 0.7 (0.5) | 0.3 (0.4) | 1.2 (1.3) | 0.0 (0.8) | 0.0 (0.0) | 5.9 (3.6) |
| Mid Columbia River_SP | 0.0 (0.1) | 0.0 (0.1) | 0.0 (2.9) | 0.3 (0.4) | 0.0 (0.1) | 0.2 (1.0) | 0.0 (0.3) | 0.0 (0.0) | 0.0 (0.0) |
| Upper Columbia River_SP | 0.0 (0.0) | 0.0 (0.2) | 0.0 (0.1) | 0.0 (0.0) | 0.0 (0.0) | 0.0 (0.0) | 0.0 (0.0) | 0.0 (0.0) | 0.0 (0.1) |
| Upper Columbia River_SU_FA | 0.0 (0.0) | 0.0 (1.4) | 0.0 (0.2) | 2.6 (0.9) | 0.0 (0.0) | 1.2 (2.9) | 0.0 (1.9) | 0.0 (0.2) | 0.0 (0.6) |
| Snake River_FA | 0.0 (0.0) | 0.0 (0.1) | 0.0 (0.0) | 0.0 (0.0) | 0.0 (0.0) | 4.9 (3.7) | 0.0 (0.1) | 0.0 (0.0) | 0.0 (0.0) |
| Snake River_SP_SU | 0.0 (0.3) | 0.0 (0.2) | 0.0 (9.8) | 0.0 (0.0) | 0.0 (0.1) | 0.0 (0.1) | 0.0 (3.0) | 0.0 (0.0) | 0.0 (0.8) |
| North & Central Oregon | 0.0 (0.0) | 0.0 (0.6) | 0.0 (3.1) | 0.0 (0.0) | 0.0 (0.0) | 0.0 (0.3) | 0.0 (0.3) | 0.0 (0.3) | 0.0 (0.7) |
| Upper Willamette River | 0.0 (0.0) | 0.0 (0.0) | 0.0 (0.7) | 0.0 (0.0) | 0.0 (0.0) | 0.0 (0.0) | 0.0 (2.7) | 0.0 (0.1) | 0.0 (0.1) |
| South Oregon coastal | 0.0 (0.5) | 0.0 (0.1) | 0.0 (3.0) | 0.0 (0.0) | 0.0 (0.0) | 0.0 (0.1) | 0.0 (0.5) | 0.0 (0.0) | 0.0 (0.6) |
| California Klamath Trinity | 0.0 (1.5) | 0.0 (0.2) | 0.0 (3.5) | 0.0 (0.0) | 0.0 (0.1) | 0.0 (0.0) | 0.0 (1.1) | 0.0 (0.1) | 0.0 (0.8) |
| California Central Valley_Fall | 0.0 (0.4) | 0.0 (0.3) | 0.0 (2.4) | 0.0 (0.0) | 0.0 (0.1) | 0.0 (0.1) | 0.5 (0.6) | 0.0 (0.0) | 0.0 (0.3) |
| California Central Valley_Spring | 0.0 (0.0) | 0.0 (0.1) | 0.0 (3.2) | 0.0 (0.0) | 0.0 (0.0) | 0.0 (0.0) | 13.8 (12.3) | 0.0 (0.1) | 0.0 (0.1) |
| Coastal California | 0.0 (0.0) | 0.0 (0.0) | 0.0 (0.0) | 0.0 (0.0) | 0.0 (0.0) | 0.0 (0.0) | 0.0 (0.0) | 0.0 (0.1) | 0.0 (1.1) |

Supplementary Table S5 continued

| Conservation Unit | Area 12 sport | | | Area 12 gillnet sockeye test fishery bycatch | | | | |
| --- | --- | --- | --- | --- | --- | --- | --- | --- |
|  | July | August | Sept | July 11 | July 15-20 | July 22-25 | August 1-3 | August 5-9 |
| Sample size | 100 | 287 | 5 | 2 | 36 | 9 | 4 | 8 |
| N PBT | 20 | 77 | 1 | 1 | 6 | 0 | 2 | 2 |
| Southeast Alaska | 0.0 (0.1) | 0.0 (0.0) | 0.0 (1.0) | 0.0 (0.6) | 0.0 (0.1) | 0.0 (0.9) | 0.0 (1.9) | 0.0 (0.1) |
| Alsek | 0.0 (0.0) | 0.0 (0.0) | 0.0 (2.6) | 0.0 (0.1) | 0.0 (0.5) | 0.0 (1.4) | 0.0 (0.2) | 0.0 (3.7) |
| Unuk | 0.0 (0.0) | 0.0 (0.1) | 0.0 (0.0) | 0.0 (0.0) | 0.0 (0.0) | 0.0 (0.0) | 0.0 (1.6) | 0.0 (0.4) |
| Taku_early timing | 0.0 (0.0) | 0.1 (0.2) | 0.0 (1.1) | 0.0 (3.6) | 0.0 (0.0) | 0.0 (0.6) | 0.0 (0.0) | 0.0 (0.3) |
| Taku_mid timing | 0.0 (0.0) | 0.0 (0.1) | 0.0 (0.1) | 0.0 (1.7) | 0.0 (0.0) | 0.0 (0.4) | 0.0 (0.1) | 0.0 (0.8) |
| Taku_late timing | 0.0 (0.1) | 0.0 (0.1) | 0.0 (0.0) | 0.0 (0.0) | 0.0 (0.5) | 0.0 (0.1) | 0.0 (0.1) | 0.0 (0.0) |
| Stikine_early timing | 0.0 (0.0) | 0.1 (0.2) | 0.0 (2.3) | 0.0 (2.4) | 0.0 (0.6) | 0.0 (0.2) | 0.0 (0.9) | 0.0 (3.0) |
| Stikine_late timing | 0.0 (0.0) | 0.1 (0.2) | 0.0 (0.1) | 0.0 (0.1) | 0.0 (0.0) | 0.0 (0.1) | 0.0 (0.2) | 0.0 (3.1) |
| Haida Gwaii-North | 0.0 (0.0) | 0.0 (0.0) | 0.0 (0.9) | 0.0 (0.0) | 0.0 (0.2) | 0.0 (0.0) | 0.0 (1.6) | 0.0 (1.7) |
| Upper Nass | 1.0 (0.8) | 0.0 (0.0) | 0.0 (4.5) | 0.0 (5.2) | 0.0 (0.1) | 0.0 (0.4) | 0.0 (2.0) | 0.0 (0.9) |
| Portland Sound-Observatory Inlet-Lower Nass | 0.0 (0.0) | 0.0 (0.0) | 0.0 (0.1) | 0.0 (1.4) | 0.0 (0.0) | 0.0 (0.4) | 0.0 (0.1) | 0.0 (0.3) |
| Ecstall | 0.0 (0.2) | 0.0 (0.0) | 0.0 (0.1) | 0.0 (0.0) | 0.0 (0.0) | 0.0 (0.0) | 0.0 (0.0) | 0.0 (0.0) |
| Skeena Estuary | 0.0 (0.1) | 0.0 (0.0) | 0.0 (0.1) | 0.0 (0.1) | 0.0 (0.1) | 0.0 (0.0) | 0.0 (0.0) | 0.0 (0.0) |
| Lower Skeena | 0.0 (0.0) | 0.0 (0.1) | 0.0 (6.5) | 0.0 (0.2) | 0.0 (0.5) | 0.0 (4.0) | 0.0 (3.4) | 0.0 (0.2) |
| Kalum_early timing | 0.0 (0.0) | 0.0 (0.0) | 0.0 (2.9) | 0.0 (0.0) | 0.0 (0.0) | 0.0 (0.0) | 0.0 (0.3) | 0.0 (0.1) |
| Kalum_late timing | 0.0 (0.0) | 0.0 (0.0) | 0.0 (0.1) | 0.0 (0.6) | 0.0 (0.0) | 0.0 (0.0) | 0.0 (0.0) | 0.0 (1.2) |
| Zymoetz | 0.0 (0.0) | 0.0 (0.0) | 0.0 (0.0) | 0.0 (0.0) | 0.0 (0.0) | 0.0 (0.3) | 0.0 (0.0) | 0.0 (0.5) |
| Sicintine | 0.0 (0.0) | 0.0 (0.0) | 0.0 (0.0) | 0.0 (0.0) | 0.0 (0.0) | 0.0 (0.0) | 0.0 (0.0) | 0.0 (0.0) |
| Middle Skeena-mainstem tributaries | 0.0 (0.0) | 0.0 (0.1) | 0.0 (2.4) | 0.0 (7.5) | 0.0 (0.1) | 0.0 (0.2) | 0.0 (1.0) | 0.0 (0.2) |
| Middle Skeena-large lakes | 0.0 (0.0) | 0.0 (0.1) | 0.0 (1.4) | 0.0 (2.3) | 0.0 (0.3) | 0.0 (0.4) | 0.0 (0.1) | 0.0 (0.1) |
| Upper Skeena | 0.0 (0.2) | 0.3 (0.4) | 0.0 (0.1) | 0.0 (1.1) | 0.0 (0.2) | 0.0 (0.1) | 0.0 (0.2) | 0.0 (0.1) |
| Upper Bulkley River | 0.0 (0.0) | 0.0 (0.0) | 0.0 (0.0) | 0.0 (0.0) | 0.0 (0.0) | 0.0 (0.0) | 0.0 (0.0) | 0.0 (0.0) |
| North and Central Coast-late timing | 2.0 (1.4) | 0.7 (0.5) | 0.0 (0.3) | 0.0 (4.1) | 0.0 (1.1) | 0.0 (0.0) | 0.0 (0.5) | 0.0 (0.0) |
| North and Central Coast-early timing | 0.0 (0.2) | 0.0 (0.0) | 0.0 (0.2) | 0.0 (0.8) | 0.0 (0.0) | 0.0 (0.0) | 0.0 (0.0) | 0.0 (0.0) |
| Rivers Inlet | 0.0 (0.1) | 0.0 (0.0) | 0.0 (0.0) | 0.0 (0.1) | 0.0 (0.1) | 0.0 (0.1) | 0.0 (0.0) | 0.0 (0.2) |
| Wannock | 0.0 (0.0) | 0.0 (0.0) | 0.0 (0.6) | 0.0 (0.0) | 0.0 (0.0) | 0.0 (0.0) | 0.0 (0.0) | 0.0 (0.0) |
| Bella Coola-Bentinck | 0.0 (0.4) | 0.0 (0.0) | 0.0 (0.0) | 0.0 (16.8) | 0.0 (0.1) | 0.0 (0.1) | 0.0 (1.3) | 0.0 (4.4) |
| Dean River | 0.0 (0.0) | 0.0 (0.0) | 0.0 (0.0) | 0.0 (0.7) | 0.0 (0.2) | 0.0 (0.0) | 0.0 (0.0) | 0.0 (0.0) |
| Docee | 0.0 (0.0) | 0.0 (0.0) | 0.0 (0.5) | 0.0 (0.0) | 0.0 (0.0) | 0.0 (0.0) | 0.0 (0.0) | 0.0 (0.3) |
| Klinaklini_SU_1.3 | 0.0 (0.0) | 0.0 (0.0) | 0.0 (0.0) | 0.0 (0.1) | 0.0 (0.0) | 11.1 (10.9) | 0.0 (0.0) | 0.0 (0.8) |
| Southern Mainland-Southern Fjords_FA_0.x | 2.0 (1.8) | 0.3 (0.3) | 0.0 (0.1) | 50.0 (24.3) | 0.0 (0.0) | 0.0 (0.0) | 0.0 (2.0) | 0.0 (0.0) |
| Southern Mainland-Georgia Strait_FA_0.x | 2.0 (1.3) | 0.0 (0.0) | 0.0 (1.9) | 0.0 (8.7) | 2.8 (2.7) | 0.0 (0.4) | 0.0 (3.0) | 0.0 (1.6) |
| Upper Fraser River_SP_1.3 | 0.0 (0.2) | 0.0 (0.1) | 0.0 (4.7) | 0.0 (1.5) | 0.0 (0.4) | 0.0 (3.9) | 0.0 (3.9) | 0.0 (1.0) |
| Middle Fraser River_SU_1.3 | 0.0 (0.2) | 0.0 (0.0) | 0.0 (0.9) | 0.0 (0.8) | 0.0 (0.9) | 0.0 (0.4) | 0.0 (2.4) | 0.0 (0.2) |
| Middle Fraser River_SP_1.3 | 0.0 (0.2) | 0.0 (0.0) | 0.0 (2.1) | 0.0 (2.2) | 0.0 (0.3) | 0.0 (0.7) | 0.0 (0.8) | 0.0 (0.5) |
| Middle Fraser River-Portage_FA_1.3 | 0.0 (0.0) | 0.0 (0.0) | 0.0 (0.0) | 0.0 (0.0) | 0.0 (0.0) | 0.0 (0.9) | 0.0 (0.1) | 0.0 (0.3) |
| Middle Fraser-Fraser Canyon_SP_1.3 | 0.0 (0.0) | 0.0 (0.0) | 0.0 (0.0) | 0.0 (4.3) | 0.0 (1.5) | 0.0 (0.0) | 0.0 (0.1) | 0.0 (0.0) |
| North Thompson_SP_1.3 | 0.0 (0.1) | 0.0 (0.1) | 0.0 (0.2) | 0.0 (0.4) | 0.0 (0.0) | 0.0 (0.1) | 0.0 (0.3) | 0.0 (0.1) |
| North Thompson_SU_1.3 | 1.0 (0.7) | 0.0 (0.0) | 0.0 (0.1) | 0.0 (0.9) | 0.0 (0.0) | 0.0 (0.4) | 0.0 (0.6) | 0.0 (1.6) |
| Shuswap River_SU_0.3 | 5.0 (1.9) | 3.6 (1.2) | 0.0 (1.5) | 0.0 (0.1) | 5.6 (4.1) | 0.0 (0.1) | 0.0 (0.1) | 0.0 (0.0) |
| South Thompson-Bessette Creek_SU_1.2 | 0.0 (0.0) | 0.0 (0.0) | 0.0 (0.0) | 0.0 (3.6) | 0.0 (0.0) | 0.0 (0.4) | 0.0 (5.2) | 0.0 (0.0) |
| South Thompson_SU_0.3 | 23.0 (3.9) | 29.8 (2.6) | 20.0 (15.2) | 0.0 (1.6) | 0.0 (0.0) | 0.0 (1.0) | 0.0 (0.3) | 12.5 (10.9) |
| South Thompson_SU_1.3 | 0.0 (0.0) | 0.0 (0.0) | 0.0 (1.7) | 0.0 (2.7) | 0.0 (0.0) | 0.0 (0.5) | 0.0 (1.2) | 0.0 (0.0) |
| Lower Thompson_SP_1.2 | 0.0 (0.1) | 0.0 (0.0) | 0.0 (1.4) | 0.0 (3.9) | 0.0 (0.4) | 0.0 (0.1) | 0.0 (0.1) | 0.0 (1.5) |
| Lower Fraser River_SP_1.3 | 0.0 (0.0) | 0.0 (0.0) | 0.0 (0.2) | 0.0 (3.5) | 0.0 (0.0) | 0.0 (0.0) | 0.0 (0.0) | 0.0 (0.0) |
| Lower Fraser River_SU_1.3 | 0.0 (0.0) | 0.3 (0.4) | 0.0 (0.5) | 0.0 (0.1) | 0.0 (0.1) | 0.0 (0.7) | 0.0 (0.1) | 0.0 (0.0) |
| Lower Fraser River-Upper Pitt_SU_1.3 | 2.0 (1.4) | 0.7 (0.4) | 0.0 (0.0) | 0.0 (0.2) | 0.0 (0.1) | 0.0 (0.0) | 0.0 (0.4) | 0.0 (0.1) |
| Maria Slough_SU_0.3 | 0.0 (0.0) | 0.0 (0.0) | 0.0 (0.0) | 0.0 (0.3) | 0.0 (0.1) | 0.0 (0.1) | 0.0 (0.0) | 0.0 (0.0) |
| Lower Fraser River_FA_0.3 | 4.0 (2.6) | 3.1 (0.8) | 0.0 (0.4) | 0.0 (0.2) | 58.3 (8.5) | 44.4 (13.9) | 25.0 (20.1) | 25.0 (12.2) |
| East Vancouver Island-North_FA_0.x | 3.0 (1.8) | 7.3 (1.2) | 0.0 (0.1) | 0.0 (7.0) | 0.0 (0.2) | 0.0 (0.4) | 0.0 (0.0) | 0.0 (0.7) |
| East Vancouver Island-Qualicum and Puntledge_FA_0.x | 7.7 (3.0) | 14.2 (2.0) | 20.0 (14.1) | 0.0 (1.7) | 0.0 (0.9) | 0.0 (0.1) | 23.0 (14.9) | 12.5 (12.1) |
| East Vancouver Island-Nanaimo and Chemainus_FA_0.x | 0.0 (0.0) | 0.0 (0.0) | 0.0 (0.0) | 0.0 (0.3) | 0.0 (0.0) | 0.0 (0.0) | 0.0 (4.2) | 0.0 (0.4) |
| East Vancouver Island-Nanaimo_SP_1.x | 0.0 (0.0) | 0.0 (0.0) | 0.0 (0.0) | 0.0 (2.4) | 0.0 (0.0) | 0.0 (0.0) | 0.0 (1.7) | 0.0 (0.0) |
| East Vancouver Island-Georgia Strait_SU_0.3 | 0.0 (0.0) | 0.0 (0.1) | 0.0 (0.6) | 0.0 (0.0) | 0.0 (0.0) | 0.0 (0.3) | 0.0 (2.4) | 0.0 (0.0) |
| East Vancouver Island-Cowichan and Koksilah_FA_0.x | 2.3 (1.7) | 1.5 (0.7) | 0.0 (0.0) | 0.0 (0.0) | 0.0 (0.0) | 0.0 (0.0) | 0.0 (0.0) | 0.0 (0.5) |
| West Vancouver Island-Nootka and Kyuquot_FA_0.x | 5.0 (1.9) | 3.9 (1.1) | 0.0 (0.6) | 0.0 (3.5) | 0.0 (0.4) | 0.0 (1.0) | 0.0 (0.2) | 0.0 (3.7) |
| West Vancouver Island-North_FA_0.x | 0.0 (0.2) | 0.0 (0.0) | 0.0 (0.3) | 0.0 (2.8) | 0.0 (0.0) | 0.0 (0.0) | 0.0 (0.3) | 0.0 (1.8) |
| West Vancouver Island-South_FA_0.x | 15.0 (3.3) | 23.0 (2.4) | 0.0 (1.8) | 0.0 (1.2) | 5.3 (3.4) | 0.0 (0.6) | 25.0 (17.4) | 12.5 (7.0) |
| Okanagan_1.x | 0.0 (0.1) | 0.0 (0.0) | 1.3 (0.4) | 0.0 (3.8) | 0.0 (0.0) | 0.0 (0.0) | 0.0 (0.3) | 0.0 (0.0) |
| Juan de Fuca | 2.0 (1.8) | 0.3 (0.4) | 0.0 (0.0) | 0.0 (0.0) | 0.0 (0.0) | 0.0 (0.2) | 0.0 (0.0) | 0.0 (0.0) |
| Coastal Washington | 0.0 (0.1) | 0.0 (0.0) | 0.0 (1.8) | 0.0 (3.0) | 0.0 (0.1) | 11.1 (10.8) | 0.0 (0.2) | 0.0 (0.3) |
| North Puget Sound | 5.9 (2.4) | 1.9 (0.4) | 13.5 (13.0) | 50.0 (24.6) | 15.4 (4.9) | 11.1 (9.1) | 0.8 (6.1) | 12.5 (0.7) |
| South Puget Sound | 12.1 (3.3) | 4.5 (1.1) | 26.5 (17.4) | 0.0 (0.4) | 5.6 (2.7) | 22.2 (12.4) | 26.2 (18.8) | 25.0 (13.2) |
| Lower Columbia River | 0.0 (0.0) | 0.3 (0.4) | 0.0 (9.0) | 0.0 (0.0) | 0.0 (0.0) | 0.0 (0.0) | 0.0 (0.1) | 0.0 (0.4) |
| Mid Columbia River_SP | 0.0 (0.3) | 0.9 (0.5) | 0.1 (1.7) | 0.0 (2.0) | 6.0 (5.1) | 0.0 (2.0) | 0.0 (3.6) | 0.0 (0.5) |
| Upper Columbia River_SP | 0.0 (0.0) | 0.0 (0.1) | 0.0 (0.0) | 0.0 (1.4) | 0.0 (0.5) | 0.0 (0.1) | 0.0 (1.9) | 0.0 (0.2) |
| Upper Columbia River_SU_FA | 3.0 (1.8) | 2.4 (0.9) | 18.7 (14.4) | 0.0 (0.0) | 0.0 (0.1) | 0.0 (0.3) | 0.0 (2.6) | 0.0 (2.8) |
| Snake River_FA | 2.0 (1.5) | 0.0 (0.0) | 0.0 (0.0) | 0.0 (0.0) | 0.0 (0.0) | 0.0 (1.3) | 0.0 (0.0) | 0.0 (0.1) |
| Snake River_SP_SU | 0.0 (0.3) | 0.0 (0.2) | 0.0 (2.7) | 0.0 (5.1) | 0.0 (0.6) | 0.0 (0.9) | 0.0 (3.0) | 0.0 (0.8) |
| North & Central Oregon | 0.0 (0.3) | 0.0 (0.0) | 0.0 (1.1) | 0.0 (4.1) | 0.0 (0.5) | 0.0 (0.2) | 0.0 (1.6) | 0.0 (0.0) |
| Upper Willamette River | 0.0 (0.0) | 0.0 (0.0) | 0.0 (0.4) | 0.0 (1.0) | 0.0 (0.0) | 0.0 (0.5) | 0.0 (0.2) | 0.0 (0.9) |
| South Oregon coastal | 0.0 (0.1) | 0.0 (0.0) | 0.0 (0.8) | 0.0 (0.7) | 0.0 (0.2) | 0.0 (0.0) | 0.0 (1.2) | 0.0 (0.5) |
| California Klamath Trinity | 0.0 (0.0) | 0.0 (0.0) | 0.0 (0.7) | 0.0 (0.1) | 0.0 (0.1) | 0.0 (1.4) | 0.0 (0.1) | 0.0 (1.0) |
| California Central Valley_Fall | 0.0 (0.0) | 0.1 (0.2) | 0.0 (1.1) | 0.0 (3.0) | 0.0 (0.1) | 0.0 (0.4) | 0.0 (1.8) | 0.0 (0.5) |
| California Central Valley_Spring | 0.0 (0.5) | 0.3 (0.3) | 0.0 (2.3) | 0.0 (4.8) | 0.0 (0.2) | 0.0 (0.1) | 0.0 (0.0) | 0.0 (2.1) |
| Coastal California | 0.0 (0.0) | 0.0 (0.0) | 0.0 (0.2) | 0.0 (0.0) | 0.0 (0.0) | 0.0 (0.0) | 0.0 (0.0) | 0.0 (1.0) |

Supplementary Table S5 continued

| Conservation Unit | Mquqwin test fishery | | | | Capilano derby | Strait of Georgia-north sport | | | | |
| --- | --- | --- | --- | --- | --- | --- | --- | --- | --- | --- |
|  | July 20-23 | July 26-29 | Aug 18-21 | Aug 24-27 | July | January | | February | | |
|  |  |  |  |  |  | Legal | Sublegal | Legal | Sublegal | Umknown |
| Sample size | 106 | 58 | 95 | 73 | 52 | 33 | 47 | 9 | 18 | 1 |
| N PBT | 13 | 10 | 24 | 25 | 39 | 3 | 23 | 1 | 7 | 1 |
| Southeast Alaska | 0.0 (0.0) | 0.0 (0.1) | 0.0 (0.2) | 0.0 (0.0) | 0.0 (0.0) | 0.0 (0.0) | 0.0 (0.0) | 0.0 (0.4) | 0.0 (0.1) | 0.0 (7.3) |
| Alsek | 0.0 (0.0) | 0.0 (0.0) | 0.0 (0.0) | 0.0 (0.1) | 0.0 (0.1) | 0.0 (0.3) | 0.0 (0.0) | 0.0 (0.5) | 0.0 (0.4) | 0.0 (5.0) |
| Unuk | 0.0 (0.0) | 0.0 (0.0) | 0.0 (0.0) | 0.0 (0.0) | 0.0 (0.0) | 0.0 (0.0) | 0.0 (0.0) | 0.0 (0.0) | 0.0 (0.0) | 0.0 (0.0) |
| Taku_early timing | 0.0 (0.0) | 0.0 (0.4) | 0.0 (0.3) | 0.0 (0.0) | 0.0 (0.0) | 0.0 (0.1) | 0.0 (0.0) | 0.0 (5.4) | 0.0 (0.0) | 0.0 (4.1) |
| Taku_mid timing | 0.0 (0.2) | 0.0 (0.1) | 0.0 (0.0) | 0.0 (0.1) | 0.0 (0.0) | 0.0 (0.1) | 0.0 (0.0) | 0.0 (0.9) | 0.0 (0.6) | 0.0 (12.5) |
| Taku_late timing | 0.0 (0.1) | 0.0 (0.1) | 0.0 (0.0) | 0.0 (0.0) | 0.0 (0.0) | 0.0 (0.2) | 0.0 (0.0) | 0.0 (0.3) | 0.0 (0.4) | 0.0 (10.7) |
| Stikine_early timing | 0.0 (0.1) | 0.0 (0.3) | 0.0 (0.0) | 0.0 (0.0) | 0.0 (0.3) | 0.0 (0.1) | 0.0 (0.1) | 0.0 (0.7) | 0.0 (0.2) | 0.0 (0.0) |
| Stikine_late timing | 0.0 (0.0) | 0.0 (0.2) | 0.0 (0.1) | 0.0 (0.1) | 0.0 (0.3) | 0.0 (0.0) | 0.0 (0.0) | 0.0 (0.1) | 0.0 (0.2) | 0.0 (0.9) |
| Haida Gwaii-North | 0.0 (0.0) | 0.0 (0.4) | 0.0 (0.0) | 0.0 (0.0) | 0.0 (0.3) | 0.0 (0.0) | 0.0 (0.0) | 0.0 (0.0) | 0.0 (0.2) | 0.0 (0.0) |
| Upper Nass | 0.0 (0.1) | 0.0 (0.1) | 0.0 (0.3) | 0.0 (0.3) | 0.0 (0.0) | 0.0 (0.8) | 0.0 (0.1) | 0.0 (0.0) | 0.0 (0.1) | 0.0 (1.0) |
| Portland Sound-Observatory Inlet-Lower Nass | 0.0 (0.0) | 0.0 (0.1) | 0.0 (0.2) | 0.0 (0.1) | 0.0 (0.2) | 0.0 (0.9) | 0.0 (0.1) | 0.0 (0.2) | 0.0 (0.0) | 0.0 (0.0) |
| Ecstall | 0.0 (0.0) | 0.0 (0.0) | 0.0 (0.0) | 0.0 (0.0) | 0.0 (0.0) | 0.0 (0.0) | 0.0 (0.0) | 0.0 (0.0) | 0.0 (0.1) | 0.0 (0.1) |
| Skeena Estuary | 0.0 (0.0) | 0.0 (0.0) | 0.0 (0.0) | 0.0 (0.0) | 0.0 (0.0) | 0.0 (0.0) | 0.0 (0.0) | 0.0 (0.0) | 0.0 (0.1) | 0.0 (0.0) |
| Lower Skeena | 0.0 (0.2) | 0.0 (0.2) | 0.0 (0.0) | 0.0 (0.0) | 0.0 (0.2) | 0.0 (0.9) | 0.0 (0.3) | 0.0 (0.2) | 0.0 (0.5) | 0.0 (2.0) |
| Kalum_early timing | 0.0 (0.0) | 0.0 (0.0) | 0.0 (0.0) | 0.0 (0.1) | 0.0 (0.0) | 0.0 (0.0) | 0.0 (0.0) | 0.0 (0.1) | 0.0 (0.0) | 0.0 (0.1) |
| Kalum_late timing | 0.0 (0.0) | 0.0 (0.0) | 0.0 (0.0) | 0.0 (0.0) | 0.0 (0.0) | 0.0 (0.3) | 0.0 (0.0) | 0.0 (0.0) | 0.0 (0.0) | 0.0 (0.0) |
| Zymoetz | 0.0 (0.0) | 0.0 (0.0) | 0.0 (0.0) | 0.0 (0.0) | 0.0 (0.0) | 0.0 (0.0) | 0.0 (0.2) | 0.0 (0.3) | 0.0 (0.1) | 0.0 (0.0) |
| Sicintine | 0.0 (0.0) | 0.0 (0.0) | 0.0 (0.0) | 0.0 (0.0) | 0.0 (0.4) | 0.0 (0.0) | 0.0 (0.0) | 0.0 (0.0) | 0.0 (0.0) | 0.0 (0.0) |
| Middle Skeena-mainstem tributaries | 0.0 (0.0) | 0.0 (0.2) | 0.0 (0.1) | 0.0 (0.0) | 0.0 (0.5) | 0.0 (0.2) | 0.0 (0.5) | 0.0 (1.1) | 0.0 (0.3) | 0.0 (0.8) |
| Middle Skeena-large lakes | 0.0 (0.0) | 0.0 (0.1) | 0.0 (0.0) | 0.0 (0.0) | 0.0 (0.0) | 0.0 (0.0) | 0.0 (0.0) | 0.0 (0.8) | 0.0 (0.4) | 0.0 (0.3) |
| Upper Skeena | 0.0 (0.0) | 0.0 (0.0) | 0.0 (0.3) | 0.0 (0.1) | 0.0 (0.0) | 0.0 (0.0) | 0.0 (0.0) | 0.0 (0.1) | 0.0 (0.8) | 0.0 (0.3) |
| Upper Bulkley River | 0.0 (0.0) | 0.0 (0.0) | 0.0 (0.0) | 0.0 (0.0) | 0.0 (0.1) | 0.0 (0.0) | 0.0 (0.0) | 0.0 (0.0) | 0.0 (0.0) | 0.0 (0.0) |
| North and Central Coast-late timing | 0.9 (0.7) | 0.0 (0.0) | 3.1 (1.7) | 2.7 (1.7) | 0.0 (0.3) | 0.0 (0.0) | 0.0 (0.0) | 0.0 (0.1) | 0.0 (0.0) | 0.0 (0.1) |
| North and Central Coast-early timing | 0.0 (0.0) | 0.0 (0.1) | 0.0 (0.0) | 0.0 (0.1) | 0.0 (0.1) | 0.0 (0.0) | 0.0 (0.0) | 0.0 (0.0) | 0.0 (0.0) | 0.0 (0.0) |
| Rivers Inlet | 0.0 (0.0) | 0.0 (0.2) | 0.0 (0.0) | 0.0 (0.0) | 0.0 (0.0) | 0.0 (0.4) | 0.0 (0.1) | 0.0 (0.0) | 0.0 (0.0) | 0.0 (7.4) |
| Wannock | 0.0 (0.0) | 0.0 (0.0) | 0.0 (0.0) | 0.0 (0.0) | 0.0 (0.0) | 0.0 (0.0) | 0.0 (0.0) | 0.0 (0.0) | 0.0 (0.3) | 0.0 (0.4) |
| Bella Coola-Bentinck | 0.0 (0.0) | 1.7 (1.3) | 0.0 (0.0) | 0.0 (0.2) | 0.0 (0.0) | 0.0 (0.0) | 0.0 (0.3) | 0.0 (0.1) | 0.0 (1.5) | 0.0 (0.2) |
| Dean River | 0.0 (0.0) | 0.0 (0.0) | 0.0 (0.2) | 0.0 (0.0) | 0.0 (0.1) | 0.0 (0.1) | 0.0 (0.4) | 0.0 (0.0) | 0.0 (0.5) | 0.0 (0.9) |
| Docee | 0.0 (0.2) | 0.0 (0.0) | 0.0 (0.0) | 0.0 (0.0) | 0.0 (0.0) | 0.0 (0.0) | 0.0 (0.0) | 0.0 (0.8) | 0.0 (0.3) | 0.0 (0.0) |
| Klinaklini_SU_1.3 | 0.0 (0.0) | 0.0 (0.0) | 0.0 (0.0) | 0.0 (0.0) | 0.0 (0.0) | 0.0 (0.1) | 0.0 (0.0) | 0.0 (0.0) | 0.0 (0.0) | 0.0 (0.1) |
| Southern Mainland-Southern Fjords_FA_0.x | 0.0 (0.0) | 0.0 (0.0) | 0.0 (0.0) | 0.0 (0.2) | 0.0 (0.0) | 0.0 (0.1) | 0.0 (0.0) | 0.0 (0.1) | 0.0 (0.0) | 0.0 (0.0) |
| Southern Mainland-Georgia Strait_FA_0.x | 0.0 (0.1) | 0.0 (0.0) | 0.0 (0.2) | 0.0 (0.1) | 0.0 (0.0) | 0.0 (0.0) | 0.0 (0.7) | 0.4 (2.8) | 0.0 (1.0) | 0.0 (2.8) |
| Upper Fraser River_SP_1.3 | 0.0 (0.1) | 0.0 (0.4) | 0.0 (0.0) | 0.0 (0.5) | 0.0 (0.3) | 0.0 (0.2) | 2.1 (1.9) | 0.0 (2.7) | 0.0 (1.6) | 0.0 (4.7) |
| Middle Fraser River_SU_1.3 | 0.9 (0.7) | 0.0 (0.2) | 0.0 (0.2) | 0.0 (0.2) | 0.0 (0.0) | 0.0 (0.2) | 0.0 (0.5) | 0.0 (0.5) | 0.0 (0.6) | 0.0 (3.4) |
| Middle Fraser River_SP_1.3 | 0.0 (0.0) | 0.0 (0.2) | 0.0 (0.2) | 0.0 (0.1) | 0.0 (0.2) | 0.0 (0.6) | 0.0 (0.1) | 0.0 (1.1) | 0.0 (0.1) | 0.0 (9.6) |
| Middle Fraser River-Portage_FA_1.3 | 0.0 (0.0) | 0.0 (0.0) | 0.0 (0.0) | 0.0 (0.0) | 0.0 (0.0) | 0.0 (0.0) | 0.0 (0.0) | 0.0 (0.0) | 0.0 (0.1) | 0.0 (0.0) |
| Middle Fraser-Fraser Canyon_SP_1.3 | 0.0 (0.0) | 0.0 (0.0) | 0.0 (0.0) | 0.0 (0.0) | 0.0 (0.0) | 0.0 (0.0) | 0.0 (0.0) | 0.0 (0.0) | 0.0 (0.0) | 0.0 (0.0) |
| North Thompson_SP_1.3 | 0.0 (0.1) | 0.0 (0.0) | 0.0 (0.0) | 0.0 (0.2) | 0.0 (0.0) | 0.0 (0.1) | 0.0 (0.1) | 0.0 (0.5) | 0.0 (0.0) | 0.0 (0.4) |
| North Thompson_SU_1.3 | 0.0 (0.0) | 0.0 (0.0) | 0.0 (0.0) | 0.0 (0.1) | 0.0 (0.2) | 0.0 (0.0) | 0.0 (0.1) | 0.0 (0.7) | 0.0 (0.8) | 0.0 (3.5) |
| Shuswap River_SU_0.3 | 0.0 (0.0) | 5.2 (2.2) | 0.0 (0.0) | 1.4 (1.6) | 0.0 (0.0) | 0.0 (0.0) | 0.0 (0.0) | 0.0 (0.0) | 0.0 (0.0) | 0.0 (2.0) |
| South Thompson-Bessette Creek_SU_1.2 | 0.0 (0.0) | 0.0 (0.0) | 0.0 (0.0) | 0.0 (0.0) | 0.0 (0.0) | 0.0 (0.0) | 0.0 (0.0) | 0.0 (0.1) | 0.0 (0.0) | 0.0 (3.8) |
| South Thompson_SU_0.3 | 14.2 (3.5) | 6.9 (3.5) | 30.5 (4.1) | 16.4 (4.1) | 3.8 (2.1) | 0.0 (0.0) | 2.1 (2.2) | 0.0 (0.5) | 0.0 (0.3) | 0.0 (7.7) |
| South Thompson_SU_1.3 | 0.0 (0.0) | 0.0 (0.0) | 0.0 (0.0) | 0.0 (0.1) | 0.0 (0.4) | 0.0 (0.0) | 0.0 (0.2) | 0.0 (2.2) | 0.0 (0.1) | 0.0 (7.7) |
| Lower Thompson_SP_1.2 | 0.0 (0.2) | 0.0 (0.2) | 0.0 (0.0) | 0.0 (0.3) | 0.0 (0.1) | 0.0 (0.2) | 0.0 (0.1) | 0.0 (0.0) | 0.0 (2.3) | 0.0 (1.7) |
| Lower Fraser River_SP_1.3 | 0.0 (0.0) | 0.0 (0.0) | 0.0 (0.0) | 0.0 (0.0) | 0.0 (0.1) | 0.0 (0.0) | 0.0 (0.0) | 0.0 (0.0) | 0.0 (0.0) | 0.0 (0.1) |
| Lower Fraser River_SU_1.3 | 0.0 (0.0) | 0.0 (0.2) | 0.0 (0.2) | 0.0 (0.0) | 0.0 (0.0) | 0.0 (0.0) | 0.0 (0.2) | 0.0 (0.0) | 0.0 (0.0) | 0.0 (0.8) |
| Lower Fraser River-Upper Pitt_SU_1.3 | 0.0 (0.0) | 0.0 (0.0) | 0.0 (0.0) | 0.0 (0.0) | 0.0 (0.6) | 0.0 (0.1) | 0.0 (0.1) | 0.0 (0.0) | 0.0 (0.2) | 0.0 (2.4) |
| Maria Slough_SU_0.3 | 0.0 (0.0) | 0.0 (0.0) | 0.0 (0.0) | 0.0 (0.0) | 0.0 (0.5) | 0.0 (0.0) | 0.0 (0.0) | 0.0 (0.0) | 0.0 (0.1) | 0.0 (0.0) |
| Lower Fraser River_FA_0.3 | 0.0 (0.0) | 0.0 (0.1) | 0.0 (0.1) | 0.0 (0.2) | 96.2 (2.7) | 3.0 (3.3) | 2.1 (1.8) | 0.0 (0.3) | 0.0 (0.2) | 0.0 (3.1) |
| East Vancouver Island-North_FA_0.x | 0.0 (0.1) | 0.0 (0.4) | 0.0 (0.0) | 1.4 (1.1) | 0.0 (0.7) | 0.0 (0.1) | 6.4 (4.1) | 0.0 (2.7) | 0.0 (0.1) | 0.0 (0.7) |
| East Vancouver Island-Qualicum and Puntledge_FA_0.x | 0.0 (0.1) | 0.0 (0.0) | 2.0 (1.1) | 0.1 (0.2) | 0.0 (0.0) | 10.9 (5.6) | 60.5 (6.8) | 21.3 (10.4) | 80.7 (12.1) | 100.0 (28.7) |
| East Vancouver Island-Nanaimo and Chemainus_FA_0.x | 0.0 (0.0) | 0.0 (0.1) | 0.0 (0.2) | 0.0 (0.0) | 0.0 (0.0) | 0.0 (0.3) | 0.0 (0.0) | 0.0 (0.1) | 0.0 (0.1) | 0.0 (1.0) |
| East Vancouver Island-Nanaimo_SP_1.x | 0.0 (0.0) | 0.0 (0.0) | 0.0 (0.0) | 0.0 (0.0) | 0.0 (0.0) | 0.0 (0.0) | 0.0 (0.1) | 0.0 (4.6) | 0.0 (0.0) | 0.0 (2.1) |
| East Vancouver Island-Georgia Strait_SU_0.3 | 0.0 (0.0) | 0.0 (0.1) | 0.0 (0.0) | 0.0 (0.0) | 0.0 (0.0) | 3.0 (2.4) | 6.4 (4.0) | 11.1 (8.0) | 11.1 (6.3) | 0.0 (9.1) |
| East Vancouver Island-Cowichan and Koksilah_FA_0.x | 0.0 (0.0) | 0.0 (0.3) | 0.0 (0.0) | 1.3 (1.5) | 0.0 (0.0) | 58.8 (7.7) | 7.5 (4.1) | 33.9 (13.6) | 2.6 (6.4) | 0.0 (0.0) |
| West Vancouver Island-Nootka and Kyuquot_FA_0.x | 35.8 (4.4) | 39.5 (6.6) | 2.1 (1.3) | 0.0 (0.4) | 0.0 (0.3) | 0.0 (0.1) | 0.0 (0.0) | 0.0 (1.1) | 0.0 (0.4) | 0.0 (6.6) |
| West Vancouver Island-North_FA_0.x | 0.0 (0.0) | 0.0 (0.0) | 0.0 (0.0) | 0.0 (0.0) | 0.0 (0.1) | 0.0 (0.0) | 0.0 (0.0) | 0.0 (0.0) | 0.0 (0.1) | 0.0 (0.4) |
| West Vancouver Island-South_FA_0.x | 17.9 (4.2) | 29.5 (6.2) | 33.7 (4.8) | 56.1 (5.4) | 0.0 (0.5) | 0.0 (1.5) | 0.0 (0.2) | 0.0 (1.2) | 0.0 (2.3) | 0.0 (2.2) |
| Okanagan_1.x | 0.1 (0.5) | 0.0 (0.0) | 0.0 (0.0) | 0.0 (0.0) | 0.0 (0.0) | 0.0 (0.3) | 0.0 (0.0) | 0.0 (0.2) | 0.0 (0.0) | 0.0 (0.0) |
| Juan de Fuca | 0.0 (0.1) | 1.7 (1.0) | 0.0 (0.0) | 0.0 (0.0) | 0.0 (0.0) | 0.0 (0.0) | 0.0 (0.0) | 0.0 (0.0) | 0.0 (1.4) | 0.0 (0.0) |
| Coastal Washington | 3.8 (1.6) | 0.0 (0.1) | 5.4 (2.4) | 4.2 (1.9) | 0.0 (0.1) | 0.0 (0.1) | 0.0 (0.1) | 0.0 (1.2) | 0.0 (0.1) | 0.0 (5.5) |
| North Puget Sound | 0.0 (0.1) | 1.7 (2.0) | 0.1 (0.2) | 0.0 (0.0) | 0.0 (0.3) | 17.1 (5.3) | 10.6 (3.4) | 11.1 (9.0) | 5.6 (5.6) | 0.0 (1.7) |
| South Puget Sound | 10.4 (2.6) | 6.9 (2.9) | 3.2 (2.0) | 2.7 (1.9) | 0.0 (0.0) | 6.4 (3.2) | 2.1 (2.1) | 22.3 (12.1) | 0.0 (0.3) | 0.0 (1.5) |
| Lower Columbia River | 7.5 (2.6) | 0.0 (0.1) | 3.0 (1.4) | 2.6 (1.4) | 0.0 (0.0) | 0.0 (0.0) | 0.0 (0.0) | 0.0 (0.1) | 0.0 (0.2) | 0.0 (1.0) |
| Mid Columbia River_SP | 0.0 (0.0) | 0.0 (0.0) | 0.0 (0.0) | 0.0 (0.0) | 0.0 (0.1) | 0.8 (1.7) | 0.0 (0.0) | 0.0 (0.2) | 0.0 (0.6) | 0.0 (1.1) |
| Upper Columbia River_SP | 0.0 (0.0) | 0.0 (0.0) | 0.0 (0.1) | 0.0 (0.0) | 0.0 (0.3) | 0.0 (0.6) | 0.0 (0.0) | 0.0 (0.1) | 0.0 (0.6) | 0.0 (0.0) |
| Upper Columbia River_SU_FA | 7.4 (2.5) | 5.2 (3.4) | 15.0 (3.8) | 11.0 (3.7) | 0.0 (0.1) | 0.0 (0.0) | 0.0 (0.1) | 0.0 (0.6) | 0.0 (0.0) | 0.0 (10.4) |
| Snake River_FA | 0.1 (0.2) | 0.0 (0.0) | 0.8 (1.3) | 0.0 (0.0) | 0.0 (0.0) | 0.0 (0.0) | 0.0 (0.0) | 0.0 (1.1) | 0.0 (0.0) | 0.0 (0.0) |
| Snake River_SP_SU | 0.0 (0.0) | 0.0 (0.1) | 0.0 (0.2) | 0.0 (0.1) | 0.0 (0.4) | 0.0 (2.0) | 0.0 (0.3) | 0.0 (1.1) | 0.0 (0.3) | 0.0 (3.5) |
| North & Central Oregon | 0.0 (0.0) | 0.0 (0.1) | 0.6 (0.9) | 0.0 (0.0) | 0.0 (0.4) | 0.0 (0.0) | 0.0 (0.4) | 0.0 (0.1) | 0.0 (0.3) | 0.0 (0.5) |
| Upper Willamette River | 0.0 (0.1) | 1.7 (1.4) | 0.0 (0.2) | 0.2 (0.4) | 0.0 (0.1) | 0.0 (0.0) | 0.0 (0.3) | 0.0 (0.0) | 0.0 (0.0) | 0.0 (0.8) |
| South Oregon coastal | 0.9 (0.8) | 0.0 (0.1) | 0.4 (0.6) | 0.0 (0.2) | 0.0 (0.1) | 0.0 (0.2) | 0.0 (0.1) | 0.0 (0.3) | 0.0 (0.4) | 0.0 (5.1) |
| California Klamath Trinity | 0.0 (0.0) | 0.0 (0.1) | 0.0 (0.0) | 0.0 (0.0) | 0.0 (0.1) | 0.0 (0.0) | 0.0 (0.1) | 0.0 (0.3) | 0.0 (1.4) | 0.0 (0.8) |
| California Central Valley_Fall | 0.0 (0.0) | 0.0 (0.1) | 0.0 (0.2) | 0.0 (0.3) | 0.0 (0.2) | 0.0 (0.3) | 0.0 (0.5) | 0.0 (1.1) | 0.0 (0.3) | 0.0 (4.6) |
| California Central Valley_Spring | 0.0 (0.1) | 0.0 (0.0) | 0.0 (0.0) | 0.0 (0.3) | 0.0 (0.1) | 0.0 (0.0) | 0.0 (0.2) | 0.0 (0.0) | 0.0 (0.2) | 0.0 (1.6) |
| Coastal California | 0.0 (0.0) | 0.0 (0.1) | 0.0 (0.0) | 0.0 (0.0) | 0.0 (0.0) | 0.0 (0.0) | 0.0 (0.1) | 0.0 (0.0) | 0.0 (1.4) | 0.0 (0.0) |

Supplementary Table S5 continued

| Conservation Unit | Strait of Georgia-north sport | | | | | | | | | |
| --- | --- | --- | --- | --- | --- | --- | --- | --- | --- | --- |
|  | March | | May | | June | | | July | | |
|  | Legal | Sublegal | Legal | Sublegal | Legal | Sublegal | Unknown | Legal | Sublegal | Unknown |
| Sample size | 6 | 19 | 10 | 1 | 207 | 45 | 3 | 591 | 48 | 49 |
| N PBT | 1 | 9 | 1 | 1 | 55 | 14 | 0 | 161 | 18 | 8 |
| Southeast Alaska | 0.0 (0.6) | 0.0 (0.2) | 0.0 (1.5) | 0.0 (7.2) | 0.0 (0.0) | 0.0 (0.1) | 0.0 (2.7) | 0.0 (0.0) | 0.0 (0.1) | 0.0 (0.0) |
| Alsek | 0.0 (1.9) | 0.0 (0.1) | 0.0 (1.5) | 0.0 (1.0) | 0.0 (0.1) | 0.0 (0.0) | 0.0 (2.9) | 0.0 (0.0) | 0.0 (0.0) | 0.0 (0.1) |
| Unuk | 0.0 (0.5) | 0.0 (0.1) | 0.0 (0.6) | 0.0 (0.0) | 0.0 (0.0) | 0.0 (0.0) | 0.0 (0.4) | 0.0 (0.0) | 0.0 (0.0) | 0.0 (0.0) |
| Taku_early timing | 0.0 (3.2) | 0.0 (0.0) | 0.0 (0.0) | 0.0 (0.8) | 0.0 (0.0) | 0.0 (0.0) | 0.0 (0.2) | 0.0 (0.0) | 0.0 (0.0) | 0.0 (0.4) |
| Taku_mid timing | 0.0 (2.0) | 0.0 (0.0) | 0.0 (0.1) | 0.0 (0.5) | 0.0 (0.0) | 0.0 (0.1) | 0.0 (1.0) | 0.0 (0.0) | 0.0 (0.0) | 0.0 (0.0) |
| Taku_late timing | 0.0 (0.0) | 0.0 (0.2) | 0.0 (2.2) | 0.0 (0.7) | 0.0 (0.0) | 0.0 (0.0) | 0.0 (0.0) | 0.0 (0.0) | 0.0 (0.0) | 0.0 (0.4) |
| Stikine_early timing | 0.0 (1.9) | 0.0 (0.4) | 0.0 (0.0) | 0.0 (4.4) | 0.0 (0.1) | 0.0 (0.0) | 0.0 (0.5) | 0.0 (0.0) | 0.0 (0.1) | 0.0 (0.1) |
| Stikine_late timing | 0.0 (0.0) | 0.0 (0.3) | 0.0 (0.0) | 0.0 (0.0) | 0.0 (0.0) | 0.0 (0.2) | 0.0 (0.0) | 0.0 (0.0) | 0.0 (0.3) | 0.0 (0.0) |
| Haida Gwaii-North | 0.0 (0.0) | 0.0 (0.0) | 0.0 (0.0) | 0.0 (0.0) | 0.0 (0.0) | 0.0 (0.0) | 0.0 (0.1) | 0.0 (0.0) | 0.0 (0.0) | 0.0 (0.0) |
| Upper Nass | 0.0 (1.2) | 0.0 (2.0) | 0.0 (0.9) | 0.0 (3.6) | 0.0 (0.1) | 0.0 (0.2) | 0.0 (5.1) | 0.0 (0.1) | 0.0 (0.8) | 0.0 (0.2) |
| Portland Sound-Observatory Inlet-Lower Nass | 0.0 (1.3) | 0.0 (0.3) | 0.0 (0.4) | 0.0 (0.2) | 0.0 (0.0) | 0.0 (0.0) | 0.0 (0.6) | 0.0 (0.0) | 0.0 (0.2) | 0.0 (0.0) |
| Ecstall | 0.0 (0.0) | 0.0 (0.3) | 0.0 (0.0) | 0.0 (0.8) | 0.0 (0.0) | 0.0 (0.0) | 0.0 (8.3) | 0.0 (0.0) | 0.0 (0.0) | 0.0 (0.0) |
| Skeena Estuary | 0.0 (0.0) | 0.0 (0.0) | 0.0 (0.0) | 0.0 (0.0) | 0.0 (0.0) | 0.0 (0.0) | 0.0 (0.0) | 0.0 (0.0) | 0.0 (0.0) | 0.0 (0.1) |
| Lower Skeena | 0.0 (1.2) | 0.0 (0.0) | 0.0 (1.3) | 0.0 (3.3) | 0.0 (0.1) | 0.0 (0.4) | 0.0 (3.7) | 0.0 (0.0) | 0.0 (0.2) | 0.0 (0.2) |
| Kalum_early timing | 0.0 (0.7) | 0.0 (0.0) | 0.0 (0.0) | 0.0 (0.0) | 0.0 (0.0) | 0.0 (0.0) | 0.0 (1.1) | 0.0 (0.0) | 0.0 (0.0) | 0.0 (0.0) |
| Kalum_late timing | 0.0 (0.2) | 0.0 (0.0) | 0.0 (0.0) | 0.0 (0.7) | 0.0 (0.0) | 0.0 (0.0) | 0.0 (0.0) | 0.0 (0.0) | 0.0 (0.1) | 0.0 (0.0) |
| Zymoetz | 0.0 (0.0) | 0.0 (0.0) | 0.0 (0.0) | 0.0 (0.0) | 0.0 (0.0) | 0.0 (0.0) | 0.0 (0.0) | 0.0 (0.0) | 0.0 (0.0) | 0.0 (0.2) |
| Sicintine | 0.0 (0.1) | 0.0 (0.0) | 0.0 (0.0) | 0.0 (0.0) | 0.0 (0.0) | 0.0 (0.0) | 0.0 (0.0) | 0.0 (0.0) | 0.0 (0.2) | 0.0 (0.0) |
| Middle Skeena-mainstem tributaries | 0.0 (5.4) | 0.0 (0.0) | 0.0 (2.1) | 0.0 (4.8) | 0.0 (0.1) | 0.0 (0.3) | 0.0 (3.3) | 0.0 (0.0) | 0.0 (0.3) | 0.0 (0.1) |
| Middle Skeena-large lakes | 0.0 (0.0) | 0.0 (0.2) | 0.0 (2.4) | 0.0 (0.1) | 0.0 (0.0) | 0.0 (0.2) | 0.0 (0.3) | 0.0 (0.0) | 0.0 (0.0) | 0.0 (0.0) |
| Upper Skeena | 0.0 (1.5) | 0.0 (0.1) | 0.0 (0.4) | 0.0 (1.7) | 0.0 (0.0) | 0.0 (0.1) | 0.0 (0.6) | 0.0 (0.0) | 0.0 (0.6) | 0.0 (0.0) |
| Upper Bulkley River | 0.0 (0.2) | 0.0 (0.0) | 0.0 (0.0) | 0.0 (7.9) | 0.0 (0.0) | 0.0 (0.1) | 0.0 (0.0) | 0.0 (0.0) | 0.0 (0.1) | 0.0 (0.0) |
| North and Central Coast-late timing | 0.0 (3.5) | 0.0 (0.1) | 0.0 (0.0) | 0.0 (0.2) | 0.0 (0.0) | 0.0 (0.2) | 0.0 (0.8) | 0.0 (0.0) | 0.0 (0.0) | 0.0 (0.2) |
| North and Central Coast-early timing | 0.0 (4.8) | 0.0 (0.0) | 0.0 (0.0) | 0.0 (0.1) | 0.0 (0.0) | 0.0 (0.3) | 0.0 (3.4) | 0.0 (0.0) | 0.0 (0.1) | 0.0 (0.2) |
| Rivers Inlet | 0.0 (0.3) | 0.0 (0.0) | 0.0 (0.3) | 0.0 (2.3) | 0.0 (0.0) | 0.0 (0.4) | 0.0 (10.7) | 0.0 (0.0) | 0.0 (0.1) | 0.0 (0.0) |
| Wannock | 0.0 (1.2) | 0.0 (0.0) | 0.0 (0.0) | 0.0 (0.0) | 0.0 (0.0) | 0.0 (0.0) | 0.0 (1.2) | 0.0 (0.0) | 0.0 (0.0) | 0.0 (0.0) |
| Bella Coola-Bentinck | 0.0 (0.0) | 0.0 (0.0) | 0.0 (0.0) | 0.0 (1.3) | 0.0 (0.5) | 0.0 (0.0) | 0.0 (1.7) | 0.0 (0.2) | 0.0 (0.5) | 0.0 (0.6) |
| Dean River | 0.0 (0.0) | 0.0 (0.4) | 0.0 (0.0) | 0.0 (2.2) | 0.0 (0.0) | 0.0 (0.0) | 0.0 (0.2) | 0.0 (0.0) | 0.0 (0.0) | 0.0 (0.0) |
| Docee | 0.0 (0.0) | 0.0 (0.1) | 0.0 (0.4) | 0.0 (0.0) | 0.0 (0.0) | 0.0 (0.2) | 0.0 (1.7) | 0.0 (0.0) | 0.0 (0.0) | 0.0 (0.0) |
| Klinaklini_SU_1.3 | 0.0 (0.8) | 0.0 (1.4) | 0.0 (0.0) | 0.0 (0.6) | 0.0 (0.1) | 0.0 (0.2) | 0.0 (0.0) | 3.6 (0.7) | 0.0 (0.1) | 7.0 (3.9) |
| Southern Mainland-Southern Fjords_FA_0.x | 0.0 (0.0) | 0.0 (0.0) | 0.0 (0.0) | 0.0 (6.5) | 1.1 (0.9) | 0.0 (0.0) | 0.0 (0.7) | 0.2 (0.1) | 0.0 (0.0) | 0.0 (0.0) |
| Southern Mainland-Georgia Strait_FA_0.x | 0.0 (1.8) | 0.0 (0.7) | 0.0 (0.3) | 0.0 (7.8) | 5.4 (1.5) | 17.8 (6.0) | 66.7 (25.3) | 4.5 (1.1) | 0.0 (0.0) | 5.3 (3.9) |
| Upper Fraser River_SP_1.3 | 0.0 (1.8) | 0.0 (1.2) | 0.0 (1.8) | 0.0 (4.7) | 0.0 (0.3) | 0.0 (0.8) | 0.0 (2.3) | 1.0 (0.4) | 0.0 (0.1) | 0.0 (0.2) |
| Middle Fraser River_SU_1.3 | 0.0 (6.1) | 0.0 (1.1) | 0.0 (0.5) | 0.0 (2.5) | 0.0 (0.0) | 0.0 (0.1) | 0.0 (2.9) | 0.7 (0.3) | 0.0 (0.0) | 3.2 (3.1) |
| Middle Fraser River_SP_1.3 | 0.0 (3.0) | 0.0 (0.7) | 0.0 (1.6) | 0.0 (6.8) | 0.0 (0.1) | 0.0 (0.0) | 0.0 (1.5) | 0.0 (0.1) | 0.0 (0.7) | 0.9 (2.5) |
| Middle Fraser River-Portage_FA_1.3 | 0.0 (1.6) | 0.0 (0.0) | 0.0 (0.3) | 0.0 (0.0) | 0.0 (0.0) | 0.0 (0.0) | 0.0 (0.0) | 0.0 (0.0) | 0.0 (0.0) | 0.0 (0.0) |
| Middle Fraser-Fraser Canyon_SP_1.3 | 0.0 (0.2) | 0.0 (0.1) | 0.0 (0.1) | 0.0 (0.3) | 0.0 (0.0) | 0.0 (0.0) | 0.0 (0.8) | 0.0 (0.0) | 0.0 (0.0) | 0.0 (0.0) |
| North Thompson_SP_1.3 | 0.0 (0.0) | 0.0 (0.0) | 0.0 (0.4) | 0.0 (0.0) | 0.0 (0.0) | 0.0 (0.0) | 0.0 (4.3) | 0.2 (0.2) | 0.0 (0.0) | 2.0 (2.0) |
| North Thompson_SU_1.3 | 0.0 (0.8) | 0.0 (0.5) | 0.0 (0.2) | 0.0 (1.5) | 0.0 (0.0) | 0.0 (0.4) | 0.0 (4.0) | 0.3 (0.3) | 0.0 (0.0) | 0.0 (0.1) |
| Shuswap River_SU_0.3 | 0.0 (1.0) | 0.0 (0.0) | 0.0 (1.2) | 0.0 (0.1) | 0.0 (0.0) | 2.2 (1.8) | 0.0 (0.2) | 3.7 (0.6) | 0.0 (0.0) | 12.3 (4.4) |
| South Thompson-Bessette Creek_SU_1.2 | 0.0 (0.0) | 0.0 (0.4) | 0.0 (0.1) | 0.0 (12.2) | 0.0 (0.0) | 0.0 (0.0) | 0.0 (0.0) | 0.0 (0.0) | 0.0 (0.0) | 0.0 (0.1) |
| South Thompson_SU_0.3 | 0.0 (1.3) | 0.0 (0.1) | 0.0 (0.2) | 0.0 (4.5) | 0.5 (0.5) | 0.0 (0.0) | 0.0 (0.2) | 1.7 (0.5) | 0.0 (0.0) | 8.1 (4.0) |
| South Thompson_SU_1.3 | 0.0 (0.0) | 0.0 (0.0) | 0.0 (0.1) | 0.0 (0.1) | 0.0 (0.0) | 0.0 (0.0) | 0.0 (0.1) | 0.2 (0.2) | 0.0 (0.2) | 0.0 (0.0) |
| Lower Thompson_SP_1.2 | 0.0 (0.4) | 0.0 (0.5) | 0.0 (0.5) | 0.0 (6.9) | 0.0 (0.0) | 0.0 (0.1) | 0.0 (0.6) | 0.2 (0.1) | 0.0 (0.7) | 2.0 (1.9) |
| Lower Fraser River_SP_1.3 | 0.0 (0.2) | 0.0 (0.0) | 0.0 (0.0) | 0.0 (0.0) | 0.0 (0.0) | 0.0 (0.0) | 0.0 (0.0) | 0.0 (0.0) | 0.0 (0.0) | 0.0 (0.0) |
| Lower Fraser River_SU_1.3 | 0.0 (0.0) | 0.0 (0.0) | 0.0 (0.4) | 0.0 (0.0) | 0.0 (0.0) | 0.0 (0.0) | 0.0 (0.0) | 0.0 (0.0) | 0.0 (0.0) | 0.0 (0.1) |
| Lower Fraser River-Upper Pitt_SU_1.3 | 0.0 (0.0) | 0.0 (0.4) | 0.0 (0.7) | 0.0 (0.2) | 0.5 (0.6) | 0.0 (0.1) | 0.0 (0.0) | 1.5 (0.4) | 0.0 (0.0) | 6.1 (3.4) |
| Maria Slough_SU_0.3 | 0.0 (0.0) | 0.0 (0.0) | 0.0 (1.0) | 0.0 (0.0) | 0.0 (0.0) | 0.0 (0.2) | 0.0 (0.0) | 0.0 (0.0) | 0.0 (0.0) | 0.0 (0.0) |
| Lower Fraser River_FA_0.3 | 0.0 (0.4) | 0.0 (0.5) | 20.0 (10.5) | 0.0 (1.6) | 14.0 (2.7) | 8.9 (3.2) | 0.0 (0.0) | 26.7 (1.9) | 10.4 (3.7) | 8.2 (3.3) |
| East Vancouver Island-North_FA_0.x | 0.0 (0.5) | 5.3 (4.1) | 0.0 (1.5) | 0.0 (11.1) | 2.9 (1.0) | 8.9 (3.3) | 0.0 (1.8) | 1.2 (0.5) | 0.0 (0.0) | 2.0 (2.0) |
| East Vancouver Island-Qualicum and Puntledge_FA_0.x | 43.6 (19.8) | 84.2 (7.4) | 59.5 (14.9) | 100.0 (26.3) | 40.4 (3.4) | 33.9 (7.4) | 0.0 (0.2) | 28.6 (1.8) | 49.0 (7.7) | 30.7 (5.4) |
| East Vancouver Island-Nanaimo and Chemainus_FA_0.x | 0.0 (0.2) | 0.0 (0.0) | 0.0 (0.0) | 0.0 (1.8) | 1.3 (0.9) | 0.0 (0.0) | 0.0 (0.0) | 0.2 (0.2) | 0.0 (0.0) | 0.0 (0.0) |
| East Vancouver Island-Nanaimo_SP_1.x | 0.0 (0.1) | 0.8 (3.9) | 0.0 (0.0) | 0.0 (0.0) | 0.0 (0.0) | 0.0 (0.0) | 0.0 (0.1) | 0.0 (0.0) | 0.0 (0.0) | 0.0 (0.0) |
| East Vancouver Island-Georgia Strait_SU_0.3 | 16.7 (11.9) | 4.5 (2.8) | 0.0 (0.1) | 0.0 (13.9) | 0.5 (0.4) | 4.4 (3.3) | 0.0 (1.6) | 0.9 (0.3) | 5.1 (3.1) | 2.0 (2.3) |
| East Vancouver Island-Cowichan and Koksilah_FA_0.x | 23.1 (16.2) | 0.0 (0.0) | 0.4 (0.8) | 0.0 (0.3) | 19.5 (3.2) | 15.0 (6.4) | 0.0 (5.3) | 16.5 (1.6) | 23.0 (5.7) | 10.1 (4.7) |
| West Vancouver Island-Nootka and Kyuquot_FA_0.x | 0.0 (1.1) | 0.0 (0.1) | 0.0 (0.3) | 0.0 (7.3) | 0.0 (0.1) | 0.0 (0.2) | 0.0 (0.2) | 0.0 (0.0) | 0.0 (0.2) | 0.0 (0.2) |
| West Vancouver Island-North_FA_0.x | 0.0 (0.0) | 0.0 (0.1) | 0.0 (0.4) | 0.0 (0.2) | 0.0 (0.1) | 0.0 (0.3) | 0.0 (0.5) | 0.0 (0.0) | 0.0 (0.1) | 0.0 (0.1) |
| West Vancouver Island-South_FA_0.x | 0.0 (2.7) | 0.0 (0.1) | 0.0 (3.4) | 0.0 (7.5) | 0.0 (0.1) | 0.0 (0.4) | 0.0 (7.8) | 0.0 (0.0) | 0.0 (0.4) | 0.0 (0.5) |
| Okanagan_1.x | 0.0 (0.0) | 0.0 (0.0) | 0.0 (0.0) | 0.0 (0.3) | 0.0 (0.0) | 0.0 (0.1) | 0.0 (0.0) | 0.0 (0.0) | 0.0 (0.2) | 0.0 (0.0) |
| Juan de Fuca | 0.0 (0.0) | 0.0 (0.5) | 0.0 (0.0) | 0.0 (5.7) | 0.0 (0.0) | 0.0 (0.0) | 0.0 (0.0) | 0.0 (0.0) | 0.0 (0.0) | 0.0 (0.1) |
| Coastal Washington | 0.0 (0.7) | 0.0 (0.0) | 0.0 (0.0) | 0.0 (0.1) | 0.0 (0.0) | 0.0 (0.1) | 0.0 (1.1) | 0.0 (0.0) | 0.0 (0.2) | 0.0 (0.0) |
| North Puget Sound | 6.4 (10.5) | 5.3 (4.0) | 10.6 (7.8) | 0.0 (2.7) | 1.9 (0.9) | 6.7 (4.4) | 0.0 (0.0) | 4.0 (1.0) | 1.9 (2.3) | 0.0 (0.0) |
| South Puget Sound | 0.0 (3.3) | 0.0 (0.1) | 9.4 (6.2) | 0.0 (6.6) | 11.6 (2.3) | 1.9 (2.7) | 33.3 (20.8) | 3.8 (0.8) | 10.5 (4.3) | 0.0 (0.0) |
| Lower Columbia River | 0.0 (1.9) | 0.0 (0.2) | 0.0 (0.0) | 0.0 (0.1) | 0.0 (0.0) | 0.0 (0.6) | 0.0 (0.1) | 0.2 (0.1) | 0.0 (0.1) | 0.0 (0.0) |
| Mid Columbia River_SP | 10.2 (12.7) | 0.0 (0.2) | 0.0 (0.2) | 0.0 (0.3) | 0.0 (0.0) | 0.2 (0.7) | 0.0 (2.3) | 0.0 (0.0) | 0.0 (0.1) | 0.0 (0.3) |
| Upper Columbia River_SP | 0.0 (0.5) | 0.0 (0.0) | 0.0 (0.5) | 0.0 (0.3) | 0.0 (0.0) | 0.0 (0.1) | 0.0 (3.6) | 0.0 (0.0) | 0.0 (0.1) | 0.0 (0.1) |
| Upper Columbia River_SU_FA | 0.0 (1.1) | 0.0 (0.9) | 0.0 (1.4) | 0.0 (6.7) | 0.5 (0.5) | 0.0 (0.5) | 0.0 (0.2) | 0.2 (0.2) | 0.0 (0.0) | 0.0 (0.3) |
| Snake River_FA | 0.0 (3.3) | 0.0 (0.0) | 0.0 (0.2) | 0.0 (0.0) | 0.0 (0.0) | 0.0 (0.0) | 0.0 (0.0) | 0.1 (0.2) | 0.0 (0.0) | 0.0 (0.0) |
| Snake River_SP_SU | 0.0 (0.7) | 0.0 (0.8) | 0.0 (2.5) | 0.0 (3.0) | 0.0 (0.1) | 0.0 (0.2) | 0.0 (1.1) | 0.0 (0.0) | 0.0 (0.3) | 0.0 (0.3) |
| North & Central Oregon | 0.0 (1.7) | 0.0 (0.3) | 0.0 (1.6) | 0.0 (9.1) | 0.0 (0.0) | 0.0 (0.0) | 0.0 (1.1) | 0.0 (0.1) | 0.0 (0.1) | 0.0 (0.1) |
| Upper Willamette River | 0.0 (3.4) | 0.0 (0.0) | 0.0 (0.0) | 0.0 (0.4) | 0.0 (0.0) | 0.0 (0.2) | 0.0 (0.0) | 0.0 (0.0) | 0.0 (0.2) | 0.0 (0.0) |
| South Oregon coastal | 0.0 (0.0) | 0.0 (0.4) | 0.0 (2.4) | 0.0 (7.2) | 0.0 (0.0) | 0.0 (0.1) | 0.0 (0.8) | 0.0 (0.0) | 0.0 (0.0) | 0.0 (0.0) |
| California Klamath Trinity | 0.0 (2.4) | 0.0 (0.0) | 0.0 (0.1) | 0.0 (0.0) | 0.0 (0.2) | 0.0 (0.0) | 0.0 (0.6) | 0.0 (0.0) | 0.0 (0.0) | 0.0 (0.0) |
| California Central Valley_Fall | 0.0 (0.9) | 0.0 (1.6) | 0.0 (0.5) | 0.0 (3.1) | 0.0 (0.2) | 0.0 (0.6) | 0.0 (2.0) | 0.0 (0.1) | 0.0 (0.4) | 0.0 (0.1) |
| California Central Valley_Spring | 0.0 (0.3) | 0.0 (0.1) | 0.0 (0.1) | 0.0 (0.0) | 0.0 (0.0) | 0.0 (0.1) | 0.0 (0.0) | 0.0 (0.0) | 0.0 (0.0) | 0.0 (0.1) |
| Coastal California | 0.0 (0.0) | 0.0 (0.0) | 0.0 (0.0) | 0.0 (0.0) | 0.0 (0.0) | 0.0 (0.0) | 0.0 (0.7) | 0.0 (0.0) | 0.0 (0.1) | 0.0 (0.3) |

Supplementary Table S5 continued

| Conservation Unit | Strait of Georgia-north sport | | | | | | | Strait of Georgia-south sport | | |
| --- | --- | --- | --- | --- | --- | --- | --- | --- | --- | --- |
|  | August | | | September | | | October | January | | February |
|  | Legal | Sublegal | Unknown | Legal | Sublegal | Unknown | Legal | Legal | Sublegal | Legal |
| Sample size | 690 | 28 | 86 | 187 | 7 | 7 | 3 | 14 | 40 | 10 |
| N PBT | 200 | 7 | 20 | 48 | 4 | 3 | 0 | 2 | 1 | 1 |
| Southeast Alaska | 0.0 (0.0) | 0.0 (0.3) | 0.0 (0.0) | 0.0 (0.0) | 0.0 (1.6) | 0.0 (0.1) | 0.0 (1.1) | 0.0 (0.4) | 0.0 (0.9) | 0.0 (0.2) |
| Alsek | 0.0 (0.0) | 0.0 (0.2) | 0.0 (0.0) | 0.0 (0.0) | 0.0 (0.0) | 0.0 (0.0) | 0.0 (0.4) | 0.0 (0.1) | 0.0 (0.3) | 0.0 (0.0) |
| Unuk | 0.0 (0.0) | 0.0 (0.0) | 0.0 (0.0) | 0.0 (0.0) | 0.0 (0.1) | 0.0 (0.7) | 0.0 (0.0) | 0.0 (0.7) | 0.0 (0.0) | 0.0 (0.0) |
| Taku_early timing | 0.0 (0.0) | 0.0 (0.0) | 0.0 (0.1) | 0.0 (0.0) | 0.0 (1.6) | 0.0 (0.2) | 0.0 (1.0) | 0.0 (0.0) | 0.0 (0.0) | 0.0 (0.1) |
| Taku_mid timing | 0.0 (0.0) | 0.0 (0.1) | 0.0 (0.0) | 0.0 (0.0) | 0.0 (1.1) | 0.0 (1.2) | 0.0 (3.3) | 0.0 (0.0) | 0.0 (0.0) | 0.0 (1.1) |
| Taku_late timing | 0.0 (0.0) | 0.0 (0.0) | 0.0 (0.2) | 0.0 (0.0) | 0.0 (0.2) | 0.0 (0.0) | 0.0 (0.0) | 0.0 (0.5) | 0.0 (0.4) | 0.0 (0.1) |
| Stikine_early timing | 0.0 (0.0) | 0.0 (0.1) | 0.0 (0.3) | 0.0 (0.1) | 0.0 (1.5) | 0.0 (0.1) | 0.0 (2.9) | 0.0 (0.5) | 0.0 (0.1) | 0.0 (0.2) |
| Stikine_late timing | 0.0 (0.0) | 0.0 (0.0) | 0.0 (0.0) | 0.0 (0.0) | 0.0 (0.0) | 0.0 (0.8) | 0.0 (1.0) | 0.0 (0.0) | 0.0 (0.0) | 0.0 (1.1) |
| Haida Gwaii-North | 0.0 (0.0) | 0.0 (0.0) | 0.0 (0.0) | 0.0 (0.0) | 0.0 (0.3) | 0.0 (0.0) | 0.0 (1.1) | 0.0 (0.4) | 0.0 (0.0) | 0.0 (0.0) |
| Upper Nass | 0.0 (0.0) | 0.0 (0.0) | 0.0 (0.1) | 0.0 (0.1) | 0.0 (2.8) | 0.0 (0.4) | 0.0 (6.6) | 0.0 (0.7) | 0.0 (0.4) | 0.0 (2.9) |
| Portland Sound-Observatory Inlet-Lower Nass | 0.0 (0.0) | 0.0 (0.1) | 0.0 (0.1) | 0.0 (0.0) | 0.0 (0.0) | 0.0 (3.8) | 0.0 (1.2) | 0.0 (0.0) | 0.0 (0.1) | 0.0 (0.6) |
| Ecstall | 0.0 (0.0) | 0.0 (0.1) | 0.0 (0.0) | 0.0 (0.0) | 0.0 (0.0) | 0.0 (0.4) | 0.0 (0.0) | 0.0 (0.0) | 0.0 (0.0) | 0.0 (0.0) |
| Skeena Estuary | 0.0 (0.0) | 0.0 (0.0) | 0.0 (0.0) | 0.0 (0.0) | 0.0 (0.0) | 0.0 (1.1) | 0.0 (0.0) | 0.0 (0.0) | 0.0 (0.0) | 0.0 (0.1) |
| Lower Skeena | 0.0 (0.0) | 0.0 (1.0) | 0.0 (0.0) | 0.0 (0.0) | 0.0 (0.3) | 0.0 (0.1) | 0.0 (1.4) | 0.0 (0.9) | 0.0 (0.0) | 0.0 (1.7) |
| Kalum_early timing | 0.0 (0.0) | 0.0 (0.0) | 0.0 (0.0) | 0.0 (0.0) | 0.0 (0.0) | 0.0 (0.1) | 0.0 (0.0) | 0.0 (0.0) | 0.0 (0.0) | 0.0 (0.2) |
| Kalum_late timing | 0.0 (0.0) | 0.0 (0.0) | 1.2 (1.3) | 0.0 (0.0) | 0.0 (0.9) | 0.0 (0.1) | 0.0 (2.3) | 0.0 (0.0) | 0.0 (0.0) | 0.0 (0.0) |
| Zymoetz | 0.0 (0.0) | 0.0 (0.0) | 0.0 (0.0) | 0.0 (0.0) | 0.0 (0.0) | 0.0 (0.4) | 0.0 (0.1) | 0.0 (0.3) | 0.0 (0.0) | 0.0 (0.3) |
| Sicintine | 0.0 (0.0) | 0.0 (0.0) | 0.0 (0.0) | 0.0 (0.0) | 0.0 (0.0) | 0.0 (0.9) | 0.0 (1.9) | 0.0 (0.0) | 0.0 (0.1) | 0.0 (0.3) |
| Middle Skeena-mainstem tributaries | 0.0 (0.0) | 0.0 (0.3) | 0.0 (0.1) | 0.0 (0.0) | 0.0 (0.9) | 0.0 (1.4) | 0.0 (0.9) | 0.0 (1.3) | 0.0 (0.1) | 0.0 (0.9) |
| Middle Skeena-large lakes | 0.0 (0.0) | 0.0 (0.0) | 0.0 (0.0) | 0.0 (0.0) | 0.0 (0.3) | 0.0 (0.5) | 0.0 (2.1) | 0.0 (0.0) | 0.0 (0.2) | 0.0 (0.0) |
| Upper Skeena | 0.0 (0.0) | 0.0 (0.3) | 0.0 (0.0) | 0.0 (0.0) | 0.0 (2.2) | 0.0 (0.0) | 0.0 (0.9) | 0.0 (0.0) | 0.0 (0.1) | 0.0 (0.7) |
| Upper Bulkley River | 0.0 (0.0) | 0.0 (1.3) | 0.0 (0.0) | 0.0 (0.0) | 0.0 (0.0) | 0.0 (0.0) | 0.0 (0.0) | 0.0 (0.0) | 0.0 (0.0) | 0.0 (0.0) |
| North and Central Coast-late timing | 0.0 (0.0) | 0.0 (0.1) | 0.0 (0.0) | 0.0 (0.0) | 0.0 (0.0) | 0.0 (0.7) | 0.0 (0.2) | 0.0 (0.5) | 0.0 (0.0) | 0.0 (0.0) |
| North and Central Coast-early timing | 0.0 (0.0) | 0.0 (0.7) | 0.0 (0.0) | 0.0 (0.0) | 0.0 (0.3) | 0.0 (0.4) | 0.0 (0.1) | 0.0 (0.5) | 0.0 (0.7) | 0.0 (0.0) |
| Rivers Inlet | 0.0 (0.0) | 0.0 (0.1) | 0.0 (0.0) | 0.0 (0.1) | 0.0 (0.4) | 0.0 (0.8) | 0.0 (0.1) | 0.0 (0.2) | 0.0 (0.1) | 0.0 (0.3) |
| Wannock | 0.0 (0.0) | 0.0 (0.0) | 0.0 (0.0) | 0.0 (0.0) | 0.0 (0.0) | 0.0 (1.0) | 0.0 (0.0) | 0.0 (0.0) | 0.0 (0.0) | 0.0 (0.0) |
| Bella Coola-Bentinck | 0.1 (0.1) | 0.0 (0.0) | 0.0 (0.0) | 0.0 (0.0) | 0.0 (0.0) | 0.0 (0.0) | 0.0 (0.0) | 0.0 (0.0) | 0.0 (0.0) | 0.0 (1.0) |
| Dean River | 0.0 (0.0) | 0.0 (0.1) | 0.0 (0.0) | 0.0 (0.0) | 0.0 (0.1) | 0.0 (0.7) | 0.0 (0.6) | 0.0 (0.0) | 0.0 (0.0) | 0.0 (0.0) |
| Docee | 0.0 (0.0) | 0.0 (0.0) | 0.0 (0.0) | 0.0 (0.0) | 0.0 (0.2) | 0.0 (0.2) | 0.0 (0.1) | 0.0 (0.0) | 0.0 (0.0) | 0.0 (0.1) |
| Klinaklini_SU_1.3 | 0.0 (0.0) | 0.0 (0.4) | 0.0 (0.0) | 0.0 (0.0) | 0.0 (0.0) | 0.0 (0.0) | 0.0 (1.0) | 0.0 (0.0) | 0.0 (0.2) | 0.0 (0.0) |
| Southern Mainland-Southern Fjords_FA_0.x | 0.2 (0.2) | 0.0 (0.0) | 0.0 (0.0) | 0.0 (0.0) | 0.0 (0.0) | 0.0 (0.0) | 0.0 (1.8) | 0.0 (0.0) | 0.0 (0.0) | 0.0 (0.0) |
| Southern Mainland-Georgia Strait_FA_0.x | 0.7 (0.3) | 0.0 (0.6) | 2.3 (1.7) | 0.0 (0.1) | 0.0 (0.0) | 0.0 (1.6) | 0.1 (1.6) | 0.0 (0.6) | 0.0 (0.6) | 0.0 (1.1) |
| Upper Fraser River_SP_1.3 | 0.1 (0.2) | 0.0 (0.4) | 0.0 (0.0) | 0.0 (0.1) | 0.0 (1.9) | 0.0 (2.2) | 0.0 (3.0) | 0.0 (2.0) | 0.0 (0.5) | 0.0 (0.5) |
| Middle Fraser River_SU_1.3 | 0.0 (0.0) | 0.0 (0.6) | 0.0 (0.0) | 0.0 (0.1) | 0.0 (0.0) | 0.0 (4.2) | 0.0 (2.9) | 0.0 (0.7) | 0.0 (0.1) | 0.0 (1.1) |
| Middle Fraser River_SP_1.3 | 0.0 (0.0) | 0.0 (0.5) | 1.2 (1.0) | 0.0 (0.1) | 0.0 (1.1) | 0.0 (2.0) | 0.0 (1.2) | 0.0 (2.6) | 0.0 (0.9) | 0.0 (2.7) |
| Middle Fraser River-Portage_FA_1.3 | 0.0 (0.0) | 0.0 (0.0) | 0.0 (0.0) | 0.0 (0.0) | 0.0 (0.0) | 0.0 (0.0) | 0.0 (0.1) | 0.0 (0.0) | 0.0 (0.0) | 0.0 (0.0) |
| Middle Fraser-Fraser Canyon_SP_1.3 | 0.0 (0.0) | 0.0 (0.4) | 0.0 (0.0) | 0.0 (0.0) | 0.0 (0.0) | 0.0 (0.0) | 0.0 (0.0) | 0.0 (0.1) | 0.0 (0.0) | 0.0 (0.0) |
| North Thompson_SP_1.3 | 0.0 (0.0) | 0.0 (0.0) | 0.0 (0.0) | 0.0 (0.0) | 0.0 (0.0) | 0.0 (0.8) | 0.0 (1.8) | 0.0 (0.2) | 0.0 (0.1) | 0.0 (2.1) |
| North Thompson_SU_1.3 | 0.0 (0.0) | 0.0 (0.3) | 0.0 (0.0) | 0.0 (0.1) | 0.0 (0.6) | 0.0 (1.1) | 0.0 (0.0) | 0.0 (0.1) | 5.0 (3.2) | 0.0 (0.2) |
| Shuswap River_SU_0.3 | 2.2 (0.6) | 0.0 (0.5) | 8.0 (2.4) | 1.1 (0.4) | 0.0 (0.3) | 0.0 (0.1) | 0.0 (0.0) | 0.0 (0.0) | 0.0 (0.0) | 0.0 (0.1) |
| South Thompson-Bessette Creek_SU_1.2 | 0.0 (0.0) | 0.0 (0.6) | 0.0 (0.0) | 0.0 (0.1) | 0.0 (2.1) | 0.0 (0.2) | 0.0 (0.5) | 0.0 (0.6) | 0.0 (0.1) | 0.0 (0.2) |
| South Thompson_SU_0.3 | 17.9 (1.9) | 0.0 (0.2) | 36.2 (4.9) | 13.9 (2.9) | 0.0 (0.8) | 0.0 (0.0) | 0.0 (0.6) | 0.0 (0.0) | 0.0 (0.0) | 0.0 (0.1) |
| South Thompson_SU_1.3 | 0.0 (0.0) | 0.0 (0.0) | 0.0 (0.0) | 0.0 (0.0) | 0.0 (0.0) | 0.0 (0.0) | 0.0 (6.6) | 0.0 (0.1) | 0.0 (0.2) | 0.0 (0.0) |
| Lower Thompson_SP_1.2 | 0.0 (0.0) | 0.0 (0.0) | 0.0 (0.0) | 0.0 (0.2) | 0.0 (2.9) | 0.0 (2.9) | 0.0 (3.2) | 0.0 (1.7) | 0.0 (0.3) | 0.0 (0.1) |
| Lower Fraser River_SP_1.3 | 0.0 (0.0) | 0.0 (0.0) | 0.0 (0.0) | 0.0 (0.0) | 0.0 (0.0) | 0.0 (0.0) | 0.0 (0.1) | 0.0 (0.0) | 0.0 (0.0) | 0.0 (0.5) |
| Lower Fraser River_SU_1.3 | 0.0 (0.0) | 0.0 (0.0) | 0.0 (0.3) | 0.0 (0.0) | 0.0 (0.0) | 0.0 (0.0) | 0.0 (0.2) | 0.0 (0.0) | 0.0 (0.0) | 0.0 (0.0) |
| Lower Fraser River-Upper Pitt_SU_1.3 | 0.1 (0.2) | 0.0 (0.0) | 0.0 (0.1) | 0.0 (0.0) | 0.0 (0.0) | 0.0 (0.1) | 0.0 (1.7) | 0.0 (0.6) | 0.0 (0.0) | 0.0 (0.3) |
| Maria Slough_SU_0.3 | 0.0 (0.0) | 0.0 (0.0) | 0.0 (0.0) | 0.0 (0.0) | 0.0 (0.0) | 0.0 (0.0) | 0.0 (0.2) | 0.0 (0.1) | 0.0 (0.0) | 0.0 (0.0) |
| Lower Fraser River_FA_0.3 | 31.3 (1.8) | 15.7 (6.6) | 20.8 (4.4) | 31.6 (3.5) | 14.3 (12.4) | 28.6 (14.3) | 0.0 (0.2) | 7.1 (4.5) | 2.5 (3.4) | 20.0 (12.9) |
| East Vancouver Island-North_FA_0.x | 2.2 (0.7) | 0.0 (0.2) | 4.7 (2.1) | 4.3 (1.2) | 0.0 (1.9) | 28.6 (14.6) | 0.0 (1.0) | 0.0 (0.1) | 0.0 (0.0) | 0.0 (0.6) |
| East Vancouver Island-Qualicum and Puntledge_FA_0.x | 24.6 (1.9) | 33.4 (8.4) | 20.4 (4.5) | 22.2 (3.1) | 70.9 (15.3) | 15.1 (11.0) | 27.5 (22.7) | 14.1 (9.7) | 0.0 (0.4) | 18.0 (9.7) |
| East Vancouver Island-Nanaimo and Chemainus_FA_0.x | 0.0 (0.0) | 3.2 (4.0) | 0.0 (0.0 ) | 0.0 (0.0) | 0.0 (0.0 ) | 0.0 (0.3) | 0.0 (0.7) | 0.0 (0.3 ) | 0.0 (0.0) | 2.0 (5.4) |
| East Vancouver Island-Nanaimo_SP_1.x | 0.0 (0.0) | 0.0 (0.0) | 0.0 (0.1) | 0.0 (0.0) | 0.0 (0.0) | 0.0 (0.0) | 0.0 (0.0) | 0.0 (0.0) | 0.0 (0.0) | 0.0 (0.0) |
| East Vancouver Island-Georgia Strait_SU_0.3 | 0.3 (0.2) | 0.0 (0.6) | 0.0 (0.1) | 0.5 (0.5) | 0.0 (0.6) | 0.0 (0.1) | 0.0 (0.0) | 7.1 (5.0) | 5.0 (2.9) | 10.0 (7.2) |
| East Vancouver Island-Cowichan and Koksilah_FA_0.x | 13.3 (1.2) | 37.1 (9.3) | 3.5 (2.1) | 19.5 (2.7) | 0.5 (1.1) | 27.8 (15.6) | 72.5 (28.3) | 0.2 (0.4) | 10.0 (4.4) | 0.0 (0.9) |
| West Vancouver Island-Nootka and Kyuquot_FA_0.x | 0.0 (0.0) | 0.0 (0.3) | 0.0 (0.4) | 0.0 (0.1) | 0.0 (0.3) | 0.0 (0.4) | 0.0 (0.4) | 0.0 (0.2) | 0.0 (1.1) | 0.0 (1.3) |
| West Vancouver Island-North_FA_0.x | 0.0 (0.0) | 0.0 (0.0) | 0.0 (0.0) | 0.0 (0.1) | 0.0 (0.0) | 0.0 (0.3) | 0.0 (2.2) | 0.0 (0.0) | 0.0 (0.3) | 0.0 (0.0) |
| West Vancouver Island-South_FA_0.x | 0.3 (0.2) | 0.0 (0.4) | 0.0 (0.1) | 0.0 (0.1) | 0.0 (1.5) | 0.0 (0.6) | 0.0 (4.2) | 0.0 (1.6) | 0.0 (0.1) | 0.0 (0.7) |
| Okanagan_1.x | 0.0 (0.0) | 0.0 (0.0) | 0.0 (0.0) | 0.0 (0.0) | 0.0 (0.0) | 0.0 (0.5) | 0.0 (0.1) | 0.0 (0.2) | 0.0 (0.0) | 0.0 (0.2) |
| Juan de Fuca | 0.1 (0.2) | 0.0 (0.0) | 0.0 (0.0) | 0.0 (0.0) | 0.0 (0.0) | 0.0 (0.3) | 0.0 (0.0) | 0.0 (0.0) | 0.0 (0.1) | 0.0 (0.0) |
| Coastal Washington | 0.0 (0.0) | 0.0 (0.1) | 0.0 (0.4) | 0.0 (0.0) | 0.0 (0.4) | 0.0 (0.0) | 0.0 (5.6) | 0.0 (0.0) | 0.0 (0.0) | 0.0 (0.0) |
| North Puget Sound | 1.6 (0.5) | 0.0 (0.2) | 1.7 (1.5) | 2.4 (0.9) | 0.0 (0.2) | 0.0 (1.1) | 0.0 (0.1) | 27.3 (11.8) | 31.1 (9.1) | 6.1 (2.0) |
| South Puget Sound | 4.2 (0.7) | 10.7 (5.4) | 0.0 (0.0) | 4.1 (1.5) | 14.3 (9.0) | 0.0 (0.5) | 0.0 (7.9) | 42.8 (12.0) | 43.8 (7.6) | 43.7 (15.5) |
| Lower Columbia River | 0.1 (0.2) | 0.0 (0.4) | 0.0 (0.1) | 0.0 (0.0) | 0.0 (0.1) | 0.0 (0.3) | 0.0 (0.2) | 0.0 (0.1) | 2.5 (2.4) | 0.0 (0.3) |
| Mid Columbia River_SP | 0.5 (0.4) | 0.0 (0.1) | 0.0 (0.1) | 0.0 (0.1) | 0.0 (0.7) | 0.0 (0.1) | 0.0 (0.4) | 1.2 (4.4) | 0.0 (0.0) | 0.3 (1.4) |
| Upper Columbia River_SP | 0.0 (0.0) | 0.0 (0.7) | 0.0 (0.0) | 0.0 (0.0) | 0.0 (0.1) | 0.0 (0.0) | 0.0 (0.5) | 0.0 (0.5) | 0.0 (0.0) | 0.0 (0.1) |
| Upper Columbia River_SU_FA | 0.0 (0.0) | 0.0 (0.2) | 0.0 (0.0) | 0.5 (0.4) | 0.0 (1.9) | 0.0 (1.1) | 0.0 (0.0) | 0.0 (0.1) | 0.0 (0.1) | 0.0 (0.5) |
| Snake River_FA | 0.0 (0.0) | 0.0 (0.0) | 0.0 (0.0) | 0.0 (0.2) | 0.0 (0.0) | 0.0 (0.0) | 0.0 (0.0) | 0.0 (0.0) | 0.0 (0.0) | 0.0 (0.0) |
| Snake River_SP_SU | 0.0 (0.0) | 0.0 (0.4) | 0.0 (0.1) | 0.0 (0.0) | 0.0 (1.6) | 0.0 (2.7) | 0.0 (0.3) | 0.0 (0.3) | 0.0 (0.4) | 0.0 (1.9) |
| North & Central Oregon | 0.0 (0.0) | 0.0 (0.7) | 0.0 (0.2) | 0.0 (0.0) | 0.0 (0.6) | 0.0 (0.1) | 0.0 (3.2) | 0.0 (0.0) | 0.0 (0.0) | 0.0 (0.5) |
| Upper Willamette River | 0.0 (0.0) | 0.0 (0.1) | 0.0 (0.0) | 0.0 (0.0) | 0.0 (0.5) | 0.0 (0.2) | 0.0 (0.0) | 0.0 (0.0) | 0.0 (0.1) | 0.0 (0.1) |
| South Oregon coastal | 0.0 (0.0) | 0.0 (0.4) | 0.0 (0.1) | 0.0 (0.0) | 0.0 (1.5) | 0.0 (0.2) | 0.0 (2.3) | 0.0 (0.0) | 0.0 (0.4) | 0.0 (2.0) |
| California Klamath Trinity | 0.0 (0.0) | 0.0 (0.1) | 0.0 (0.3) | 0.0 (0.0) | 0.0 (1.1) | 0.0 (0.8) | 0.0 (0.0) | 0.0 (0.2) | 0.0 (0.1) | 0.0 (0.2) |
| California Central Valley_Fall | 0.0 (0.0) | 0.0 (0.2) | 0.0 (0.2) | 0.0 (0.0) | 0.0 (2.2) | 0.0 (2.3) | 0.0 (3.4) | 0.0 (2.4) | 0.0 (0.3) | 0.0 (0.6) |
| California Central Valley_Spring | 0.0 (0.0) | 0.0 (0.0) | 0.0 (0.0) | 0.0 (0.0) | 0.0 (0.0) | 0.0 (0.1) | 0.0 (0.4) | 0.0 (0.0) | 0.0 (0.3) | 0.0 (0.0) |
| Coastal California | 0.0 (0.0) | 0.0 (0.0) | 0.0 (0.0) | 0.0 (0.0) | 0.0 (3.8) | 0.0 (0.0) | 0.0 (0.0) | 0.0 (0.0) | 0.0 (0.0) | 0.0 (0.2) |

Supplementary Table S5 continued

| Conservation Unit | Strait of Georgia-south sport | | | | | | | | | |
| --- | --- | --- | --- | --- | --- | --- | --- | --- | --- | --- |
|  | February | March | | | April | | May | | June | |
|  | Sublegal | Legal | Sublegal | Unknown | Legal | Sublegal | Legal | Sublegal | Legal | Sublegal |
| Sample size | 10 | 50 | 47 | 2 | 31 | 71 | 61 | 35 | 53 | 31 |
| N PBT | 0 | 5 | 6 | 0 | 8 | 17 | 14 | 3 | 15 | 3 |
| Southeast Alaska | 0.0 (0.5) | 0.0 (0.1) | 0.0 (0.1) | 0.0 (5.4) | 0.0 (1.0) | 0.0 (0.0) | 0.0 (0.0) | 0.0 (0.4) | 0.0 (0.2) | 0.0 (0.2) |
| Alsek | 0.0 (0.0) | 0.0 (0.1) | 0.0 (0.0) | 0.0 (1.8) | 0.0 (0.2) | 0.0 (0.0) | 0.0 (0.4) | 0.0 (0.2) | 0.0 (0.1) | 0.0 (0.1) |
| Unuk | 0.0 (0.5) | 0.0 (0.0) | 0.0 (0.0) | 0.0 (3.2) | 0.0 (1.4) | 0.0 (0.0) | 0.0 (0.0) | 0.0 (0.0) | 0.0 (0.0) | 0.0 (0.0) |
| Taku_early timing | 0.0 (0.0) | 0.0 (0.0) | 0.0 (0.1) | 0.0 (0.3) | 0.0 (0.1) | 0.0 (0.0) | 0.0 (0.0) | 0.0 (0.1) | 0.0 (0.4) | 0.0 (0.0) |
| Taku_mid timing | 0.0 (0.3) | 0.0 (0.7) | 0.0 (0.1) | 0.0 (5.9) | 0.0 (0.1) | 0.0 (0.0) | 0.0 (0.0) | 0.0 (0.0) | 0.0 (0.0) | 0.0 (0.1) |
| Taku_late timing | 0.0 (0.5) | 0.0 (0.0) | 0.0 (0.0) | 0.0 (3.5) | 0.0 (0.1) | 0.0 (0.1) | 0.0 (0.0) | 0.0 (0.0) | 0.0 (0.0) | 0.0 (0.0) |
| Stikine_early timing | 0.0 (0.0) | 0.0 (0.1) | 0.0 (0.4) | 0.0 (0.2) | 0.0 (0.3) | 0.0 (0.2) | 0.0 (0.0) | 0.0 (0.0) | 0.0 (0.1) | 0.0 (0.0) |
| Stikine_late timing | 0.0 (1.8) | 0.0 (0.0) | 0.0 (0.0) | 0.0 (0.9) | 0.0 (0.2) | 0.0 (0.1) | 0.0 (0.0) | 0.0 (0.0) | 0.0 (0.9) | 0.0 (0.2) |
| Haida Gwaii-North | 0.0 (0.0) | 0.0 (0.0) | 0.0 (0.6) | 0.0 (0.3) | 0.0 (0.0) | 0.0 (0.0) | 0.0 (0.0) | 0.0 (0.0) | 0.0 (0.0) | 0.0 (0.0) |
| Upper Nass | 0.0 (0.6) | 0.0 (0.0) | 0.0 (0.3) | 0.0 (6.3) | 0.0 (0.1) | 0.0 (0.1) | 0.0 (0.1) | 0.0 (0.1) | 0.0 (0.2) | 0.0 (0.0) |
| Portland Sound-Observatory Inlet-Lower Nass | 0.0 (0.6) | 0.0 (0.0) | 0.0 (0.0) | 0.0 (7.5) | 0.0 (0.1) | 0.0 (0.1) | 0.0 (0.1) | 0.0 (0.9) | 0.0 (0.0) | 0.0 (0.0) |
| Ecstall | 0.0 (0.2) | 0.0 (0.0) | 0.0 (0.0) | 0.0 (0.0) | 0.0 (0.0) | 0.0 (0.0) | 0.0 (0.0) | 0.0 (0.0) | 0.0 (0.1) | 0.0 (0.4) |
| Skeena Estuary | 0.0 (0.0) | 0.0 (0.3) | 0.0 (0.0) | 0.0 (0.3) | 0.0 (0.0) | 0.0 (0.0) | 0.0 (0.0) | 0.0 (0.1) | 0.0 (0.1) | 0.0 (0.0) |
| Lower Skeena | 0.0 (0.5) | 0.0 (0.0) | 0.0 (0.1) | 0.0 (0.6) | 0.0 (0.0) | 0.0 (0.1) | 0.0 (0.1) | 0.0 (0.4) | 0.0 (0.2) | 0.0 (0.1) |
| Kalum_early timing | 0.0 (0.0) | 0.0 (0.0) | 0.0 (0.0) | 0.0 (0.3) | 0.0 (0.0) | 0.0 (0.0) | 0.0 (0.0) | 0.0 (0.0) | 0.0 (0.0) | 0.0 (0.0) |
| Kalum_late timing | 0.0 (0.0) | 0.0 (0.1) | 0.0 (0.0) | 0.0 (0.2) | 0.0 (0.0) | 0.0 (0.0) | 0.0 (0.0) | 0.0 (0.0) | 0.0 (0.0) | 0.0 (0.0) |
| Zymoetz | 0.0 (0.5) | 0.0 (0.0) | 0.0 (0.0) | 0.0 (0.0) | 0.0 (0.0) | 0.0 (0.0) | 0.0 (0.0) | 0.0 (0.0) | 0.0 (0.0) | 0.0 (0.0) |
| Sicintine | 0.0 (0.1) | 0.0 (0.2) | 0.0 (0.0) | 0.0 (0.0) | 0.0 (0.0) | 0.0 (0.0) | 0.0 (0.0) | 0.0 (1.0) | 0.0 (0.0) | 0.0 (0.0) |
| Middle Skeena-mainstem tributaries | 0.0 (1.1) | 0.0 (0.2) | 0.0 (0.1) | 0.0 (2.2) | 0.0 (0.3) | 0.0 (0.1) | 0.0 (0.0) | 0.0 (0.2) | 0.0 (0.1) | 0.0 (0.3) |
| Middle Skeena-large lakes | 0.0 (0.0) | 0.0 (0.3) | 0.0 (0.0) | 0.0 (1.4) | 0.0 (0.1) | 0.0 (0.1) | 0.0 (0.7) | 0.0 (0.0) | 0.0 (0.1) | 0.0 (0.0) |
| Upper Skeena | 0.0 (0.3) | 0.0 (0.1) | 0.0 (0.0) | 0.0 (4.8) | 0.0 (0.0) | 0.0 (0.0) | 0.0 (0.3) | 0.0 (0.0) | 0.0 (0.0) | 0.0 (0.2) |
| Upper Bulkley River | 0.0 (0.2) | 0.0 (0.0) | 0.0 (0.0) | 0.0 (0.0) | 0.0 (0.0) | 0.0 (0.3) | 0.0 (0.0) | 0.0 (0.0) | 0.0 (0.0) | 0.0 (0.2) |
| North and Central Coast-late timing | 0.0 (0.6) | 0.0 (0.1) | 0.0 (0.0) | 0.0 (2.7) | 0.0 (0.4) | 0.0 (0.0) | 0.0 (0.4) | 0.0 (0.4) | 0.0 (0.0) | 0.0 (0.1) |
| North and Central Coast-early timing | 0.0 (0.4) | 0.0 (0.0) | 0.0 (0.2) | 0.0 (0.0) | 0.0 (0.0) | 0.0 (0.0) | 0.0 (0.3) | 0.0 (0.0) | 0.0 (0.4) | 0.0 (0.0) |
| Rivers Inlet | 0.0 (0.3) | 0.0 (0.1) | 0.0 (0.1) | 0.0 (0.5) | 0.0 (0.0) | 0.0 (0.0) | 0.0 (0.0) | 0.0 (0.1) | 0.0 (0.1) | 0.0 (0.3) |
| Wannock | 0.0 (0.0) | 0.0 (0.0) | 0.0 (0.1) | 0.0 (0.0) | 0.0 (0.0) | 0.0 (0.0) | 0.0 (0.0) | 0.0 (0.0) | 0.0 (0.0) | 0.0 (0.1) |
| Bella Coola-Bentinck | 0.0 (0.5) | 0.0 (0.1) | 0.0 (0.1) | 0.0 (0.3) | 0.0 (0.0) | 0.0 (0.2) | 0.0 (0.2) | 0.0 (0.0) | 0.0 (0.0) | 0.0 (0.0) |
| Dean River | 0.0 (0.5) | 0.0 (0.2) | 0.0 (0.4) | 0.0 (0.6) | 0.0 (0.3) | 0.0 (0.0) | 0.0 (0.0) | 0.0 (0.0) | 0.0 (0.1) | 0.0 (0.0) |
| Docee | 0.0 (0.3) | 0.0 (0.1) | 0.0 (0.0) | 0.0 (0.1) | 0.0 (0.0) | 0.0 (0.0) | 0.0 (0.0) | 0.0 (0.1) | 0.0 (0.0) | 0.0 (0.0) |
| Klinaklini_SU_1.3 | 0.0 (3.1) | 0.0 (0.0) | 0.0 (0.0) | 0.0 (0.0) | 0.0 (0.0) | 0.0 (0.0) | 0.0 (0.1) | 0.0 (0.1) | 0.0 (0.0) | 0.0 (0.0) |
| Southern Mainland-Southern Fjords_FA_0.x | 0.0 (0.0) | 0.0 (0.0) | 0.0 (0.1) | 0.0 (0.0) | 0.0 (0.0) | 0.0 (0.0) | 0.0 (0.0) | 0.0 (0.0) | 0.0 (0.0) | 0.0 (0.1) |
| Southern Mainland-Georgia Strait_FA_0.x | 0.0 (1.4) | 4.0 (2.9) | 2.1 (2.4) | 0.0 (8.3) | 9.7 (4.7) | 2.8 (2.0) | 1.6 (1.2) | 2.9 (2.2) | 5.7 (3.8) | 6.5 (5.0) |
| Upper Fraser River_SP_1.3 | 0.0 (2.0) | 0.0 (0.3) | 0.0 (0.3) | 0.0 (3.8) | 0.0 (0.5) | 0.0 (0.8) | 0.0 (0.2) | 0.0 (0.4) | 0.0 (0.8) | 0.0 (1.1) |
| Middle Fraser River_SU_1.3 | 0.0 (2.5) | 0.0 (0.0) | 0.0 (0.0) | 0.0 (4.7) | 0.0 (0.3) | 1.4 (1.3) | 0.0 (0.4) | 0.0 (0.4) | 0.0 (0.2) | 0.0 (0.4) |
| Middle Fraser River_SP_1.3 | 0.0 (3.2) | 0.0 (0.2) | 0.0 (0.1) | 0.0 (6.4) | 0.0 (0.3) | 0.0 (0.0) | 0.0 (0.2) | 0.0 (0.6) | 0.0 (0.3) | 0.0 (0.2) |
| Middle Fraser River-Portage_FA_1.3 | 0.0 (0.0) | 0.0 (0.0) | 0.0 (0.0) | 0.0 (0.0) | 0.0 (0.0) | 0.0 (0.0) | 0.0 (0.0) | 0.0 (0.2) | 0.0 (0.0) | 0.0 (0.0) |
| Middle Fraser-Fraser Canyon_SP_1.3 | 0.0 (0.0) | 0.0 (0.0) | 0.0 (0.0) | 0.0 (0.0) | 0.0 (0.0) | 0.0 (0.2) | 0.0 (0.0) | 0.0 (0.0) | 0.0 (0.0) | 0.0 (0.0) |
| North Thompson_SP_1.3 | 0.0 (0.0) | 0.0 (0.0) | 0.0 (0.0) | 0.0 (0.0) | 0.0 (0.0) | 0.0 (0.0) | 0.0 (0.1) | 0.0 (0.0) | 0.0 (0.0) | 0.0 (0.0) |
| North Thompson_SU_1.3 | 0.0 (0.2) | 0.0 (0.7) | 0.0 (0.0) | 0.0 (0.0) | 0.0 (0.0) | 1.4 (1.8) | 0.0 (0.1) | 0.0 (0.0) | 0.0 (0.1) | 0.0 (0.1) |
| Shuswap River_SU_0.3 | 0.0 (0.3) | 0.0 (0.0) | 0.0 (0.0) | 0.0 (0.8) | 0.0 (0.2) | 0.0 (0.1) | 0.0 (0.1) | 0.0 (1.0) | 0.0 (0.0) | 0.0 (0.1) |
| South Thompson-Bessette Creek_SU_1.2 | 0.0 (0.3) | 0.0 (0.0) | 0.0 (0.1) | 0.0 (0.0) | 0.0 (0.0) | 0.0 (0.0) | 0.0 (0.1) | 0.0 (0.0) | 0.0 (0.0) | 0.0 (0.5) |
| South Thompson_SU_0.3 | 0.0 (1.8) | 0.0 (0.0) | 0.0 (0.1) | 0.0 (3.9) | 0.0 (0.3) | 0.0 (0.7) | 0.0 (0.0) | 2.9 (2.2) | 1.9 (1.6) | 6.5 (3.6) |
| South Thompson_SU_1.3 | 0.0 (2.4) | 0.0 (0.1) | 0.0 (0.0) | 0.0 (0.5) | 0.0 (0.0) | 0.0 (0.0) | 0.0 (0.0) | 0.0 (0.5) | 0.0 (0.0) | 0.0 (0.2) |
| Lower Thompson_SP_1.2 | 0.0 (0.4) | 0.0 (0.8) | 0.0 (0.1) | 0.0 (0.8) | 0.0 (0.0) | 0.0 (0.2) | 0.0 (0.1) | 0.0 (0.2) | 0.0 (0.2) | 0.0 (0.5) |
| Lower Fraser River_SP_1.3 | 0.0 (1.0) | 0.0 (0.0) | 0.0 (0.0) | 0.0 (0.3) | 0.0 (0.0) | 0.0 (0.0) | 0.0 (0.2) | 0.0 (0.0) | 0.0 (0.1) | 0.0 (0.0) |
| Lower Fraser River_SU_1.3 | 0.0 (0.1) | 0.0 (0.0) | 0.0 (0.0) | 0.0 (0.6) | 0.0 (0.0) | 0.0 (0.0) | 0.0 (0.0) | 0.0 (0.1) | 0.0 (0.0) | 0.0 (0.0) |
| Lower Fraser River-Upper Pitt_SU_1.3 | 0.0 (0.2) | 0.0 (0.1) | 0.0 (0.0) | 0.0 (0.1) | 0.0 (0.5) | 0.0 (0.0) | 0.0 (0.0) | 0.0 (0.0) | 0.0 (0.0) | 0.0 (0.6) |
| Maria Slough_SU_0.3 | 0.0 (0.0) | 0.0 (0.0) | 0.0 (0.0) | 0.0 (0.3) | 0.0 (0.0) | 0.0 (0.1) | 0.0 (0.0) | 0.0 (0.0) | 0.0 (0.0) | 0.0 (0.0) |
| Lower Fraser River_FA_0.3 | 0.0 (0.8) | 10.0 (4.0) | 6.4 (3.7) | 0.0 (0.3) | 38.7 (8.6) | 36.6 (4.8) | 23.0 (4.1) | 14.3 (5.0) | 35.8 (4.9) | 9.7 (3.9) |
| East Vancouver Island-North_FA_0.x | 0.0 (0.0) | 0.0 (0.0) | 0.0 (0.5) | 0.0 (1.9) | 3.2 (4.0) | 0.0 (0.3) | 0.0 (0.0) | 0.0 (0.5) | 0.0 (0.2) | 0.0 (0.2) |
| East Vancouver Island-Qualicum and Puntledge_FA_0.x | 8.8 (9.6) | 4.2 (3.0) | 18.4 (7.3) | 0.0 (3.2) | 0.4 (0.6) | 10.1 (4.0) | 4.4 (2.7) | 5.7 (3.6) | 8.6 (3.8) | 12.9 (5.8) |
| East Vancouver Island-Nanaimo and Chemainus_FA_0.x | 0.0 (0.1) | 0.0 (0.0) | 0.0 (0.0 ) | 0.0 (1.0) | 0.0 (0.0) | 1.2 (1.3) | 0.0 (0.1) | 0.0 (0.0) | 0.0 (0.3) | 0.0 (0.0) |
| East Vancouver Island-Nanaimo_SP_1.x | 0.0 (0.0) | 0.0 (0.0) | 0.0 (0.0) | 0.0 (1.1) | 0.0 (0.0) | 0.0 (0.0) | 0.0 (0.2) | 0.0 (0.0) | 0.0 (0.2) | 0.0 (0.0) |
| East Vancouver Island-Georgia Strait_SU_0.3 | 10.0 (6.4) | 0.0 (0.1) | 4.3 (3.6) | 0.0 (3.0) | 0.0 (0.1) | 2.8 (1.5) | 1.6 (1.5) | 0.0 (0.3) | 0.0 (0.2) | 0.0 (0.1) |
| East Vancouver Island-Cowichan and Koksilah_FA_0.x | 11.2 (11.5) | 5.9 (3.5) | 2.9 (2.5) | 0.0 (0.0) | 15.8 (6.1) | 4.2 (2.5) | 8.7 (3.8) | 5.8 (4.1) | 11.1 (3.6) | 0.0 (0.0) |
| West Vancouver Island-Nootka and Kyuquot_FA_0.x | 0.0 (0.8) | 0.0 (0.1) | 0.0 (0.0) | 0.0 (0.8) | 0.0 (0.5) | 0.0 (0.1) | 0.0 (0.1) | 0.0 (0.1) | 0.0 (0.4) | 0.0 (0.1) |
| West Vancouver Island-North_FA_0.x | 0.0 (1.2) | 0.0 (0.0) | 0.0 (0.3) | 0.0 (0.0) | 0.0 (0.1) | 0.0 (0.2) | 0.0 (0.0) | 0.0 (0.0) | 0.0 (0.1) | 0.0 (0.0) |
| West Vancouver Island-South_FA_0.x | 0.0 (1.2) | 0.0 (0.2) | 0.0 (0.1) | 0.0 (2.9) | 0.0 (0.6) | 0.0 (0.2) | 0.0 (0.4) | 0.0 (0.1) | 0.0 (0.3) | 0.0 (0.4) |
| Okanagan_1.x | 0.0 (0.0) | 0.0 (0.0) | 0.0 (0.0) | 0.0 (0.0) | 0.0 (0.0) | 0.0 (0.0) | 0.0 (0.0) | 0.0 (0.0) | 0.0 (0.0) | 0.0 (0.0) |
| Juan de Fuca | 0.0 (0.0) | 0.0 (0.0) | 0.0 (0.1) | 0.0 (0.1) | 0.0 (0.0) | 0.0 (0.0) | 0.0 (0.0) | 0.0 (0.0) | 0.0 (0.0) | 0.0 (0.0) |
| Coastal Washington | 0.0 (2.4) | 0.0 (0.0) | 0.0 (0.6) | 0.0 (8.0) | 0.0 (0.1) | 0.0 (0.0) | 0.0 (0.1) | 0.0 (0.1) | 0.0 (0.2) | 0.0 (0.9) |
| North Puget Sound | 16.2 (11.0) | 28.5 (5.9) | 30.1 (7.0) | 0.0 (3.8) | 13.4 (7.4) | 22.1 (5.9) | 15.3 (4.3) | 14.9 (4.4) | 9.2 (3.1) | 28.8 (8.9) |
| South Puget Sound | 53.8 (14.9) | 39.8 (5.6) | 35.1 (7.4) | 100.0 (24.7) | 18.8 (8.1) | 17.3 (4.3) | 45.1 (6.2) | 53.6 (7.2) | 27.8 (5.7) | 32.5 (6.7) |
| Lower Columbia River | 0.0 (0.7) | 0.0 (0.2) | 0.0 (0.1) | 0.0 (1.1) | 0.0 (0.1) | 0.0 (0.2) | 0.0 (0.5) | 0.0 (0.0) | 0.0 (0.0) | 3.2 (2.9) |
| Mid Columbia River_SP | 0.0 (0.0) | 7.7 (5.3) | 0.8 (1.1) | 0.0 (0.9) | 0.0 (0.0) | 0.0 (0.0) | 0.2 (1.4) | 0.0 (0.1) | 0.0 (0.1) | 0.0 (0.0) |
| Upper Columbia River_SP | 0.0 (2.1) | 0.0 (0.0) | 0.0 (0.0) | 0.0 (3.9) | 0.0 (1.0) | 0.0 (0.1) | 0.0 (0.1) | 0.0 (0.2) | 0.0 (0.1) | 0.0 (0.0) |
| Upper Columbia River_SU_FA | 0.0 (0.1) | 0.0 (0.3) | 0.0 (0.3) | 0.0 (2.6) | 0.0 (0.4) | 0.0 (0.0) | 0.0 (0.2) | 0.0 (0.1) | 0.0 (0.0) | 0.0 (1.5) |
| Snake River_FA | 0.0 (0.0) | 0.0 (0.0) | 0.0 (0.0) | 0.0 (0.0) | 0.0 (0.0) | 0.0 (0.0) | 0.0 (0.0) | 0.0 (0.0) | 0.0 (0.0) | 0.0 (0.0) |
| Snake River_SP_SU | 0.0 (1.4) | 0.0 (0.1) | 0.0 (0.8) | 0.0 (2.6) | 0.0 (0.3) | 0.0 (0.3) | 0.0 (0.4) | 0.0 (1.5) | 0.0 (0.0) | 0.0 (0.4) |
| North & Central Oregon | 0.0 (0.6) | 0.0 (0.4) | 0.0 (0.1) | 0.0 (0.0) | 0.0 (0.1) | 0.0 (0.5) | 0.0 (0.2) | 0.0 (0.1) | 0.0 (0.3) | 0.0 (0.9) |
| Upper Willamette River | 0.0 (0.0) | 0.0 (0.1) | 0.0 (0.1) | 0.0 (5.7) | 0.0 (0.2) | 0.0 (1.0) | 0.0 (0.0) | 0.0 (0.1) | 0.0 (0.0) | 0.0 (0.1) |
| South Oregon coastal | 0.0 (1.9) | 0.0 (0.0) | 0.0 (0.0) | 0.0 (2.0) | 0.0 (0.3) | 0.0 (0.2) | 0.0 (0.1) | 0.0 (0.4) | 0.0 (0.1) | 0.0 (0.1) |
| California Klamath Trinity | 0.0 (0.1) | 0.0 (0.1) | 0.0 (0.0) | 0.0 (9.8) | 0.0 (0.2) | 0.0 (0.1) | 0.0 (0.0) | 0.0 (0.0) | 0.0 (0.0) | 0.0 (0.0) |
| California Central Valley_Fall | 0.0 (1.8) | 0.0 (0.1) | 0.0 (0.2) | 0.0 (4.4) | 0.0 (0.0) | 0.0 (0.3) | 0.0 (0.2) | 0.0 (0.5) | 0.0 (0.1) | 0.0 (0.1) |
| California Central Valley_Spring | 0.0 (0.0) | 0.0 (0.0) | 0.0 (0.5) | 0.0 (0.0) | 0.0 (0.0) | 0.0 (0.0) | 0.0 (0.1) | 0.0 (0.3) | 0.0 (0.0) | 0.0 (0.1) |
| Coastal California | 0.0 (0.0) | 0.0 (0.0) | 0.0 (0.0) | 0.0 (0.9) | 0.0 (0.0) | 0.0 (0.0) | 0.0 (0.0) | 0.0 (0.0) | 0.0 (0.0) | 0.0 (0.0) |

Supplementary Table S5 continued

| Conservation Unit | Strait of Georgia-south sport | | | | | | | | | | |
| --- | --- | --- | --- | --- | --- | --- | --- | --- | --- | --- | --- |
|  | June | July | | August | | | September | | | October | |
|  | Unknown | Legal | Sublegal | Legal | Sublegal | Unknown | Legal | Sublegal | Unknown | Legal | Sublegal |
| Sample size | 13 | 177 | 74 | 302 | 88 | 16 | 68 | 10 | 8 | 2 | 10 |
| N PBT | 4 | 32 | 14 | 34 | 16 | 2 | 29 | 1 | 1 | 1 | 0 |
| Southeast Alaska | 0.0 (0.3) | 0.0 (0.0) | 0.0 (0.2) | 0.0 (0.0) | 0.0 (0.2) | 0.0 (1.3) | 0.0 (0.3) | 0.0 (0.3) | 0.0 (0.6) | 0.0 (0.2) | 0.0 (0.4) |
| Alsek | 0.0 (1.0) | 0.0 (0.0) | 0.0 (0.0) | 0.0 (0.0) | 0.0 (0.1) | 0.0 (0.2) | 0.0 (0.4) | 0.0 (0.1) | 0.0 (0.2) | 0.0 (0.3) | 0.0 (0.5) |
| Unuk | 0.0 (0.1) | 0.0 (0.0) | 0.0 (0.3) | 0.0 (0.0) | 0.0 (0.0) | 0.0 (0.0) | 0.0 (0.0) | 0.0 (0.0) | 0.0 (0.0) | 0.0 (1.0) | 0.0 (0.8) |
| Taku_early timing | 0.0 (0.2) | 0.0 (0.0) | 0.0 (0.1) | 0.0 (0.1) | 0.0 (0.2) | 0.0 (1.7) | 0.0 (0.0) | 0.0 (0.0) | 0.0 (1.4) | 0.0 (0.2) | 0.0 (0.0) |
| Taku_mid timing | 0.0 (1.1) | 0.0 (0.0) | 0.0 (0.0) | 0.0 (0.0) | 0.0 (0.0) | 0.0 (0.1) | 0.0 (0.1) | 0.0 (1.1) | 0.0 (0.2) | 0.0 (0.0) | 0.0 (0.2) |
| Taku_late timing | 0.0 (0.0) | 0.0 (0.0) | 0.0 (0.1) | 0.0 (0.0) | 0.0 (0.0) | 0.0 (0.0) | 0.0 (0.2) | 0.0 (0.9) | 0.0 (1.2) | 0.0 (0.7) | 0.0 (1.8) |
| Stikine_early timing | 0.0 (0.4) | 0.0 (0.1) | 0.0 (0.4) | 0.0 (0.0) | 0.0 (0.0) | 0.0 (0.2) | 0.0 (0.0) | 0.0 (3.5) | 0.0 (3.2) | 0.0 (4.6) | 0.0 (0.1) |
| Stikine_late timing | 0.0 (0.2) | 0.0 (0.0) | 0.0 (0.0) | 0.0 (0.0) | 0.0 (0.0) | 0.0 (1.3) | 0.0 (0.0) | 0.0 (0.9) | 0.0 (0.0) | 0.0 (0.0) | 0.0 (0.1) |
| Haida Gwaii-North | 0.0 (0.0) | 0.0 (0.1) | 0.0 (0.0) | 0.0 (0.0) | 0.0 (0.0) | 0.0 (0.0) | 0.0 (0.0) | 0.0 (1.6) | 0.0 (0.0) | 0.0 (1.1) | 0.0 (0.1) |
| Upper Nass | 0.0 (0.6) | 0.0 (0.0) | 0.0 (0.4) | 0.0 (0.0) | 0.0 (0.0) | 0.0 (0.1) | 0.0 (0.4) | 0.0 (0.7) | 0.0 (0.4) | 0.0 (7.0) | 0.0 (1.4) |
| Portland Sound-Observatory Inlet-Lower Nass | 0.0 (0.5) | 0.0 (0.1) | 0.0 (0.2) | 0.0 (0.1) | 0.0 (0.0) | 0.0 (0.0) | 0.0 (0.0) | 0.0 (0.0) | 0.0 (0.1) | 0.0 (4.9) | 0.0 (0.3) |
| Ecstall | 0.0 (0.3) | 0.0 (0.0) | 0.0 (0.0) | 0.0 (0.0) | 0.0 (0.1) | 0.0 (0.0) | 0.0 (0.0) | 0.0 (0.9) | 0.0 (0.0) | 0.0 (1.2) | 0.0 (0.0) |
| Skeena Estuary | 0.0 (0.0) | 0.0 (0.1) | 0.0 (0.0) | 0.0 (0.0) | 0.0 (0.1) | 0.0 (0.0) | 0.0 (0.0) | 0.0 (0.0) | 0.0 (0.4) | 0.0 (0.0) | 0.0 (0.3) |
| Lower Skeena | 0.0 (0.4) | 0.0 (0.0) | 0.0 (0.0) | 0.0 (0.0) | 0.0 (0.1) | 0.0 (0.7) | 0.0 (0.1) | 0.0 (0.1) | 0.0 (0.1) | 0.0 (4.4) | 0.0 (0.1) |
| Kalum_early timing | 0.0 (0.2) | 0.0 (0.0) | 0.0 (0.0) | 0.0 (0.0) | 0.0 (0.0) | 0.0 (0.0) | 0.0 (0.0) | 0.0 (0.0) | 0.0 (0.4) | 0.0 (0.2) | 0.0 (0.0) |
| Kalum_late timing | 0.0 (0.0) | 0.0 (0.0) | 0.0 (0.0) | 0.0 (0.0) | 0.0 (0.0) | 0.0 (0.0) | 0.0 (0.2) | 0.0 (0.0) | 0.0 (0.0) | 0.0 (0.4) | 0.0 (0.0) |
| Zymoetz | 0.0 (0.2) | 0.0 (0.0) | 0.0 (0.0) | 0.0 (0.0) | 0.0 (0.0) | 0.0 (0.6) | 0.0 (0.1) | 0.0 (0.0) | 0.0 (0.0) | 0.0 (0.0) | 0.0 (0.0) |
| Sicintine | 0.0 (0.0) | 0.0 (0.0) | 0.0 (0.0) | 0.0 (0.0) | 0.0 (0.0) | 0.0 (0.1) | 0.0 (0.0) | 0.0 (0.0) | 0.0 (0.0) | 0.0 (0.0) | 0.0 (0.0) |
| Middle Skeena-mainstem tributaries | 0.0 (0.8) | 0.0 (0.0) | 0.0 (0.3) | 0.0 (0.1) | 0.0 (0.3) | 0.0 (1.6) | 0.0 (0.2) | 0.0 (0.4) | 0.0 (0.7) | 0.0 (1.8) | 0.0 (1.2) |
| Middle Skeena-large lakes | 0.0 (0.1) | 0.0 (0.1) | 0.0 (0.1) | 0.0 (0.0) | 0.0 (0.0) | 0.0 (0.0) | 0.0 (0.0) | 0.0 (0.1) | 0.0 (0.0) | 0.0 (0.4) | 0.0 (0.2) |
| Upper Skeena | 0.0 (0.1) | 0.0 (0.1) | 0.0 (0.1) | 0.0 (0.1) | 0.0 (0.0) | 0.0 (0.0) | 0.0 (0.0) | 0.0 (0.4) | 0.0 (2.2) | 0.0 (7.6) | 0.0 (0.3) |
| Upper Bulkley River | 0.0 (0.9) | 0.0 (0.0) | 0.0 (0.0) | 0.0 (0.0) | 0.0 (0.0) | 0.0 (0.0) | 0.0 (0.0) | 0.0 (0.0) | 0.0 (1.5) | 0.0 (0.0) | 0.0 (0.0) |
| North and Central Coast-late timing | 0.0 (0.4) | 0.0 (0.0) | 0.0 (0.0) | 0.0 (0.0) | 0.0 (0.0) | 0.0 (0.0) | 0.0 (0.0) | 0.0 (0.2) | 0.0 (0.1) | 0.0 (2.0) | 0.0 (0.1) |
| North and Central Coast-early timing | 0.0 (0.0) | 0.0 (0.0) | 0.0 (0.0) | 0.0 (0.0) | 0.0 (0.0) | 0.0 (0.0) | 0.0 (0.0) | 0.0 (0.0) | 0.0 (0.3) | 0.0 (0.0) | 0.0 (0.0) |
| Rivers Inlet | 0.0 (0.8) | 0.0 (0.1) | 0.0 (0.0) | 0.0 (0.0) | 0.0 (0.0) | 0.0 (0.0) | 0.0 (0.0) | 0.0 (0.3) | 0.0 (0.3) | 0.0 (6.6) | 0.0 (1.7) |
| Wannock | 0.0 (0.0) | 0.0 (0.0) | 0.0 (0.0) | 0.0 (0.0) | 0.0 (0.0) | 0.0 (0.1) | 0.0 (0.0) | 0.0 (0.0) | 0.0 (0.0) | 0.0 (0.0) | 0.0 (0.0) |
| Bella Coola-Bentinck | 0.0 (0.1) | 0.0 (0.0) | 0.0 (0.0) | 0.0 (0.0) | 0.0 (0.0) | 0.0 (0.0) | 0.0 (0.2) | 0.0 (0.0) | 0.0 (2.9) | 0.0 (4.5) | 0.0 (0.5) |
| Dean River | 0.0 (0.3) | 0.0 (0.0) | 0.0 (0.0) | 0.0 (0.0) | 0.0 (0.2) | 0.0 (0.2) | 0.0 (0.0) | 0.0 (0.1) | 0.0 (0.3) | 0.0 (0.0) | 0.0 (2.9) |
| Docee | 0.0 (0.2) | 0.0 (0.0) | 0.0 (0.0) | 0.0 (0.0) | 0.0 (0.1) | 0.0 (0.0) | 0.0 (0.0) | 0.0 (0.2) | 0.0 (0.0) | 0.0 (0.0) | 0.0 (0.0) |
| Klinaklini_SU_1.3 | 0.0 (2.2) | 0.0 (0.0) | 0.0 (0.0) | 0.1 (0.2) | 0.0 (0.2) | 0.0 (0.0) | 0.0 (0.0) | 0.0 (0.1) | 0.0 (0.8) | 0.0 (0.0) | 0.0 (0.0) |
| Southern Mainland-Southern Fjords_FA_0.x | 0.0 (0.0) | 0.0 (0.0) | 0.0 (0.0) | 0.0 (0.0) | 0.0 (0.0) | 0.0 (0.0) | 0.0 (0.1) | 0.0 (0.0) | 0.0 (0.1) | 0.0 (0.0) | 0.0 (0.0) |
| Southern Mainland-Georgia Strait_FA_0.x | 0.0 (0.7) | 0.6 (0.6) | 1.4 (1.1) | 1.0 (0.5) | 2.3 (2.0) | 0.0 (0.2) | 0.0 (0.1) | 10.0 (9.1) | 0.0 (1.1) | 0.0 (3.6) | 0.0 (0.5) |
| Upper Fraser River_SP_1.3 | 0.0 (0.8) | 0.0 (0.0) | 0.0 (0.1) | 0.0 (0.0) | 4.2 (2.3) | 0.0 (2.2) | 0.0 (0.7) | 0.0 (0.3) | 0.0 (1.6) | 0.0 (3.4) | 10.0 (7.7) |
| Middle Fraser River_SU_1.3 | 0.0 (0.7) | 0.6 (0.6) | 0.0 (0.0) | 0.4 (0.4) | 0.9 (1.2) | 0.0 (0.1) | 0.0 (0.0) | 0.0 (0.2) | 0.0 (0.0) | 0.0 (6.4) | 10.0 (9.7) |
| Middle Fraser River_SP_1.3 | 0.0 (0.3) | 0.0 (0.1) | 0.0 (0.4) | 0.6 (0.5) | 1.7 (1.9) | 0.0 (1.5) | 0.0 (0.2) | 0.0 (0.4) | 0.0 (8.2) | 0.0 (6.2) | 0.0 (1.7) |
| Middle Fraser River-Portage_FA_1.3 | 0.0 (0.1) | 0.0 (0.0) | 0.0 (0.0) | 0.0 (0.0) | 0.0 (0.0) | 0.0 (0.0) | 0.0 (0.0) | 0.0 (0.0) | 0.0 (0.0) | 0.0 (0.0) | 0.0 (0.4) |
| Middle Fraser-Fraser Canyon_SP_1.3 | 0.0 (0.2) | 0.0 (0.0) | 0.0 (0.0) | 0.0 (0.0) | 0.0 (0.0) | 0.0 (0.0) | 0.0 (0.0) | 0.0 (0.1) | 0.0 (0.0) | 0.0 (0.0) | 0.0 (0.3) |
| North Thompson_SP_1.3 | 0.0 (0.3) | 0.0 (0.0) | 0.0 (0.0) | 0.3 (0.5) | 0.0 (0.0) | 0.0 (0.3) | 0.0 (0.0) | 0.0 (0.0) | 0.0 (1.4) | 0.0 (1.3) | 0.0 (0.4) |
| North Thompson_SU_1.3 | 0.0 (0.2) | 0.0 (0.0) | 1.4 (1.1) | 0.0 (0.2) | 3.5 (1.7) | 6.3 (5.9) | 0.0 (0.1) | 0.0 (0.1) | 0.0 (0.2) | 0.0 (0.1) | 0.0 (0.6) |
| Shuswap River_SU_0.3 | 0.0 (0.5) | 1.1 (0.8) | 0.0 (0.0) | 2.2 (1.0) | 5.6 (2.0) | 0.0 (0.1) | 0.0 (0.0) | 0.0 (0.0) | 0.0 (0.0) | 0.0 (0.0) | 0.0 (0.0) |
| South Thompson-Bessette Creek_SU_1.2 | 0.0 (0.0) | 0.0 (0.0) | 0.0 (0.0) | 0.0 (0.0) | 0.0 (0.0) | 0.0 (0.0) | 0.0 (0.8) | 0.0 (0.4) | 0.0 (0.0) | 0.0 (0.0) | 0.0 (0.0) |
| South Thompson_SU_0.3 | 0.0 (2.2) | 2.3 (1.3) | 1.4 (1.3) | 61.7 (2.8) | 25.0 (4.5) | 43.8 (12.3) | 23.5 (4.7) | 20.0 (9.7) | 12.5 (10.0) | 0.0 (0.1) | 10.0 (8.7) |
| South Thompson_SU_1.3 | 0.0 (0.1) | 0.0 (0.0) | 0.0 (0.0) | 0.0 (0.0) | 0.0 (0.0) | 0.0 (0.2) | 0.0 (0.0) | 0.0 (0.0) | 0.0 (0.2) | 0.0 (0.2) | 0.0 (0.5) |
| Lower Thompson_SP_1.2 | 0.0 (0.3) | 0.6 (0.6) | 0.0 (0.0) | 0.0 (0.0) | 1.1 (0.8) | 0.0 (0.2) | 0.0 (0.0) | 0.0 (0.9) | 0.0 (0.8) | 0.0 (2.2) | 0.0 (1.2) |
| Lower Fraser River_SP_1.3 | 0.0 (0.0) | 0.0 (0.0) | 0.0 (0.0) | 0.0 (0.1) | 0.0 (0.0) | 0.0 (0.0) | 0.0 (0.6) | 0.0 (0.0) | 0.0 (0.0) | 0.0 (0.7) | 0.0 (0.0) |
| Lower Fraser River_SU_1.3 | 0.0 (0.4) | 0.0 (0.0) | 0.0 (0.2) | 0.0 (0.0) | 0.0 (0.1) | 0.0 (1.6) | 0.0 (0.0) | 0.0 (0.0) | 0.0 (0.0) | 0.0 (0.6) | 0.0 (0.0) |
| Lower Fraser River-Upper Pitt_SU_1.3 | 0.0 (0.1) | 0.0 (0.0) | 0.0 (0.1) | 0.3 (0.3) | 0.0 (0.0) | 0.0 (0.3) | 0.0 (0.0) | 0.0 (0.0) | 0.0 (0.0) | 0.0 (1.3) | 0.0 (0.0) |
| Maria Slough_SU_0.3 | 0.0 (0.1) | 0.0 (0.0) | 0.0 (0.0) | 0.0 (0.0) | 0.0 (0.0) | 0.0 (0.0) | 0.0 (0.0) | 0.0 (0.1) | 0.0 (0.0) | 0.0 (0.1) | 0.0 (0.0) |
| Lower Fraser River_FA_0.3 | 38.5 (11.9) | 26.0 (2.9) | 21.4 (4.9) | 11.3 (1.9) | 21.6 (5.1) | 0.0 (0.2) | 47.1 (5.9) | 20.0 (10.4) | 25.0 (14.0) | 50.0 (25.4) | 0.0 (0.2) |
| East Vancouver Island-North_FA_0.x | 0.0 (1.0) | 0.0 (0.0) | 0.0 (0.3) | 1.0 (0.6) | 0.0 (0.1) | 0.0 (0.1) | 1.5 (1.5) | 0.0 (0.0) | 0.0 (0.1) | 0.0 (0.2) | 0.0 (0.3) |
| East Vancouver Island-Qualicum and Puntledge_FA_0.x | 14.7 (8.2) | 9.2 (2.1) | 11.1 (3.8) | 5.5 (1.4) | 13.8 (4.2) | 18.1 (9.6) | 14.3 (4.2) | 19.3 (12.4) | 20.4 (12.0) | 0.0 (5.9) | 0.0 (2.3) |
| East Vancouver Island-Nanaimo and Chemainus_FA_0.x | 0.0 (2.4) | 0.0 (0.0) | 0.0 (0.2) | 0.1 (0.2) | 0.0 (0.0) | 0.0 (0.0) | 0.0 (0.2) | 0.0 (0.0) | 0.0 (0.0) | 0.0 (2.0) | 0.0 (0.8) |
| East Vancouver Island-Nanaimo_SP_1.x | 0.0 (0.0) | 0.0 (0.0) | 0.0 (0.0) | 0.0 (0.0) | 0.0 (0.0) | 0.0 (0.0) | 0.0 (0.0) | 0.0 (0.2) | 0.0 (0.1) | 0.0 (0.0) | 0.0 (0.7) |
| East Vancouver Island-Georgia Strait_SU_0.3 | 0.0 (0.1) | 0.6 (0.7) | 4.1 (2.2) | 0.0 (0.0) | 2.5 (1.8) | 0.0 (0.1) | 1.5 (1.2) | 0.0 (0.6) | 0.0 (0.0) | 0.0 (0.5) | 30.0 (10.7) |
| East Vancouver Island-Cowichan and Koksilah_FA_0.x | 0.7 (4.5) | 10.0 (2.5) | 6.5 (2.1) | 6.1 (1.3) | 3.0 (2.2) | 0.6 (1.8) | 4.8 (2.6) | 10.7 (9.5) | 17.1 (10.9) | 0.0 (3.8) | 10.0 (11.7) |
| West Vancouver Island-Nootka and Kyuquot_FA_0.x | 0.0 (0.3) | 0.5 (0.5) | 0.0 (0.1) | 0.0 (0.1) | 0.0 (0.1) | 0.0 (1.0) | 0.0 (0.2) | 0.0 (0.3) | 0.0 (3.8) | 0.0 (3.3) | 0.0 (0.2) |
| West Vancouver Island-North_FA_0.x | 0.0 (0.0) | 0.0 (0.0) | 0.0 (0.0) | 0.0 (0.0) | 0.0 (0.0) | 0.0 (0.0) | 0.0 (0.0) | 0.0 (0.0) | 0.0 (0.0) | 0.0 (0.6) | 0.0 (0.0) |
| West Vancouver Island-South_FA_0.x | 0.0 (0.5) | 0.1 (0.4) | 0.0 (0.0) | 0.7 (0.6) | 0.0 (0.2) | 0.0 (0.7) | 1.5 (1.8) | 0.0 (1.3) | 25.0 (12.7) | 0.0 (6.1) | 0.0 (1.0) |
| Okanagan_1.x | 0.0 (0.1) | 0.0 (0.0) | 2.1 (2.2) | 0.0 (0.0) | 0.0 (0.0) | 0.0 (0.2) | 0.0 (0.0) | 0.0 (0.0) | 0.0 (1.9) | 0.0 (0.0) | 0.0 (0.0) |
| Juan de Fuca | 0.0 (0.3) | 0.0 (0.0) | 0.0 (0.1) | 0.0 (0.0) | 0.0 (0.0) | 0.0 (0.0) | 0.0 (0.1) | 0.0 (0.2) | 0.0 (0.0) | 0.0 (0.0) | 0.0 (0.0) |
| Coastal Washington | 0.0 (0.1) | 0.0 (0.0) | 0.0 (0.0) | 0.0 (0.1) | 0.0 (0.2) | 0.0 (0.3) | 0.0 (0.1) | 0.0 (0.1) | 0.0 (0.9) | 0.0 (0.0) | 0.0 (1.3) |
| North Puget Sound | 0.0 (0.0) | 9.7 (1.6) | 15.0 (4.0) | 1.3 (0.5) | 9.5 (2.8) | 6.4 (6.7) | 2.8 (2.4) | 0.0 (0.1) | 0.0 (0.6) | 50.0 (27.2) | 0.0 (0.3) |
| South Puget Sound | 46.1 (12.8) | 37.8 (3.2) | 35.1 (5.8) | 7.3 (1.3) | 5.1 (2.4) | 18.6 (10.7) | 2.9 (2.0) | 20.0 (9.9) | 0.0 (0.1) | 0.0 (0.0) | 30.0 (10.9) |
| Lower Columbia River | 0.0 (0.0) | 0.0 (0.0) | 0.0 (0.0) | 0.0 (0.0) | 0.0 (0.0) | 0.0 (0.0) | 0.0 (0.4) | 0.0 (0.0) | 0.0 (0.0) | 0.0 (1.2) | 0.0 (0.3) |
| Mid Columbia River_SP | 0.0 (0.3) | 1.2 (1.5) | 0.0 (0.1) | 0.0 (0.0) | 0.2 (0.5) | 6.3 (9.3) | 0.2 (0.6) | 0.0 (0.3) | 0.0 (0.3) | 0.0 (5.8) | 0.0 (0.2) |
| Upper Columbia River_SP | 0.0 (0.3) | 0.0 (0.1) | 0.0 (0.0) | 0.0 (0.0) | 0.0 (0.0) | 0.0 (0.2) | 0.0 (0.1) | 0.0 (0.1) | 0.0 (0.3) | 0.0 (4.4) | 0.0 (0.2) |
| Upper Columbia River_SU_FA | 0.0 (0.4) | 0.0 (0.0) | 0.2 (0.8) | 0.0 (0.0) | 0.0 (0.1) | 0.0 (0.1) | 0.0 (0.0) | 0.0 (2.8) | 0.0 (2.7) | 0.0 (0.0) | 0.0 (0.0) |
| Snake River_FA | 0.0 (0.0) | 0.0 (0.0) | 0.4 (1.4) | 0.0 (0.0) | 0.0 (0.1) | 0.0 (0.0) | 0.0 (0.0) | 0.0 (0.0) | 0.0 (0.0) | 0.0 (0.0) | 0.0 (1.4) |
| Snake River_SP_SU | 0.0 (1.7) | 0.0 (0.1) | 0.0 (0.3) | 0.0 (0.2) | 0.0 (0.0) | 0.0 (1.2) | 0.0 (0.2) | 0.0 (0.1) | 0.0 (4.8) | 0.0 (7.2) | 0.0 (0.8) |
| North & Central Oregon | 0.0 (0.1) | 0.0 (0.0) | 0.0 (0.0) | 0.0 (0.1) | 0.0 (0.1) | 0.0 (0.2) | 0.0 (0.1) | 0.0 (1.6) | 0.0 (0.3) | 0.0 (3.1) | 0.0 (0.6) |
| Upper Willamette River | 0.0 (1.9) | 0.0 (0.0) | 0.0 (0.0) | 0.0 (0.0) | 0.0 (0.0) | 0.0 (0.0) | 0.0 (0.0) | 0.0 (0.0) | 0.0 (0.0) | 0.0 (1.1) | 0.0 (0.2) |
| South Oregon coastal | 0.0 (0.2) | 0.0 (0.0) | 0.0 (0.1) | 0.0 (0.0) | 0.0 (0.2) | 0.0 (0.7) | 0.0 (0.4) | 0.0 (0.9) | 0.0 (1.8) | 0.0 (5.4) | 0.0 (1.0) |
| California Klamath Trinity | 0.0 (0.6) | 0.0 (0.0) | 0.0 (0.0) | 0.0 (0.0) | 0.0 (0.0 | 0.0 (0.0) | 0.0 (0.0) | 0.0 (0.8) | 0.0 (0.1) | 0.0 (1.3) | 0.0 (0.1) |
| California Central Valley_Fall | 0.0 (0.2) | 0.0 (0.0) | 0.0 (0.1) | 0.0 (0.0) | 0.0 (0.1) | 0.0 (0.3) | 0.0 (0.1) | 0.0 (1.1) | 0.0 (0.9) | 0.0 (1.7) | 0.0 (1.2) |
| California Central Valley_Spring | 0.0 (0.0) | 0.0 (0.0) | 0.0 (0.0) | 0.0 (0.0) | 0.0 (0.0) | 0.0 (0.1) | 0.0 (0.0) | 0.0 (0.0) | 0.0 (1.1) | 0.0 (0.5) | 0.0 (0.7) |
| Coastal California | 0.0 (0.0) | 0.0 (0.0) | 0.0 (0.0) | 0.0 (0.0) | 0.0 (0.0) | 0.0 90.0) | 0.0 (0.0) | 0.0 (0.0) | 0.0 (0.4) | 0.0 (0.0) | 0.0 (0.0) |

Supplementary Table S5 continued

| Conservation Unit | Juan de Fuca Strait sport | | | | WCVI-north sport | | | WCVI-south sport | | |
| --- | --- | --- | --- | --- | --- | --- | --- | --- | --- | --- |
|  | April | August | September | October | June | July | August | June | July | August |
| Sample size | 4 | 263 | 29 | 6 | 203 | 1,582 | 900 | 119 | 349 | 913 |
| N PBT | 0 | 5 | 5 | 0 | 54 | 282 | 172 | 18 | 60 | 326 |
| Southeast Alaska | 0.0 (0.7) | 0.0 (0.0) | 0.0 (0.2) | 0.0 (0.6) | 0.0 (0.0) | 0.0 (0.0) | 0.0 (0.0) | 0.0 (0.1) | 0.0 (0.0) | 0.0 (0.0) |
| Alsek | 0.0 (7.7) | 0.0 (0.0) | 0.0 (0.0) | 0.0 (1.3) | 0.0 (0.1) | 0.0 (0.0) | 0.0 (0.0) | 0.0 (0.0) | 0.0 (0.0) | 0.0 (0.0) |
| Unuk | 0.0 (0.0) | 0.0 (0.0) | 0.0 (0.0) | 0.0 (0.0) | 0.0 (0.0) | 0.0 (0.0) | 0.0 (0.0) | 0.0 (0.0) | 0.0 (0.0) | 0.0 (0.0) |
| Taku_early timing | 0.0 (0.2) | 0.0 (0.0) | 0.0 (0.5) | 0.0 (0.0) | 0.0 (0.0) | 0.0 (0.0) | 0.0 (0.0) | 0.0 (0.1) | 0.0 (0.0) | 0.0 (0.0) |
| Taku_mid timing | 0.0 (0.2) | 0.0 (0.1) | 0.0 (0.5) | 0.0 (0.1) | 0.0 (0.0) | 0.0 (0.0) | 0.0 (0.0) | 0.0 (0.0) | 0.0 (0.0) | 0.0 (0.0) |
| Taku_late timing | 0.0 (0.8) | 0.0 (0.0) | 0.0 (0.8) | 0.0 (0.0) | 0.0 (0.0) | 0.0 (0.0) | 0.0 (0.0) | 0.0 (0.0) | 0.0 (0.0) | 0.0 (0.0) |
| Stikine_early timing | 0.0 (1.0) | 0.0 (0.0) | 0.0 (0.3) | 0.0 (0.2) | 0.0 (0.0) | 0.0 (0.0) | 0.0 (0.0) | 0.0 (0.0) | 0.0 (0.0) | 0.0 (0.0) |
| Stikine_late timing | 0.0 (2.6) | 0.0 (0.0) | 0.0 (0.1) | 0.0 (1.2) | 0.0 (0.1) | 0.0 (0.0) | 0.0 (0.0) | 0.0 (0.0) | 0.0 (0.0) | 0.0 (0.0) |
| Haida Gwaii-North | 0.0 (0.1) | 0.0 (0.0) | 0.0 (0.0) | 0.0 (0.0) | 0.0 (0.0) | 0.0 (0.0) | 0.0 (0.0) | 0.0 (0.0) | 0.0 (0.0) | 0.0 (0.0) |
| Upper Nass | 0.0 (1.0) | 0.0 (0.0) | 0.0 (0.7) | 0.0 (0.0) | 0.0 (0.1) | 0.0 (0.0) | 0.0 (0.0) | 0.0 (0.1) | 0.0 (0.0) | 0.0 (0.0) |
| Portland Sound-Observatory Inlet-Lower Nass | 0.0 (0.4) | 0.0 (0.0) | 0.0 (0.0) | 0.0 (0.4) | 0.0 (0.0) | 0.0 (0.1) | 0.0 (0.0) | 0.0 (0.0) | 0.0 (0.0) | 0.0 (0.0) |
| Ecstall | 0.0 (0.1) | 0.0 (0.0) | 0.0 (0.1) | 0.0 (1.0) | 0.0 (0.0) | 0.0 (0.0) | 0.0 (0.0) | 0.0 (0.0) | 0.0 (0.0) | 0.0 (0.0) |
| Skeena Estuary | 0.0 (0.0) | 0.0 (0.0) | 0.0 (0.0) | 0.0 (0.0) | 0.0 (0.0) | 0.0 (0.0) | 0.0 (0.0) | 0.0 (0.0) | 0.0 (0.0) | 0.0 (0.0) |
| Lower Skeena | 0.0 (3.3) | 0.0 (0.0) | 0.0 (0.2) | 0.0 (0.0) | 0.0 (0.0) | 0.0 (0.0) | 0.0 (0.0) | 0.0 (0.2) | 0.0 (0.0) | 0.0 (0.0) |
| Kalum_early timing | 0.0 (0.4) | 0.0 (0.0) | 0.0 (0.0) | 0.0 (0.0) | 0.0 (0.0) | 0.0 (0.0) | 0.0 (0.0) | 0.0 (0.0) | 0.0 (0.0) | 0.0 (0.0) |
| Kalum_late timing | 0.0 (0.0) | 0.0 (0.0) | 0.0 (0.0) | 0.0 (5.4) | 0.0 (0.0) | 0.1 (0.0) | 0.0 (0.0) | 0.0 (0.0) | 0.0 (0.0) | 0.0 (0.0) |
| Zymoetz | 0.0 (0.0) | 0.0 (0.0) | 0.0 (0.0) | 0.0 (0.0) | 0.0 (0.0) | 0.0 (0.0) | 0.0 (0.0) | 0.0 (0.0) | 0.0 (0.0) | 0.0 (0.0) |
| Sicintine | 0.0 (0.0) | 0.0 (0.0) | 0.0 (0.0) | 0.0 (0.0) | 0.0 (0.0) | 0.0 (0.0) | 0.0 (0.0) | 0.0 (0.0) | 0.0 (0.0) | 0.0 (0.0) |
| Middle Skeena-mainstem tributaries | 0.0 (4.7) | 0.0 (0.2) | 0.0 (0.1) | 0.0 (0.2) | 0.0 (0.0) | 0.0 (0.0) | 0.0 (0.0) | 0.0 (0.2) | 0.0 (0.0) | 0.0 (0.0) |
| Middle Skeena-large lakes | 0.0 (0.3) | 0.0 (0.1) | 0.0 (0.1) | 0.0 (0.6) | 0.0 (0.0) | 0.0 (0.0) | 0.0 (0.0) | 0.0 (0.3) | 0.0 (0.0) | 0.0 (0.0) |
| Upper Skeena | 0.0 (0.4) | 0.0 (0.1) | 0.0 (0.3) | 0.0 (0.5) | 0.0 (0.0) | 0.0 (0.0) | 0.0 (0.0) | 0.0 (0.2) | 0.0 (0.1) | 0.0 (0.0) |
| Upper Bulkley River | 0.0 (0.0) | 0.0 (0.0) | 0.0 (0.0) | 0.0 (0.0) | 0.0 (0.1) | 0.0 (0.0) | 0.0 (0.0) | 0.0 (0.0) | 0.0 (0.0) | 0.0 (0.0) |
| North and Central Coast-late timing | 0.0 (0.0) | 0.0 (0.1) | 0.0 (0.1) | 0.0 (0.0) | 0.0 (0.9) | 0.0 (0.3) | 0.0 (0.5) | 0.0 (0.0) | 0.0 (0.3) | 0.0 (0.2) |
| North and Central Coast-early timing | 0.0 (0.0) | 0.0 (0.0) | 0.0 (0.1) | 0.0 (0.0) | 0.0 (0.0) | 0.0 (0.0) | 0.0 (0.0) | 0.0 (0.0) | 0.0 (0.0) | 0.0 (0.0) |
| Rivers Inlet | 0.0 (0.1) | 0.0 (0.0) | 0.0 (0.0) | 0.0 (2.3) | 0.0 (0.0) | 0.0 (0.0) | 0.0 (0.0) | 0.0 (0.0) | 0.0 (0.0) | 0.0 (0.0) |
| Wannock | 0.0 (1.5) | 0.0 (0.0) | 0.0 (0.0) | 0.0 (0.0) | 0.0 (0.0) | 0.0 (0.0) | 0.0 (0.0) | 0.0 (0.0) | 0.0 (0.0) | 0.0 (0.0) |
| Bella Coola-Bentinck | 0.0 (0.3) | 0.0 (0.1) | 0.0 (0.4) | 0.0 (2.9) | 1.0 (0.6) | 0.3 (0.1) | 0.0 (0.0) | 0.0 (0.0) | 0.3 (0.3) | 0.0 (0.0) |
| Dean River | 0.0 (1.1) | 0.0 (0.0) | 0.0 (0.1) | 0.0 (0.1) | 0.0 (0.0) | 0.0 (0.0) | 0.0 (0.0) | 0.0 (0.0) | 0.0 (0.0) | 0.0 (0.0) |
| Docee | 0.0 (0.1) | 0.0 (0.0) | 0.0 (0.0) | 0.0 (1.9) | 0.0 (0.0) | 0.0 (0.0) | 0.0 (0.0) | 0.0 (0.0) | 0.0 (0.0) | 0.0 (0.0) |
| Klinaklini_SU_1.3 | 0.0 (2.2) | 0.0 (0.0) | 0.0 (0.1) | 0.0 (0.3) | 0.0 (0.1) | 0.1 (0.1) | 0.1 (0.1) | 0.0 (0.1) | 0.0 (0.0) | 0.0 (0.0) |
| Southern Mainland-Southern Fjords_FA_0.x | 0.0 (1.6) | 0.0 (0.1) | 0.0 (0.0) | 0.0 (0.0) | 0.0 (0.0) | 0.0 (0.0) | 0.0 (0.0) | 0.0 (0.0) | 0.0 (0.0) | 0.0 (0.0) |
| Southern Mainland-Georgia Strait_FA_0.x | 0.0 (1.2) | 0.1 (0.3) | 0.0 (0.5) | 0.0 (1.6) | 0.0 (0.1) | 0.0 (0.0) | 0.0 (0.0) | 0.0 (0.0) | 0.0 (0.0) | 0.0 (0.0) |
| Upper Fraser River_SP_1.3 | 0.0 (2.0) | 0.0 (0.1) | 0.0 (0.9) | 0.0 (1.1) | 0.0 (0.0) | 0.0 (0.0) | 0.0 (0.0) | 2.5 (1.4) | 0.0 (0.0) | 0.0 (0.0) |
| Middle Fraser River_SU_1.3 | 0.0 (1.8) | 0.0 (0.1) | 0.0 (0.1) | 0.0 (0.6) | 0.0 (0.0) | 0.1 (0.1) | 0.7 (0.3) | 0.0 (0.0) | 0.0 (0.0) | 0.1 (0.1) |
| Middle Fraser River_SP_1.3 | 0.0 (2.6) | 0.0 (0.1) | 0.0 (0.6) | 0.0 (4.1) | 0.0 (0.0) | 0.0 (0.0) | 0.0 (0.0) | 0.0 (0.1) | 0.3 (0.3) | 0.0 (0.0) |
| Middle Fraser River-Portage_FA_1.3 | 0.0 (2.9) | 0.4 (0.3) | 0.0 (0.0) | 0.0 (0.0) | 0.0 (0.0) | 0.0 (0.0) | 0.0 (0.0) | 0.0 (0.0) | 0.0 (0.0) | 0.0 (0.0) |
| Middle Fraser-Fraser Canyon_SP_1.3 | 0.0 (0.0) | 0.0 (0.0) | 0.0 (0.0) | 0.0 (0.0) | 0.0 (0.0) | 0.0 (0.0) | 0.0 (0.0) | 0.0 (0.0) | 0.0 (0.0) | 0.0 (0.0) |
| North Thompson_SP_1.3 | 0.0 (0.0) | 0.1 (0.3) | 0.0 (0.0) | 0.0 (0.4) | 0.0 (0.0) | 0.0 (0.0) | 0.0 (0.0) | 0.0 (0.0) | 0.0 (0.0) | 0.0 (0.0) |
| North Thompson_SU_1.3 | 0.0 (0.8) | 0.7 (0.5) | 0.0 (0.4) | 0.0 (0.0) | 0.0 (0.0) | 0.1 (0.1) | 0.1 (0.1) | 0.0 (0.0) | 0.5 (0.5) | 0.1 (0.1) |
| Shuswap River_SU_0.3 | 0.0 (0.1) | 0.8 (0.6) | 0.0 (0.0) | 0.0 (0.0) | 0.0 (0.0) | 0.4 (0.2) | 0.1 (0.1) | 1.7 (1.2) | 2.6 (0.7) | 0.9 (0.3) |
| South Thompson-Bessette Creek_SU_1.2 | 0.0 (7.9) | 0.0 (0.1) | 0.0 (0.2) | 0.0 (0.3) | 0.0 (0.0) | 0.0 (0.0) | 0.0 (0.0) | 0.0 (0.1) | 0.0 (0.0) | 0.0 (0.0) |
| South Thompson_SU_0.3 | 0.0 (0.2) | 64.9 (3.0) | 31.0 (8.5) | 0.0 (0.7) | 3.0 (1.0) | 4.9 (0.6) | 16.3 (1.2) | 0.0 (0.0) | 9.2 (1.4) | 16.8 (1.2) |
| South Thompson_SU_1.3 | 0.0 (1.8) | 0.0 (0.0) | 0.0 (0.9) | 0.0 (0.0) | 0.0 (0.0) | 0.0 (0.0) | 0.0 (0.0) | 0.8 (0.9) | 0.0 (0.0) | 0.0 (0.0) |
| Lower Thompson_SP_1.2 | 0.0 (0.2) | 0.0 (0.1) | 0.0 (0.9) | 0.0 (0.7) | 0.0 (0.0) | 0.0 (0.0) | 0.0 (0.0) | 0.0 (0.0) | 0.0 (0.0) | 0.0 (0.0) |
| Lower Fraser River_SP_1.3 | 0.0 (0.0) | 0.0 (0.0) | 0.0 (0.0) | 0.0 (0.0) | 0.0 (0.0) | 0.0 (0.0) | 0.0 (0.0) | 0.0 (0.0) | 0.0 (0.0) | 0.0 (0.0) |
| Lower Fraser River_SU_1.3 | 0.0 (0.0) | 0.0 (0.0) | 0.0 (0.2) | 0.0 (0.0) | 0.0 (0.0) | 0.0 (0.0) | 0.0 (0.0) | 0.0 (0.0) | 0.0 (0.0) | 0.0 (0.0) |
| Lower Fraser River-Upper Pitt_SU_1.3 | 0.0 (0.0) | 0.0 (0.0) | 0.0 (0.0) | 0.0 (4.9) | 0.5 (0.5) | 0.0 (0.0) | 0.0 (0.0) | 0.0 (0.0) | 0.0 (0.0) | 0.0 (0.0) |
| Maria Slough_SU_0.3 | 0.0 (0.0) | 0.0 (0.0) | 0.0 (0.0) | 0.0 (0.0) | 0.0 (0.0) | 0.1 (0.1) | 0.0 (0.0) | 0.0 (0.0) | 0.0 (0.0) | 0.0 (0.0) |
| Lower Fraser River_FA_0.3 | 0.0 (0.2) | 2.3 (1.0) | 17.2 (5.4) | 0.0 (0.1) | 0.0 (0.1) | 0.2 (0.1) | 1.3 (0.4) | 3.4 (1.6) | 2.9 (0.8) | 1.1 (0.4) |
| East Vancouver Island-North_FA_0.x | 0.0 (1.4) | 0.0 (0.0) | 0.0 (0.1) | 0.0 (1.0) | 0.0 (0.0) | 0.3 (0.2) | 0.1 (0.1) | 2.5 (1.4) | 0.0 (0.0) | 0.2 (0.2) |
| East Vancouver Island-Qualicum and Puntledge_FA_0.x | 0.0 (0.0) | 0.2 (0.3) | 6.2 (5.8) | 0.0 (0.4) | 1.5 (0.7) | 0.4 (0.2) | 1.0 (0.4) | 0.0 (0.0) | 2.0 (1.0) | 0.6 (0.4) |
| East Vancouver Island-Nanaimo and Chemainus_FA_0.x | 0.0 (2.3) | 0.0 (0.0) | 0.0 (0.0) | 0.0 (0.0) | 0.0 (0.0) | 0.0 (0.0) | 0.0 (0.0) | 0.1 (0.2) | 0.0 (0.0) | 0.0 (0.0) |
| East Vancouver Island-Nanaimo_SP_1.x | 0.0 (2.0) | 0.0 (0.0) | 0.0 (0.0) | 0.0 (0.1) | 0.0 (0.0) | 0.0 (0.0) | 0.0 (0.0) | 0.0 (0.0) | 0.0 (0.0) | 0.0 (0.0) |
| East Vancouver Island-Georgia Strait_SU_0.3 | 0.0 (0.8) | 0.0 (0.0) | 0.0 (0.0) | 0.0 (0.0) | 0.0 (0.1) | 0.0 (0.0) | 0.0 (0.0) | 0.2 (0.4) | 0.0 (0.0) | 0.0 (0.0) |
| East Vancouver Island-Cowichan and Koksilah_FA_0.x | 0.0 (0.3) | 0.1 (0.2) | 6.9 (4.2) | 0.0 (0.0) | 0.0 (0.0) | 0.5 (0.2) | 0.6 (0.3) | 1.4 (1.2) | 0.6 (0.5) | 0.8 (0.3) |
| West Vancouver Island-Nootka and Kyuquot_FA_0.x | 0.0 (4.4) | 0.0 (0.1) | 0.0 (1.0) | 0.0 (1.4) | 39.5 (3.3) | 54.4 (1.4) | 20.0 (1.4) | 5.9 (1.8) | 7.4 (1.3) | 3.4 (0.6) |
| West Vancouver Island-North_FA_0.x | 0.0 (0.2) | 0.0 (0.0) | 0.0 (0.0) | 0.0 (0.1) | 2.0 (1.0) | 2.2 (0.4) | 2.8 (0.2) | 0.3 (0.2) | 0.2 (0.2) | 0.0 (0.0) |
| West Vancouver Island-South_FA_0.x | 0.0 (1.0) | 7.6 (1.6) | 27.6 (7.4) | 0.0 (3.8) | 39.3 (3.2) | 26.2 (1.1) | 32.1 (1.7) | 23.6 (3.4) | 34.1 (2.3) | 67.3 (1.7) |
| Okanagan_1.x | 0.0 (0.1) | 0.0 (0.1) | 0.0 (0.0) | 0.0 (2.4) | 0.0 (0.0) | 0.0 (0.0) | 1.4 (0.6) | 0.0 (0.0) | 0.0 (0.0) | 0.0 (0.0) |
| Juan de Fuca | 0.0 (0.0) | 0.0 (0.0) | 0.0 (0.1) | 0.0 (0.0) | 0.5 (0.5) | 0.2 (0.1) | 0.0 (0.0) | 0.8 (1.0) | 1.4 (0.6) | 0.0 (0.0) |
| Coastal Washington | 0.0 (3.7) | 0.4 (0.3) | 3.4 (3.2) | 0.0 (0.2) | 0.0 (0.0) | 0.8 (0.2) | 3.3 (0.5) | 0.8 (0.9) | 0.3 (0.3) | 0.1 (0.1) |
| North Puget Sound | 11.3 (14.1) | 4.6 (1.2) | 0.0 (0.3) | 33.4 (15.6) | 6.1 (1.9) | 2.0 (0.2) | 1.4 (0.3) | 22.4 (3.9) | 8.7 (1.4) | 1.8 (0.5) |
| South Puget Sound | 88.6 (21.9) | 16.3 (2.3) | 7.6 (4.9) | 50.0 (16.8) | 3.4 (1.5) | 0.9 (0.3) | 0.3 (0.2) | 19.0 (3.7) | 12.8 (2.1) | 2.9 (0.6) |
| Lower Columbia River | 0.0 (1.1) | 0.4 (0.4) | 0.0 (0.1) | 16.6 (9.4) | 1.5 (0.8) | 0.9 (0.2) | 2.7 (0.6) | 8.4 (2.5) | 3.4 (0.8) | 1.2 (0.3) |
| Mid Columbia River_SP | 0.0 (3.1) | 0.0 (0.0) | 0.0 (0.7) | 0.1 (0.7) | 0.3 (0.5) | 0.1 (0.2) | 0.1 (0.1) | 3.0 (3.0) | 4.8 (1.4) | 0.0 (0.0) |
| Upper Columbia River_SP | 0.0 (2.2) | 0.0 (0.0) | 0.0 (0.2) | 0.0 (3.8) | 0.0 (0.0) | 0.0 (0.0) | 0.0 (0.0) | 0.0 (0.0) | 0.0 (0.1) | 0.0 (0.0) |
| Upper Columbia River_SU_FA | 0.0 (7.7) | 1.1 (0.7) | 0.0 (0.1) | 0.0 (1.8) | 1.0 (0.7) | 2.6 (0.4) | 7.5 (0.9) | 0.2 (0.8) | 2.6 (0.8) | 0.8 (0.3) |
| Snake River_FA | 0.0 (0.1) | 0.0 (0.0) | 0.0 (0.1) | 0.0 (0.0) | 0.0 (0.0) | 0.3 (0.2) | 2.2 (0.6) | 0.7 (0.8) | 2.2 (0.8) | 0.8 (0.3) |
| Snake River_SP_SU | 0.0 (0.4) | 0.0 (0.1) | 0.0 (0.2) | 0.0 (1.3) | 0.0 (0.0) | 0.0 (0.0) | 0.0 (0.0) | 0.0 (0.2) | 0.0 (0.0) | 0.0 (0.0) |
| North & Central Oregon | 0.0 (0.5) | 0.0 (0.0) | 0.0 (0.2) | 0.0 (0.4) | 0.0 (0.2) | 0.6 (0.3) | 2.6 (0.7) | 0.0 (0.0) | 0.0 (0.0) | 0.1 (0.1) |
| Upper Willamette River | 0.0 (0.3) | 0.0 (0.0) | 0.0 (0.0) | 0.0 (0.6) | 0.0 (0.0) | 0.1 (0.1) | 0.1 (0.2) | 0.8 (0.7) | 0.0 (0.2) | 0.0 (0.0) |
| South Oregon coastal | 0.0 (2.3) | 0.0 (0.0) | 0.0 (0.4) | 0.0 (1.2) | 0.0 (0.0) | 0.9 (0.2) | 3.1 (0.6) | 0.0 (0.0) | 0.9 (0.7) | 0.3 (0.2) |
| California Klamath Trinity | 0.0 (0.0) | 0.0 (0.0) | 0.0 (0.1) | 0.0 (1.5) | 0.0 (0.1) | 0.0 (0.0) | 0.1 (0.1) | 0.0 (0.1) | 0.0 (0.0) | 0.1 (0.1) |
| California Central Valley_Fall | 0.0 (3.5) | 0.0 (0.0) | 0.0 (0.1) | 0.0 (1.3) | 0.5 (0.6) | 0.2 (0.1) | 0.3 (0.2) | 1.7 (0.9) | 1.3 (0.6) | 0.2 (0.1) |
| California Central Valley_Spring | 0.0 (0.1) | 0.0 (0.0) | 0.0 (0.0) | 0.0 (0.0) | 0.0 (0.1) | 0.0 (0.0) | 0.0 (0.0) | 0.0 (0.0) | 1.3 (0.7) | 0.0 (0.0) |
| Coastal California | 0.0 (7.4) | 0.0 (0.1) | 0.0 (0.0) | 0.0 (0.0) | 0.0 (0.0) | 0.0 (0.0) | 0.0 (0.0) | 0.0 (0.0) | 0.0 (0.0) | 0.0 (0.0) |

Supplementary Table S5 concluded

| Conservation Unit | WCVI south sport | Area 23 commercial | Taaq-wiihak troll | | | | WCVI troll | | | Area 25 Net |
| --- | --- | --- | --- | --- | --- | --- | --- | --- | --- | --- |
|  |  |  | NWVI | | SWVI | | NWVI | SWVI | | NWVI |
|  | Sept | August | July | August | July | August | Sept | August | Sept | August |
| Sample size | 65 | 122 | 20 | 39 | 72 | 7 | 15 | 48 | 75 | 197 |
| N PBT | 29 | 101 | 1 | 1 | 13 | 0 | 0 | 1 | 3 | 3 |
| Southeast Alaska | 0.0 (0.5) | 0.0 (0.1) | 0.0 (0.1) | 0.0 (0.6) | 0.0 (0.2) | 0.0 (0.3) | 0.0 (0.0) | 0.0 (0.1) | 0.0 (0.0) | 0.0 (0.0) |
| Alsek | 0.0 (0.1) | 0.0 (0.0) | 0.0 (0.1) | 0.0 (0.1) | 0.0 (0.1) | 0.0 (1.1) | 0.0 (0.2) | 0.0 (0.1) | 0.0 (0.1) | 0.0 (0.0) |
| Unuk | 0.0 (0.0) | 0.0 (0.1) | 0.0 (0.0) | 0.0 (0.0) | 0.0 (0.0) | 0.0 (0.0) | 0.0 (0.0) | 0.0 (0.0) | 0.0 (0.0) | 0.0 (0.0) |
| Taku_early timing | 0.0 (0.0) | 0.0 (0.1) | 0.0 (0.2) | 0.0 (0.3) | 0.0 (0.1) | 0.0 (2.5) | 0.0 (0.4) | 0.0 (0.4) | 0.0 (0.2) | 0.0 (0.0) |
| Taku_mid timing | 0.0 (0.0) | 0.0 (0.0) | 0.0 (0.1) | 0.0 (0.0) | 0.0 (0.0) | 0.0 (0.9) | 0.0 (0.0) | 0.0 (0.4) | 0.0 (0.0) | 0.0 (0.0) |
| Taku_late timing | 0.0 (0.0) | 0.0 (0.0) | 0.0 (1.4) | 0.0 (0.1) | 0.0 (0.0) | 0.0 (0.1) | 0.0 (0.0) | 0.0 (0.1) | 0.0 (0.0) | 0.0 (0.0) |
| Stikine_early timing | 0.0 (0.0) | 0.0 (0.0) | 0.0 (0.3) | 0.0 (0.1) | 0.0 (0.1) | 0.0 (0.4) | 0.0 (0.2) | 0.0 (0.2) | 0.0 (0.1) | 0.0 (0.0) |
| Stikine_late timing | 0.0 (0.0) | 0.0 (0.0) | 0.0 (0.1) | 0.0 (0.0) | 0.0 (0.0) | 0.0 (0.5) | 0.0 (0.2) | 0.0 (0.0) | 0.0 (0.0) | 0.0 (0.0) |
| Haida Gwaii-North | 0.0 (0.0) | 0.0 (0.0) | 0.0 (0.1) | 0.0 (0.4) | 0.0 (0.0) | 0.0 (0.0) | 0.0 (0.0) | 0.0 (0.0) | 0.0 (0.0) | 0.0 (0.0) |
| Upper Nass | 0.0 (0.3) | 0.0 (0.0) | 0.0 (0.6) | 0.0 (0.3) | 0.0 (0.3) | 0.0 (1.8) | 0.0 (2.5) | 0.0 (0.2) | 0.0 (0.0) | 0.0 (0.2) |
| Portland Sound-Observatory Inlet-Lower Nass | 0.0 (0.1) | 0.0 (0.0) | 0.0 (1.2) | 0.0 (0.0) | 0.0 (0.0) | 0.0 (0.6) | 0.0 (0.1) | 0.0 (0.3) | 0.0 (0.0) | 0.0 (0.0) |
| Ecstall | 0.0 (0.0) | 0.0 (0.0) | 0.0 (0.0) | 0.0 (0.0) | 0.0 (0.1) | 0.0 (0.0) | 0.0 (0.1) | 0.0 (0.0) | 0.0 (0.1) | 0.0 (0.0) |
| Skeena Estuary | 0.0 (0.0) | 0.0 (0.0) | 0.0 (0.0) | 0.0 (0.0) | 0.0 (0.1) | 0.0 (0.0) | 0.0 (0.0) | 0.0 (0.0) | 0.0 (0.0) | 0.0 (0.0) |
| Lower Skeena | 0.0 (0.1) | 0.0 (0.0) | 0.0 (1.4) | 0.0 (0.1) | 0.0 (0.2) | 0.0 (1.2) | 0.0 (0.2) | 0.0 (0.1) | 0.0 (0.0) | 0.0 (0.1) |
| Kalum_early timing | 0.0 (0.0) | 0.0 (0.0) | 0.0 (0.0) | 0.0 (0.0) | 0.0 (0.0) | 0.0 (0.0) | 0.0 (0.0) | 0.0 (0.0) | 0.0 (0.0) | 0.0 (0.0) |
| Kalum_late timing | 0.0 (0.0) | 0.0 (0.0) | 0.0 (0.1) | 0.0 (0.0) | 0.0 (0.0) | 0.0 (0.3) | 0.0 (0.1) | 0.0 (0.0) | 0.0 (0.0) | 0.0 (0.1) |
| Zymoetz | 0.0 (0.0) | 0.0 (0.0) | 0.0 (0.0) | 0.0 (0.0) | 0.0 (0.0) | 0.0 (2.5) | 0.0 (0.9) | 0.0 (0.0) | 0.0 (0.0) | 0.0 (0.0) |
| Sicintine | 0.0 (0.0) | 0.0 (0.0) | 0.0 (0.0) | 0.0 (0.0) | 0.0 (0.0) | 0.0 (0.0) | 0.0 (0.0) | 0.0 (0.0) | 0.0 (0.0) | 0.0 (0.0) |
| Middle Skeena-mainstem tributaries | 0.0 (0.1) | 0.0 (0.2) | 0.0 (0.7) | 0.0 (0.3) | 0.0 (0.2) | 0.0 (0.9) | 0.0 (0.6) | 0.0 (0.2) | 0.0 (0.0) | 0.0 (0.0) |
| Middle Skeena-large lakes | 0.0 (0.0) | 0.0 (0.0) | 0.0 (0.0) | 0.0 (0.6) | 0.0 (0.0) | 0.0 (0.3) | 0.0 (0.0) | 0.0 (0.1) | 0.0 (0.0) | 0.0 (0.0) |
| Upper Skeena | 0.0 (0.2) | 0.0 (0.1) | 0.0 (0.0) | 0.0 (0.2) | 0.0 (0.1) | 0.0 (0.1) | 0.0 (0.3) | 0.0 (0.5) | 0.0 (0.1) | 0.0 (0.0) |
| Upper Bulkley River | 0.0 (0.2) | 0.0 (0.0) | 0.0 (0.0) | 0.0 (0.0) | 0.0 (0.0) | 0.0 (0.0) | 0.0 (0.0) | 0.0 (0.0) | 0.0 (0.1) | 0.0 (0.0) |
| North and Central Coast-late timing | 0.0 (0.4) | 0.0 (0.0) | 0.0 (0.1) | 0.0 (0.0) | 0.0 (0.0) | 0.0 (0.0) | 0.0 (0.0) | 0.0 (0.0) | 0.0 (0.0) | 0.0 (0.0) |
| North and Central Coast-early timing | 0.0 (0.0) | 0.0 (0.0) | 0.0 (0.2) | 0.0 (0.3) | 0.0 (0.0) | 0.0 (1.8) | 0.0 (0.0) | 0.0 (0.1) | 0.0 (0.0) | 0.0 (0.0) |
| Rivers Inlet | 0.0 (0.0) | 0.0 (0.0) | 0.0 (0.2) | 0.0 (0.0) | 0.0 (0.0) | 0.0 (1.3) | 0.0 (0.4) | 0.0 (0.0) | 0.0 (0.0) | 0.0 (0.0) |
| Wannock | 0.0 (0.0) | 0.0 (0.0) | 0.0 (0.2) | 0.0 (0.0) | 0.0 (0.0) | 0.0 (0.1) | 0.0 (0.0) | 0.0 (0.0) | 0.0 (0.0) | 0.0 (0.0) |
| Bella Coola-Bentinck | 0.0 (0.4) | 0.0 (0.1) | 0.0 (0.1) | 0.0 (0.2) | 0.0 (0.1) | 0.0 (1.9) | 0.0 (0.0) | 0.0 (0.2) | 0.0 (0.0) | 0.0 (0.0) |
| Dean River | 0.0 (0.0) | 0.0 (0.0) | 0.0 (0.0) | 0.0 (0.0) | 0.0 (0.0) | 0.0 (3.1) | 0.0 (0.0) | 0.0 (0.0) | 0.0 (0.0) | 0.0 (0.0) |
| Docee | 0.0 (0.0) | 0.0 (0.0) | 0.0 (0.0) | 0.0 (0.0) | 0.0 (0.0) | 0.0 (0.0) | 0.0 (0.0) | 0.0 (0.0) | 0.0 (0.0) | 0.0 (0.0) |
| Klinaklini_SU_1.3 | 0.0 (0.0) | 0.0 (0.0) | 0.0 (0.2) | 0.0 (0.0) | 0.0 (0.0) | 0.0 (1.6) | 0.0 (0.0) | 0.0 (0.1) | 0.0 (0.0) | 0.0 (0.0) |
| Southern Mainland-Southern Fjords_FA_0.x | 0.0 (0.0) | 0.0 (0.0) | 0.0 (0.2) | 0.0 (0.0) | 0.0 (0.0) | 0.0 (0.0) | 0.0 (0.0) | 0.0 (0.0) | 0.0 (0.0) | 0.0 (0.0) |
| Southern Mainland-Georgia Strait_FA_0.x | 0.0 (0.2) | 0.0 (0.1) | 0.0 (0.3) | 0.0 (0.1) | 0.0 (0.5) | 0.0 (0.8) | 0.0 (0.3) | 0.0 (0.2) | 0.0 (0.3) | 0.0 (0.1) |
| Upper Fraser River_SP_1.3 | 0.0 (0.3) | 0.0 (0.0) | 0.0 (1.2) | 0.0 (0.2) | 0.0 (0.4) | 0.0 (0.6) | 0.0 (1.0) | 0.0 (0.1) | 0.0 (0.2) | 0.0 (0.0) |
| Middle Fraser River_SU_1.3 | 0.0 (0.2) | 0.0 (0.1) | 0.0 (0.1) | 0.0 (0.2) | 1.4 (1.2) | 0.0 (1.0) | 0.0 (0.7) | 0.0 (0.0) | 0.0 (0.1) | 0.0 (0.0) |
| Middle Fraser River_SP_1.3 | 0.0 (0.3) | 0.0 (0.1) | 0.0 (0.4) | 0.0 (0.5) | 0.0 (0.2) | 0.0 (4.6) | 0.0 (0.4) | 0.0 (0.1) | 0.0 (0.2) | 0.0 (0.0) |
| Middle Fraser River-Portage_FA_1.3 | 0.0 (0.1) | 0.0 (0.0) | 0.0 (0.0) | 0.0 (0.0) | 0.0 (0.2) | 0.0 (0.0) | 0.0 (0.0) | 0.0 (0.0) | 0.0 (0.0) | 0.0 (0.0) |
| Middle Fraser-Fraser Canyon_SP_1.3 | 0.0 (0.0) | 0.0 (0.0) | 0.0 (0.0) | 0.0 (0.0) | 0.0 (0.0) | 0.0 (2.5) | 0.0 (0.0) | 0.0 (0.0) | 0.0 (0.0) | 0.0 (0.0) |
| North Thompson_SP_1.3 | 0.0 (0.0) | 0.0 (0.0) | 0.0 (0.0) | 0.0 (0.3) | 0.0 (0.4) | 0.0 (1.1) | 0.0 (0.0) | 0.0 (0.0) | 0.0 (0.0) | 0.0 (0.0) |
| North Thompson_SU_1.3 | 0.0 (0.2) | 0.0 (0.0) | 0.0 (0.3) | 0.0 (0.1) | 1.4 (1.1) | 0.0 (0.5) | 0.0 (0.2) | 0.0 (0.0) | 0.0 (0.1) | 0.0 (0.0) |
| Shuswap River_SU_0.3 | 0.0 (0.0) | 0.0 (0.1) | 0.0 (0.3) | 0.0 (0.0) | 1.0 (0.9) | 0.7 (3.5) | 0.0 (0.0) | 0.0 (0.0) | 0.0 (0.0) | 0.0 (0.0) |
| South Thompson-Bessette Creek_SU_1.2 | 0.0 (0.0) | 0.0 (0.0) | 0.0 (0.0) | 0.0 (0.5) | 0.0 (0.0) | 0.0 (0.4) | 0.0 (0.0) | 0.0 (0.0) | 0.0 (0.0) | 0.0 (0.0) |
| South Thompson_SU_0.3 | 0.0 (0.1) | 0.0 (0.1) | 30.0 (9.8) | 30.8 (5.7) | 14.3 (3.2) | 13.6 (11.9) | 6.7 (5.1) | 33.3 (7.0) | 1.3 (1.2) | 0.0 (0.0) |
| South Thompson_SU_1.3 | 0.0 (0.0) | 0.0 (0.1) | 0.0 (0.2) | 0.0 (0.2) | 0.0 (0.0) | 0.0 (0.5) | 0.0 (1.1) | 0.0 (0.0) | 0.0 (0.0) | 0.0 (0.0) |
| Lower Thompson_SP_1.2 | 0.0 (0.1) | 0.0 (0.0) | 0.0 (0.7) | 0.0 (0.6) | 0.0 (0.2) | 0.0 (2.5) | 0.0 (2.1) | 0.0 (0.3) | 0.0 (0.0) | 0.0 (0.0) |
| Lower Fraser River_SP_1.3 | 0.0 (0.0) | 0.0 (0.0) | 0.0 (0.0) | 0.0 (0.1) | 0.0 (0.0) | 0.0 (0.1) | 0.0 (0.1) | 0.0 (0.0) | 0.0 (0.0) | 0.0 (0.0) |
| Lower Fraser River_SU_1.3 | 0.0 (0.1) | 0.0 (0.0) | 0.0 (0.0) | 0.0 (0.1) | 0.0 (0.1) | 0.0 (1.1) | 0.0 (0.0) | 0.0 (0.3) | 0.0 (0.0) | 0.0 (0.0) |
| Lower Fraser River-Upper Pitt_SU_1.3 | 0.0 (0.3) | 0.0 (0.1) | 0.0 (0.1) | 0.0 (0.2) | 0.0 (0.1) | 0.0 (0.0) | 0.0 (0.3) | 0.0 (0.1) | 0.0 (0.0) | 0.0 (0.1) |
| Maria Slough_SU_0.3 | 0.0 (0.0) | 0.0 (0.0) | 0.0 (0.0) | 0.0 (0.0) | 0.0 (0.0) | 0.0 (0.1) | 0.0 (0.8) | 0.0 (0.0) | 0.0 (0.0) | 0.0 (0.0) |
| Lower Fraser River_FA_0.3 | 1.5 (1.4) | 0.0 (0.0) | 0.0 (0.6) | 2.6 (2.1) | 1.4 (1.2) | 0.0 (0.5) | 0.0 (1.1) | 6.3 (3.4) | 12.0 (3.8) | 0.0 (0.1) |
| East Vancouver Island-North_FA_0.x | 1.5 (1.5) | 0.0 (0.0) | 0.0 (0.9) | 0.0 (0.1) | 0.0 (0.0) | 0.0 (0.3) | 0.0 (0.5) | 0.0 (0.0) | 0.0 (0.4) | 0.0 (0.1) |
| East Vancouver Island-Qualicum and Puntledge_FA_0.x | 0.0 (0.2) | 0.0 (0.1) | 0.0 (0.1) | 0.0 (0.1) | 1.3 (1.6) | 0.0 (0.1) | 0.4 (0.4) | 0.0 (0.1) | 0.0 (0.1) | 0.0 (0.0) |
| East Vancouver Island-Nanaimo and Chemainus_FA_0.x | 0.0 (0.0) | 0.0 (0.0) | 0.0 (0.2) | 0.0 (0.0) | 0.0 (0.0) | 0.0 (3.4) | 0.3 (0.0) | 0.0 (0.1) | 0.0 (0.0) | 0.0 (0.0) |
| East Vancouver Island-Nanaimo_SP_1.x | 0.0 (0.0) | 0.0 (0.0) | 0.0 (0.0) | 0.0 (0.0) | 0.0 (0.0) | 0.0 (0.0) | 0.0 (0.0) | 0.0 (0.3) | 0.0 (0.0) | 0.0 (0.0) |
| East Vancouver Island-Georgia Strait_SU_0.3 | 0.0 (0.0) | 0.0 (0.0) | 0.0 (0.5) | 0.0 (0.0) | 0.0 (0.0) | 0.0 (0.0) | 0.0 (0.1) | 0.0 (0.0) | 0.0 (0.0) | 0.0 (0.0) |
| East Vancouver Island-Cowichan and Koksilah_FA_0.x | 3.1 (2.3) | 0.0 (0.0) | 0.0 (0.3) | 5.1 (3.4) | 0.0 (0.1) | 0.0 (0.0) | 5.9 (6.3) | 0.0 (0.1) | 4.0 (2.1) | 0.0 (0.0) |
| West Vancouver Island-Nootka and Kyuquot_FA_0.x | 0.0 (0.0) | 0.0 (0.1) | 0.0 (0.7) | 0.0 (0.2) | 2.9 (1.6) | 0.0 (1.4) | 0.0 (0.4) | 0.0 (0.5) | 0.0 (0.0) | 99.8 (0.6) |
| West Vancouver Island-North_FA_0.x | 0.0 (0.2) | 0.0 (0.0) | 0.0 (0.8) | 0.0 (0.0) | 0.0 (0.0) | 0.0 (0.1) | 0.0 (0.0) | 0.0 (0.1) | 0.0 (0.1) | 0.0 (0.0) |
| West Vancouver Island-South_FA_0.x | 92.3 (3.4) | 100.0 (0.8) | 30.0 (9.2) | 2.6 (2.3) | 26.2 (5.5) | 0.0 (1.2) | 0.0 (0.7) | 0.0 (0.3) | 2.7 (1.8) | 0.2 (0.5) |
| Okanagan_1.x | 0.0 (0.0) | 0.0 (0.0) | 0.4 (0.7) | 0.0 (0.5) | 0.0 (0.0) | 0.0 (0.0) | 0.0 (0.0) | 0.0 (0.0) | 0.0 (0.0) | 0.0 (0.0) |
| Juan de Fuca | 0.0 (0.0) | 0.0 (0.0) | 0.0 (0.0) | 0.0 (0.0) | 0.0 (0.1) | 0.0 (0.0) | 0.0 (0.0) | 0.0 (0.1) | 0.0 (0.0) | 0.0 (0.0) |
| Coastal Washington | 0.0 (0.1) | 0.0 (0.0) | 5.5 (5.1) | 5.1 (3.1) | 1.4 (1.3) | 0.0 (0.8) | 6.7 (6.0) | 2.1 (1.9) | 4.0 (3.0) | 0.0 (0.0) |
| North Puget Sound | 1.5 (1.1) | 0.0 (0.4) | 0.0 (0.1) | 0.0 (0.1) | 1.6 (1.5) | 0.0 (0.3) | 0.0 (0.2) | 0.0 (0.1) | 11.2 (2.6) | 0.0 (0.0) |
| South Puget Sound | 0.0 (0.0) | 0.0 (0.0) | 5.0 (4.7) | 0.0 (0.1) | 27.6 (5.7) | 0.0 (0.0) | 0.0 (1.5) | 18.7 (5.8) | 16.7 (4.0) | 0.0 (0.0) |
| Lower Columbia River | 0.0 (0.0) | 0.0 (0.1) | 0.0 (0.9) | 10.3 (4.0) | 12.5 (4.4) | 0.0 (5.1) | 6.1 (7.2) | 14.2 (5.4) | 8.9 (3.6) | 0.0 (0.0) |
| Mid Columbia River_SP | 0.0 (0.0) | 0.0 (0.2) | 0.0 (0.2) | 0.0 (0.7) | 0.0 (0.0) | 0.0 (1.4) | 0.5 (0.7) | 0.4 (2.3) | 2.8 (1.4) | 0.0 (0.1) |
| Upper Columbia River_SP | 0.0 (0.0) | 0.0 (0.0) | 0.0 (0.1) | 0.0 (0.0) | 0.0 (0.1) | 0.0 (0.1) | 0.0 (0.1) | 0.0 (0.1) | 0.0 (0.1) | 0.0 (0.0) |
| Upper Columbia River_SU_FA | 0.0 (0.1) | 0.0 (0.0) | 4.9 (5.7) | 7.9 (4.2) | 0.0 (0.0) | 25.5 (14.2) | 33.4 (10.7) | 8.9 (4.3) | 16.5 (5.3) | 0.0 (0.0) |
| Snake River_FA | 0.0 (0.0) | 0.0 (0.0) | 0.0 (0.0) | 15.2 (5.3) | 0.0 (0.0) | 3.0 (4.9) | 0.0 (0.1) | 5.7 (3.5) | 2.2 (2.2) | 0.0 (0.0) |
| Snake River_SP_SU | 0.0 (0.1) | 0.0 (0.1) | 0.0 (1.4) | 0.0 (0.5) | 0.0 (0.7) | 0.0 (1.8) | 0.0 (0.6) | 0.0 (0.2) | 0.0 (0.1) | 0.0 (0.1) |
| North & Central Oregon | 0.0 (0.0) | 0.0 (0.0) | 8.3 (4.5) | 8.5 (4.4) | 0.0 (0.1) | 45.6 (20.7) | 16.3 (9.3) | 4.2 (2.4) | 3.6 (2.3) | 0.0 (0.0) |
| Upper Willamette River | 0.0 (0.2) | 0.0 (0.0) | 0.0 (1.7) | 0.0 (0.0) | 0.0 (0.0) | 0.0 (0.0) | 6.6 (6.6) | 0.0 (0.1) | 0.3 (0.9) | 0.0 (0.0) |
| South Oregon coastal | 0.0 (0.2) | 0.0 (0.0) | 10.8 (8.9) | 9.4 (4.5) | 1.4 (0.2) | 11.5 (16.8) | 17.0 (8.2) | 0.0 (0.8) | 3.1 (1.9) | 0.0 (0.2) |
| California Klamath Trinity | 0.0 (0.0) | 0.0 (0.0) | 0.0 (0.10 | 0.1 (0.0) | 0.0 (0.0) | 0.0 (0.4) | 0.0 (0.0) | 0.0 (0.0) | 0.0 (0.0) | 0.0 (0.0) |
| California Central Valley_Fall | 0.0 (0.0) | 0.0 (0.1) | 1.0 (3.5) | 2.6 (2.7) | 1.9 (1.7) | 0.0 (0.9) | 0.0 (0.6) | 1.9 (2.7) | 8.0 (3.2) | 0.0 (0.0) |
| California Central Valley_Spring | 0.0 (0.3) | 0.0 (0.0) | 4.0 (4.6) | 0.0 (0.1) | 3.6 (2.7) | 0.0 (0.0) | 0.0 (0.3) | 4.4 (3.9) | 2.7 (1.3) | 0.0 (0.0) |
| Coastal California | 0.0 (0.0) | 0.0 (0.0) | 0.0 (0.0) | 0.0 (0.0) | 0.0 (0.0) | 0.0 (0.0) | 0.0 (0.2) | 0.0 (0.0) | 0.0 (0.0) | 0.0 (0.0) |

Supplementary Table S6. Number of individuals genotyped (N) and those subsequently identified via parentage-based tagging (N-PBT) by age (years) for Chinook salmon populations sampled in 2018 fisheries in British Columbia. Fisheries were northern troll, northern sport, Skeena River test, Johnstone Strait sport, Strait of Georgia (SoG) north and south sport, Capilano River derby, Juan de Fuca (JDF) sport, west coast Vancouver Island (WCVI) north and south sport, and west coast Vancouver Island T’aaq-wiihak north (NWVI-T’aaq) and south (SWVI-T’aaq) troll.

| CU | Population | Age | Northern troll | Northern sport | Skeena River test | Johnstone Strait sport | SoG north sport | | | SoG south sport |
| --- | --- | --- | --- | --- | --- | --- | --- | --- | --- | --- |
|  |  |  |  |  |  |  | Legal | Sublegal | Unknown | Legal |
|  | N genotyped |  | 1,222 | 419 | 58 | 499 | 688 | 74 | 112 | 674 |
|  | N-PBT |  | 84 | 77 | 3 | 141 | 147 | 17 | 28 | 85 |
| Kalum-late | Kitsumkalum River-lower | 4 | 1 |  |  |  |  |  |  |  |
|  |  | 5 |  | 1 | 3 |  |  |  |  |  |
| BCR-Bent | Atnarko River | 3 | 1 |  |  |  |  |  |  |  |
| SMn-SFj | Phillips River | 3 |  |  |  | 3 |  |  |  |  |
| SMn-GStr | Tenderfoot Creek | 2 |  |  |  |  | 1 |  |  |  |
| STh-SHUR | Shuswap River-lower | 2 |  |  |  |  |  | 1 |  |  |
|  |  | 3 |  | 1 |  |  |  |  |  |  |
|  |  | 4 | 3 |  |  | 1 | 1 |  |  | 1 |
|  | Shuswap River-mid | 4 | 1 |  |  | 3 | 1 |  |  |  |
| LFR-fall | Harrison River | 2 |  |  |  |  |  |  |  |  |
|  |  | 3 |  |  |  |  | 2 |  | 1 | 3 |
|  |  | 4 |  |  |  |  |  |  |  | 1 |
|  | Chilliwack | 2 |  |  |  |  |  | 2 |  | 1 |
|  |  | 3 | 1 | 1 |  | 2 | 48 |  | 4 | 33 |
|  |  | 4 |  | 1 |  |  | 9 |  |  | 9 |
|  | Capilano | 2 |  |  |  |  |  | 1 |  | 1 |
|  |  | 3 |  |  |  | 1 | 20 |  | 1 | 22 |
| NEVI | Quinsam River | 3 | 2 | 5 |  | 16 | 3 |  | 1 |  |
|  |  | 4 | 2 | 3 |  | 10 | 5 |  |  |  |
|  |  | 5 |  | 5 |  |  |  |  |  |  |
| QP-fall | Puntledge summer | 3 |  |  |  |  | 5 |  | 1 |  |
|  |  | 4 |  |  |  | 1 |  |  |  |  |
|  | Puntledge fall | 2 |  |  |  |  |  | 6 | 1 |  |
|  |  | 3 | 3 |  |  | 7 | 16 | 1 | 7 | 3 |
|  |  | 4 |  | 1 |  | 2 | 7 |  |  | 1 |
|  | Qualicum River | 2 |  |  |  |  |  |  |  |  |
|  |  | 3 |  | 1 |  | 3 | 10 | 4 | 5 | 3 |
|  |  | 4 |  | 1 |  | 4 | 5 |  | 2 | 2 |
| CWCH-KOK | Cowichan River | 2 |  |  |  |  |  |  |  |  |
|  |  | 3 |  |  |  |  | 8 | 2 | 5 | 5 |
|  |  | 4 |  |  |  |  | 5 |  |  |  |
| NoKy | Burman River | 4 |  |  |  | 1 |  |  |  |  |
|  | Conuma | 4 |  |  |  | 7 |  |  |  |  |
|  | Gold River | 3 |  |  |  | 1 |  |  |  |  |
|  |  | 4 | 1 |  |  |  |  |  |  |  |
|  | Leiner River | 4 |  |  |  |  |  |  |  |  |
|  | Tlupana River | 4 |  |  |  |  |  |  |  |  |
| SWVI | Bedwell River | 3 |  |  |  |  |  |  |  |  |
|  | Nahmint | 3 | 1 |  |  | 1 |  |  |  |  |
|  |  | 5 | 1 |  |  |  |  |  |  |  |
|  | Nitinat River | 3 |  | 1 |  |  |  |  |  |  |
|  |  | 4 | 2 | 1 |  |  |  |  |  |  |
|  | Robertson Creek | 2 |  |  |  |  |  |  |  |  |
|  |  | 3 | 40 | 15 |  | 45 |  |  |  |  |
|  |  | 4 | 20 | 30 |  | 27 |  |  |  |  |
|  |  | 5 | 3 | 8 |  | 3 | 1 |  |  |  |
|  | Sarita River | 3 | 1 | 1 |  | 2 |  |  |  |  |
|  |  | 4 | 1 | 1 |  |  |  |  |  |  |
|  | Thornton Creek | 2 |  |  |  |  |  |  |  |  |
|  |  | 3 |  |  |  | 1 |  |  |  |  |

Supplementary Table S6 concluded

| CU | Population | Age | SoG south sport | Capilano derby | JDF sport | WCVI north sport | WCVI south sport | NWVI-T’aaq | SWVI-T’aaq | Total |
| --- | --- | --- | --- | --- | --- | --- | --- | --- | --- | --- |
|  |  |  | Sublegal |  |  |  |  |  |  |  |
|  | N genotyped |  | 471 | 49 | 611 | 786 | 572 | 23 | 28 | 6,286 |
|  | N PBT |  | 56 | 34 | 41 | 82 | 83 | 0 | 11 | 889 |
| Kalum-late | Kitsumkalum River-lower | 4 |  |  |  |  |  |  |  | 1 |
|  |  | 5 |  |  |  |  |  |  |  | 4 |
| BCR-Bent | Atnarko River | 3 |  |  |  |  |  |  |  | 1 |
| SMn-SFj | Phillips River | 3 |  |  |  |  |  |  |  | 3 |
| SMn-GStr | Tenderfoot Creek | 2 |  |  |  |  |  |  |  | 1 |
| STh-SHUR | Shuswap River-lower | 2 |  |  |  |  |  |  |  | 1 |
|  |  | 3 |  |  | 1 |  |  |  |  | 2 |
|  |  | 4 | 1 |  | 7 |  | 1 |  |  | 15 |
|  | Shuswap River-mid | 4 |  |  |  |  |  |  |  | 5 |
| LFR-fall | Harrison River | 2 | 1 |  |  |  |  |  |  | 1 |
|  |  | 3 | 1 |  |  |  |  |  |  | 7 |
|  |  | 4 |  |  |  | 1 |  |  |  | 2 |
|  | Chilliwack | 2 | 19 |  | 1 |  |  |  |  | 23 |
|  |  | 3 | 7 | 1 | 6 |  | 2 |  |  | 105 |
|  |  | 4 |  | 1 |  | 1 |  |  |  | 21 |
|  | Capilano | 2 | 5 |  | 2 |  |  |  |  | 9 |
|  |  | 3 | 4 | 32 | 3 |  |  |  |  | 83 |
| NEVI | Quinsam River | 3 |  |  |  |  |  |  |  | 27 |
|  |  | 4 |  |  |  |  |  |  |  | 20 |
|  |  | 5 |  |  |  |  |  |  |  | 5 |
| QP-fall | Puntledge summer | 3 |  |  |  |  |  |  |  | 6 |
|  |  | 4 |  |  |  |  |  |  |  | 1 |
|  | Puntledge fall | 2 | 3 |  |  |  |  |  |  | 10 |
|  |  | 3 |  |  |  | 3 |  |  |  | 40 |
|  |  | 4 |  |  |  | 2 |  |  |  | 13 |
|  | Qualicum River | 2 | 4 |  |  |  |  |  |  | 4 |
|  |  | 3 | 3 |  |  | 1 |  |  |  | 30 |
|  |  | 4 |  |  | 1 | 1 | 2 |  |  | 18 |
| CWCH-KOK | Cowichan River | 2 | 4 |  | 1 |  |  |  |  | 5 |
|  |  | 3 | 3 |  |  | 1 | 1 |  |  | 25 |
|  |  | 4 |  |  | 2 |  | 1 |  |  | 8 |
| NoKy | Burman River | 4 |  |  |  |  |  |  |  | 1 |
|  | Conuma | 4 |  |  |  | 5 |  |  |  | 12 |
|  | Gold River | 3 |  |  |  |  |  |  |  | 1 |
|  |  | 4 |  |  |  | 1 |  |  |  | 2 |
|  | Leiner River | 4 |  |  |  | 1 |  |  |  | 1 |
|  | Tlupana River | 4 |  |  |  | 1 |  |  |  | 1 |
| SWVI | Bedwell River | 3 |  |  |  | 2 |  |  |  | 2 |
|  | Nahmint | 3 |  |  |  |  |  |  |  | 2 |
|  |  | 5 |  |  |  |  |  |  |  | 1 |
|  | Nitinat River | 3 |  |  |  |  | 1 |  |  | 2 |
|  |  | 4 |  |  |  |  | 2 |  |  | 5 |
|  | Robertson Creek | 2 |  |  | 1 |  |  |  |  | 1 |
|  |  | 3 | 1 |  | 7 | 33 | 41 |  | 7 | 189 |
|  |  | 4 |  |  | 7 | 22 | 22 |  | 4 | 132 |
|  |  | 5 |  |  | 2 | 2 | 2 |  |  | 21 |
|  | Sarita River | 3 |  |  |  | 1 | 3 |  |  | 8 |
|  |  | 4 |  |  |  |  |  |  |  | 2 |
|  | Thornton Creek | 2 |  |  |  |  | 1 |  |  | 1 |
|  |  | 3 |  |  |  | 4 | 4 |  |  | 9 |

Supplementary Table S7. Number of individuals genotyped (N) and those subsequently identified via parentage-based tagging (N-PBT) by age (years) for Chinook salmon populations sampled in 2019 fisheries in British Columbia. Fisheries were northern troll, northern sport, Skeena River test, Bella Coola First Nation, central Gillnet, central sport, Johnstone Strait (JS) sport, Strait of Georgia (SoG) north and south sport, Juan de Fuca (JDF) sport, west coast Vancouver Island (WCVI) north and south sport, west coast Vanouver Island troll, west coast Vancouver Island T’aaq-wiihak north (NWVI-T’aaq) and south (SWVI-T’aaq) troll, Area 25 net, Mququin test fishery, and Area 12 Chinook bycatch in sockeye salmon test fishery.

|  |  | Fishery | | | | | | | | | | | |
| --- | --- | --- | --- | --- | --- | --- | --- | --- | --- | --- | --- | --- | --- |
| Population | Age | Northern troll | Northern sport | Bella Coola FN | Central gillnet | Central sport | JS sport | Strait of Georgia north sport | | | Strait of Georgia south sport | | |
|  |  |  |  |  |  |  |  | Legal | Sublegal | Unknown | Legal | Sublegal | Unknown |
| N |  | 661 | 1,018 | 116 | 180 | 262 | 1,085 | 1,736 | 213 | 146 | 820 | 416 | 39 |
| N PBT |  | 33 | 185 | 40 | 46 | 92 | 305 | 470 | 83 | 32 | 180 | 61 | 7 |
| Yakoun River | 3 | 1 | 1 |  |  |  |  |  |  |  |  |  |  |
| Kitsumkalum | 3 |  |  |  |  |  |  |  |  | 1 |  |  |  |
|  | 4 |  | 3 |  |  |  |  |  |  |  |  |  |  |
|  | 5 |  | 4 |  |  |  |  |  |  |  |  |  |  |
|  | 6 |  | 1 |  |  |  |  |  |  |  |  |  |  |
| Kitimat River | 3 |  | 4 | 2 | 1 | 2 |  |  |  |  |  |  |  |
| Atnarko River | 2 |  |  |  |  |  |  |  |  |  |  |  |  |
|  | 3 |  | 1 | 2 | 6 | 2 |  |  |  |  |  |  |  |
|  | 4 |  | 4 | 9 | 29 | 7 | 1 |  |  |  |  |  |  |
|  | 5 |  | 5 | 2 | 11 | 1 |  |  |  |  |  |  |  |
| Wannock | 3 |  |  |  |  |  | 1 |  |  |  |  |  |  |
| Phillips River | 3 |  | 1 | 1 |  |  | 1 |  |  |  |  |  |  |
|  | 4 |  | 2 |  |  | 3 | 2 | 1 |  |  |  |  |  |
|  | 5 |  |  |  |  |  | 1 |  |  |  |  |  |  |
|  | 6 |  | 1 |  |  |  |  |  |  |  |  |  |  |
| Ashlu River | 3 |  |  |  |  |  |  |  |  |  |  | 1 |  |
| Mamquam River | 3 |  |  |  |  |  |  | 1 |  |  | 1 | 2 |  |
| Tenderfoot Creek | 3 |  |  |  |  |  |  | 2 | 2 |  |  | 1 |  |
| Capilano River | 2 |  |  |  |  |  |  |  | 1 |  |  | 1 |  |
|  | 3 |  | 1 |  |  | 2 | 4 | 42 | 1 | 3 | 43 | 3 | 1 |
|  | 4 |  |  |  |  |  | 1 | 5 |  |  | 12 |  |  |
| Shuswap River lower | 2 |  |  |  |  |  |  |  |  |  |  | 1 |  |
|  | 3 |  | 4 | 1 |  |  | 1 | 5 | 1 | 1 | 3 | 1 |  |
| Chilko River | 3 |  |  |  |  |  |  |  |  |  |  | 1 |  |
|  | 4 |  |  |  |  |  |  |  |  |  |  |  |  |
| Nicola River | 4 |  |  |  |  |  |  | 1 |  |  | 1 |  |  |
| Harrison River | 2 |  |  |  |  |  |  |  |  |  |  | 1 |  |
|  | 3 |  |  |  |  |  |  | 14 | 1 |  | 4 |  |  |
|  | 4 |  |  |  |  |  |  | 1 |  |  | 1 |  |  |
| Chilliwack River | 2 |  |  |  |  |  |  |  | 5 |  |  | 22 |  |
|  | 3 |  | 1 |  |  | 2 | 1 | 140 | 2 | 5 | 77 | 6 | 3 |
|  | 4 |  |  |  |  | 1 |  | 16 |  | 1 | 9 |  | 1 |
|  | 5 |  |  |  |  |  |  |  |  | 1 |  |  |  |
| Nimpkish River | 3 |  |  |  |  |  |  |  |  | 1 |  |  |  |
| Quinsam River | 2 |  |  |  |  |  |  |  | 3 |  |  |  |  |
|  | 3 |  | 9 | 1 |  | 5 | 15 | 7 | 1 |  |  |  |  |
|  | 4 |  | 16 | 2 |  | 11 | 27 | 9 |  | 4 | 2 |  |  |
|  | 5 |  | 5 | 1 |  | 1 | 1 | 1 |  | 1 |  |  |  |
|  | 6 |  | 1 |  |  |  |  |  |  |  |  |  |  |
| Puntledge River summer | 2 |  |  |  |  |  |  |  | 1 |  |  |  |  |
|  | 3 |  |  |  |  |  |  | 1 | 2 |  |  | 1 |  |
|  | 4 |  |  |  |  |  | 1 | 1 |  |  | 1 |  |  |
| Puntledge River fall | 2 |  |  |  |  | 1 |  | 1 | 28 |  | 1 | 8 |  |
|  | 3 | 2 | 8 | 2 |  | 4 | 24 | 134 | 10 | 8 | 11 |  |  |
|  | 4 |  |  |  |  | 4 | 10 | 9 |  | 1 |  |  |  |
| Qualicum River | 2 | 1 |  |  |  |  |  | 1 | 18 | 1 |  | 11 |  |
|  | 3 |  | 5 |  |  | 3 | 2 | 41 | 3 | 1 | 6 | 1 | 1 |
|  | 4 |  | 1 |  |  | 1 | 3 | 16 |  |  | 1 |  | 1 |
|  | 5 |  |  |  |  |  |  |  |  | 1 |  |  |  |
| Cowichan River | 2 |  | 1 |  |  | 1 |  |  | 1 |  |  |  |  |
|  | 3 |  |  |  |  |  | 1 | 11 | 3 | 2 | 5 |  |  |
|  | 4 |  | 1 |  |  |  | 4 | 9 |  |  | 2 |  |  |
| Burman River | 4 |  | 2 | 1 |  | 1 | 12 |  |  |  |  |  |  |
|  | 5 |  | 1 |  |  |  | 2 |  |  |  |  |  |  |
| Conuma River | 5 |  |  | 1 |  |  | 2 |  |  |  |  |  |  |
| Leiner River | 5 |  |  |  |  |  |  |  |  |  |  |  |  |
| Bedwell River | 3 |  | 1 |  |  |  |  |  |  |  |  |  |  |
|  | 4 |  | 2 | 1 |  | 1 | 12 |  |  |  |  |  |  |
| Nahmint River | 3 |  |  |  |  |  | 1 |  |  |  |  |  |  |
|  | 4 |  |  |  |  |  |  |  |  |  |  |  |  |
| Nitinat River | 3 |  | 1 | 2 |  | 2 | 2 |  |  |  |  |  |  |
|  | 4 |  | 3 | 3 |  |  |  |  |  |  |  |  |  |
|  | 5 |  |  |  |  |  |  |  |  |  |  |  |  |
| Robertson Creek | 2 | 1 |  |  |  |  |  |  |  |  |  |  |  |
|  | 3 | 7 | 11 | 1 |  | 8 | 19 | 1 |  |  |  |  |  |
|  | 4 | 21 | 80 | 8 |  | 28 | 152 |  |  |  |  |  |  |
|  | 5 |  | 3 |  |  |  | 6 |  |  |  |  |  |  |
| Sarita River | 3 |  |  |  |  | 1 |  |  |  |  |  |  |  |
|  | 4 |  | 3 |  |  | 2 | 6 |  |  |  |  |  |  |
|  | 5 |  |  |  |  |  |  |  |  |  |  |  |  |
| Thornton Creek | 3 |  |  |  |  |  |  |  |  |  |  |  |  |
|  | 4 |  | 1 | 1 |  |  | 2 |  |  |  |  |  |  |

Supplementary Table S7 concluded

|  |  |  | | Fishery | | | | | | | | |
| --- | --- | --- | --- | --- | --- | --- | --- | --- | --- | --- | --- | --- |
| Population | Age | JDF sport | WCVI sport | | Taaq-wihaak WCVI troll | WCVI troll | Area 23 net | Area 25 net | Mququin test | Area 12 test | Skeena test | Total |
| N |  | 302 | 4,131 | | 138 | 138 | 122 | 197 | 332 | 59 | 422 | 12,533 |
| N PBT |  | 10 | 941 | | 15 | 4 | 101 | 3 | 72 | 11 | 11 | 2,702 |
| Yakoun River | 3 |  |  | |  |  |  |  |  |  |  | 2 |
| Kitsumkalum | 3 |  |  | |  |  |  |  |  |  | 2 | 3 |
|  | 4 |  |  | |  |  |  |  |  |  | 6 | 9 |
|  | 5 |  |  | |  |  |  |  |  |  | 3 | 7 |
|  | 6 |  |  | |  |  |  |  |  |  | 1 | 2 |
| Kitimat River | 3 |  |  | |  |  |  |  |  |  |  | 7 |
| Atnarko River | 2 |  |  | |  |  |  |  | 1 |  |  | 1 |
|  | 3 |  |  | |  |  |  |  |  |  |  | 11 |
|  | 4 |  | 4 | |  |  |  |  |  |  |  | 54 |
|  | 5 |  |  | |  |  |  |  |  |  |  | 19 |
| Wannock | 3 |  |  | |  |  |  |  |  |  |  | 1 |
| Phillips River | 3 |  |  | |  |  |  |  |  | 1 |  | 4 |
|  | 4 |  |  | |  |  |  |  |  |  |  | 8 |
|  | 5 |  |  | |  |  |  |  |  |  |  | 1 |
|  | 6 |  |  | |  |  |  |  |  |  |  | 1 |
| Ashlu River | 3 |  |  | |  |  |  |  |  |  |  | 1 |
| Mamquam River | 3 |  |  | |  |  |  |  |  |  |  | 4 |
| Tenderfoot Creek | 3 |  |  | |  |  |  |  |  |  |  | 5 |
| Capilano River | 2 |  |  | |  |  |  |  |  |  |  | 2 |
|  | 3 |  | 10 | | 1 |  |  |  |  |  |  | 111 |
|  | 4 |  |  | |  |  |  |  |  |  |  | 18 |
| Shuswap River lower | 2 |  |  | |  |  |  |  |  |  |  | 1 |
|  | 3 |  | 1 | |  |  |  |  |  | 1 |  | 19 |
| Chilko River | 3 |  |  | |  |  |  |  |  |  |  | 1 |
|  | 4 |  | 1 | |  |  |  |  |  |  |  | 1 |
| Nicola River | 4 |  |  | |  |  |  |  |  |  |  | 2 |
| Harrison River | 2 |  | 1 | |  |  |  |  |  |  |  | 2 |
|  | 3 |  | 1 | |  |  |  |  |  | 1 |  | 21 |
|  | 4 |  |  | |  |  |  |  |  |  |  | 2 |
| Chilliwack River | 2 |  |  | |  |  |  |  |  | 1 |  | 28 |
|  | 3 | 3 | 12 | | 1 | 3 |  |  |  | 4 |  | 260 |
|  | 4 |  | 6 | |  |  |  |  |  |  |  | 34 |
|  | 5 |  |  | |  |  |  |  |  |  |  | 1 |
| Nimpkish River | 3 |  |  | |  |  |  |  |  |  |  | 1 |
| Quinsam River | 2 |  | 1 | |  |  |  |  |  |  |  | 4 |
|  | 3 |  |  | |  |  |  |  |  |  |  | 38 |
|  | 4 |  | 9 | |  |  |  |  | 1 |  |  | 81 |
|  | 5 |  | 1 | |  |  |  |  |  |  |  | 11 |
|  | 6 |  |  | |  |  |  |  |  |  |  | 1 |
| Puntledge River summer | 2 |  |  | |  |  |  |  |  |  |  | 1 |
|  | 3 |  |  | |  |  |  |  |  |  |  | 4 |
|  | 4 |  |  | |  |  |  |  |  |  |  | 3 |
| Puntledge River fall | 2 |  | 1 | |  |  |  |  |  |  |  | 40 |
|  | 3 |  | 2 | |  |  |  |  |  | 1 |  | 206 |
|  | 4 | 1 | 4 | |  |  |  |  |  |  |  | 29 |
| Qualicum River | 2 |  |  | |  |  |  |  |  |  |  | 32 |
|  | 3 |  | 1 | |  |  |  |  |  |  |  | 64 |
|  | 4 |  | 2 | |  |  |  |  | 1 |  |  | 26 |
|  | 5 |  | 1 | |  |  |  |  |  |  |  | 2 |
| Cowichan River | 2 |  |  | |  |  |  |  |  |  |  | 3 |
|  | 3 |  | 1 | |  |  |  |  |  |  |  | 23 |
|  | 4 |  | 2 | |  |  |  |  |  |  |  | 18 |
| Burman River | 4 |  | 48 | |  |  |  |  | 5 |  |  | 71 |
|  | 5 |  | 5 | |  |  |  |  |  |  |  | 8 |
| Conuma River | 5 |  | 5 | |  |  |  |  |  |  |  | 9 |
| Leiner River | 5 |  | 4 | |  |  |  |  | 1 |  |  | 5 |
| Bedwell River | 3 |  | 1 | |  |  |  |  |  |  |  | 2 |
|  | 4 |  | 48 | |  |  |  | 2 | 4 |  |  | 71 |
| Nahmint River | 3 |  |  | |  |  |  |  |  |  |  | 1 |
|  | 4 |  | 7 | |  |  |  |  |  |  |  | 7 |
| Nitinat River | 3 |  | 7 | |  |  |  |  |  |  |  | 14 |
|  | 4 |  | 8 | |  |  |  |  |  |  |  | 14 |
|  | 5 |  | 1 | |  |  |  |  |  |  |  | 1 |
| Robertson Creek | 2 | 1 | 4 | |  |  |  |  |  |  |  | 6 |
|  | 3 | 2 | 116 | | 5 |  | 8 |  | 12 | 2 |  | 192 |
|  | 4 | 3 | 603 | | 7 |  | 93 |  | 49 |  |  | 1,044 |
|  | 5 |  | 12 | |  |  |  |  | 1 |  |  | 22 |
| Sarita River | 3 |  | 9 | |  |  |  |  |  |  |  | 10 |
|  | 4 |  | 25 | |  |  |  |  |  |  |  | 36 |
|  | 5 |  | 3 | |  |  |  |  |  |  |  | 3 |
| Thornton Creek | 3 |  | 6 | |  |  |  |  |  |  |  | 6 |
|  | 4 |  | 15 | |  | 1 |  |  | 1 |  |  | 21 |

Supplementary Table S8. Age distributions of Chinook salmon marine fishery catch in British Columbia in 2018 and 2019 derived from coded-wire tags (CWT) and parentage-based tagging (PBT).

| Population | Age | 2018 | | 2019 | |
| --- | --- | --- | --- | --- | --- |
|  |  | CWT | PBT | CWT | PBT |
|  |  |  |  |  |  |
| n |  | 50 | 7 | 41 | 21 |
| Kitsumkalum | 2 | 0.0 | 0.0 | 0.7 | 0.0 |
|  | 3 | 1.4 | 0.0 | 0.7 | 14.3 |
|  | 4 | 24.8 | 28.6 | 51.8 | 42.9 |
|  | 5 | 72.8 | 71.4 | 26.6 | 33.3 |
|  | 6 | 0.0 | 0.0 | 20.2 | 9.5 |
| n |  |  |  | 114 | 40 |
| Atnarko River lower | 2 |  |  | 0.0 | 2.5 |
|  | 3 |  |  | 19.0 | 22.5 |
|  | 4 |  |  | 63.5 | 70.0 |
|  | 5 |  |  | 17.5 | 5.0 |
| n |  |  |  | 93 | 45 |
| Atnarko River upper | 3 |  |  | 10.3 | 4.4 |
|  | 4 |  |  | 62.5 | 57.8 |
|  | 5 |  |  | 27.2 | 37.8 |
|  |  |  |  | CWT | PBT |
| n |  |  |  | 32 | 14 |
| Phillips River | 3 |  |  | 56.9 | 28.7 |
|  | 4 |  |  | 31.7 | 57.1 |
|  | 5 |  |  | 3.5 | 7.1 |
|  | 6 |  |  | 7.9 | 7.1 |
| n |  | 252 | 21 | 174 | 19 |
| Shuswap River lower | 2 | 0.5 | 0.0 | 1.0 |  |
|  | 3 | 5.6 | 14.3 | 58.6 | 100.0 |
|  | 4 | 85.1 | 85.7 | 38.4 | 0.0 |
|  | 5 | 8.8 | 0.0 | 2.0 | 0.0 |
| n |  | 59 | 9 | 82 | 24 |
| Harrison River | 2 | 0.0 | 0.0 | 0.0 | 4.2 |
|  | 3 | 44.6 | 77.8 | 91.7 | 87.5 |
|  | 4 | 55.4 | 22.2 | 8.3 | 8.3 |
| n |  | 287 | 131 | 359 | 296 |
| Chilliwack River | 2 | 5.3 | 1.5 | 3.9 | 0.4 |
|  | 3 | 80.1 | 81.7 | 84.3 | 87.8 |
|  | 4 | 14.6 | 16.8 | 11.8 | 11.5 |
|  | 5 | 0.0 | 0.0 | 0.0 | 0.3 |
| n |  | 203 | 69 | 234 | 132 |
| Quinsam River | 2 | 0.6 | 0.0 | 0.6 | 0.8 |
|  | 3 | 33.2 | 49.3 | 23.8 | 28.8 |
|  | 4 | 47.4 | 36.2 | 64.7 | 61.4 |
|  | 5 | 18.8 | 14.5 | 10.6 | 8.3 |
|  | 6 | 0.0 | 0.0 | 0.3 | 0.8 |
| n |  | 30 | 58 | 14 | 238 |
| Puntledge River fall | 2 | 11.2 | 1.7 | 7.1 | 1.2 |
|  | 3 | 49.5 | 74.1 | 64.6 | 86.6 |
|  | 4 | 39.3 | 24.1 | 28.3 | 12.2 |
| n |  | 44 | 50 | 39 | 94 |
| Big Qualicum River | 2 | 10.7 | 0.0 | 0.0 | 2.1 |
|  | 3 | 32.1 | 62.0 | 78.4 | 68.1 |
|  | 4 | 55.0 | 38.0 | 21.6 | 27.7 |
|  | 5 | 0.0 | 0.0 | 0.0 | 2.1 |
| n |  | 241 | 34 | 122 | 43 |
| Cowichan River | 2 | 0.7 | 2.9 | 2.2 | 4.6 |
|  | 3 | 60.7 | 73.5 | 62.8 | 53.5 |
|  | 4 | 38.6 | 23.5 | 35.0 | 41.9 |
| n |  |  |  | 31 | 73 |
| Bedwell River | 3 |  |  | 12.4 | 2.7 |
|  | 4 |  |  | 87.6 | 97.3 |
| n |  | 139 | 5 | 137 | 8 |
| Nahmint River | 2 | 0.4 | 0.0 | 0.0 | 0.0 |
|  | 3 | 74.5 | 60.0 | 14.3 | 12.5 |
|  | 4 | 4.4 | 40.0 | 85.2 | 87.5 |
|  | 5 | 20.4 | 0.0 | 0.5 | 0.0 |
| n |  |  |  | 20 | 29 |
| Nitinat River | 3 |  |  | 100.0 | 48.3 |
|  | 4 |  |  |  | 48.3 |
|  | 5 |  |  |  | 3.4 |
| n |  | 1,283 | 459 | 2,158 | 1,164 |
| Robertson Creek | 2 | 0.2 | 0.2 | 0.4 | 0.5 |
|  | 3 | 58.9 | 53.2 | 13.3 | 15.8 |
|  | 4 | 36.5 | 39.7 | 84.4 | 81.8 |
|  | 5 | 4.1 | 6.9 | 1.9 | 1.9 |
|  | 6 | 0.3 | 0.0 | 0.0 | 0.0 |

Supplementary Table S9. Origin and age determined via PBT of individuals included in 2018 hatchery broodstocks. Percentage not assigned is the percentage of the 2018 hatchery broodstock fish that could not be assigned via PBT to any hatchery broodstock genotyped in either 2013-2016.

| Population | Brood Received | Brood Genotyped | Assigned to | Number Assigned (# 2-parent assignments) | % assigned | % not assigned |
| --- | --- | --- | --- | --- | --- | --- |
| Nahmint | 24 | 19 | 2015 Nahmint | 8 (8) | 42.1 | 52.6 |
|  |  |  | 2013 Nahmint | 1 (1) | 5.3 |  |
| Nicola | 150 | 147 | 2015 Nicola | 2 (2) | 1.4 | 55.8 |
|  |  |  | 2014 Nicola | 63 (60) | 42.9 |  |
| Nitinat | 700 | 685 | 2015 Nitinat | 6 (6) | 0.9 | 95.8 |
|  |  |  | 2014 Nitinat | 22 (22) | 3.2 |  |
|  |  |  | 2013 Sarita | 1 (1) | 0.1 |  |
| Phillips | 33 | 32 | No assign | 0 (0) | 0.0 | 100.0 |
| Harrison | 245 | 212 | No assign | 0 (0) | 0.0 | 100.0 |
| Puntledge Summer | 288 | 286 | 2016 Puntledge Summer | 1 (1) | 0.3 | 11.5 |
|  |  |  | 2015 Puntledge Summer | 91 (84) | 31.8 |  |
|  |  |  | 2014 Puntledge Summer | 149 (144) | 52.1 |  |
|  |  |  | 2016 Puntledge Fall | 1 (1) | 0.3 |  |
|  |  |  | 2015 Puntledge Fall | 10 (10) | 3.5 |  |
|  |  |  | 2014 Puntledge Fall | 1 (1) | 0.3 |  |
| Puntledge Fall | 822 | 815 | 2015 Puntledge Summer | 3 (3) | 0.4 | 27.9 |
|  |  |  | 2014 Puntledge Summer | 4 (4) | 0.5 |  |
|  |  |  | 2016 Puntledge Fall | 8 (8) | 1.0 |  |
|  |  |  | 2015 Puntledge Fall | 388 (370) | 47.6 |  |
|  |  |  | 2014 Puntledge Fall | 181 (146) | 22.2 |  |
|  |  |  | 2013 Puntledge Fall | 1 (1) | 0.1 |  |
|  |  |  | 2015 Quinsam | 1 (0) | 0.1 |  |
|  |  |  | 2014 Quinsam | 1 (1) | 0.1 |  |
|  |  |  | 2015 Robertson | 1 (1) | 0.1 |  |
| Big Qualicum | 2029 | 1925 | 2015 Cowichan | 1(1) | 0.1 | 57.1 |
|  |  |  | 2015 Puntledge Fall | 1 (1) | 0.1 |  |
|  |  |  | 2016 Qualicum | 7 (5) | 0.4 |  |
|  |  |  | 2015 Qualicum | 429 (396) | 22.3 |  |
|  |  |  | 2014 Qualicum | 381 (339) | 19.8 |  |
|  |  |  | 2013 Qualicum | 5 (5) | 0.3 |  |
|  |  |  | 2015 Quinsam | 1 (1) | 0.1 |  |
|  |  |  | 2014 Robertson | 1 (1) | 0.1 |  |
| Sarita | 224 | 210 | 2015 Nahmint | 1 (1) | 0.5 | 17.1 |
|  |  |  | 2015 Robertson | 1 (1) | 0.5 |  |
|  |  |  | 2016 Sarita | 13 (13) | 6.2 |  |
|  |  |  | 2015 Sarita | 106 (106) | 50.5 |  |
|  |  |  | 2014 Sarita | 35 (35) | 16.7 |  |
|  |  |  | 2013 Sarita | 18 (18) | 8.6 |  |
| Shushwap Lower | 180 | 180 | 2014 Shushwap Lower | 6 (6) | 3.3 | 96.7 |
| Shushwap Middle | 102 | 102 | 2015 Shushwap Middle | 1 (1) | 1.0 | 95.1 |
|  |  |  | 2014 Shushwap Middle | 4 (4) | 3.9 |  |
| Atnarko | 1045 | 989 | 2015 Atnarko | 48 (43) | 4.9 | 66.5 |
|  |  |  | 2014 Atnarko | 206 (169) | 20.8 |  |
|  |  |  | 2013 Atnarko | 77 (73) | 7.8 |  |
| Capilano (Chilliwack brood) | 433 | 399 | 2015 Chilliwack | 204 (181) | 51.1 | 42.9 |
|  |  |  | 2014 Chilliwack | 24 (24) | 6.0 |  |
| Chilliwack | 973 | 967 | 2016 Chilliwack | 23 (23) | 2.4 | 51.4 |
|  |  |  | 2015 Chilliwack | 377 (354) | 39.0 |  |
|  |  |  | 2014 Chilliwack | 70 (67) | 7.2 |  |
| Cowichan | 334 | 305 | 2016 Cowichan | 3 (3) | 1.0 | 87.5 |
|  |  |  | 2015 Cowichan | 26 (22) | 8.5 |  |
|  |  |  | 2014 Cowichan | 9 (9) | 3.0 |  |
| Thornton | 38 | 38 | 2015 Thornton | 7 (7) | 18.4 | 81.6 |
| Quinsam | 1415 | 1372 | 2015 Quinsam | 75 (71) | 5.5 | 26.2 |
|  |  |  | 2014 Quinsam | 702 (689) | 51.2 |  |
|  |  |  | 2013 Quinsam | 234 (231) | 17.1 |  |
|  |  |  | 2015 Robertson | 1 (1) | 0.1 |  |
| Robertson | 3820 | 3709 | 2015 Robertson | 313 (303) | 8.4 | 73.5 |
|  |  |  | 2014 Robertson | 580 (562) | 15.6 |  |
|  |  |  | 2013 Robertson | 90 (90) | 2.4 |  |

Supplementary Table S10. Origin and age determined via PBT of individuals included in 2019 hatchery broodstocks. Percentage not assigned is the percentage of the 2019 hatchery broodstock fish that could not be assigned via PBT to any hatchery broodstock genotyped in either 2013-2017.

| Population | Brood Received | Brood Genotyped | Assigned to | Number Assigned (# 2-parent assignments) | % assigned | % not assigned |
| --- | --- | --- | --- | --- | --- | --- |
| Nahmint | 35 | 35 | 2016 Nahmint | 3 (2) | 8.6 | 40.0 |
|  |  |  | 2015 Nahmint | 18 (18) | 51.4 |  |
| Nicola | 162 | 162 | 2016 Spius | 3 (3) | 1.9 | 73.5 |
|  |  |  | 2015 Coldwater | 4 (4) | 2.5 |  |
|  |  |  | 2015 Nicola | 35 (35) | 21.6 |  |
|  |  |  | 2014 Nicola | 1 (1) | 0.6 |  |
| Nitinat | 2749 | 2724 | 2016 Nitinat | 10 (10) | 0.4 | 97.8 |
|  |  |  | 2015 Nitinat | 36 (36) | 1.3 |  |
|  |  |  | 2014 Nitinat | 1 (1) | 0.0 |  |
|  |  |  | 2015 Robertson | 11 (8) | 0.4 |  |
|  |  |  | 2016 Sarita | 1 (1) | 0.0 |  |
| Phillips | 41 | 41 | 2016 Phillips | 10 (10) | 24.4 | 68.3 |
|  |  |  | 2015 Phillips | 3 (3) | 7.3 |  |
| Harrison | 248 | 242 | No assign | NA | 0.0 | 100.0 |
| Puntledge Summer | 153 | 150 | 2017 Puntledge Summer | 1 (1) | 0.7 | 27.3 |
|  |  |  | 2016 Puntledge Summer | 20 (20) | 13.3 |  |
|  |  |  | 2015 Puntledge Summer | 80 (80) | 53.3 |  |
|  |  |  | 2014 Puntledge Summer | 1 (1) | 0.7 |  |
|  |  |  | 2016 Puntledge Fall | 1 (1) | 0.7 |  |
|  |  |  | 2015 Puntledge Fall | 6 (6) | 4.0 |  |
| Puntledge Fall | 968 | 968 | 2017 Puntledge Summer | 3 (3) | 0.3 | 28.5 |
|  |  |  | 2016 Puntledge Summer | 1 (1) | 0.1 |  |
|  |  |  | 2015 Puntledge Summer | 9 (9) | 0.9 |  |
|  |  |  | 2017 Puntledge Fall | 18 (1) | 1.9 |  |
|  |  |  | 2016 Puntledge Fall | 384 (361) | 39.7 |  |
|  |  |  | 2015 Puntledge Fall | 276 (265) | 28.5 |  |
|  |  |  | 2017 Robertson | 1 (0) | 0.1 |  |
| Big Qualicum | 1749 | 1732 | 2016 Cowichan | 1 (1) | 0.1 | 67.0 |
|  |  |  | 2015 Cowichan | 1 (1) | 0.1 |  |
|  |  |  | 2016 Puntledge Fall | 4 (4) | 0.2 |  |
|  |  |  | 2015 Puntledge Fall | 2 (2) | 0.1 |  |
|  |  |  | 2017 Qualicum | 15 (15) | 0.9 |  |
|  |  |  | 2016 Qualicum | 224 (164) | 12.9 |  |
|  |  |  | 2015 Qualicum | 315 (300) | 18.2 |  |
|  |  |  | 2014 Qualicum | 6 (6) | 0.3 |  |
|  |  |  | 2016 Quinsam | 1 (1) | 0.1 |  |
|  |  |  | 2015 Quinsam | 2 (2) | 0.1 |  |
| Sarita | 296 | 296 | 2014 Nitinat | 1 (1) | 0.3 | 25.7 |
|  |  |  | 2017 Sarita | 8 (8) | 2.7 |  |
|  |  |  | 2016 Sarita | 67 (61) | 22.6 |  |
|  |  |  | 2015 Sarita | 137 (137) | 46.3 |  |
|  |  |  | 2014 Sarita | 7 (7) | 2.4 |  |
| Shushwap Lower | 260 | 260 | 2016 Shushwap Lower | 12 (12) | 4.6 | 94.6 |
|  |  |  | 2015 Shushwap Lower | 2 (2) | 0.8 |  |
| Shushwap Middle | 100 | 99 | 2016 Shushwap Middle | 2 (2) | 2.0 | 98.0 |
| Atnarko | 971 | 946 | 2016 Atnarko | 13 (11) | 1.4 | 68.5 |
|  |  |  | 2015 Atnarko | 223 (202) | 23.6 |  |
|  |  |  | 2014 Atnarko | 61 (56) | 6.5 |  |
| Capilano (Chilliwack brood) | 422 | 413 | 2017 Chilliwack | 6 (6) | 1.5 | 52.3 |
|  |  |  | 2016 Chilliwack | 179 (178) | 43.3 |  |
|  |  |  | 2015 Chilliwack | 12 (12) | 2.9 |  |
| Chilliwack | 1561 | 1514 | 2017 Chilliwack | 19 (16) | 1.3 | 58.2 |
|  |  |  | 2016 Chilliwack | 546 (539) | 36.1 |  |
|  |  |  | 2015 Chilliwack | 68 (68) | 4.5 |  |
| Cowichan | 308 | 307 | 2016 Cowichan | 12 (11) | 3.9 | 94.1 |
|  |  |  | 2015 Cowichan | 6 (6) | 2.0 |  |
| Thornton | 88 | 88 | 2016 Thornton | 15 (14) | 17.0 | 43.2 |
|  |  |  | 2015 Thornton | 35 (26) | 39.8 |  |
| Quinsam | 1350 | 1341 | 2016 Quinsam | 14 (14) | 1.0 | 38.6 |
|  |  |  | 2015 Quinsam | 717 (674) | 53.5 |  |
|  |  |  | 2014 Quinsam | 91 (91) | 6.8 |  |
|  |  |  | 2013 Quinsam | 1 (0) | 0.1 |  |
| Robertson | 4038 | 4032 | 2016 Robertson | 124 (188) | 3.1 | 78.7 |
|  |  |  | 2015 Robertson | 707 (679) | 17.5 |  |
|  |  |  | 2014 Robertson | 27 (27) | 0.7 |  |
| Spius | 42 | 42 | 2015 Coldwater | 4 (4) | 9.5 | 26.2 |
|  |  |  | 2015 Spius | 27 (27) | 64.3 |  |
| Coldwater | 63 | 63 | 2015 Coldwater | 3 (3) | 4.8 | 90.5 |
|  |  |  | 2015 Nicola | 3 (0) | 4.8 |  |
| Kitimat | 620 | 608 | 2014 Atnarko | 1 (1) | 0.2 | 76.2 |
|  |  |  | 2016 Kitimat | 144 (126) | 23.7 |  |
| Bedwell | 30 | 29 | 2016 Bedwell | 4 (3) | 13.8 | 6.9 |
|  |  |  | 2015 Bedwell | 22 (0) | 75.9 |  |
|  |  |  | 2015 Cypre | 1 (0) | 3.4 |  |

List of Supplementary Figures

Supplementary Figure S1. Boundaries of conservation units defined for Chinook salmon in southern British Columbia. The map was prepared with ArcGIS with the shape files located at Open Government at <https://search.open.canada.ca/en/od/?sort=last_modified_tdt%20desc&page=1&od-search-portal=Open%20Data&search_text=NuSEDS>.

Supplementary Figure S2. Boundaries of conservation units defined for Chinook salmon in northern British Columbia. The map was prepared with ArcGIS with the shape files located at Open Government at <https://search.open.canada.ca/en/od/?sort=last_modified_tdt%20desc&page=1&od-search-portal=Open%20Data&search_text=NuSEDS>.

Supplementary Figure S3. Distribution of expected heterozygosity for 389 SNPs surveyed in Chinook salmon populations ranging from Russia to California.

Supplementary Figure S4. Distribution of F_ST_ for 389 SNPs surveyed in Chinook salmon populations ranging from Russia to California.

Supplementary Figure S5. F_ST_ for each of the 389 SNPs utilized for genetic stock ID – sex ID and species ID amplicons not shown. Results are plotted by position along the chromosome, or evenly spaced for SNPs on unlinked scaffolds. Counts above the plot indicate the number of SNPs per chromosome. Markers with duplicate mapping positions utilize position identified in Supplementary Table 2.

Supplementary Figure S1

Supplementary Figure S2


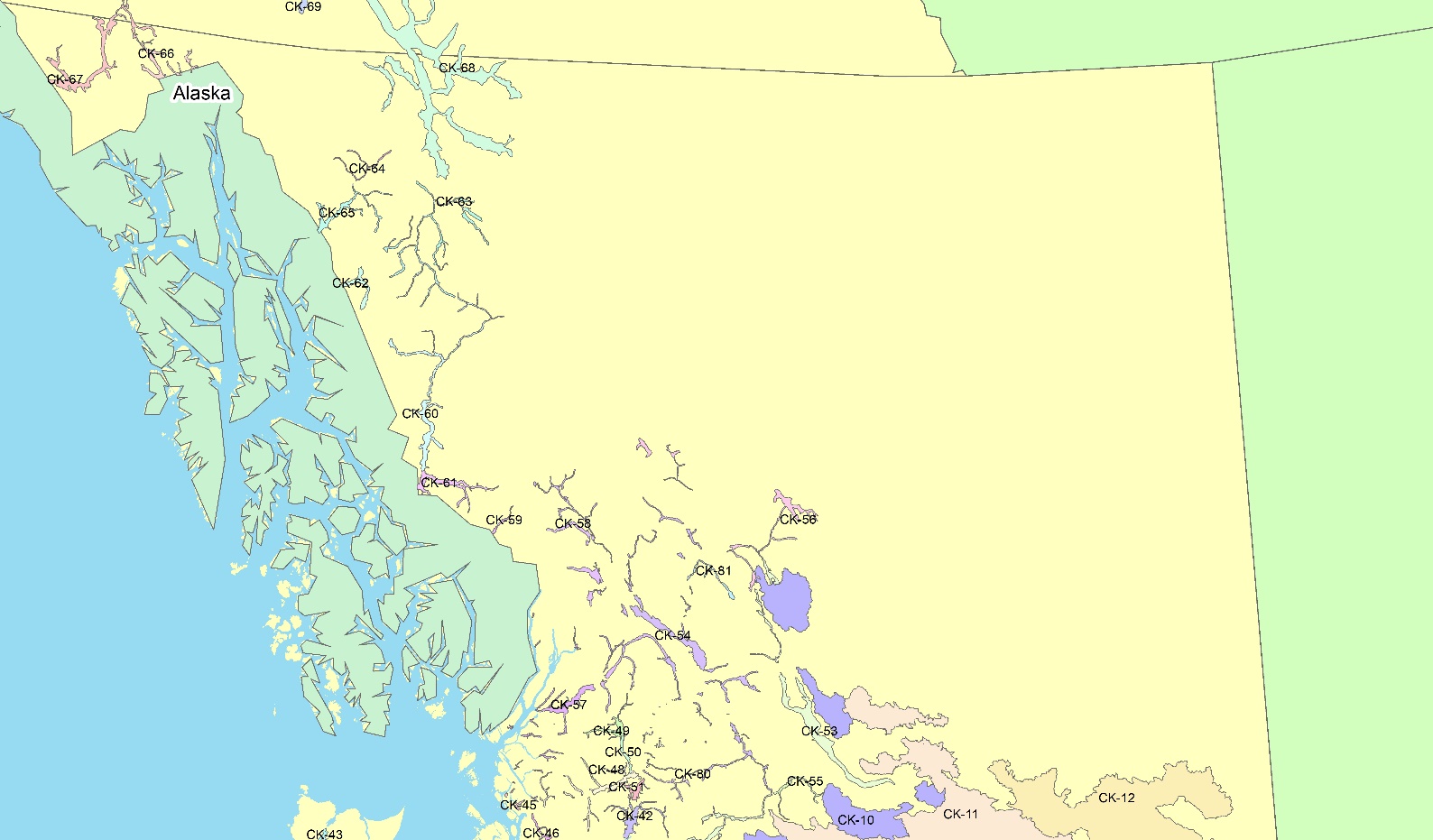


Supplementary Figure S3. Distribution of expected heterozygosity for 389 SNPs surveyed in Chinook salmon populations ranging from Russia to California.


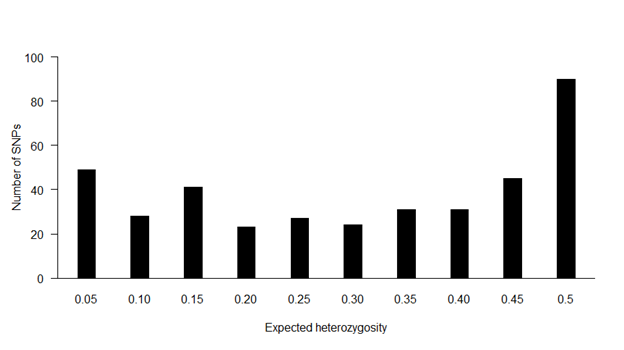


Supplementary Figure S4. Distribution of F_ST_ for 389 SNPs surveyed in Chinook salmon populations ranging from Russia to California.


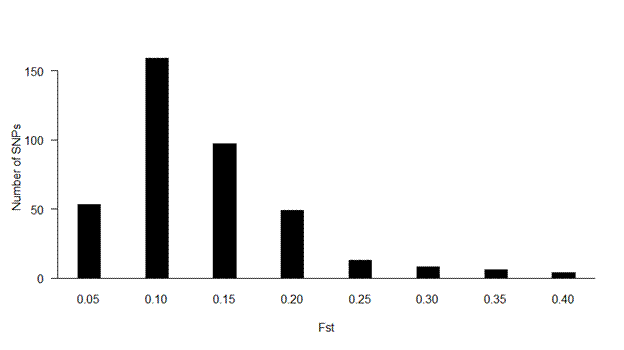


Supplementary Figure S5. F_ST_ for each of the 389 SNPs utilized for genetic stock ID – sex ID and species ID amplicons not shown. Results are plotted by position along the chromosome, or evenly spaced for SNPs on unlinked scaffolds. Counts above the plot indicate the number of SNPs per chromosome. Markers with duplicate mapping positions utilize position identified in Supplementary Table 2.


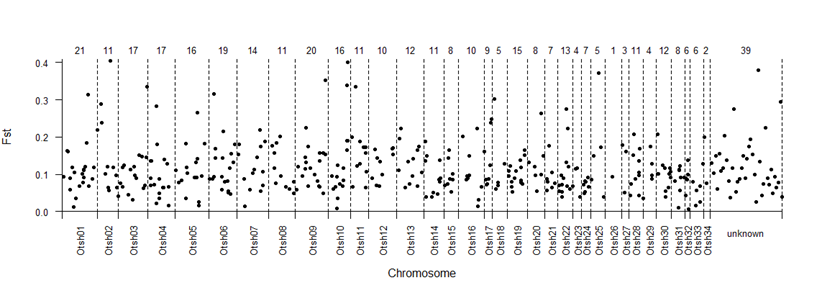

Supplement: Supplementary file 1 — Supplementary Material [file EVA-14-1365-s001.docx]
